# Estimating Permeabilities from Biased Molecular Dynamics with a Green–Kubo Formalism for Transport across the Blood-Brain Barrier

**DOI:** 10.1021/acs.jcim.6c01624

**Published:** 2026-07-21

**Authors:** Christian Jorgensen

**Affiliations:** School of Medicine, Pharmacy and Biomedical Sciences, Faculty of Science & Health, 6697 University of Portsmouth, Portsmouth PO1 2DT, Hampshire, U.K.

## Abstract

In this work, we explore the uses of biased sampling simulations including constant-velocity SMD (cv-SMD) and umbrella sampling to study molecular permeation of three highly distinct solutes spanning over 3 orders of magnitude in the permeability space, across a model of the blood-brain barrier (BBB). These molecules range from among the slowest permeants known (temozolomide; *P*_app_ ∼ 10^–6^ cm s^–1^) to among the fastest compound known (ethanol; *P*_app_ ∼ 10^–3^ cm s^–1^). We arrive at the transmembrane solute permeability, *P*_sim_, by the Inhomogeneous Solubility-Diffusion equation combined with an unbiasing procedure involving the Green–Kubo relations, to find a ∼1 order of magnitude agreement with experiments. We find that the converged thermodynamics of crossing span distinct topologies for each of the compounds considered, while noting that there is an apparent relationship between the thermodynamic barrier height (Δ*G* ^‡^) and experimental permeability, *P*_app_ for these molecules.

## Graphical abstract

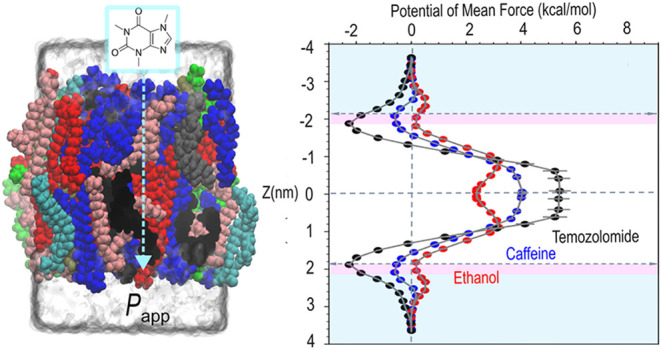

## Introduction

The blood-brain barrier (BBB) restricts the brain from the circulatory system.^1–3^ Routine permeability estimations of therapeutic passage across key biological barriers remain a challenge in the field of drug delivery.^4^ For central nervous system (CNS) delivery, early screening approaches began with the likes of Lipinski’s rule of five,^5^ in which simple cheminformatic properties were to predict if a drug could cross the blood-brain barrier. Unfortunately, many compounds display non-Lipinski behavior, and as such, costly and nonroutine *in vitro* or *in vivo* assessment are required. Recently the emergence of artificial intelligence (AI)-based cheminformatic models to enhance the predictive capabilities for CNS drug delivery^6^ by integrating more complex patterns and interactions, including LightBBB^7^ and the Cliques model.^8^ Such predictive models yield atomic-detail insights into the physical process of solutes crossing biological barriers, which require MD simulations or the likes.^9,10^ As such, the use of tissue-specific permeability (*P*) remains the gold standard both as a precise measure of barrier penetration,^11–13^ as well as a key parameter in the later stages of drug development.^4,14–17^

Recent permeability research has investigated high-throughput approaches, such as the Bereau group, who propose the use of high-throughput coarse-grained (HTCG) simulations to derive a permeability surface in terms of just molecular descriptors, namely the bulk partitioning free energy and p*K*_a_.^18^ Similarly, the same group has demonstrated the use of coarse-grain MD (CG-MD) to evaluate the permeability of hundreds of therapeutics at once.^19^ Other groups have used CG-MD to evaluate a permeability variation based on either a lipophilicity gradient, or an acyl chain length.^20^ These foundational HTS models point us to the need for larger libraries of compounds when evaluating the influence of a parameter on the permeability. However, the HTS approach for atomistic MD (AA-MD) has proven computationally intractable so far for scientists with routine resources.^13^

In this work, we set out to explore the use of constant-velocity SMD (cv-SMD) to study molecular permeation of three highly distinct solutes across a model of the blood-brain barrier. This is a highly heterogeneous membrane modeled by nine lipid components, and as such, requires above average simulation times to ensure the convergence in the potential of mean force (PMF) and sufficient lag times to sample the plateau in transport property correlation assessment, and for which conventional MD simulations would require timescales far greater than those conventionally available. One of the drugs considered is temozolomide, the only FDA approved compound for brain cancer. Gleaning a mechanistic understanding of temozolomide delivery across the BBB has immense potential for improving the success rate for treating glioblastoma.

### Permeability Estimation: The Inhomogeneous Solubility Diffusion (ISD) Theoretical Framework

The ISD equation is one of the most common approaches to calculate the transmembrane permeability *P*_sim_ (cm s^–1^) from biased simulations, as first demonstrated by Marrink et al. for water permeation,^13,21,22^ but is now a routine approach with a variety of enhanced sampling techniques, including replica-exchange methods such as the Replica Exchange Transition Interface Sampling (RETIS) method,^23^ Adaptive Biasing Force (ABF),^24,25^ metadynamics,^26^ and umbrella sampling.^27^

In the ISD equation (eq 1), the transmembrane permeability *P*_sim_ is calculated by solving the Smoluchowski equation under stationary conditions. This requires inputting the position-dependent diffusion *D*(*z*) (cm^2^ s^–1^) and free energy Δ*G*(z) (kcal mol^–1^) along the transition coordinate *z*, as described in eq 1, in which *RT* denote the product of the molar gas constant, *R*, and the temperature, *T*

1 $$\frac{1}{P_{\mathrm{sim}}}=\int_{z_{0}}^{z_{1}}\frac{\exp\left(\Delta G\left(z\right)/\mathrm{RT}\right)}{D\left(z\right)}dz$$

One of the reasons there have not been routine HTS permeability evaluations with the ISD/AA-MD approach is the recognized difficulty to converge such calculations for heterogeneous membranes.^4,22,24,28^ The first component of the ISD equation, the PMF Δ*G*(*z*), can be evaluated with a variety of methods described above. For the second component, the diffusion coefficients *D*(*z*), methods such as Bayesian inference^29,30^ have been employed. When using the ISD framework with ABF, a posthoc Bayesian inference is needed to obtain the kinetics.^24,25^ When using the ISD framework with umbrella sampling,^31^ the PMF is evaluated with WHAM,^32^ while the permeability^33^ has been calculated with DHAM.^33,34^

### Permeability Estimation: Applying the ISD/AA-MD Framework with cv-SMD and the Green–Kubo Equations

In neat solvents, diffusion coefficients can be calculated from the mean squared displacement (via the Einstein–Smoluchowski equations)^35–38^ or from velocity autocorrelation functions (via Green–Kubo relations; eq 5).^39,40^ We argue here that the latter procedure’s use of autocorrelation function is tantamount to an unbiasing procedure, along the same lines of the Bayesian posthoc correction required to access kinetics from ABF. Indeed, the use of the Green–Kubo relations requires that the solute be constrained to a point *i* in *z*, as first described by Marrink,^41^ and this bias is then corrected for by estimating the autocorrelation function of the biasing force in time. The first component, the PMF Δ*G*(z), can be calculated by numerical integration of the average *z* component of the force *F_z_* of the constrained molecule along the pulled distance *z*_0_ to *z*,^42,43^ as per eq 2

2 $$\Delta G\left(z\right)=G\left(z\right)-G\left(z_{0}\right)=-\int_{z_{0}}^{z}\left\langle F_{z}\left(z^{\prime }\right)\right\rangle dz^{\prime }$$

We first define the unnormalized autocorrelation *C*_*Fz*,*i*_ of the raw force fluctuation Δ*F*_*z*,*i*_ in window *i* obtained from cv-SMD, *F*_*z*,*i*_, for window *i* eq 3

3 $$C_{F,z,i}=\left\langle {\Delta F}_{z}{\left(z,t\right)\Delta F}_{z}\left(z,0\right)\right\rangle$$

The Green–Kubo relations have emerged as a powerful avenue to the diffusion constant^44^ from biased sampling techniques such as SMD, by linking the integral of the force autocorrelation function, *C*_*F*_, to the diffusion constant

4 $$C_{F}=\int_{0}^{\infty }\left\langle {\Delta F}_{z}{\left(z,t\right)\Delta F}_{z}\left(z,0\right)\right\rangle dt$$

The diffusion constant along the *z* pulling coordinate, *D_z_*, is calculated via the Green–Kubo relations eq 5, derived from the Fluctuation–Dissipation Theorem.^45^ In summary, *D_z_* depends on the autocorrelation *C*_*F*_, and (*RT*)^2^ is the square of the macroscopic ideal gas thermal factor

5 $$D_{z}=\frac{{\left(\mathrm{RT}\right)}^{2}}{C_{F}}=\frac{{\left(\mathrm{RT}\right)}^{2}}{\int_{0}^{\infty }\left\langle {\Delta F}_{z}{\left(z,t\right)\Delta F}_{z}\left(z,0\right)\right\rangle dt}$$

A full derivation of eq 5 is provided in SI Extended Methods 1.4 and 1.5. The justification and full unit analysis is provided in the SI Extended Methods Section. In previous research, we have shown that high-temperature simulations can be used to estimate permeabilities at body temperature from direct Arrhenius fits^9,10,46^ or by fitting the rates based on a modified form of Kramer’s theory of reaction rate,^9^ but both procedures suffer from accuracy setbacks due to the nonlinear effects that arise at higher temperatures. There are instances where temperature acceleration is not feasible due to the artifacts it induces, and for this reason in this work we wish to demonstrate the use of the ISD approach with the orthogonal diffusion constant calculated by the Green–Kubo equation by multiμs converged biased sampling trajectories on transport across the blood-brain barrier.

The plateau of the integral of the ACF in eq 5 does not always converge, and for this reason alternative approaches have gained traction. The method of Fitzgerald et al.^47^ involves fitting the early lags of the ACF onto a general exponential that has the same shape as the ACF. This exponential has the form eq 6

6 $$C_{F,z,i}=\left\langle {\Delta F}_{z}\left(z,t\right){\Delta F}_{z}\left(z,0\right)\right\rangle =a\exp\left(-bt^{-c}\right)$$

The advantage of this method is that integrating such a general exponential has an analytical solution based on fitting coefficients (*a*, *b*, *c*) in the form of a gamma function Γ. The solution is *C_F_* eq 7

7 $$C_{F}=\int_{0}^{\infty }\left\langle {\Delta F}_{z}\left(z,t\right){\Delta F}_{z}\left(z,0\right)\right\rangle dt=a\left(b^{-c}\right)\Gamma \left(1+c\right)$$

In Table 1, we provide a summary of physical parameters and variables employed in eqs 1 to 7, as an aid to the reader.

**Table 1:** Summary of Physical Parameters and Variables Used in eqs 1 to 7

Parameter	Units	Equation	Definition
*z*	nm	([Disp-formula eq1]), ([Disp-formula eq2]), ([Disp-formula eq3]), ([Disp-formula eq4]), ([Disp-formula eq5]), ([Disp-formula eq6]) ([Disp-formula eq7])	*z*-axis based reaction coordinate
*z*_*i*_	nm	([Disp-formula eq1]), ([Disp-formula eq2]), ([Disp-formula eq3]), ([Disp-formula eq4]), ([Disp-formula eq5]), ([Disp-formula eq6]) ([Disp-formula eq7])	Reference *z* value or *z* starting value at a point *i*
*i*	None	([Disp-formula eq2]), ([Disp-formula eq3]), ([Disp-formula eq4])	File index, e.g., window *i*. For 40 pulling windows, *i* = {0, ..., 39}. *i* values are discrete such that *i* = 0 ≠ *i* = 1
*P*_sim_	cm s^–1^	([Disp-formula eq1])	Transmembrane permeability
*D*(*z*)	cm^2^ s^–1^	([Disp-formula eq1])	Position-dependent diffusivity
Δ*G*(*z*)	kcal mol^–1^ or kJ mol^–1^	([Disp-formula eq1])	free energy of permeation
⟨*F*_*z*_(*z*′)⟩	J mol^–1^ nm^–1^, kJ mol^–1^ nm^–1^ or kcal mol nm^–1^	([Disp-formula eq2])	Average of the instantaneous force along the *z* coordinate of the permeating drug measured in the SMD or US simulations
Δ*F_z_*	J mol^–1^ nm^–1^, kJ mol^–1^ nm^–1^ or kcal mol nm^–1^	([Disp-formula eq3]), ([Disp-formula eq4]), ([Disp-formula eq5]), ([Disp-formula eq6]), ([Disp-formula eq7])	Raw force fluctuation measured in the SMD or US simulations
*C*_*Fz*,*i*_	(J mol^–1^ nm^–1^)^2^ or J^2^ mol^–2^ nm^–2^	([Disp-formula eq3]), ([Disp-formula eq6])	Unnormalized autocorrelation function of the raw force fluctuation Δ*F_z_* in window *i*
*C*_*F*_	(J mol^–1^ nm^–1^)^2^ ns	([Disp-formula eq4]), ([Disp-formula eq7])	Integral of the force autocorrelation function *C*_*Fz*,*i*_

## Materials and Methods

### A Lipid Bilayer Model of Human Brain Microvascular Endothelial Cells (hBMECs)

An atomic detail molecular model of the apical hBMEC lipid bilayer (Figure 1A) was utilized as first described in previous work,^9^ consisting of a patch of 96 lipids (48 per leaflet) in an area of ∼25 nm^2^.

**Figure 1:**
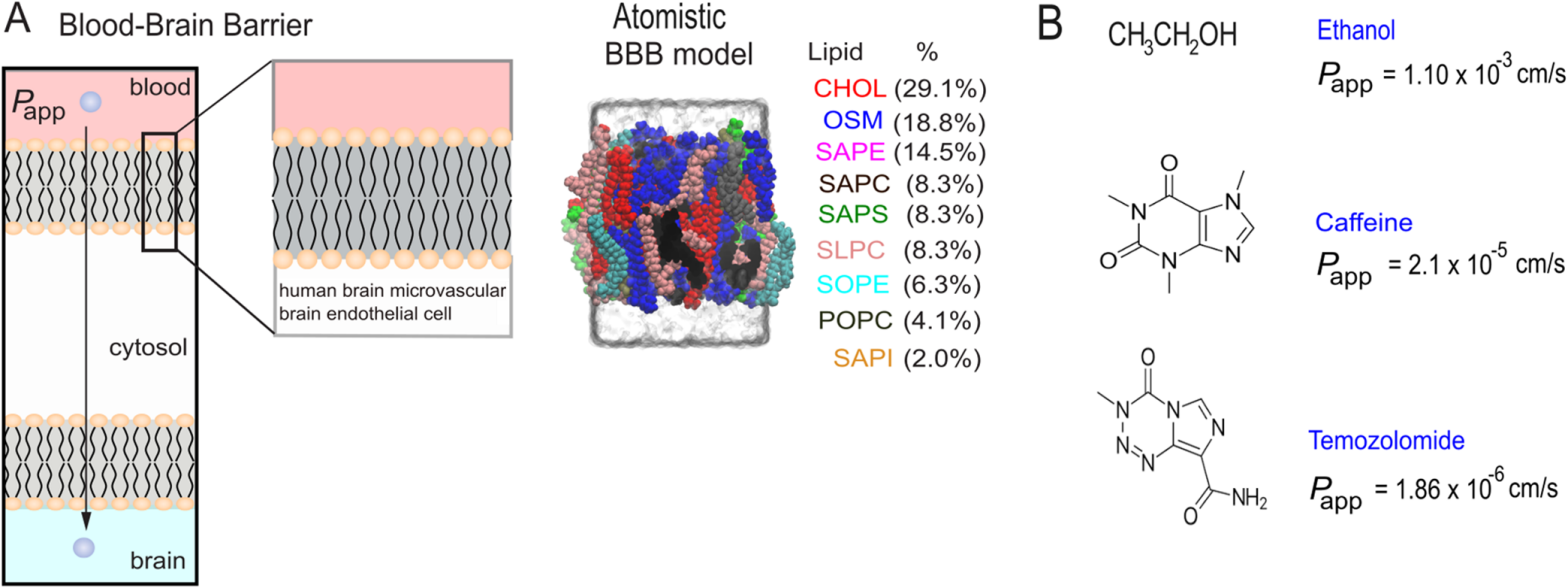
Atomistic model of the endothelial barrier component of the blood-brain barrier and representative CNS compounds. (A) Representation of the human brain microvascular endothelial cell (hBMEC) membrane that forms a key element of the blood-brain barrier (BBB), showing the atomistic model of the apical hBMEC membrane and composition of the atomistic BBB apical hBMEC membrane model (*N* = 96 lipid molecules). (B) Compounds whose permeability was assessed across the BBB with a Green–Kubo formalism: ethanol, caffeine and temozolomide. Ethanol was chosen as the canonical rapidly permeating CNS molecule (*P*_app_ = 1.10 × 10^–3^ cm s^–1^), caffeine was chosen as an example of a prominent CNS molecule with intermediate CNS permeability (*P*_app_ = 2.1 × 10^–5^ cm s^–1^), and temozolomide, a chemotherapeutic and the only FDA approved therapy for glioblastoma, with slow CNS permeability (*P*_app_ = 1.86 × 10^–6^ cm s^–1^).

### CNS Compounds Spanning a Representative Range of Permeabilities

Three molecular solutes of particular interest for CNS drug delivery were chosen (Figure 1B). Ethanol was chosen as the canonical rapidly permeating CNS molecule (*P*_app_ = 1.10 × 10^–3^ cm s^–1^; red blood cell estimate),^48^ caffeine was chosen as an example of a prominent CNS molecule with intermediate CNS permeability (*P*_app_ = 2.1 × 10^–5^ cm s^–1^; MDCK),^9^ and temozolomide, a chemotherapeutic and the only FDA approved therapy for glioblastoma^49,50^ that is also associated with a slow CNS permeability (*P*_app_ = 1.86 × 10^–6^ cm s^–1^; 3D brain perfusion).^51^ These molecules span permeabilities from the slow range of 10^–6^ cm s^–1^ (temozolomide) to the very fast range of 10^–3^ cm s^–1^ (ethanol). Because solute transport is dependent, in part, on the choice of solute force field,^18,33,52–54^ we obtained force field parameters in a standardized fashion using the CGenFF program^55,56^ (version 2.5.1) to obtain bonded and nonbonded parameters via the automated parameter assignment tool (SILCSBIO) of CGenFF. All molecules are simulated in their neutral form, for comparison.

### Biased-Sampling Simulations: Constant-Velocity Steered Molecular Dynamics (cv-SMD) Seeding with Umbrella Sampling Production Runs

The cv-SMD simulations were performed using GROMACS 2024.4 (www.gromacs.org)^57^ with the CHARMM36 all-atom force field for lipids,^58^ the CHARMM general force field (CGenFF) for molecular solutes^59^ and the TIP3P water model as solvent.^60^ Electrostatic interactions were computed using particle-mesh-Ewald (PME),^61^ and a cutoff of 10 Å was used for nonbonded interactions. Bonds involving hydrogen atoms were restrained using LINCS^62^ to permit a 2 fs time-step. Neighbor lists were updated every 5 steps. All simulations were performed in the NPT (constant number of particle, pressure and temperature) ensemble, with lipids coupled separately from the solvent and drug molecule to a heat bath with temperatures at 310 K. A time constant τ_T_ = 0.1 ps was utilized in combination with the velocity rescaling algorithm.^63^ In the equilibration stage, an atmospheric pressure of 1 bar was maintained in the simulation box using the Berendsen semi-isotropic pressure coupling^64^ with compressibility κ*_z_* = κ*_xy_* = 4.5 × 10^–5^ bar^–1^. In the production runs we utilized the stochastic cell rescale (C-rescale) semi-isotropic pressure coupling^65^ with compressibility κ*_xy_* = 4.5 × 10^–5^ bar^–1^ and time constant τ_P_ = 5 ps.

Beginning with equilibrated membrane from prior work^10^ we add a single solute molecule to the water region using VMD.^66^ We pull the solute molecule along a reference coordinate *z*, at a rate of 0.005 nm ps^–1^, by specifying a spring constant *k* using the *pull* code of GROMACS^57^ in the NPT ensemble. Using semi-isotropic pressure coupling we use κ*_xy_* = 4.5 × 10^–5^ bar^–1^ and κ*_z_* = 0 bar^–1^ to prevent the layer thicknesses from fluctuating. The initial windows are generated by a rapid pull, consisting of 40 ps of pulling at 0.005 nm ps^–1^, followed by a short equilibration for 400 ps with a 2 fs time step, with the *z* separation between the solute and the bilayer midplane kept fixed. Each of these windows are separated by ∼0.2 nm and are used a starting point for the production run stage, in which we converge the force *F*_*z*,*i*_ needed to restrain the drug at the point *i*, and which serve as the input into the Green–Kubo relation eq 3. The production run samples the force *F*_*z*,*i*_ at fixed *z* for 500 ns per window, with the average force taken per window, by first ensuring the convergence of the average force from a plateau assessment of the running force. We calculate the average force profile for the last 150 ns of production run per window, discarding 350 ns as equilibration. For a 0.2 nm window spacing we require ∼40 windows, meaning the full production run comprises ∼20 μs per drug. The PMF in Figure 2 is calculated from eq 2 by integration of the average force profile depicted in Figure 3.

**Figure 2:**
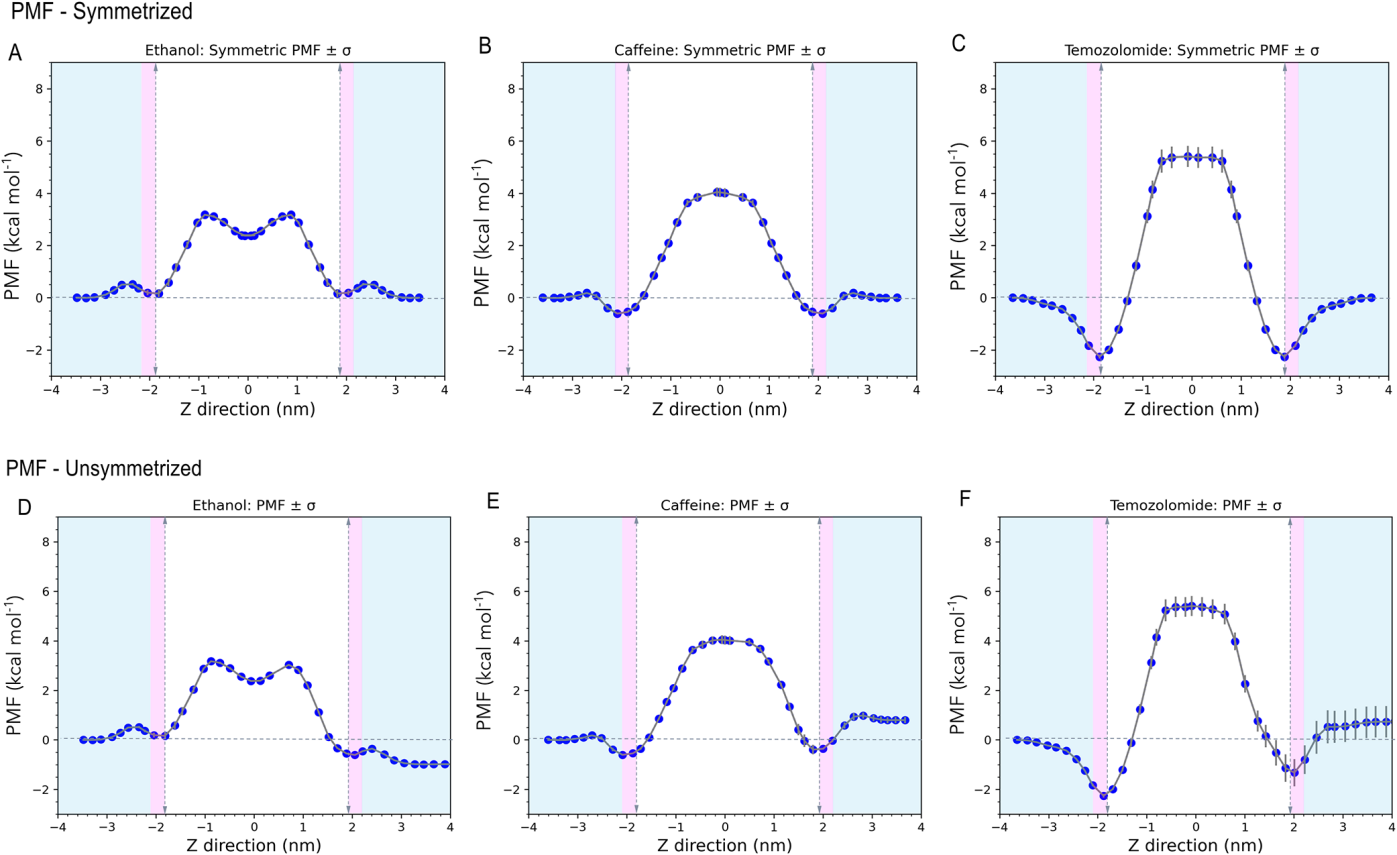
Individual potential of mean force (PMF) curves for ethanol, caffeine and temozolomide, respectively, from restrained simulations using simulation windows of 500 ns for the (A–C) symmetrized, and (D–F) unsymmetrized versions. The PMF is obtained by numerical integration of the average force, by ∫_–*z*_^*z*^⟨*F*(*z*)⟩d*z*, and symmetrization is applied at *z* = 0. The error bars are calculated as the standard deviation (σ) of the PMF at 3 time points: 400, 450, and 500 ns. The total simulation time was 19.5 μs for ethanol (39 windows), 19.5 μs for caffeine (39 windows) and 20 μs for temozolomide (40 windows). Color code: blue, water region; pink, polar headgroup region. White, inner core of membrane.

**Figure 3:**
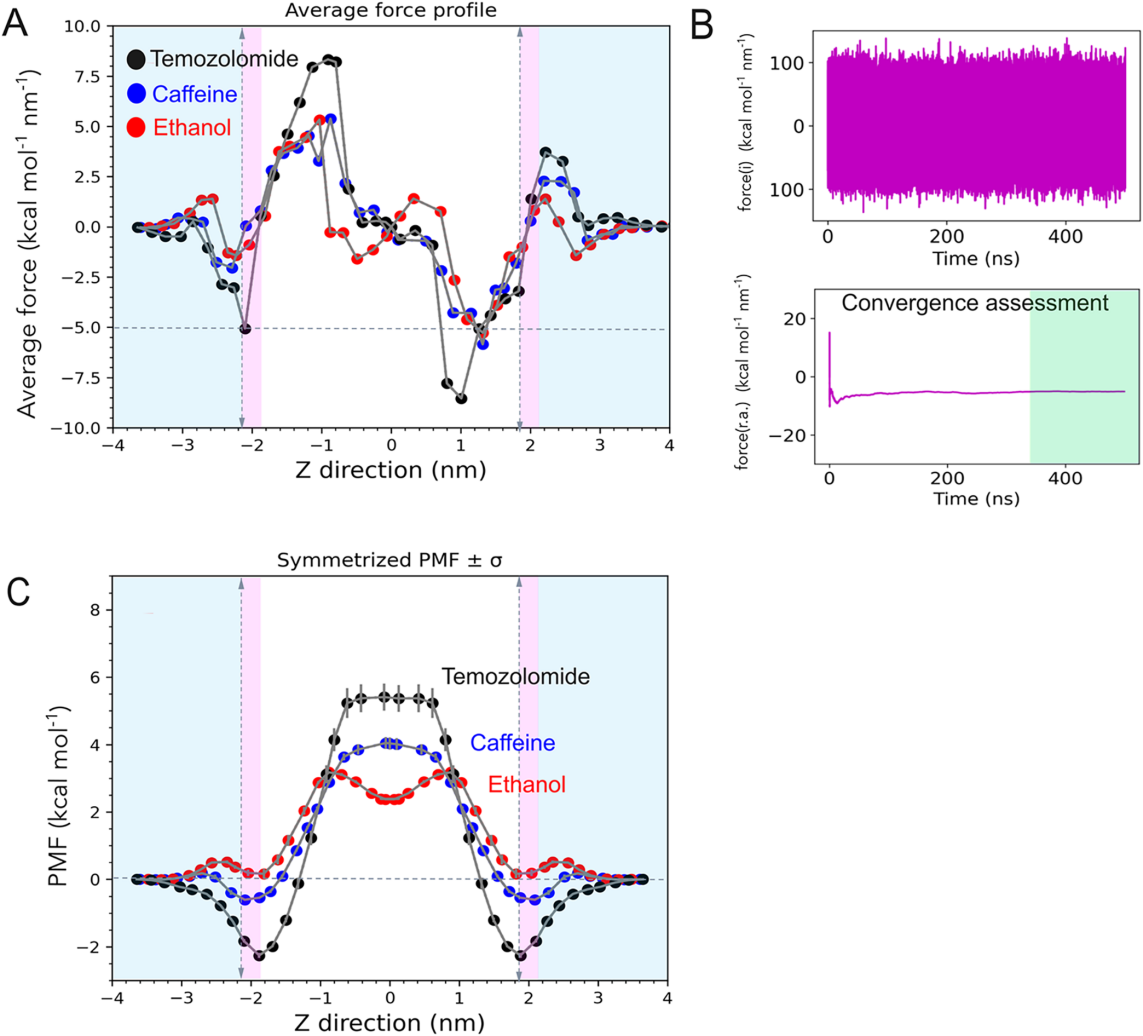
Comparison of the force profiles and potential of mean force (PMF) for ethanol, caffeine and temozolomide, respectively, from 500 ns biased sampling production runs of the force. (A) Average force profile (kcal mol^–1^ nm^–1^) for ethanol, caffeine and temozolomide, respectively, as a function of window along *z*, obtained by taking the average over the period 350 to 500 ns. (B) Instantaneous force profile (kcal mol^–1^ nm^–1^) reproduced directly from the GROMACS output (.xvg), and the subsequent running average force profile, which is the updated average value of the force in kcal mol^–1^ nm^–1^. (C) The PMF is obtained by numerical integration of the average force, as explained in eq 2: ∫_–*z*_^*z*^⟨*F*(*z*)⟩d*z*, using the cumtrapz function in scipy. The error bars are calculated as the standard deviation (σ) of the PMF at four time points: 350 ns, 400 ns, 450 and 500 ns, to aid in assessing the convergence over 150 ns and the associated late-stage relaxation in the instantaneous force.

### Diffusion Calculation

The unnormalized autocorrelation function (ACF) was computed using the acf (statsmodels.tsa.stattools.acf) routine from the *statsmodels* library^67^ implemented in Python.^68^ Analyses were performed in a Jupyter Notebook environment.^69^ The ACF was evaluated by considering a force value in the timeseries *f*(*i*) to a delayed version of itself. We analyzed the timeseries with either 10^3^, 1 × 10^4^ or 5 × 10^4^ lags, which amounts to analyzing each value *f*(*i*) to a delayed *f*(*i* – δ), i.e., delayed up to 5 × 10^4^ prior values in the force. For subsequent analysis, we settled on the long-lag regime of 5 × 10^4^ lags”. The force timeseries is printed every 50 MD timesteps, corresponding to a lag time of 100 fs. We used the default settings of acf, which ensures that the procedure automatically subtracts the sample mean from the series before computing the autocorrelations. The resulting autocorrelations are demonstrated for ethanol, caffeine and temozolomide in Figures S3–S5, and the comparison of resulting diffusivities (cm^2^ s^–1^) when using nlags set to a range between 10^3^ to 5 × 10^4^ is shown in Figure 5. The diffusivity eq 5 was calculated by estimating the integral of the ACF with the method of Fitzgerald et al.^47^ The fit was performed with curve_fit procedure of the scipy.optimize package with a maximum of 5,000 fitting iterations (maxfev).

### PMF Calculation

The PMF in Figure 2 was calculated by taking the average force at 500 ns and numerically integrating using the cumtrapz function in scipy. The error bar was calculated by taking the standard deviation between the PMF at 350, 400, 450, and 500 ns. The unsymmetrized form (Figure 2) was compared to the PMF obtained by gmx wham (Figure S16) and was found to be very similar, with comparable barrier heights and topologies.

## Results

### Thermodynamics of Translocation across a Blood–brain Barrier Endothelial Membrane

From 58 μs of production run simulation trajectories, we reconstruct the average force profile and then calculate the PMF by numerical integration. The resulting symmetrized PMFs are provided for ethanol (Figure 2A), caffeine (Figure 2B) and temozolomide (Figure 2C), as well as the unsymmetrized for ethanol (Figure 2D), caffeine (Figure 2E) and temozolomide (Figure 2F).

In Figure 3A we depict the average force profile for ethanol, caffeine and temozolomide, a demonstration of the running force profile (Figure 3B) for convergence assessment of the force profile, as well as the final PMF (Figure 3C). In Figure S1 we show the systematic assessment of plateau in the running force, which is attained in all the windows force profiles after ∼200 ns. This convergence analysis is complemented by assessing the convergence of the PMF with added sampling in Figure S2. From this analysis, it can be discerned that the ethanol and caffeine PMF converge after ∼400 ns of sampling per window, after which they no longer change with added sampling. For temozolomide, the full 500 ns per window are required to conclude convergence in the PMF (Figure S2).

We estimate the barrier height Δ*G* ^‡^ (Table 2) from the PMF in Figure 2, observing the progressive increase in barrier heights from ethanol (3.18 kcal mol^–1^) to temozolomide (7.63 kcal mol^–1^), and this is reflected in a progressively slower experimental permeability from ethanol (1.10 × 10^–3^ cm s^–1^) to temozolomide (1.86 × 10^–6^ cm s^–1^). In general, the topology of the PMF turns from a classical hydrophilic compound PMF with a single-barrier behavior^9,10^ to exhibiting a true local minimum at the polar headgroup region, followed by a large hydrophilic for temozolomide.

**Table 2:** Simulation Time (μs), Barrier Height Δ*G* ^**‡**^ (kcal mol^‑1^) from PMF, As Well As Experimental Permeabilities for Ethanol, Caffeine, and Temozolomide, Respectively

Molecule	Simulation time (μs)	Δ*G* ^‡^ (kcal mol^–1^)	*P*_app_ (cm s^–1^)
Ethanol	19.5	3.18	1.10 × 10^–3^
Caffeine	19.5	4.64	2.10 × 10^–5^
Temozolomide	20	7.63	1.86 × 10^–6^

Previous studies by Chipot and colleagues^17,25^ performed PMF benchmarks with a variety of techniques, including between ABF and umbrella sampling (US) or replica-exchange MD (REMD). In similar fashion, we show a control simulation in Figure 4 of the PMF for ethanol from biased sampling when compared to the PMF obtained from unbiased MD simulations at 310 K using a number *N* of ethanol molecules populated randomly in the water box.^9^ Through sufficient sampling, spontaneous transitions of ethanol are observed, and a converged probability distribution function can be inferred.^9^ The PMF itself is broader in *z* than the classical ethanol PMF in POPC by Gullingsud and Schulten.^70^ This arises because a POPC bilayer has a thickness of ∼3.7 nm^70^ to ∼3.8 nm^71,72^ while simulated mammalian membrane models have been found to have a bilayer thickness of ∼4.7 nm,^72^ in line with the calculated bilayer thickness of the hBMEC BBB membrane used here, which was estimated to be ∼4.75 nm.^9^

**Figure 4:**
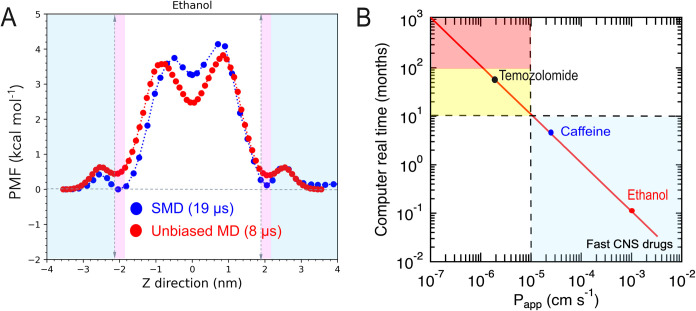
Comparison of biased cv-SMD-US simulations to unbiased MD shows that both methods capture the overall topology of ethanol permeation. (A) PMF comparison between one sampled from unbiased MD in red to our PMF from numerical integration of the average force profile from biased cv-SMD-US simulations (blue) using the cumtrapz function in scipy. The data set in red is reproduced from our previous work^9^ at 310 K under a Creative Commons Attribution 4.0 International License, 2019 Springer Nature Limited. (B) This comparison is only possible for compounds with a permeability faster than >10^–4^ cm s^–1^, due to the sampling limitations from unbiased MD. Distribution of experimental permeability *P*_app_ (cm s^–1^) relative to the back-calculated value of computational time (months) for a single GPU, reproduced from our previous work.[Bibr ref10] 2021 American Chemical Society. This analysis motivated the need for biased sampling techniques. While conventional MD might be sufficient for ethanol, already at caffeine it is computationally impractical to use conventional MD due to the timescales needed, while for temozolomide it is computationally prohibitive to run unbiased MD.

### Inferring Kinetics of Translocation across a Blood-Brain Barrier Membrane from the Green–Kubo Equations

We now proceed to use the ISD framework (eq 1) to calculate the transmembrane permeability of each molecule by inputting the converged PMF of the molecule (Figure 3) together with the diffusivity *D_z_* obtained from the Green–Kubo equation (eq 5). The autocorrelation function (ACF) was computed with the unnormalized acf­(statsmodels.tsa.stattools.acf) routine from the *statsmodels* library^67^ implemented in Python.^68^ Since the acf routine returns the normalized ACF starting from a default value of 1, it was then unnormalized subsequently back to a range reflecting the magnitude of the pulling force. Analyses were performed in a Jupyter Notebook environment.^69^ The ACF was evaluated on a sample of 500,000 force values (350 to 400 ns), by considering a force value in the timeseries *f*(*i*) to a delayed version of itself. We analyzed the timeseries with 5 × 10^4^ lags, which amounts to analyzing each value *f*(*i*) to a delayed *f*(*i* – δ) up to 5 × 10^4^ prior values in the force, i.e., ACF ρ(0), ρ(1), ρ(2), ..., ρ­(50,000). The force timeseries is printed every 50 MD timesteps, corresponding to a lag time of 100 fs. With this protocol we can evaluate the long-time decorrelation in adjacent force samples. We consider up to 5 × 10^4^ lags to include the long-lag correlation behavior. A justification of these choices is provided in the Supporting Information, specifically Figures S8–S15.

The procedure is demonstrated for example windows in Figure 5, showcasing the autocorrelation procedure done on the converged portion of the force timeseries, 350 to 500 ns. The windows are labeled *N* = 1, ..., 39 for ethanol and caffeine, and *N* = 1, ..., 40 for temozolomide. The first windows (*N* = 1–5) corresponds to the *z*-constrained lateral diffusion of the drug in the water box, then the subsequent windows (6–12) consist of the drug permeating the polar headgroup region of the lipids, followed by windows 13–20 in which the drug translocates into the center of the membrane, which is the region of least polarity. The subsequent windows (21–39) span the extrusion of the drug starting at the center of the membrane all the way back to the water region. The resulting autocorrelations are demonstrated for ethanol, caffeine and temozolomide in Figure S3–S5. The ACF was integrated with the integrate.cumtrapz protocol.

**Figure 5:**
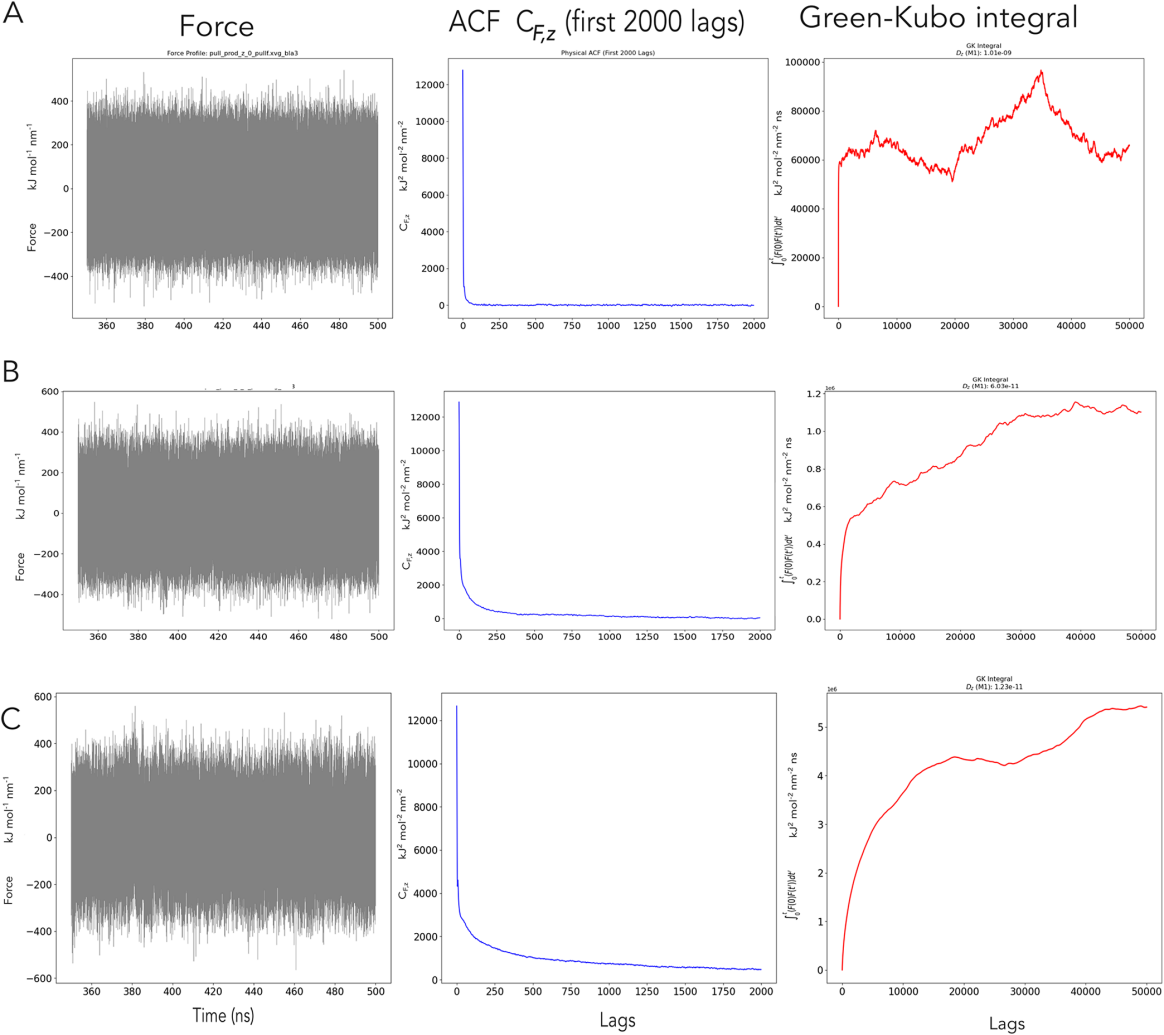
Autocorrelation and integral behavior demonstrated for caffeine transport windows 0, 5, and 10. The results presented are for analyses done on the raw force files generated for each of the select windows in the converged regime of the force, i.e., between 350 and 500 ns. Each calculation of the autocorrelation function uses the *N* = 5 × 10^4^ lags that was demonstrated in Figures S7–S15 to be optimal. The workflow is as follows: (A) the converged portion of the raw force (kJ mol^–1^ nm^–1^) between 350 and 500 ns is input; (B) the unnormalized autocorrelation function, *C*_*F,z*_, is calculated and shown for the first 2000 lags; (C) the integral of the ACF *C*_*F,z*_ is taken with cumtrapz in scipy, shown for 50,000 lags.

As seen in Figures 5 and S3–S5, the integral does not plateau in certain cases.

To avoid the ambiguities from estimating the plateau of the numerically divergent integral of the ACF, we resort to using the method of Fitzgerald et al. (eq 6 and 7) for all estimations of the integral of the ACF, and as such, for subsequent diffusivity calculations. We parametrized a set of initial fitting parameters (*a, b, c*) of eq 6. Upon investigating, we opted for initial fitting parameters (var.(*F*_*z*_), 4.0, 2.0), where var.(*F*) is the variance in the force profile *i*, which yielded converged fits with the curve_fit procedure of the scipy.optimize package with a maximum of 5000 fitting iterations (maxfev). The resulting diffusivities (cm^2^ s^–1^) are depicted in Figure 6, analysis with 1 × 10^4^, 5 × 10^4^, and 1 × 10^5^ lags. Due to the highly correlated of the nature of the force in the windows where the drug is constrained in *z* in the membrane, we used up to 5 × 10^4^ lags when doing the calculations. We point the reader to the validation in Figures S8–S15, which confirms that 5 × 10^4^ lags is sufficient to yield agreement across all molecules. This value was used to compute *D_z_* with eq 5, with error bars σ_*D*z_ reported as the standard deviation between the plateau value at lag 30,000, 35,000, 40,000, 45,000, and 50,000. We corrected few pathological plateaus with the procedure of Fitzgerald et al.,^47^ fitting the early 2000 lags of the ACF onto a general exponential of the form eq 6.

**Figure 6:**
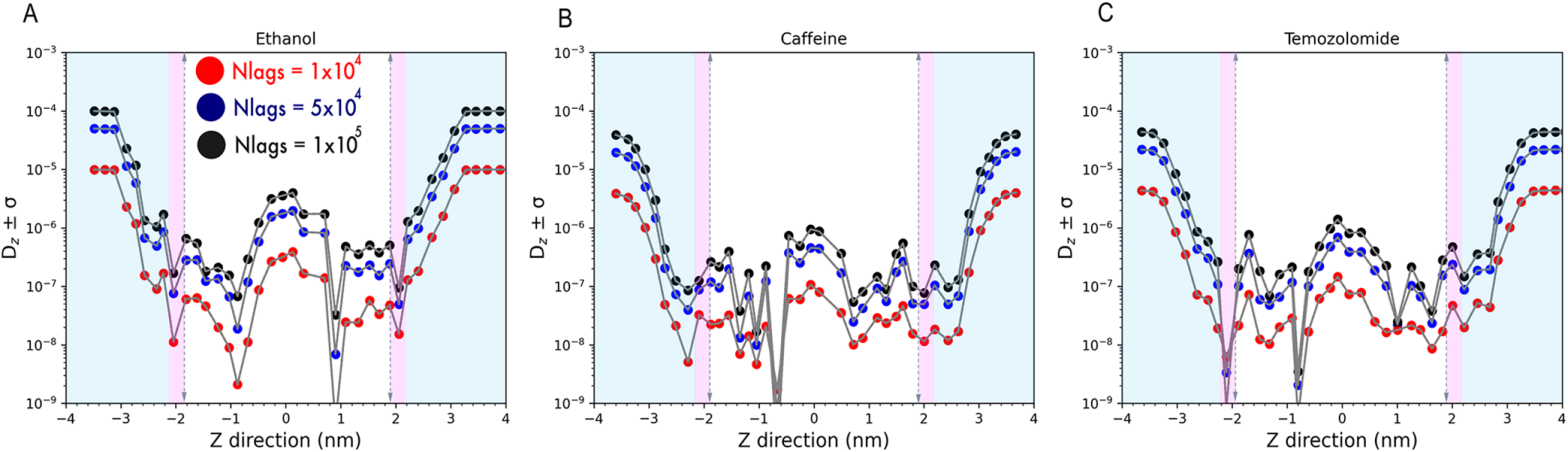
Position-dependent diffusivity for (A) ethanol, (B) caffeine, and (C) temozolomide diffusion across the BBB from eq 5. To calculate the diffusivity, the procedure of Fitzgerald et al.^47^ was used, fitting the early 2,000 lags of the ACF onto a general exponential of the form eq 6. This was then reweighted by (*RT*)^2^ to yield *D_z_*. Results from three number of lags in the autocorrelation calculation (nlags) were considered: 10^4^, 5 × 10^4^, and 10^5^ lags show that the diffusivity reaches consistency at 5 × 10^4^ lags, in line with the validation plots (Figures S7–S15). All analysis was performed for the window 350 to 500 ns timeseries.

The diffusivities in Figure 6 were input into eq 1, together with the PMF of Figure 2. The resulting transmembrane drug permeabilities (cm s^–1^) are displayed in Table 3 for ethanol, caffeine and temozolomide. The procedure employed here cv-SMD-US/ISD procedure can estimate the experimental permeability to ∼1 order magnitude agreement, with the error to experiment ranging from 6 to 29. In Figure 7 we consider the scatterplot of *P*_sim_ to *P*_app_, as well relationship between experimental permeability *P*_app_ to the barrier height, which increases from ethanol (3.18 kcal mol^–1^) to temozolomide (7.63 kcal mol^–1^), and this is reflected in a progressively slower experimental permeability from ethanol (1.10 × 10^–3^ cm s^–1^) to temozolomide (1.86 × 10^–6^ cm s^–1^).

**Figure 7:**
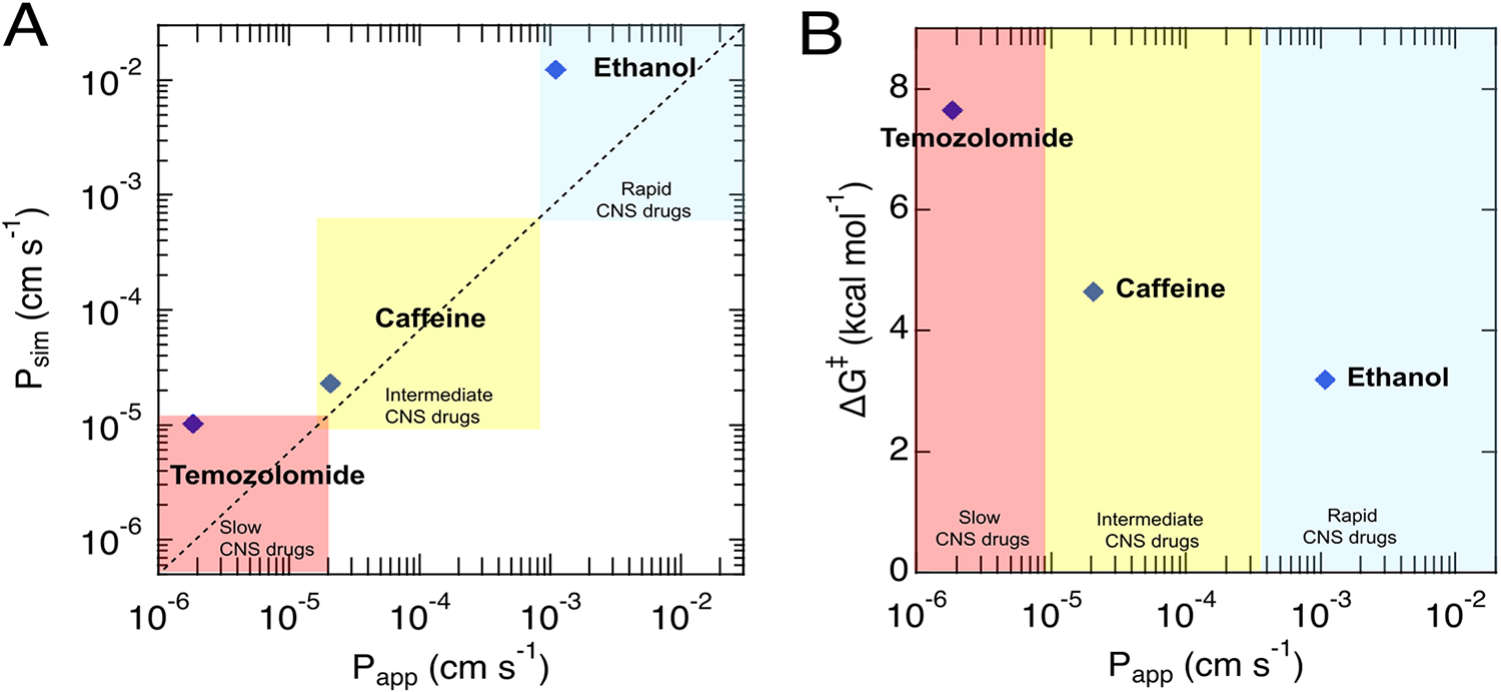
(A) Permeability scatter between experimental *P*_app_ and simulated *P*_sim_ values. Regions denoting slow, intermediate and rapid permeation span the following regions: slow (1 × 10^–6^ to 1 × 10^–5^ cm s^–1^; red), intermediate (1 × 10^–5^ to 1 × 10^–3^ cm s^–1^; yellow) and fast (1 × 10^–3^ to 3 × 10^–1^ cm s^–1^; cyan). (B) Barrier height Δ*G* ^‡^ (kcal mol^–1^) for permeant across the BBB membrane, plotted along the associated experimental permeability *P*_app_ (cm s^–1^).

**Table 3:** Resulting Simulated Permeability *P*_sim_ (cm s^–1^) Using the cv-SMD-US/ISD Approach (eq 1) Together with the Green–Kubo Equation and Asymmetric PMF from Numerical Integration; Experimental Permeability *P*_app_ (cm s^–1^); Ratio of Simulated to Experimental Permeability, *P*_sim_/*P*_app_; Original Source and Reference for Experimental Data

Molecular	*P*_sim_ (cm s^–1^)	*P*_app_ (cm s^–1^)	*P*_sim_/*P*_app_	Cell line or source of *P*_app_ (cm s^–1^)	Source
Ethanol	1.22 × 10^–2^	1.10 × 10^–3^	11.1	Red blood cell	^48^
Caffeine	2.25 × 10^–5^	2.10 × 10^–5^	1.1	MDCK	^9^
Temozolomide	1.01 × 10^–5^	1.86 × 10^–6^	5.4	3D brain perfusion	^51^

## Discussion

In this work, we predict the permeability of three small-molecule compounds that are known to cross the blood-brain barrier, through a model of the human brain endothelium. The justification and full lipid composition of the apical component of the BBB endothelium is given in Table 4. The method, cv-SMD-US/ISD, has been employed on three compounds with varying permeabilities, and was found to have ∼1 order magnitude precision to experiment, and can adequately assign and distinguish between permeabilities on the order of 10^–3^ cm s^–1^ (fast) to the slowest permeabilities known, below 10^–6^ cm s^–1^. This broad testing of permeability values is important for any methodology, and we caution against solely benchmarking against a single part of the permeability space, such as those methods tested in the fast regime.^33^

**Table 4:** Composition of BBB Apical Endothelial Membrane^a^

Lipid	Number	% composition	Justification
Cholesterol	28	29.1	Cholesterol component
OSM	18	18.8	Sphingomyelin component
SAPE	14	14.5	Phospholipid component
SAPC	8	8.3	Phospholipid component
SAPS	8	8.3	Phospholipid component
SLPC	8	8.3	Phospholipid component
SOPE	6	6.3	Phospholipid component
SOPE	6	6.3	Phospholipid component
POPC	4	4.1	Phospholipid component
SAPI	2	2.0	Phospholipid component
Total	96	100	-

a The general principles of the endothelial membranes are laid out in previous work.^9^ Some governing principles include the need for ∼30% cholesterol, sphingomyelin lipids at ∼20% and remainder of phospholipids. *N*-oleoyl-sphingomyelin (OSM; 18), 1-palmitoyl-2-oleoyl-glycero-3-phosphocholine (POPC; 4), 1-stearoyl-2-arachidonoyl-snglycero-3-phosphocholine (SAPC; 8), 1-stearoyl-2 arachidonoyl-sn-glycero-3-phosphoethanolamine (SAPE; 14), 1-stearoyl-2-oleoyl-sn-glycero-3-phosphoethanolamine (SOPE; 6), 1-stearoyl-2-arachidonoyl-sn-glycero-3-phospho-l-serine (SAPS; 8), 1-stearoyl-2-linoleoyl-sn-glycero-3-phosphocholine (SLPC; 8), 1-stearoyl-2-arachidonoyl-sn-glycero-3-phosphoinositol (SAPI; 2) and cholesterol (28).

The shape and magnitude of the diffusivity curves in Figure 6 obtained with the Green–Kubo eq 5 follows those reported elsewhere^4,17,25^ using ABF, REUS and US techniques for small-molecule permeation through homogeneous bilayers such as DMPC. The rapid diffusion in the water phase ranges between 8.14 × 10^–5^ to 2.10 × 10^–4^ cm^2^ s^–1^ for ethanol and caffeine, respectively (Figure 6). These values are like those reported by Chipot for transport of codeine, benzoic acid or urea estimated by either US or REUS, in which the diffusivity in the aqueous windows is in the ∼10^–5^ cm^2^ s^–1^ range. When the permeant enters the bilayer phase, the diffusivity drops to values in the ∼2.6 × 10^–7^ to 3 × 10^–8^ cm^2^ s^–1^ range.

We further explored the role of the asymmetry in the underlying PMF, and its effect on the permeability. In Figure 2, an initial comparison is made between symmetric and asymmetric PMFs. In Table 5 we present a systematic comparison between the effect of a symmetrized versus unsymmetrized PMF on the ISD permeability. As we see, the simulated permeability using the ISD equation eq 1 is consistently at most ∼1 order of magnitude from the experimental permeability, but it does appear that the symmetrization procedure brings the agreement slightly closer.

**Table 5:** Resulting Simulated Permeability *P*_sim_ (cm s^–1^) Using the cv-SMD-US/ISD Approach eq 1 Together with the Green–Kubo Equation and Either Asymmetric PMF or a Symmetrized PMF as Depicted in Figure 2 Resulting in *P*_sim,unsym_ and *P*_sim,sym_; Experimental Permeability *P*_app_ (cm s^–1^); Ratio of Symmetrized Simulated Permeability to Experimental Permeability, *P*_sim,sym_/*P*_app_; Ratio of Unsymmetrized Simulated Permeability to Experimental Permeability, *P*_sim,unsym_/*P*_app_; Original Ssource and Reference for Experimental Data

Molecular	*P*_sim_ (cm s^–1^) with Δ*G*_unsym_	*P*_sim_ (cm s^–1^) with Δ*G*_sym_	*P*_app_ (cm s^–1^)	*P*_sim,unsym_/*P*_app_	*P*_sim,sym_/*P*_app_
Ethanol	1.22 × 10^–2^	7.21 × 10^–3^	1.10 × 10^–3^	11.1	6.6
Caffeine	2.25 × 10^–5^	3.23 × 10^–5^	2.10 × 10^–5^	1.1	1.5
Temozolomide	1.01 × 10^–5^	8.40 × 10^–6^	1.86 × 10^–6^	5.4	4.5

Our findings in Table 5 show that it is possible to rank the compounds in the correct order, from the very slowest (temozolomide) to the very fastest (ethanol). There is an offset to experiment, and this offset is well documented elsewhere, with reported permeability error ranges from ABF having been found to be ∼1 order of magnitude faster,^4,17,25^ while cases with US have reported the simulated permeability to be 1 order of magnitude slower for e.g., urea permeation.^25^

We find there to be a correlation between the experimental permeability and the barrier height in the PMF sampled from numerical integration of the average force required to restrain the drug along *z* (Figure 7B). While kinetics and thermodynamics are fundamentally uncoupled, this point follows the remark of Chipot and colleagues^25^ that the ISD permeability is generally more sensitive to the value of Δ*G* than to the value *D_z_*.

There are limitations to the cv-SMD-US/ISD methodology. First, the convergence of the approach can require upward of 500 ns per window for the very slow drugs (temozolomide). However, it should be said about this point that we are not aware of any prior simulation work that considered drugs with experimental permeabilities below 10^–6^ cm s^–1^, as these very slow molecules are mostly confined to brain crossing, due to the high rejection rate of the blood-brain barrier compared to other barriers such as epithelial. As such, benchmarking the cv-SMD-US/ISD method on such slow molecules is an important test that ought to be generalized for other enhanced sampling permeability protocols. Recent work by Harris et al.^44^ shows that the ISD framework can break down when it ignores Markovian effects along the reaction coordinate. When a solute moves through the membrane, slow reorganization of the environment occurs. Instead of responding simultaneously, the environment exerts a delayed frictional drag force on the solute, described by the Generalized Langevin Equation (GLE). A major limitation of the SMD/ISD approach is the lack of explicit incorporation of Markovian effects. This limitation is particular critical for temozolomide, a polar molecule. Markovian effects are more accentuated for polar molecules compared to more hydrophobic molecules. A third limiting factor of using cv-SMD-US/ISD is the reliance on autocorrelation functions to measure the decorrelation of the force in each window.^47^ However, as pointed out by Lee et al.,^25^ from systematic benchmarking of the components of the ISD on the final permeability value, the PMF is the dominant component in the ISD equation, with diffusivity playing only a minor effect in the overall permeability. Furthermore, the assumption of simulating all compounds in the neutral form is a limitation that ought to be addressed in the future, e.g., via the use of constant-pH MD simulations^73^ or the likes. All in all, this approach merits further exploration of a broader range of permeants with different types of biological membranes. While the use of the endothelial membrane is a good start, there are other important biological barriers that should be explored.

## Conclusion

Transport of molecules across the blood-brain barrier is a major limiting factor in developing the next generation of CNS therapeutics for brain cancer, Alzheimer’s and Parkinson’s disease. Computational modeling of transport across the CNS can significantly improve our understanding of the underlying biophysics of the endothelial CNS, including transport properties and thermodynamics. The use of permeability remains the gold standard metric, but it is a challenging property to estimate correctly *in vitro* or *in vivo*.

In this work, we used computational modeling to estimate the permeability of three CNS active compounds–ethanol, caffeine and temozolomide–through a model of the human brain endothelium using multiμs steered molecular dynamics simulations with the inhomogeneous-solubility diffusion equation. The method, cv-SMD-US/ISD, was employed on three compounds of distinct experimental permeabilities, and was found to predict the permeability to within order-of-magnitude accuracy. The procedure was tested on fast permeabilities of the order of 10^–3^ cm s^–1^ to the slowest permeabilities known (<10^–6^ cm s^–1^). Further use of this method is warranted to map out additional compounds, thereby helping to expand our understanding of CNS therapeutic delivery.

## Supplementary Material







## Data Availability

A Zenodo repository is included for the production trajectories (.xtc) of ethanol, caffeine, and temozolomide at https://zenodo.org/records/20920485, including .tpr, index, topology and initial .pdb files.

## Notes

Ethanol caffeine temozolomide contains the starting snapshot, index file, associated parameters and topology for ethanol, caffeine, and temozolomide (ZIP)
Extended methods: the unit analysis of the inhomogeneous solubility-diffusion (ISD) framework; the unit analysis of the Green–Kubo relation; approximating the limit of the Green Kubo integral by a Γ function; the Green–Kubo relations and the Fluctuation–Dissipation Theorem for mobility; the Green–Kubo relations and the Fluctuation–Dissipation Theorem for force; convergence assessment of the unsymmetrized potential of mean force (PMF) for ethanol, caffeine and temozolomide (Figure S1); autocorrelation analysis for ethanol, caffeine and temozolomide (Figure S2); autocorrelation analysis for ethanol (Figure S3); autocorrelation analysis for caffeine (Figure S4); autocorrelation analysis for temozolomide (Figure S5); full trace for the integral of the autocorrelation for ethanol, caffeine and temozolomide analyzed for interval 350 to 500 ns using *N* = 5 × 10^4^ lags. (Figure S7); systematic evaluation of the autocorrelation function of a single force profile for caffeine (Figures S8–S13); systematic evaluation of the autocorrelation function of a single force profile for ethanol (Figure S14); systematic evaluation of the autocorrelation function of a single force profile for temozolomide (Figure S15); individual potential of mean force (PMF) curves for ethanol, caffeine and temozolomide, using gmx wham and compared to numerical integration (Figure S16); CHARMM General Force Field (CGenFF) charge models for temozolomide, caffeine and ethanol (Table S1); Green–Kubo components for ethanol per window (Table S2); Green–Kubo components for caffeine per window (Table S3); Green–Kubo components for temozolomide per window (Table S4); Γ function fitting coefficients (*a*, *b*, *c*) components obtained by solving the fit of the ACF onto the general exponential in eq S5 for ethanol per window (Table S5); Γ function fitting coefficients (*a*, *b*, *c*) components obtained by solving the fit of the ACF onto the general exponential in eq S5 for caffeine per window (Table S6); Γ function fitting coefficients (*a*, *b*, *c*) components obtained by solving the fit of the ACF onto the general exponential in eq S5 for temozolomide per window (Table S7) (PDF)
Support data Jorgensen JCIM contains the raw data for calculating the permeabilities in Table 3, including the PMF in Figure 2 and diffusivities in Tables S2–S4 (XLSX)

## Abbreviations

Cv-SMD: constant-velocity Steered Molecular Dynamics
CNS: central nervous systems
BBB: blood-brain barrier
hBMEC: human brain microvascular endothelial cell
ISD: inhomogeneous solubility-diffusion
ABF: adaptive biasing force
US: umbrella sampling
REMD: replica exchange Molecular Dynamics
2D: two-dimensional
3D: three-dimensional
PMF: Potential of mean force
MDCK: Madin Darby kidney cell
