# Supplementary material for: Estimating Permeabilities from Biased Molecular Dynamics with a Green–Kubo Formalism for Transport across the Blood-Brain Barrier
Source: J Chem Inf Model. 2026 Jul 21;66(15):9495–506. doi: 10.1021/acs.jcim.6c01624 (PMC13471738; doi:10.1021/acs.jcim.6c01624)
Supplement: Supplementary file 1 [file ci6c01624_si_002.pdf]

Estimating permeabilities from biased molecular dynamics with a Green-Kubo formalism for transport across the blood-brain barrier.

Christian Jorgensen[1*]

[1]. **School of Medicine, Pharmacy and Biomedical Sciences, Faculty of Science & Health, University of Portsmouth, Portsmouth, PO1 2DT, Hampshire, UK**

[*] **Correspondence to Christian.Jorgensen@port.ac.uk**

Table of Contents

1. **Extended Methods**

1.1 Unit analysis of the Inhomogeneous Solubility-Diffusion (ISD) framework (Equation S1)

1.2 Unit analysis of the Green-Kubo relation (Equation S2)

1.3 Approximating the limit of the Green Kubo integral by a Gamma function (Equation S6)

1.4 The Green-Kubo relations and the Fluctuation-Dissipation Theorem for mobility

1.5 The Green-Kubo relations and the Fluctuation-Dissipation Theorem for force

2. **List of Figures**

Figure S1. **Convergence assessment of the unsymmetrized potential of mean force (PMF) for (A) ethanol, (B) caffeine and (C) temozolomide, using window simulation lengths of 350, 400, 450 and 500 ns *per window*.** The PMF are obtained by numerical integration of the average force plateau $\int_{-z}^{z}\langle F(z)\rangle dz$. The total simulation time for ethanol and caffeine was 13.7 µs (350 ns), 15.6 µs (400 ns), 17.6 µs (450 ns) and 19.5 µs (500 ns). The total simulation time for temozolomide was 14 µs (350 ns), 16 µs (400 ns), 18 µs (450 ns) and 20 µs (500 ns).

Figure S2. **Autocorrelation analysis for (A-C) ethanol, caffeine and temozolomide, respectively.** The results presented consider two lag time analyses: (upper) $N = 10^3$ lags and (lower) 10^4 lags, respectively. The analysis takes in the raw force (kJ mol^{-1} nm^{-1}), calculates the autocorrelation function.

Figure S3. **Autocorrelation analysis for ethanol.** (A) The results presented are for lag time of N = 5 x 10^4 lags. The analysis takes in the (A) raw force (kJ mol^{-1} nm^{-1}), calculates the autocorrelation function in (B) and subsequent integral of the ACF (C).

Figure S4. **Autocorrelation analysis for caffeine.** (A) The results presented are for lag time of N = 5 x 10^4 lags. The analysis takes in the (A) raw force (kJ mol^{-1} nm^{-1}), calculates the autocorrelation function in (B) and subsequent integral of the ACF (C).

Figure S5. **Autocorrelation analysis for temozolomide.** (A) The results presented are for lag time of N = 5 x 10^4 lags. The analysis takes in the (A) raw force (kJ mol^{-1} nm^{-1}), calculates the autocorrelation function in (B) and subsequent integral of the ACF (C).

Figure S6. **Full trace for the integral of the autocorrelation for (A) ethanol, (B) caffeine and (C) temozolomide analyzed for interval 350 ns to 500 ns using N = 5 x 10^4 lags**. The analysis takes in the raw force (kJ mol^{-1} nm^{-1}), calculates the autocorrelation function in, and subsequently computes the integral of the ACF.

Figure S7. Systematic evaluation of the autocorrelation function of a single force profile pull_prod_z_5_pullf.xvg varying (a) the force profile interval, (b) the lag number. The force segments considered were those segments used for convergence analysis, (i) 350 – 400 ns, (ii) 350 – 450 ns, and (iii) 350 – 500 ns.

Figure S8. Systematic evaluation of the autocorrelation function of a single force profile pull_prod_z_10_pullf.xvg varying (a) the force profile interval, (b) the lag number. The force segments considered were those segments used for convergence analysis, (i) 350 – 400 ns, (ii) 350 – 450 ns, and (iii) 350 – 500 ns.

Figure S9. Systematic evaluation of the autocorrelation function of a single force profile pull_prod_z_15_pullf.xvg varying (a) the force profile interval, (b) the lag number. The force segments considered were those segments used for convergence analysis, (i) 350 – 400 ns, (ii) 350 – 450 ns, and (iii) 350 – 500 ns.

Figure S10. Systematic evaluation of the autocorrelation function of a single force profile pull_prod_z_20_pullf.xvg varying (a) the force profile interval, (b) the lag number. The force segments considered were those segments used for convergence analysis, (i) 350 – 400 ns, (ii) 350 – 450 ns, and (iii) 350 – 500 ns.

Figure S11. Systematic evaluation of the autocorrelation function of a single force profile pull_prod_z_25_pullf.xvg varying (a) the force profile interval, (b) the lag number. The force segments considered were those segments used for convergence analysis, (i) 350 – 400 ns, (ii) 350 – 450 ns, and (iii) 350 – 500 ns.

Figure S12. Systematic evaluation of the autocorrelation function of a single force profile pull_prod_z_30_pullf.xvg varying (a) the force profile interval, (b) the lag number. The force segments considered were those segments used for convergence analysis, (i) 350 – 400 ns, (ii) 350 – 450 ns, and (iii) 350 – 500 ns.

Figure S13. Systematic evaluation of the autocorrelation function of a single force profile pull_prod_z_35_pullf.xvg varying (a) the force profile interval, (b) the lag

number. The force segments considered were those segments used for convergence analysis, (i) 350 – 400 ns, (ii) 350 – 450 ns, and (iii) 350 – 500 ns.

Figure S14. Systematic evaluation of the autocorrelation function of a single force profile pull_prod_z_05_pullf.xvg of ethanol varying (a) the force profile interval, (b) the lag number. The force segments considered were those segments used for convergence analysis, (i) 350 – 400 ns, (ii) 350 – 450 ns, and (iii) 350 – 500 ns.

Figure S15. Systematic evaluation of the autocorrelation function of a single force profile pull_prod_z_05_pullf.xvg of temozolomide varying (a) the force profile interval, (b) the lag number. The force segments considered were those segments used for convergence analysis, (i) 350 – 400 ns, (ii) 350 – 450 ns, and (iii) 350 – 500 ns.

Figure S16. **Individual potential of mean force (PMF) curves for ethanol, caffeine and temozolomide, respectively, using (A)-(C) gmx wham on simulation windows of 500 ns, and compared to PMFs obtained from numerical integration of the average force profile. We depict the (D)-(F) symmetrized, and (G)-(I) unsymmetrized versions.** Numerical integration of the average force, by $\int_{-z}^{z}\langle F(z)\rangle dz$, and symmetrization is applied at $z = 0$. The error bars are calculated as the standard deviation (σ) of the PMF at 3 timepoints: 400 ns, 450 ns and 500 ns. The total simulation time was 19.5 μs for ethanol (39 windows), 19.5 μs for caffeine (39 windows) and 20 μs for temozolomide (40 windows).

3. List of Tables

Table S1. CHARMM General Force Field (CGenFF) charge models for the drugs being considered: temozolomide, caffeine and ethanol.

Table S2. Green-Kubo components (Equation S1) for ethanol per window: (1) average *z* value of window, (2) *D*(z) (cm^2s^{-1}) from Green-Kubo relation using the converged part of the force (350 to 500 ns) and 5 x 10^4 lags. *D*(z) is calculated by analytical solution of the fit to a general exponential function (Fitzgerald et al., 2023), *G*(z) (kJ mol^{-1}) from integration of force profile, ISD equation S1 component $\exp(\Delta G(z)/RT)/D(z)$ ($cm^{-2}s$), ISD equation S1 component $\Delta z * \exp(\Delta G(z)/RT)/D(z)$ ($cm^{-1}s$), Δz (cm).

Table S3. Green-Kubo components (Equation S1) for caffeine per window: (1) average *z* value of window, (2) *D*(z) (cm^2s^{-1}) from Green-Kubo relation using the converged part of the force (350 to 500 ns) and 5 x 10^4 lags. *D*(z) is calculated by solution of the fit to a general exponential function (Fitzgerald et al., 2023), *G*(z) (kJ mol^{-1}) from integration of force profile, ISD equation S1 component $\exp(\Delta G(z)/RT)/D(z)$ ($cm^{-2}s$), ISD equation S1 component $\Delta z * \exp(\Delta G(z)/RT)/D(z)$ ($cm^{-1}s$), Δz (cm).

Table S4. Green-Kubo components (Equation S1) for temozolomide per window: (1) average *z* value of window, (2) *D*(z) (cm^2s^{-1}) from Green-Kubo relation using the converged part of the force (350 to 500 ns) and 5 x 10^4 lags. *D*(z) is calculated by solution of the fit to a general exponential function (Fitzgerald et al., 2023). *G*(z) (kJ mol^{-1}) from integration of force profile, ISD equation S1 component $\exp(\Delta G(z)/RT)/D(z)$ ($cm^{-2}s$), ISD equation S1 component $\Delta z * \exp(\Delta G(z)/RT)/D(z)$ ($cm^{-1}s$), Δz (cm).

Table S5. Gamma function fitting coefficients (*a, b, c*) components obtained by solving the fit of the ACF onto the general exponential in Equation S5 for ethanol per window: (1) average *z*

value of window, fitting coefficients (*a, b, c*), and resulting diffusivity *D*(z) (cm^{2}s^{-1}) obtained from the Green-Kubo equation.

Table S6. Gamma function fitting coefficients (*a, b, c*) components obtained by solving the fit of the ACF onto the general exponential in Equation S5 for caffeine per window: (1) average *z* value of window, fitting coefficients (*a, b, c*), and resulting diffusivity *D*(z) (cm^{2}s^{-1}) obtained from the Green-Kubo equation.

Table S7. Gamma function fitting coefficients (*a, b, c*) components obtained by solving the fit of the ACF onto the general exponential in Equation S5 for temozolomide per window: (1) average *z* value of window, fitting coefficients (*a, b, c*), and resulting diffusivity *D*(z) (cm^{2}s^{-1}) obtained from the Green-Kubo equation.

1. Extended Methods

1.1 Unit analysis of the Inhomogeneous Solubility-Diffusion (ISD) framework (Equation S1)

The ISD framework (Equation 1) is one of the most common approaches to calculate the transcellular permeability P_{sim} (cm s^{-1}) from biased simulations. The inputs are the position-dependent diffusion *D*(*z*) (cm^2 s^{-1}) and free energy Δ*G*(*z*) (kJ mol^{-1}) along the transition coordinate *z* (nm), and the permeability directly follows from solving the Smoluchowski equation under stationary conditions, as described in Equation S1. Here by use of either Riemann sum or Simpson's rule, we approximate the integral by the numerical sum:

$$\frac{1}{P_{sim}} = \int_{z_1}^{z_2} \frac{\exp\left(\Delta G(z)/RT\right)}{D(z)} dz \approx \sum_{z_1}^{z_2} \frac{\exp\left(\Delta G(z)/RT\right)}{D(z)} \Delta z \qquad \text{(Equation S1)}$$

Here *RT* denote the product of the molar gas constant, *R*, and the temperature, *T*. The entire numerator, $\exp(\Delta G(z)/RT)$, is dimensionless, and the denominator, *D*(*z*), has units cm^2 s^{-1}. When multiplied by the width Δz $(in\ cm)$, the units of reciprocal permeability are cm^{-1} s.

1.2 Unit analysis of the Green-Kubo relation (Equation S2)

One outstanding problem related to Equation S1 is how to obtain the diffusivity *D*(*z*). In neat solvents, the *D*(*z*) diffusion coefficients can be calculated from the force autocorrelation functions (via Green-Kubo relations) (Green, 1954; Kubo, 1957). The Green-Kubo relations have emerged as a powerful avenue to the diffusion constant (Harris et al., 2024), by linking the integral of the force autocorrelation function, $C_F = \int_0^{\infty} \langle \Delta F_z(z,t) \Delta F_z(z,0) \rangle\, dt$, to the *z* diffusion constant, *D*(*z*) (cm^2 s^{-1}), as described in Equation S2. Here by use of either Riemann sum or Simpson's rule, we approximate the integral by the numerical sum along N_{lags}:

$$D(z) = \frac{(RT)^2}{C_F} = \frac{(RT)^2}{\int_0^{\infty} \langle \Delta F_z(z,t) \Delta F_z(z,0) \rangle dt} \qquad \text{(Equation S2)}$$

$$\approx \frac{(RT)^2}{\sum_0^{N_{lag}} \langle \Delta F_z(z,t) \Delta F_z(z,0) \rangle \Delta t}$$

$$\approx \frac{(RT)^2}{\sum_0^{N_{lag}} \langle \Delta F_z(z,t) \Delta F_z(z,0) \rangle \Delta t}$$

At 310 K, RT corresponds to 2.577 kJ mol^{-1}, and thus, the numerator of the square of the gas constant thermalization factor, $(RT)^2$, works out to be 6.6409 kJ^2 mol^{-2}. We now derive the units of the denominator of Equation S2, namely the integral of the autocorrelation function. For a force time series $\Delta F_z(z,0)$ or $\Delta F_z(z,t)$ with associated units kJ mol^{-1} nm^{-1}, the autocorrelation function $\langle \Delta F_z(z,t) \Delta F_z(z,0) \rangle$ has units kJ^2 mol^{-2} nm^{-2}. Upon integrating the autocorrelation function or approximating by numerical integration along N_{lags}, the resulting units are kJ^2 mol^{-2} nm^{-2} ns. As such, $D(z)$ calculated by the Green-Kubo relation (Equation S3), yields $D(z)$ with the units nm^2 ns^{-1}.

$$D(z)\left(\frac{nm^2}{ns}\right) = \frac{(RT)^2}{C_F} = \frac{(RT)^2}{\int_0^{\infty} \langle \Delta F_z(z,t) \Delta F_z(z,0) \rangle dt} \quad \text{(Equation S3)}$$

$$\approx \frac{(RT)^2}{\sum_0^{N_{lag}} \langle \Delta F_z(z,t) \Delta F_z(z,0) \rangle \Delta t}$$

$$= \frac{6.6409}{\sum_0^{N_{lag}} \langle \Delta F_z(z,t) \Delta F_z(z,0) \rangle \Delta t} \times \left(\frac{kJ^2 mol^{-2}}{kJ^2 mol^{-2} nm^{-2}\, ns}\right)$$

$$= \frac{6.6409}{\sum_0^{N_{lag}} \langle \Delta F_z(z,t) \Delta F_z(z,0) \rangle \Delta t} \times \left(\frac{1}{nm^{-2}\, ns}\right)$$

We worked out the correction factor to convert to standardized units of cm^2 s^{-1}. This factor is obtained as follows: cm^2 s^{-1} = $1x10^{-5}$ nm^2 ns^{-1}. Thus, extracted Green-Kubo diffusivities need to be corrected by a factor of $1x10^{-5}$ (Equation S4):

$$D(z)\left(\frac{cm^2}{s}\right) = 1 \times 10^{-5} \times \frac{6.6409}{\sum_0^{N_{lag}} \langle \Delta F_z(z,t) \Delta F_z(z,0) \rangle \Delta t} \times \left(\frac{1}{nm^{-2}\, ns}\right) \quad \text{(Equation S4)}$$

$$= \frac{6.6409 \times 10^{-5}}{\sum_0^{N_{lag}} \langle \Delta F_z(z,t) \Delta F_z(z,0) \rangle \Delta t} \times \left(\frac{1}{cm^{-2}\, s}\right)$$

The input of $D(z)$ into Equation S1 should be in units of cm^2 s^{-1} to ensure consistency with the resulting permeability output in cm s^{-1}.

1.3 Approximating the limit of the Green Kubo integral by a Gamma function (Equation S6)

The Green-Kubo expressions have proven a successful avenue to estimate transport coefficients but suffer from the common problem that the integral of the correlation function typically does not converge (Español et al., 2019). A solution proposed by the group of Ed Lyman is to introduce an analytic approximation to the long-time behaviour ($t \to \infty$) of the Green-Kubo integral in the autocorrelation function and then fit the early part of the force autocorrelation function $C_z = \int_0^{\infty} \langle \Delta F_z(z,t) \Delta F_z(z,0) \rangle \, dt$ onto a general exponential (Fitzgerald et al., 2023) as that of Equation S5:

$$C_z = \langle \Delta F_z(z,t) \Delta F_z(z,0) \rangle = a exp(-bt^{-c}) \qquad \text{(Equation S5)}$$

The result of said fit is a set of coefficients (a, b, c).

$$C_F = \int_0^{\infty} \langle \Delta F_z(z,t) \Delta F_z(z,0) \rangle \, dt = a \; (b^{-c}) \Gamma(1 + c) \qquad \text{(Equation S6)}$$

The resulting value, C_F, is in units nm^2 ns^{-1}. In order to input C_F into Equation S2 to obtain $D(z)$ in consistent units (cm^2 s^{-1}), a scaling factor needs to be applied to the value of C_F (nm^2 ns^{-1}) of the type cm^2 s^{-1} = $1x10^{-5}$ nm^2 ns^{-1}.

$$C_F \text{ (cm}^2\text{ s}^{-1}) = 1x10^{-5} \; C_F \text{ (nm}^2\text{ ns}^{-1})$$

1.4 The Green-Kubo relations and the Fluctuation-Dissipation Theorem for mobility

It was shown by Einstein,(Einstein, 1905) and later discussed by (Kubo, 1966) that the friction coefficient mγ of a Brownian particle is related to the diffusion constant *D* of the travelling particle:

$$D = \frac{kT}{m\gamma} \qquad \text{(Equation S7)}$$

Under a potential *V*(*x*) the net flux *j* of the particle present at concentration *c* travelling along *x* with drift velocity *v* is:

$$j(\mathrm{x}) = -D \frac{\partial c(\mathrm{x})}{\partial \mathrm{x}} + v_d c(\mathrm{x}) \qquad \text{(Equation S8)}$$

For a particle travelling along x in a given time *t*, there is a probability distribution of the location of the particle, as such, the diffusion constant can be written in terms of said probability distribution as an ensemble in thermal equilibrium:

$$D = \lim_{t \mapsto \infty} \frac{1}{2t} \langle x(t) - x(0) \rangle^2 \qquad \text{(Equation S9)}$$

Rearranging:

$$x(t) - x(0) = \int_0^t v(t')dt' \qquad \text{(Equation S10)}$$

We can re-write equation S9 as:

$$\begin{aligned} D &= \lim_{t \mapsto \infty} \frac{1}{2t} \int_0^t dt_1 \int_0^t dt_2 \, \langle v(t_1) v(t_2) \rangle \\ &= \lim_{t \mapsto \infty} \frac{1}{t} \int_0^t dt_1 \int_0^{t-t_1} dt' \, \langle v(t_1) v(t_1 + t') \rangle \\ &= \int_0^\infty \langle v(t_0) v(t_1 + t') \rangle dt \end{aligned} \qquad \text{(Equation S11)}$$

The Einstein relation for the mobility of the particle (dimensionless), the inverse of the friction coefficient mγ, is therefore an expression of the Fluctuation-Dissipation Theorem (Kubo, 1966), and is given by:

$$\mu = \frac{1}{m\gamma} = \frac{D}{kT} = \frac{1}{kT} \int_0^\infty \langle v(t_0) v(t_1 + t') \rangle dt \qquad \text{(Equation S12)}$$

For a mol of particles ($RT = kT/N_A$), the molar mobility $\bar{\mu}$ would be:

$$\bar{\mu} = \frac{1}{m\gamma N_A} = \frac{D}{RT} = \frac{1}{RT} \int_0^\infty \langle v(t_0) v(t_1 + t') \rangle dt \qquad \text{(Equation S13)}$$

1.5 The Green-Kubo relations and the Fluctuation-Dissipation Theorem for force

For a single particle, the Fluctuation-Dissipation Theorem, states that the friction coefficient of said particle is related to the force fluctuations ΔF experienced in that window:

$$\frac{1}{m\gamma} = \frac{D}{kT} = \frac{1}{kT} \int_0^\infty \langle \Delta F(0) \Delta F(t) \rangle dt \qquad \text{(Equation S14)}$$

For a mol of particles, the Fluctuation-Dissipation Theorem, states that the molar friction coefficient of said ensemble of particles is related to the force fluctuations ΔF (J mol^{-1} nm^{-1}) experienced in that window:

$$\Xi = \frac{1}{m\gamma N_A} = \frac{D}{RT} = \frac{1}{RT} \int_0^\infty \langle \Delta F(0) \Delta F(t) \rangle dt \qquad \text{(Equation S15)}$$

For the diffusion coefficient D_z, we therefore arrive at the formula for Equation 5 (Green-Kubo relations) as:

$$D_z = \frac{RT}{m\gamma N_A} = \frac{RT}{\Xi} = \frac{RT}{\frac{1}{RT}\int_0^{\infty}\langle \Delta F_z(0)\Delta F_z(t)\rangle dt} = \frac{(RT)^2}{\int_0^{\infty}\langle \Delta F_z(0)\Delta F_z(t)\rangle dt} \quad \text{(Equation S16)}$$

2. Figures

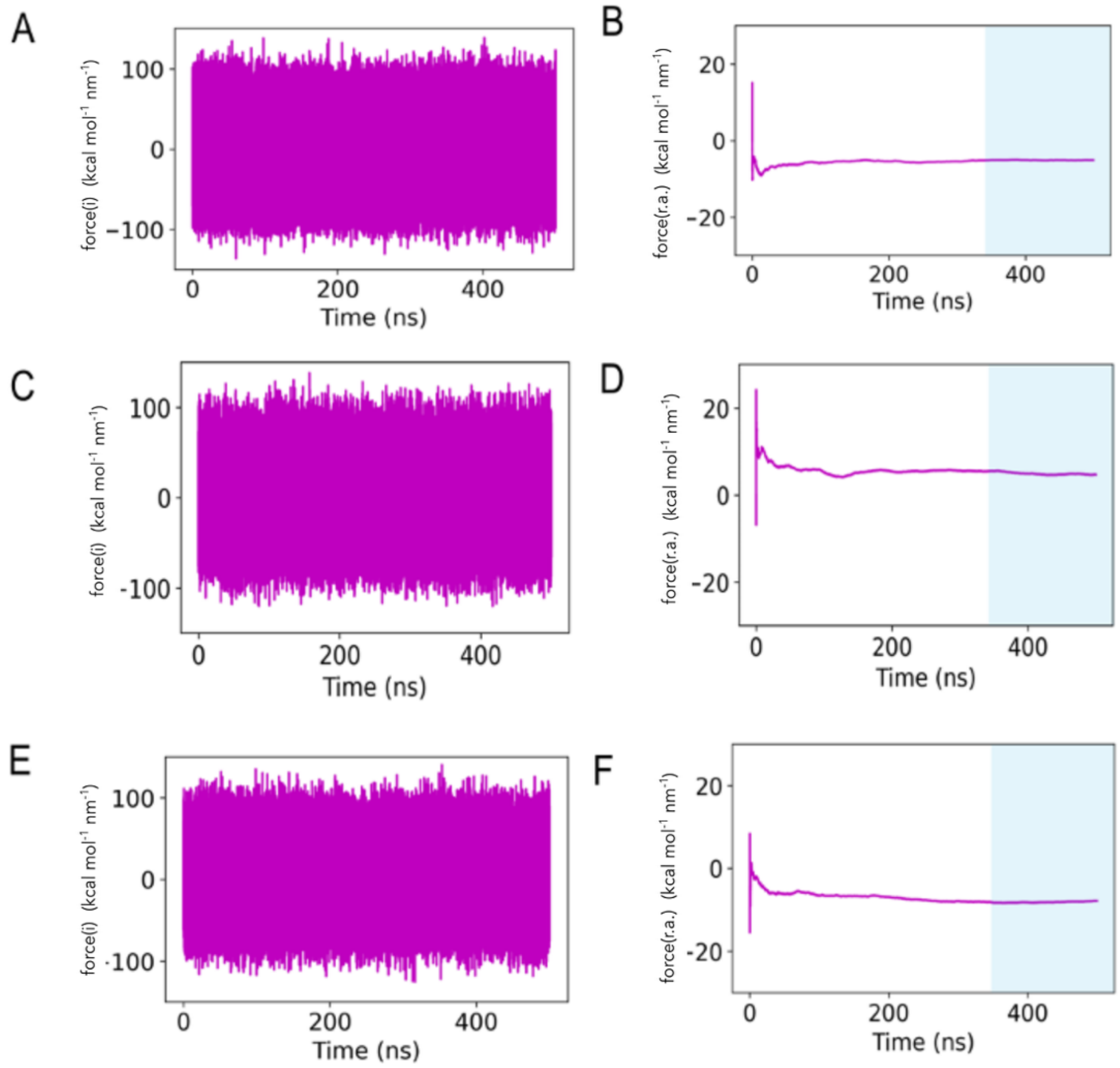

Figure S1. **Convergence assessment of the force sampling over 500 ns production runs demonstrated for ethanol, caffeine and temozolomide**. (A, C, E) Raw force profile output from the *gmx pull* code for ethanol, caffeine and temozolomide, respectively. (B, D, F) Running average of the raw force profile output for ethanol, caffeine and temozolomide, respectively, revealing that the running average reaches a predictable plateau after t = 350 ns, thus allowing for the correct estimation of $\langle F(z) \rangle$ as input into both equations 2 and 3. For purposes of ease of visualization, units of kcal mol^{-1} nm^{-1} are implemented.

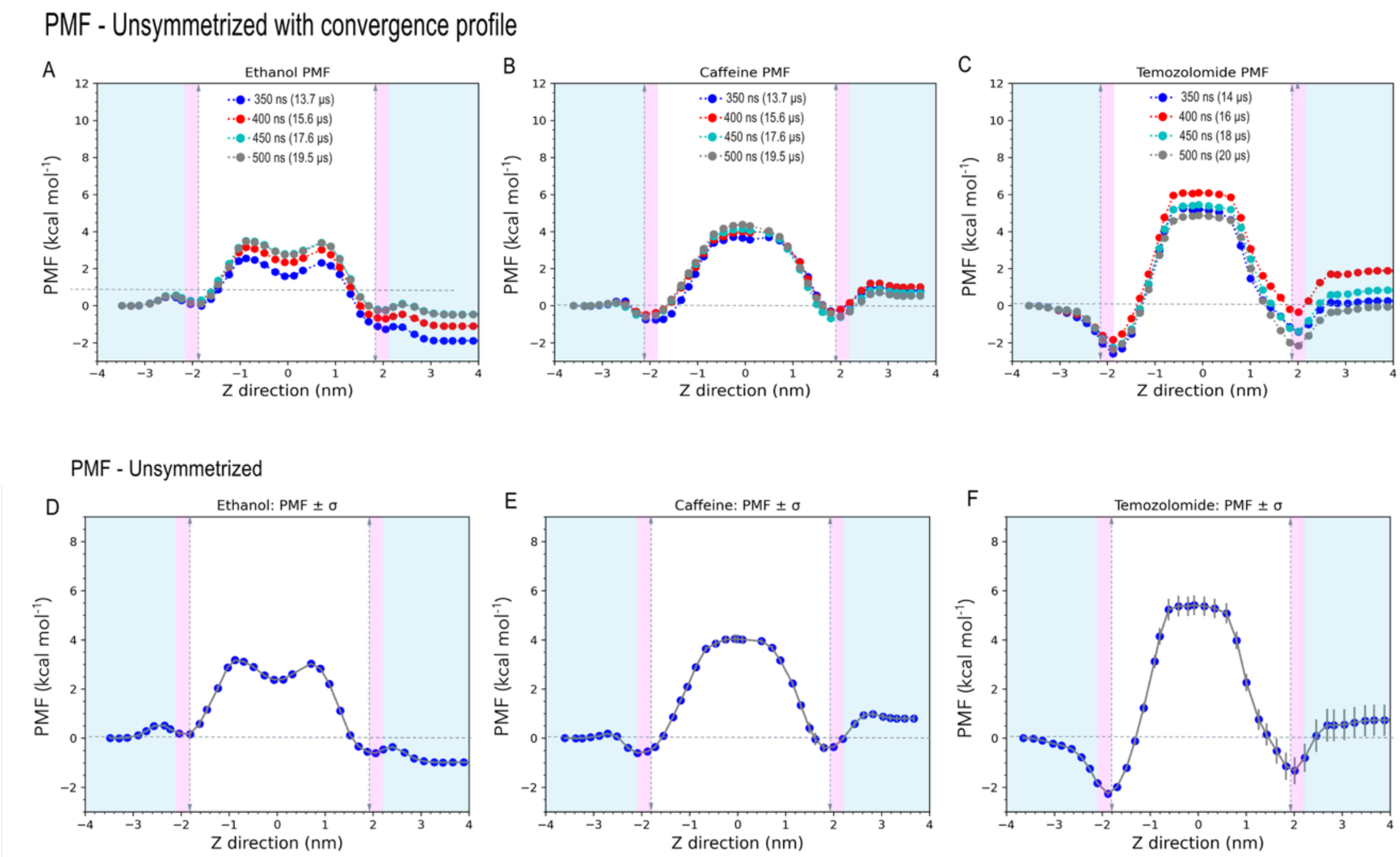

Figure S2. **Convergence assessment of the unsymmetrized potential of mean force (PMF) for (A) ethanol, (B) caffeine and (C) temozolomide, using window simulation lengths of 350, 400, 450 and 500 ns *per window*.** The PMF are obtained by numerical integration of the average force plateau $\int_{-z}^{z} \langle F(z) \rangle dz$. The total simulation time for ethanol and caffeine was 13.7 µs (350 ns), 15.6 µs (400 ns), 17.6 µs (450 ns) and 19.5 µs (500 ns). The total simulation time for temozolomide was 14 µs (350 ns), 16 µs (400 ns), 18 µs (450 ns) and 20 µs (500 ns).

Autocorrelation calculation.

The autocorrelation function (ACF) was computed the autocorrelation function (ACF) using the unnormalized `acf` (`statsmodels.tsa.stattools.acf`) routine from the *statsmodels* library (Seabold and Perktold, 2010) implemented in Python (Rossum, 2009). Analyses were performed in a Jupyter Notebook environment (Kluyver et al., 2016). The ACF was evaluated with an `nlags` parameter of either 10^3 to $5x10^4$ to capture long-range correlations in the force signal, providing insight into periodicity and persistence across the entire recording. We used the default settings of `acf`, which ensures that the procedure automatically subtracts the sample mean from the series before computing the autocorrelations.

The resulting autocorrelations are demonstrated for example windows for ethanol in **Figure S3**, caffeine in **Figure S4** and temozolomide in **Figure S5**. The full trace series is provided in **Figure S6** to **S7** for `nlags` set to $5x10^4$. The comparison of resulting diffusivities (cm^2 s^{-1}) when using `nlags` set to either 10^4 or $5x10^4$, is shown in **Figure 6**.

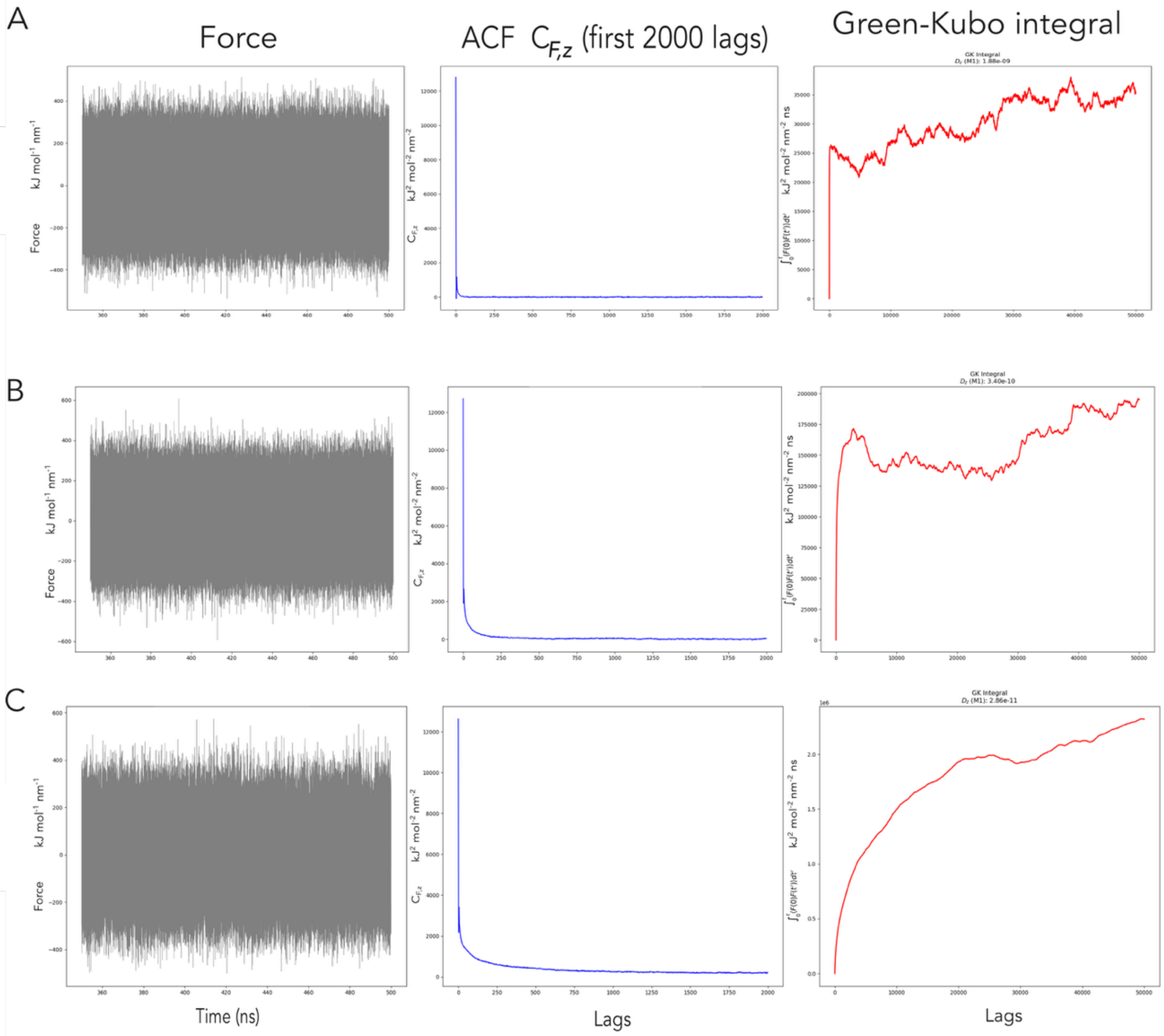

Figure S3. **Autocorrelation analysis for ethanol.** (A) The results presented are for lag time of N = 5 x 10^4 lags. The analysis takes in the (A) raw force (kJ mol^{-1} nm^{-1}), calculates the autocorrelation function in (B) and subsequent integral of the ACF (C).

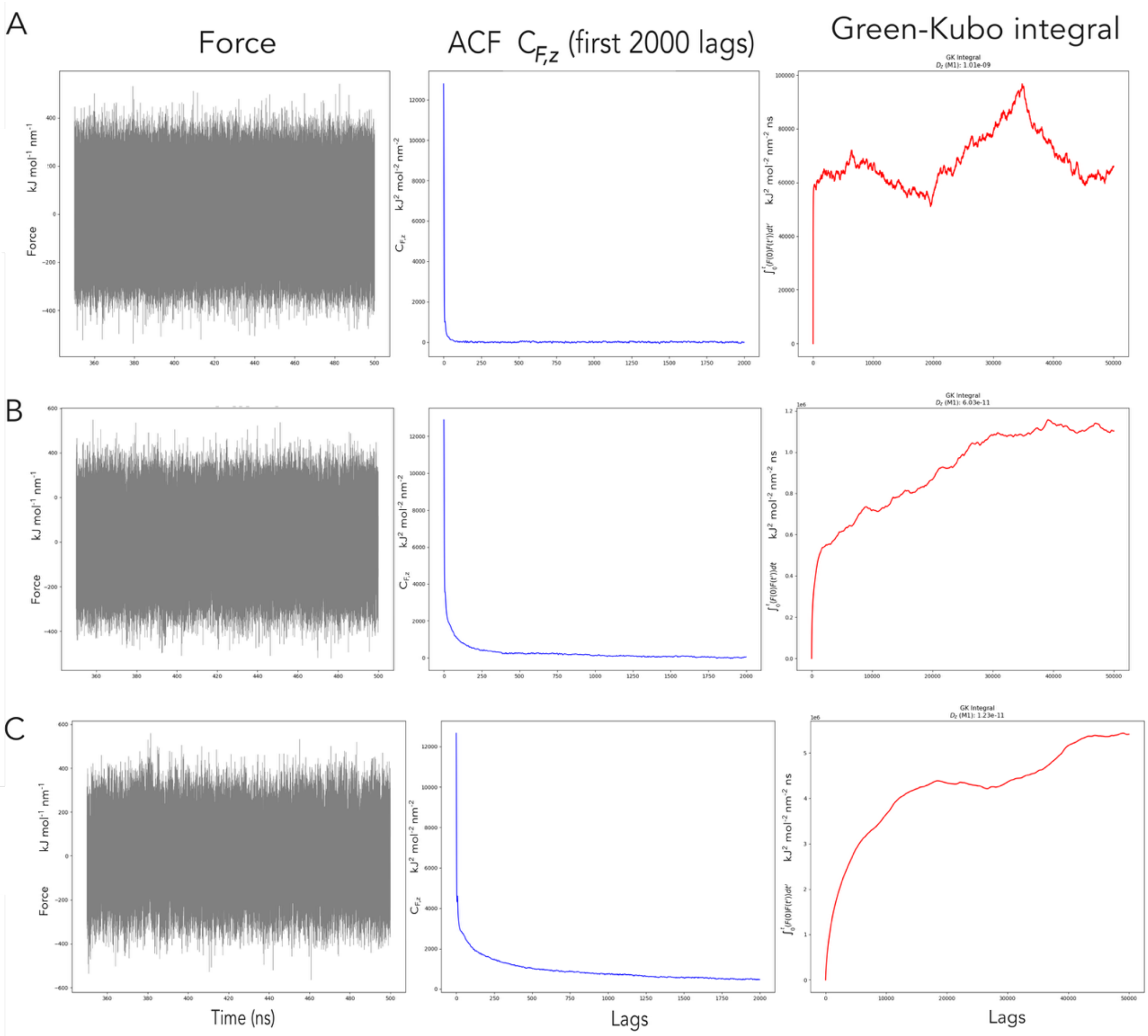

Figure S4. **Autocorrelation analysis for caffeine.** (A) The results presented are for lag time of N = 5 x 10^4 lags. The analysis takes in the (A) raw force (kJ mol^{-1} nm^{-1}), calculates the autocorrelation function in (B) and subsequent integral of the ACF (C).

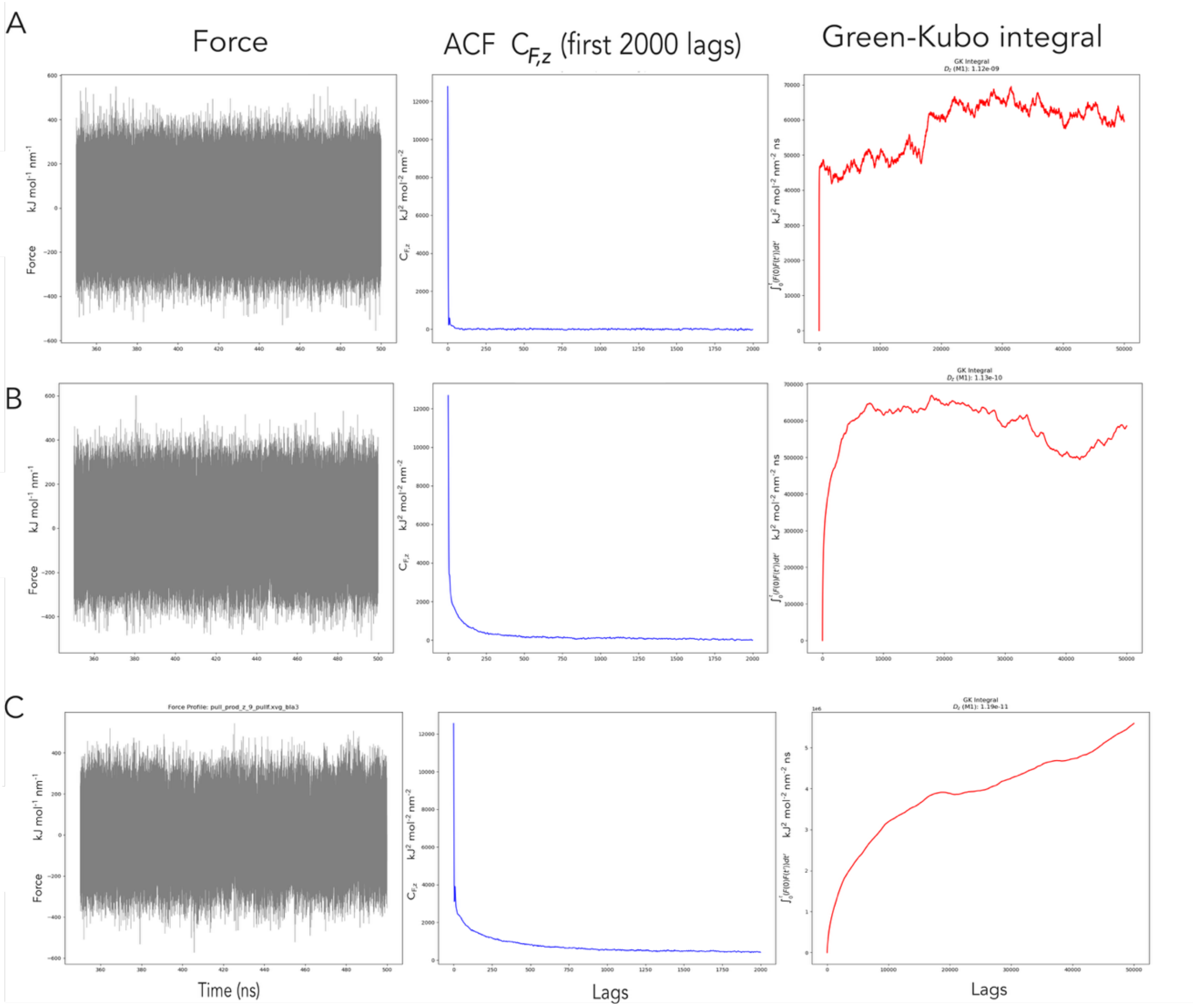

Figure S5. **Autocorrelation analysis for temozolomide.** (A) The results presented are for lag time of $N = 5 \times 10^4$ lags. The analysis takes in the (A) raw force (kJ mol^{-1} nm^{-1}), calculates the autocorrelation function in (B) and subsequent integral of the ACF (C).

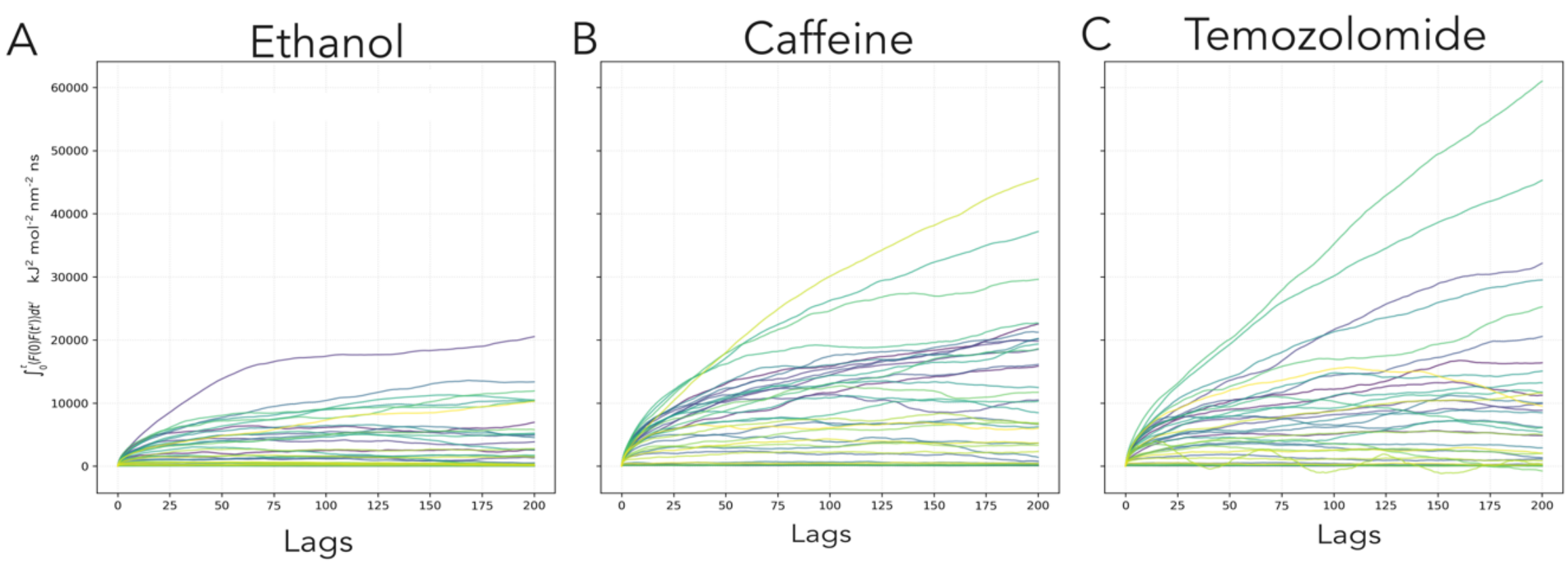

Figure S6. **Full trace for the integral of the autocorrelation for (A) ethanol, (B) caffeine and (C) temozolomide analyzed for interval 350 ns to 500 ns using $N = 5 \times 10^4$ lags**. The analysis takes in the raw force (kJ mol^{-1} nm^{-1}), calculates the autocorrelation function in, and subsequently computes the integral of the ACF.

3. Autocorrelation parameter justification

For purposes of convergence, we rely on lessons learned from the evaluation of the PMF, where a total of 500 ns is needed for satisfactorily meeting the criteria of convergence (**Figure S2**), with us discarding the first 350 ns of the force trace ahead of a convergence analysis.

We benchmark the choices for analysing kinetics in a set of equally spaced pulling windows from the caffeine simulations (windows 05, 10, 15, 20, 25, 30 and 35), with each figure showcasing autocorrelation function analysis for a single force file. We find that the choices made in the paper (50,000 lags; 150 ns window of analysis) are optimal for yielding consistent analysis.

(1) **Number of force values considered**: we observe that while there is not a significant difference between panels (A)-(C), in which the number of force values increases from 500,000 to 1,500,000, it therefore is an appropriate strategy to work with the longest force trace (1,500,000) due to it also being the segment we use for the PMF calculation.

(2) **Time interval between force samples (100 fs).**

Our 500 ns pulling windows produce 5,000,000 force samples, with a printing frequency of 100 fs, that is determined by *pull-nstxout* set to print force samples every 50 timesteps.

(3) **Number of lags**. As we see, the autocorrelation function behaves in a consistent fashion for analysis with $N_{lags} > 10^3$ as indicated with a blue color plateau shading. This pattern repeats for all force profiles (**Figures S7** to **S15**).

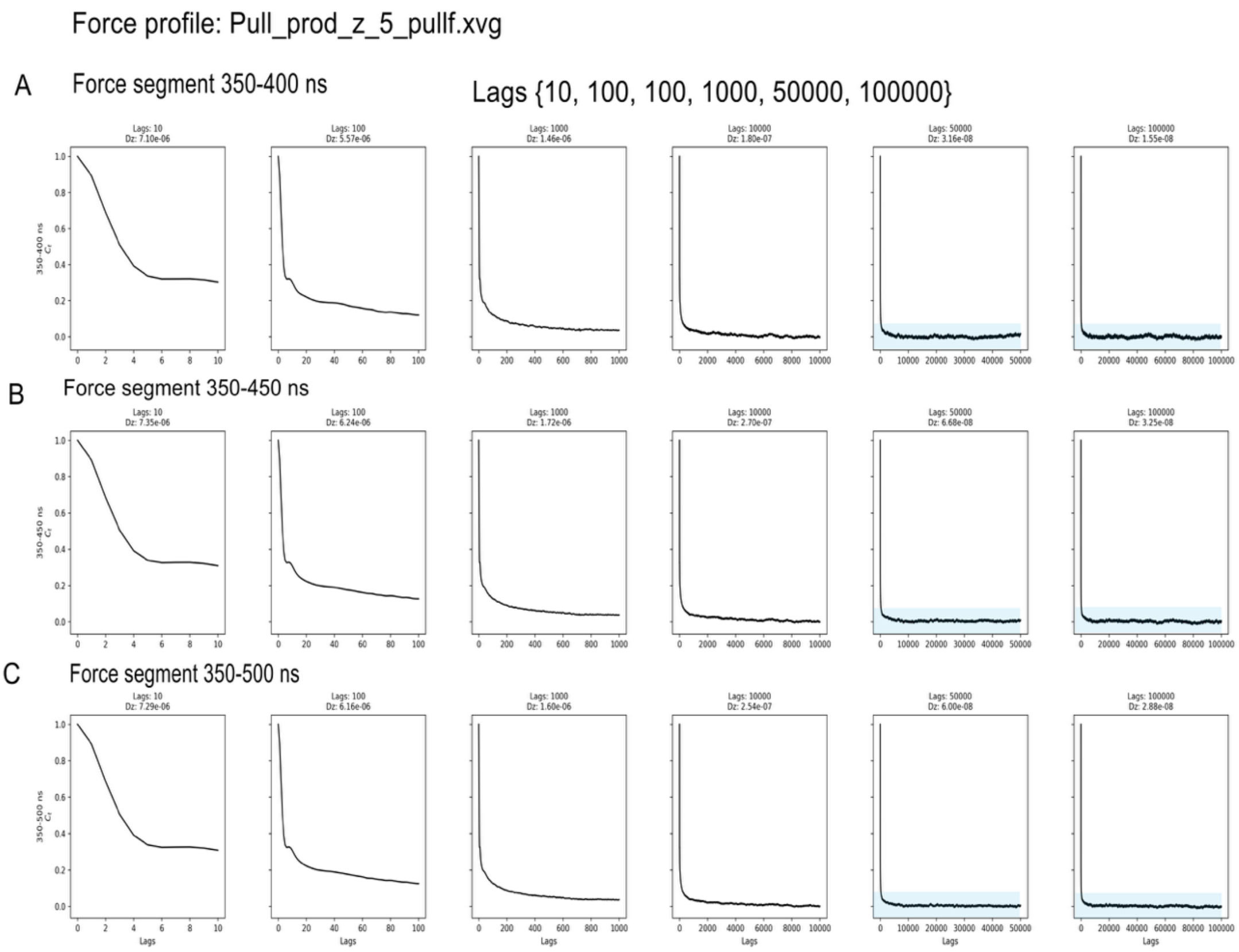

Figure S7. Systematic evaluation of the autocorrelation function of a single force profile pull_prod_z_5_pullf.xvg varying (a) the force profile interval, (b) the lag number. The force segments considered were those segments used for convergence analysis, (i) 350 – 400 ns, (ii) 350 – 450 ns, and (iii) 350 – 500 ns.

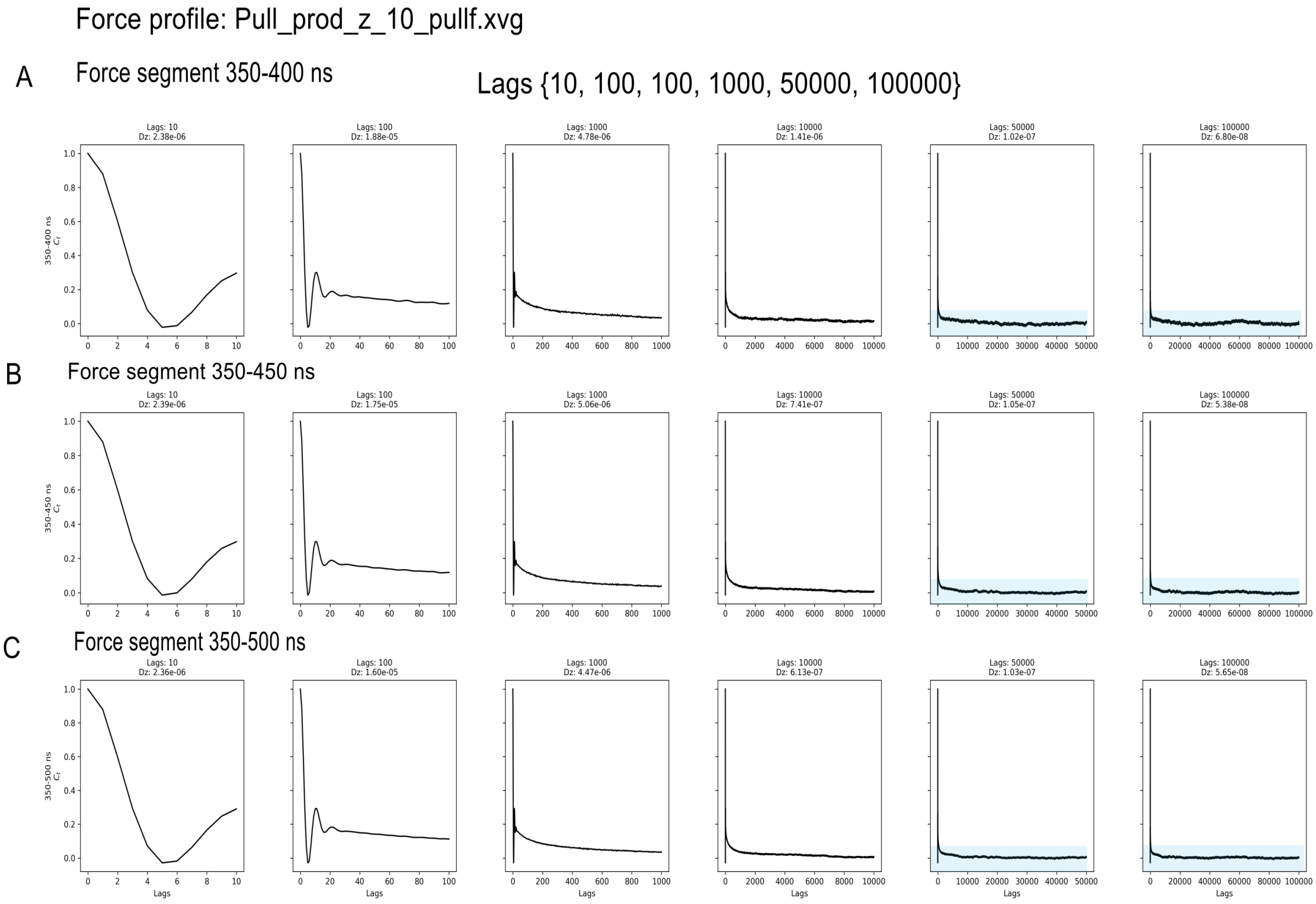

Figure S8. Systematic evaluation of the autocorrelation function of a single force profile pull_prod_z_10_pullf.xvg varying (a) the force profile interval, (b) the lag number. The force segments considered were those segments used for convergence analysis, (i) 350 – 400 ns, (ii) 350 – 450 ns, and (iii) 350 – 500 ns.

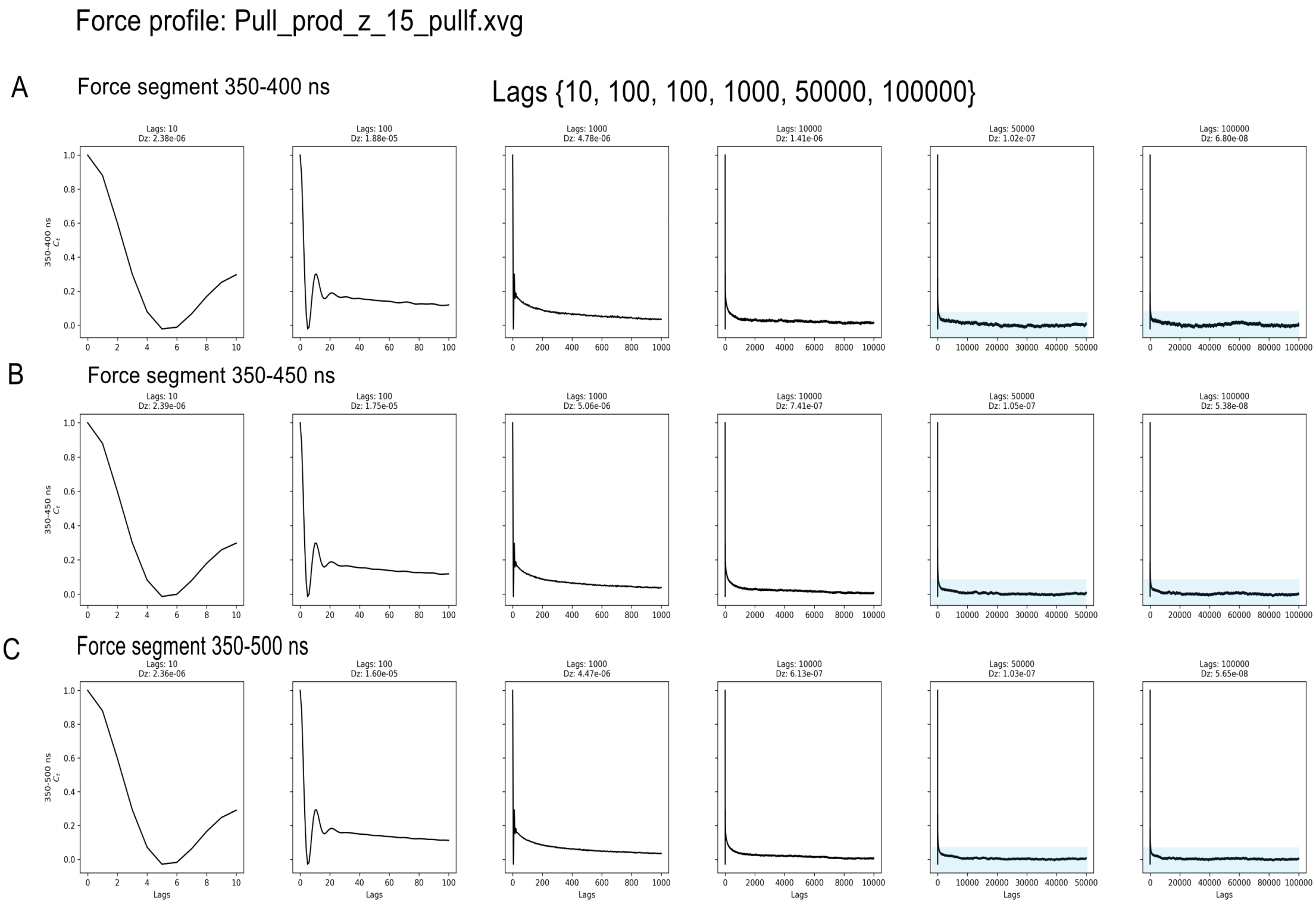

Figure S9. Systematic evaluation of the autocorrelation function of a single force profile pull_prod_z_15_pullf.xvg varying (a) the force profile interval, (b) the lag number. The force segments considered were those segments used for convergence analysis, (i) 350 – 400 ns, (ii) 350 – 450 ns, and (iii) 350 – 500 ns.

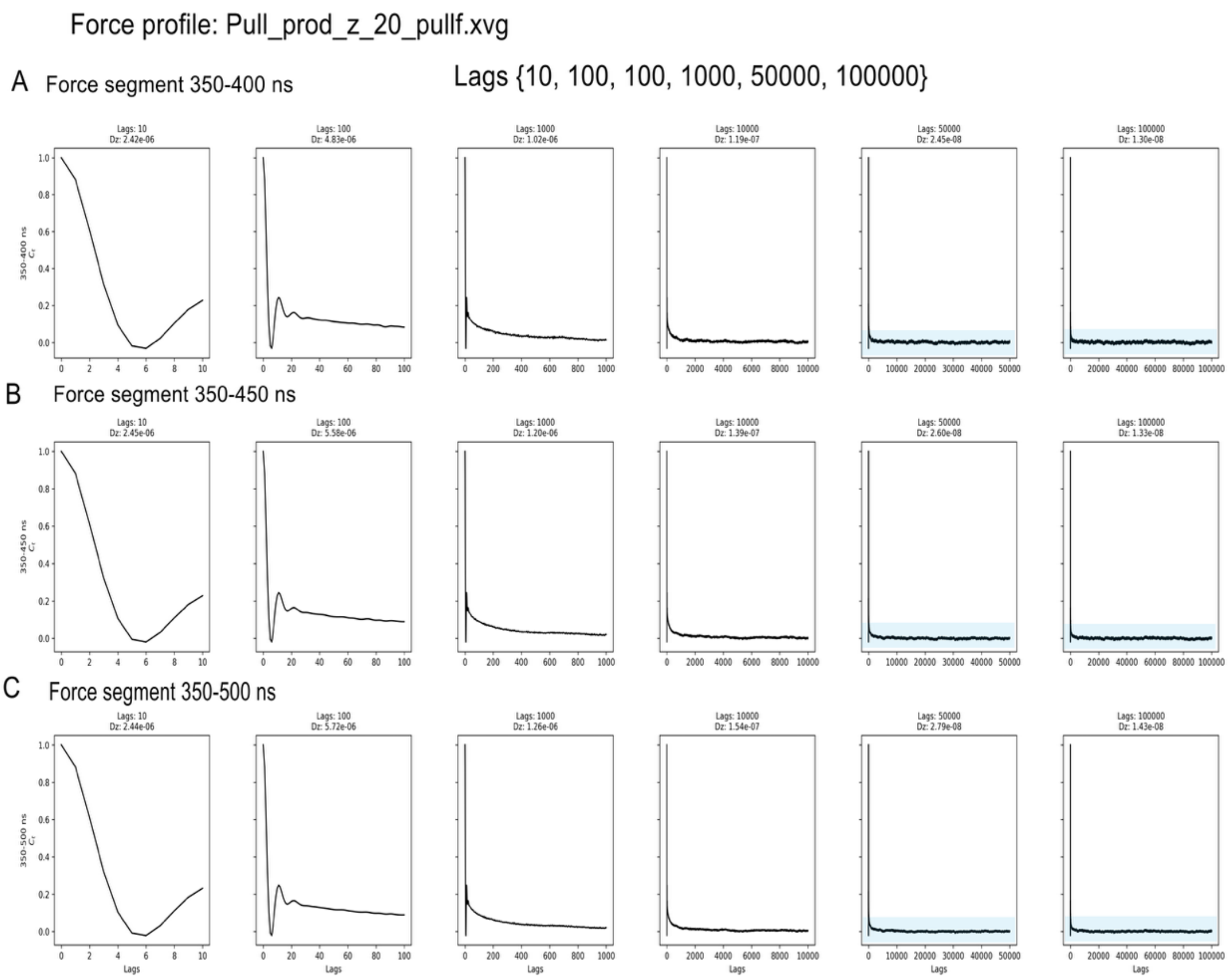

Figure S10. Systematic evaluation of the autocorrelation function of a single force profile pull_prod_z_20_pullf.xvg varying (a) the force profile interval, (b) the lag number. The force segments considered were those segments used for convergence analysis, (i) 350 – 400 ns, (ii) 350 – 450 ns, and (iii) 350 – 500 ns.

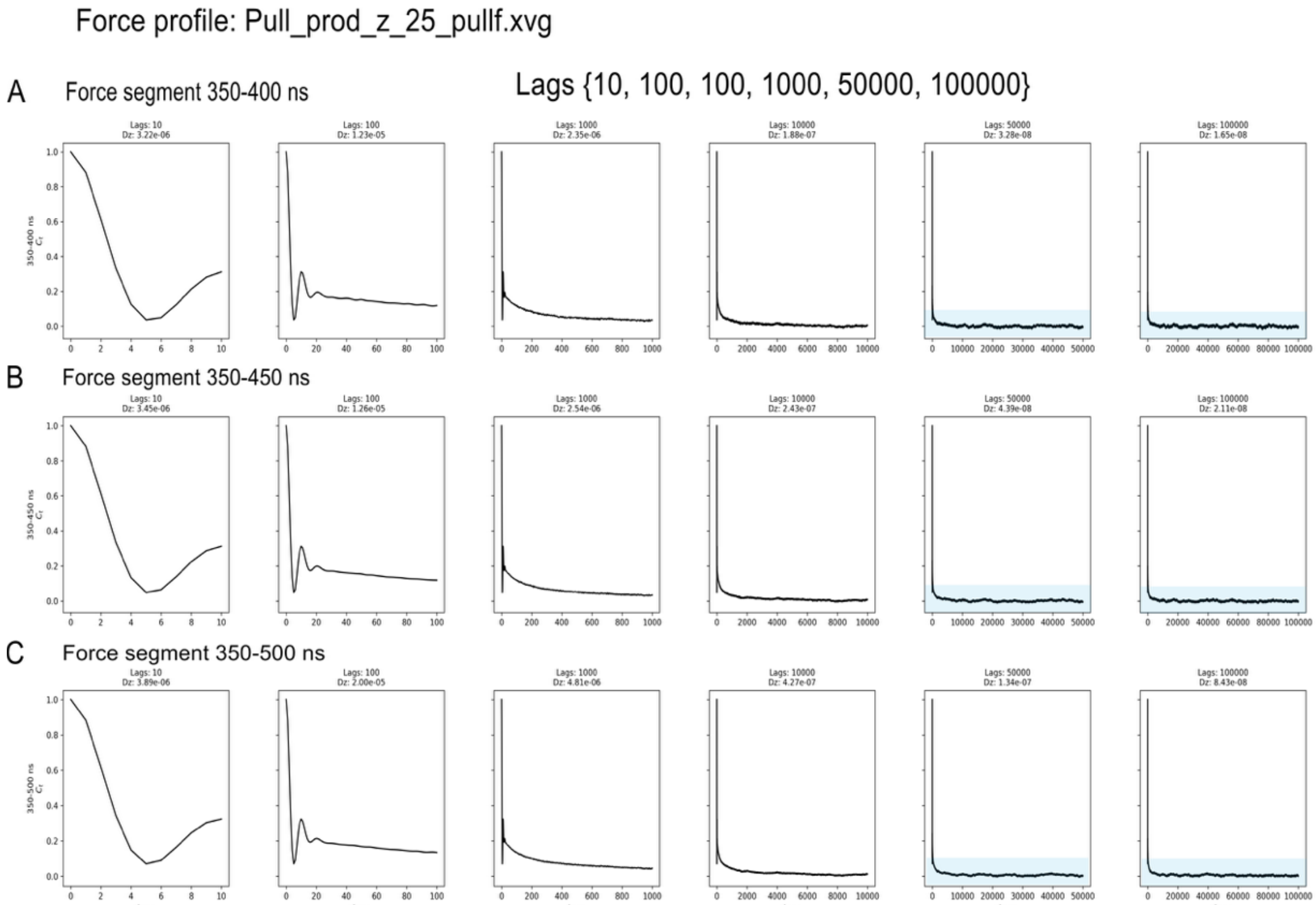

Figure S11. Systematic evaluation of the autocorrelation function of a single force profile pull_prod_z_25_pullf.xvg varying (a) the force profile interval, (b) the lag number. The force segments considered were those segments used for convergence analysis, (i) 350 – 400 ns, (ii) 350 – 450 ns, and (iii) 350 – 500 ns.

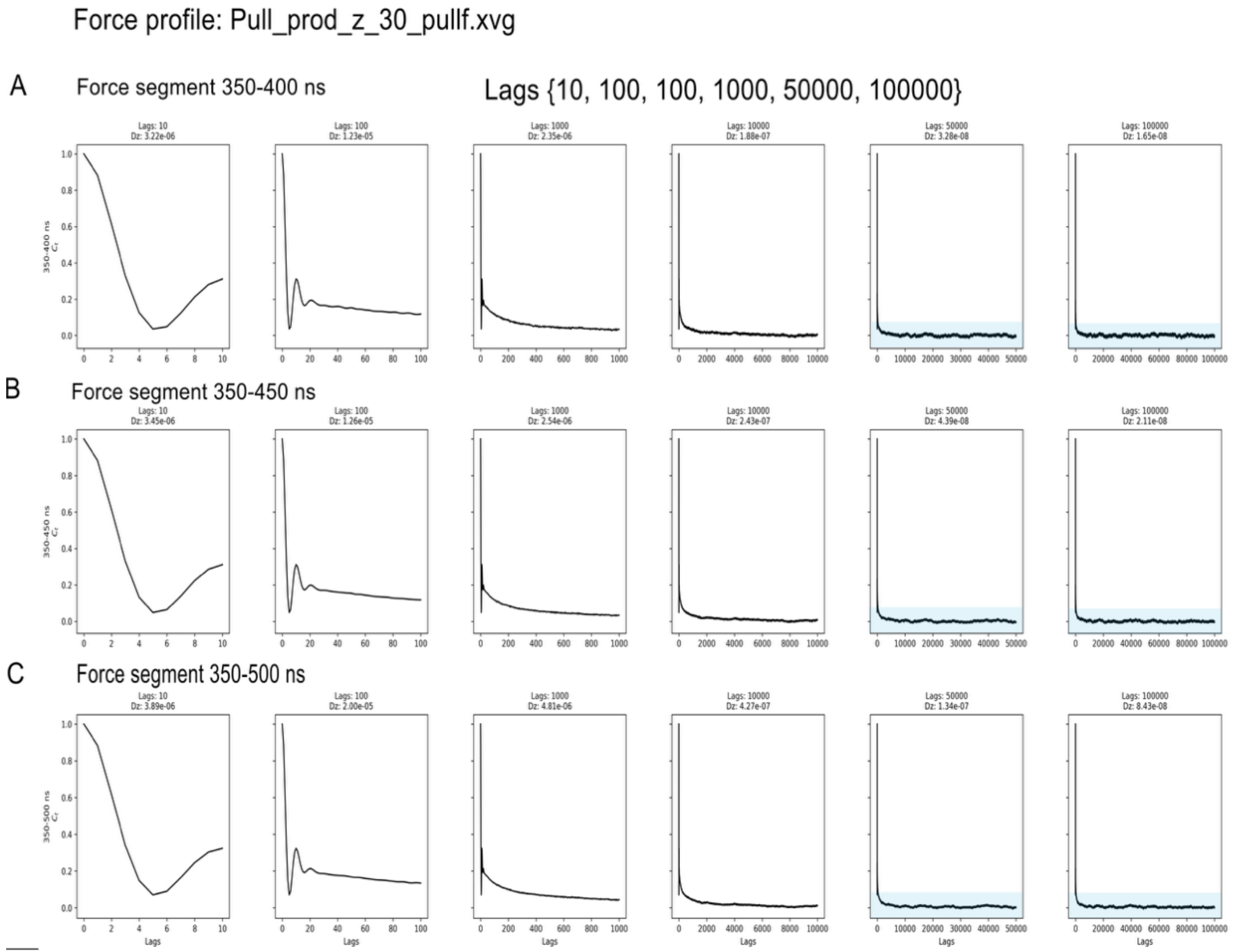

Figure S12. Systematic evaluation of the autocorrelation function of a single force profile pull_prod_z_30_pullf.xvg varying (a) the force profile interval, (b) the lag number. The force segments considered were those segments used for convergence analysis, (i) 350 – 400 ns, (ii) 350 – 450 ns, and (iii) 350 – 500 ns.

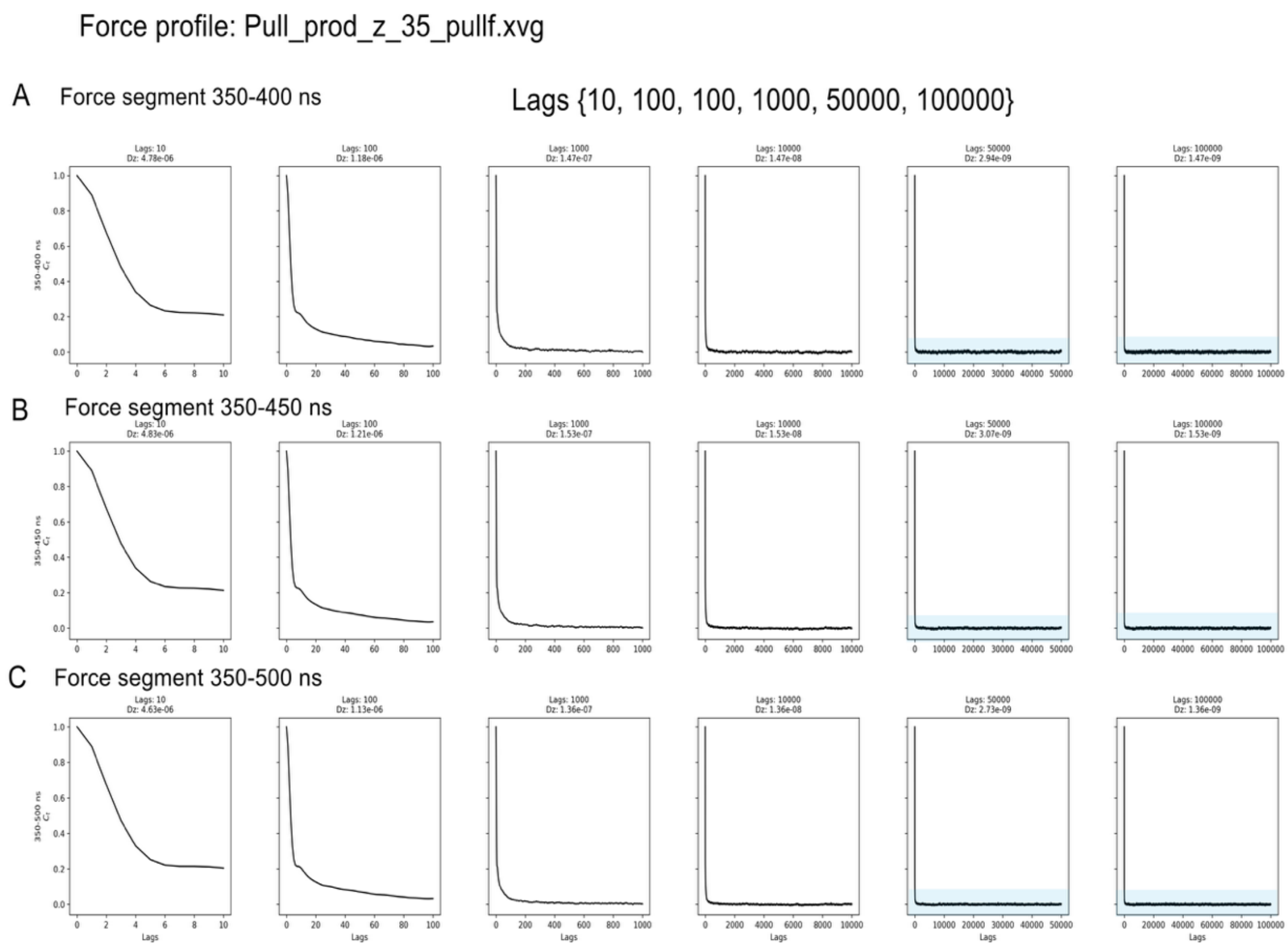

Figure S13. Systematic evaluation of the autocorrelation function of a single force profile pull_prod_z_35_pullf.xvg varying (a) the force profile interval, (b) the lag number. The force segments considered were those segments used for convergence analysis, (i) 350 – 400 ns, (ii) 350 – 450 ns, and (iii) 350 – 500 ns.

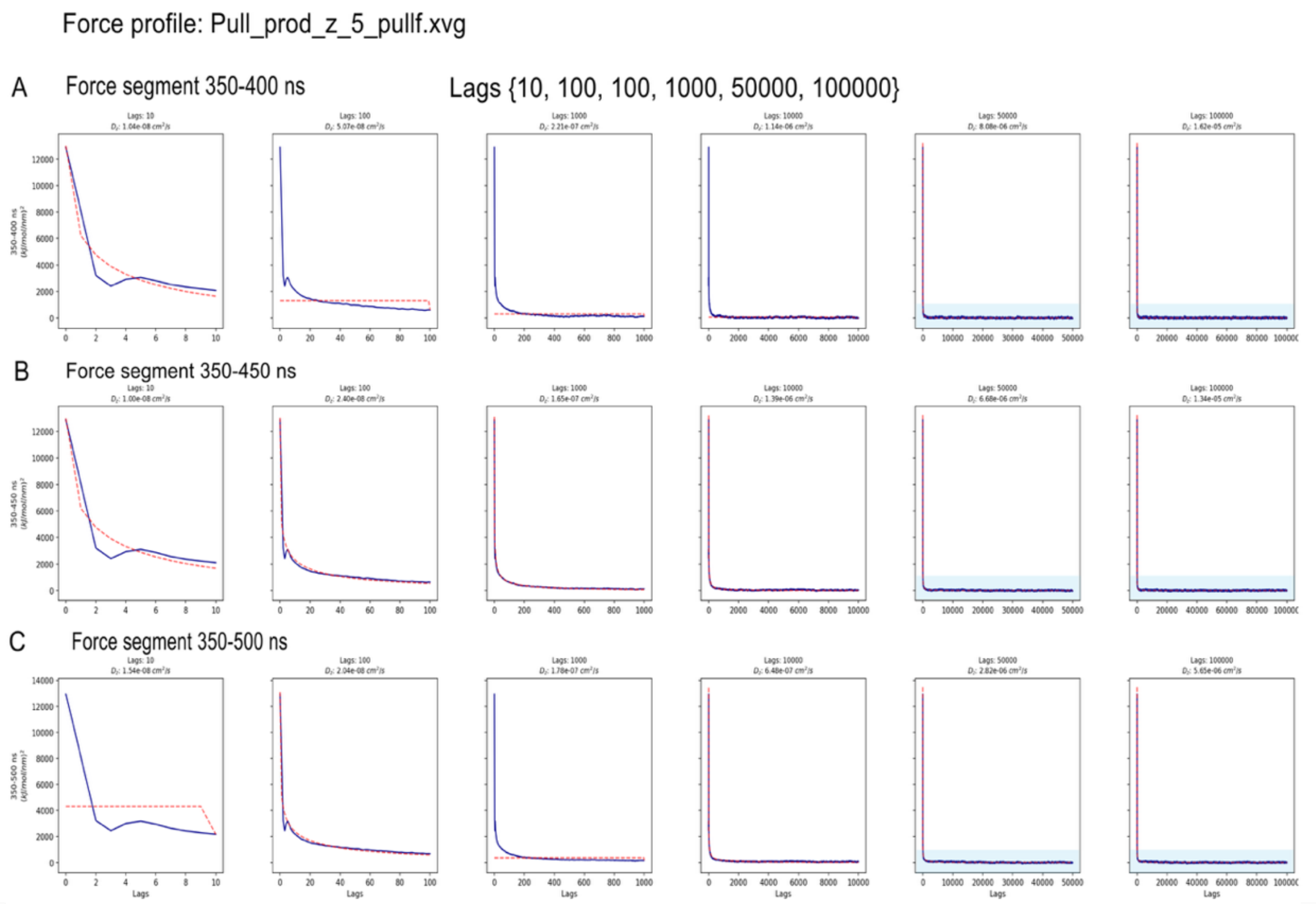

Figure S14. Systematic evaluation of the autocorrelation function of a single force profile pull_prod_z_05_pullf.xvg of ethanol varying (a) the force profile interval, (b) the lag number. The force segments considered were those segments used for convergence analysis, (i) 350 – 400 ns, (ii) 350 – 450 ns, and (iii) 350 – 500 ns.

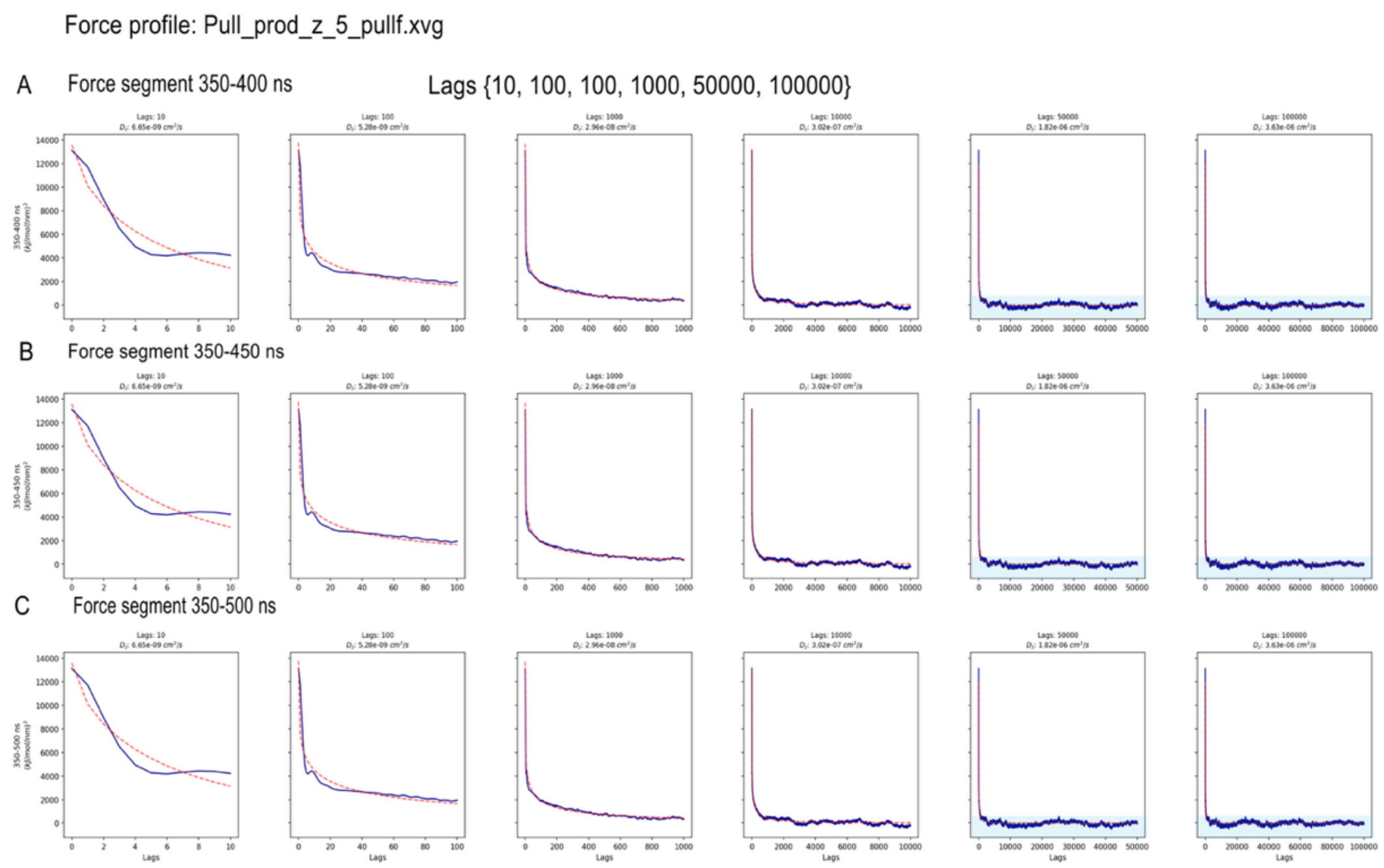

Figure S15. Systematic evaluation of the autocorrelation function of a single force profile pull_prod_z_05_pullf.xvg of temozolomide varying (a) the force profile interval, (b) the lag number. The force segments considered were those segments used for convergence analysis, (i) 350 – 400 ns, (ii) 350 – 450 ns, and (iii) 350 – 500 ns.

4. **Comparison of PMF calculated from *gmx wham* to those from numerical integration.**

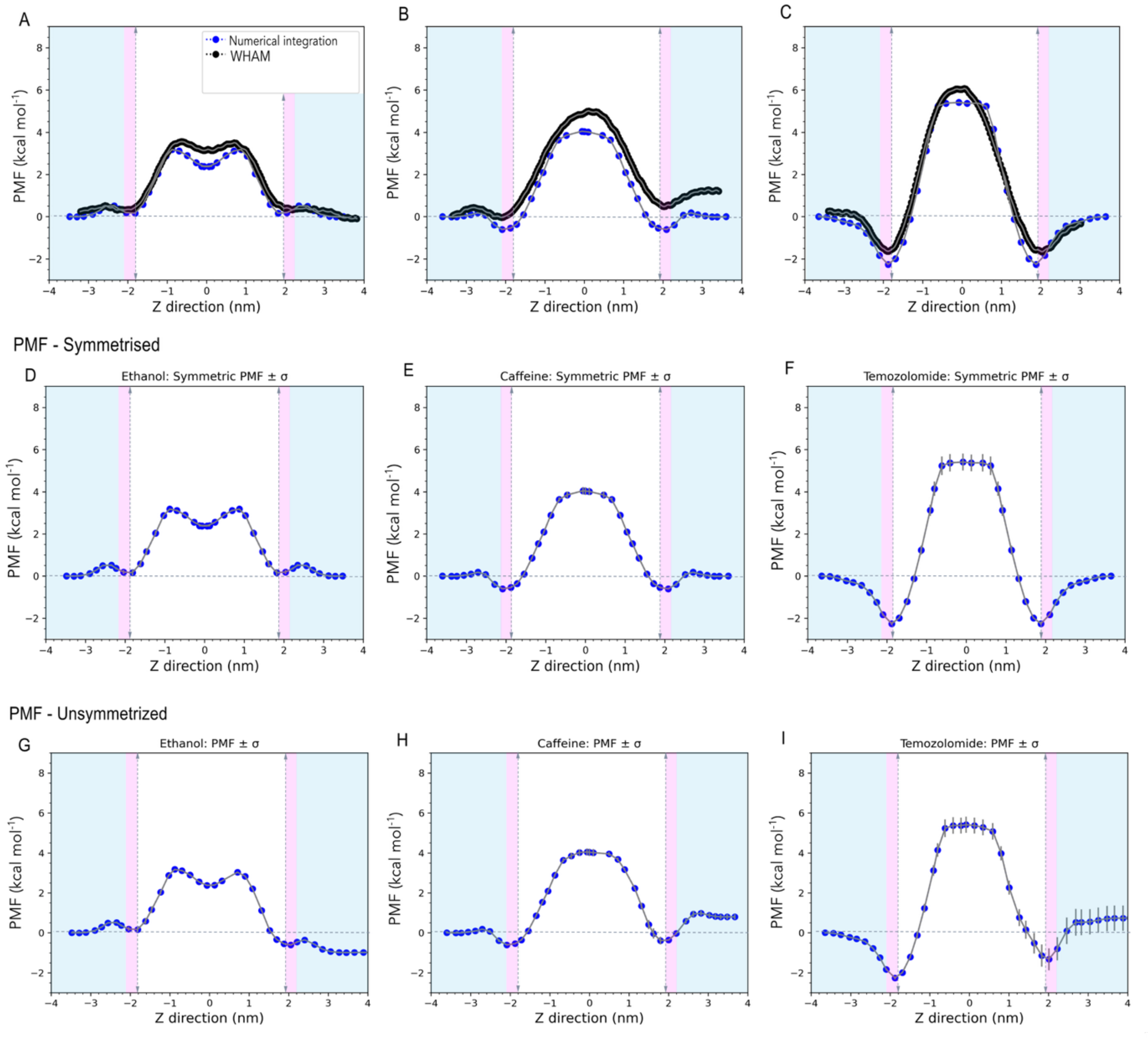

Figure S16. **Individual potential of mean force (PMF) curves for ethanol, caffeine and temozolomide, respectively, using (A)-(C) gmx wham on simulation windows of 500 ns, and compared to PMFs obtained from numerical integration of the average force profile. We depict the (D)-(F) symmetrized, and (G)-(I) unsymmetrized versions.** Numerical integration of the average force, by $\int_{-z}^{z} \langle F(z) \rangle dz$, and symmetrization is applied at $z = 0$. The error bars are calculated as the standard deviation (σ) of the PMF at 3 timepoints: 400 ns, 450 ns and 500 ns. The total simulation time was 19.5 µs for ethanol (39 windows), 19.5 µs for caffeine (39 windows) and 20 µs for temozolomide (40 windows).

1. Tables

Table S1. CHARMM General Force Field (CGenFF) charge models for the drugs being considered: temozolomide, caffeine and ethanol.

Molecule	CGenFF atom type	Partial charge	
Temozolomide		Charge 0.0	
O1	OG2D4	-0.422	
O2	OG2D1	-0.516	
N1	NG2RC0	-0.444	
N2	NG2R61	0.109	
N3	NG2R50	-0.507	
N4	NG2R62	-0.274	
N5	NG2R62	-0.117	
N6	NG2S2	-0.770	
C1	CG2RC0	0.174	
C2	CG2R51	-0.186	
C3	CG2R63	0.855	
C4	CG2R53	0.491	
C5	CG331	-0.268	
C6	CG2O1	0.721	
H1	HGR52	0.134	
H2	HGA3	0.090	
H3	HGA3	0.090	
H4	HGA3	0.090	
H5	HGP1	0.375	
H6	HGP1	0.375	
Caffeine		Model 1 Charge 0.0	Model 2 SILCS-BIO Charge 0.0
C1	CG331	-0.269	-0.276
N1	NG2R51	-0.111	-0.082
C2	CG2R53	0.317	0.265
N2	NG2R50	-0.579	-0.745
C3	CG2RC0	-0.481	-0.172
C4	CG2RC0	0.413	0.475
C5	CG2R63	0.936	0.466
O1	OG2D4	-0.514	-0.488
N3	NG2R61	-0.388	0.345
C6	CG2R63	0.693	0.027
O2	OG2D4	-0.378	-0.443
N4	NG2R61	-0.036	-0.052
C7	CG331	-0.267	-0.078
C8	CG331	-0.267	-0.150
H1	HGA3	0.090	0.090
H2	HGA3	0.090	0.090
H3	HGA3	0.090	0.090
H4	HGR52	0.121	0.098
H5	HGA3	0.090	0.090
H6	HGA3	0.090	0.090
H7	HGA3	0.090	0.090
H8	HGA3	0.090	0.090
H9	HGA3	0.090	0.090
H10	HGA3	0.090	0.090
Ethanol		Charge 0.0	
C1	CG321	0.048	
H1	HGA2	0.090	
C2	CG331	-0.270	
H2	HGA3	0.090	
H3	HGA3	0.090	
H4	HGA3	0.090	
H5	HGA2	0.090	
O	OG311	-0.648	
H6	HGP1	0.420	

Table S2. Green-Kubo components (Equation S1) for ethanol per window: (1) average z value of window, (2) D(z) (cm^{2}s^{-1}) from Green-Kubo relation using the converged part of the force (350 to 500 ns) and 5 x 10^4 lags. D(z) is calculated by analytical solution of the fit to a general exponential function (Fitzgerald et al., 2023), G(z) (kJ mol^{-1}) from integration of force profile, ISD equation S1 component $\exp(\Delta G(z)/RT)/D(z)$ (cm^{-2} s), ISD equation S1 component $\Delta z * \exp(\Delta G(z)/RT)/D(z)$ (cm^{-1} s), Δz (cm).

Ethanol z (nm)	D(z) (cm^{2}s^{-1})	G(z) (kJ mol^{-1})	$\frac{\exp(\Delta G(z)/RT)}{D(z)}$ cm^{-2} s	$\frac{\exp(\Delta G(z)/RT)}{D(z)}\Delta z$ cm^{-1} s	Δz (cm)
-3.48	2.10×10^{-4}	0.00	4.80×10^{3}	9.10×10^{-5}	1.89×10^{-8}
-3.29	2.10×10^{-4}	-0.01	5.00×10^{3}	8.40×10^{-5}	1.69×10^{-8}
-3.12	2.00×10^{-4}	0.06	5.90×10^{3}	1.30×10^{-4}	2.26×10^{-8}
-2.90	4.80×10^{-5}	0.48	3.30×10^{4}	5.60×10^{-4}	1.69×10^{-8}
-2.73	2.50×10^{-5}	1.19	9.10×10^{4}	1.40×10^{-3}	1.59×10^{-8}
-2.57	2.80×10^{-6}	2.08	8.20×10^{5}	1.80×10^{-2}	2.21×10^{-8}
-2.35	2.10×10^{-6}	2.16	8.80×10^{5}	1.00×10^{-2}	1.20×10^{-8}
-2.23	3.60×10^{-6}	1.52	3.80×10^{5}	7.10×10^{-3}	1.85×10^{-8}
-2.04	3.20×10^{-7}	0.80	4.10×10^{6}	9.30×10^{-2}	2.26×10^{-8}
-1.82	1.20×10^{-6}	0.69	2.20×10^{6}	4.40×10^{-2}	1.99×10^{-8}
-1.62	1.20×10^{-6}	2.44	5.50×10^{6}	8.70×10^{-2}	1.57×10^{-8}
-1.46	5.20×10^{-7}	4.84	5.20×10^{7}	1.20×10^{0}	2.28×10^{-8}
-1.23	5.60×10^{-7}	8.49	1.90×10^{8}	3.80×10^{0}	2.02×10^{-8}
-1.03	2.80×10^{-7}	12.02	6.10×10^{8}	9.40×10^{0}	1.53×10^{-8}
-0.88	7.80×10^{-8}	13.27	2.00×10^{9}	3.60×10^{1}	1.84×10^{-8}
-0.69	5.00×10^{-7}	13.00	2.20×10^{8}	4.40×10^{0}	2.03×10^{-8}
-0.49	2.50×10^{-6}	12.09	2.50×10^{7}	5.80×10^{-1}	2.26×10^{-8}
-0.26	6.50×10^{-6}	10.66	7.30×10^{6}	1.50×10^{-1}	2.02×10^{-8}
-0.06	7.30×10^{-6}	9.93	6.60×10^{6}	1.20×10^{-1}	1.90×10^{-8}
0.13	8.20×10^{-6}	9.99	8.30×10^{6}	1.60×10^{-1}	1.91×10^{-8}
0.32	3.50×10^{-6}	10.88	3.90×10^{7}	1.50×10^{0}	3.88×10^{-8}
0.71	3.40×10^{-6}	12.67	2.90×10^{7}	5.60×10^{-1}	1.96×10^{-8}
0.90	2.90×10^{-8}	11.84	1.20×10^{9}	2.30×10^{1}	1.86×10^{-8}
1.09	9.50×10^{-7}	9.22	6.50×10^{6}	1.50×10^{-1}	2.29×10^{-8}
1.32	7.30×10^{-7}	4.68	1.70×10^{6}	3.40×10^{-2}	2.05×10^{-8}
1.52	9.70×10^{-7}	0.51	1.80×10^{6}	3.00×10^{-2}	1.72×10^{-8}
1.69	6.40×10^{-7}	-1.38	3.80×10^{6}	7.30×10^{-2}	1.91×10^{-8}
1.89	1.00×10^{-6}	-2.30	2.60×10^{6}	4.50×10^{-2}	1.70×10^{-8}
2.06	2.10×10^{-7}	-2.55	1.00×10^{7}	1.60×10^{-1}	1.58×10^{-8}
2.21	2.70×10^{-6}	-1.91	6.70×10^{5}	1.30×10^{-2}	1.93×10^{-8}
2.41	4.10×10^{-6}	-1.48	6.20×10^{5}	1.50×10^{-2}	2.47×10^{-8}
2.65	1.40×10^{-5}	-2.42	2.60×10^{5}	5.40×10^{-3}	2.04×10^{-8}
2.86	3.30×10^{-5}	-3.43	1.40×10^{5}	2.80×10^{-3}	2.04×10^{-8}
3.06	9.60×10^{-5}	-3.93	5.10×10^{4}	1.10×10^{-3}	2.13×10^{-8}
3.27	2.10×10^{-4}	-4.10	2.40×10^{4}	4.40×10^{-4}	1.84×10^{-8}
3.46	2.10×10^{-4}	-4.13	2.40×10^{4}	4.90×10^{-4}	2.03×10^{-8}
3.66	2.10×10^{-4}	-4.12	2.40×10^{4}	5.40×10^{-4}	2.31×10^{-8}
3.89	2.10×10^{-4}	-4.10	2.40×10^{4}	4.90×10^{-4}	2.06×10^{-8}
4.10	2.10×10^{-4}	-4.13	4.80×10^{3}	9.90×10^{-5}	2.06×10^{-8}
			1/P = $\sum \frac{\exp(\Delta G(z)/RT)}{D(z)}\Delta z$ (cm^{-1} s)	8.19×10^{1}	
			P (cm s^{-1}) =	1.22×10^{-2}	

Table S3. Green-Kubo components (Equation S1) for caffeine per window: (1) average z value of window, (2) D(z) (cm^{2}s^{-1}) from Green-Kubo relation using the converged part of the force (350 to 500 ns) and 5 x 10^4 lags. D(z) is calculated by solution of the fit to a general exponential function (Fitzgerald et al., 2023), G(z) (kJ mol^{-1}) from integration of force profile, ISD equation S1 component $\exp(\Delta G(z)/RT)/D(z)$ (cm^{-2} s), ISD equation S1 component $\Delta z * \exp(\Delta G(z)/RT)/D(z)$ (cm^{-1} s), Δz (cm).

Caffeine z (nm)	D(z) (cm^{2}s^{-1})	G(z) (kJ mol^{-1})	$\frac{\exp(\Delta G(z)/RT)}{D(z)}$ cm^{-2} s	$\frac{\exp(\Delta G(z)/RT)}{D(z)}\Delta z$ cm^{-1} s	Δz (cm)
-3.60	8.14 × 10^{-5}	0.00	3.22 × 10^{4}	7.30 × 10^{-4}	2.27 × 10^{-8}
-3.38	6.88 × 10^{-5}	-0.05	3.73 × 10^{4}	4.93 × 10^{-4}	1.32 × 10^{-8}
-3.24	4.80 × 10^{-5}	-0.04	5.38 × 10^{4}	9.48 × 10^{-4}	1.76 × 10^{-8}
-3.07	2.10 × 10^{-5}	0.18	1.34 × 10^{5}	2.44 × 10^{-3}	1.83 × 10^{-8}
-2.88	6.21 × 10^{-6}	0.51	5.14 × 10^{5}	9.01 × 10^{-3}	1.75 × 10^{-8}
-2.71	8.65 × 10^{-7}	0.75	4.05 × 10^{6}	7.83 × 10^{-2}	1.93 × 10^{-8}
-2.52	3.07 × 10^{-7}	0.14	9.02 × 10^{6}	2.03 × 10^{-1}	2.26 × 10^{-8}
-2.29	1.65 × 10^{-7}	-1.65	8.36 × 10^{6}	1.68 × 10^{-1}	2.01 × 10^{-8}
-2.09	3.68 × 10^{-7}	-2.48	2.71 × 10^{6}	5.73 × 10^{-2}	2.11 × 10^{-8}
-1.88	4.98 × 10^{-7}	-2.10	2.32 × 10^{6}	3.65 × 10^{-2}	1.57 × 10^{-8}
-1.72	3.96 × 10^{-7}	-0.91	4.65 × 10^{6}	8.00 × 10^{-2}	1.72 × 10^{-8}
-1.55	8.23 × 10^{-7}	1.42	5.52 × 10^{6}	1.11 × 10^{-1}	2.01 × 10^{-8}
-1.35	5.53 × 10^{-8}	4.62	2.84 × 10^{8}	4.56 × 10^{0}	1.60 × 10^{-8}
-1.19	2.84 × 10^{-7}	7.45	1.66 × 10^{8}	2.37 × 10^{0}	1.43 × 10^{-8}
-1.04	4.17 × 10^{-8}	9.79	2.80 × 10^{9}	4.76 × 10^{1}	1.70 × 10^{-8}
-0.87	5.11 × 10^{-7}	12.87	7.54 × 10^{8}	1.58 × 10^{1}	2.09 × 10^{-8}
-0.67	6.54 × 10^{-10}	16.17	2.12 × 10^{12}	4.40 × 10^{4}	2.07 × 10^{-8}
-0.46	1.56 × 10^{-6}	17.42	1.45 × 10^{9}	2.93 × 10^{1}	2.02 × 10^{-8}
-0.26	1.06 × 10^{-6}	18.08	2.74 × 10^{9}	5.38 × 10^{1}	1.96 × 10^{-8}
-0.06	1.91 × 10^{-6}	18.32	1.68 × 10^{9}	2.64 × 10^{1}	1.57 × 10^{-8}
0.10	1.87 × 10^{-6}	18.03	1.53 × 10^{9}	6.10 × 10^{1}	3.98 × 10^{-8}
0.50	7.09 × 10^{-7}	16.91	2.61 × 10^{9}	5.75 × 10^{1}	2.20 × 10^{-8}
0.72	1.03 × 10^{-7}	15.59	1.08 × 10^{10}	1.84 × 10^{2}	1.71 × 10^{-8}
0.89	1.78 × 10^{-7}	13.28	2.55 × 10^{9}	6.61 × 10^{1}	2.59 × 10^{-8}
1.15	3.88 × 10^{-7}	8.63	1.92 × 10^{8}	3.23 × 10^{0}	1.68 × 10^{-8}
1.31	2.34 × 10^{-7}	5.07	8.04 × 10^{7}	1.46 × 10^{0}	1.81 × 10^{-8}
1.50	7.31 × 10^{-7}	1.68	6.86 × 10^{6}	8.48 × 10^{-2}	1.23 × 10^{-8}
1.62	1.12 × 10^{-6}	0.08	2.41 × 10^{6}	4.22 × 10^{-2}	1.75 × 10^{-8}
1.79	2.12 × 10^{-7}	-1.70	6.38 × 10^{6}	1.33 × 10^{-1}	2.08 × 10^{-8}
2.00	2.07 × 10^{-7}	-2.35	5.09 × 10^{6}	9.79 × 10^{-2}	1.92 × 10^{-8}
2.19	4.37 × 10^{-7}	-1.31	3.60 × 10^{6}	8.72 × 10^{-2}	2.42 × 10^{-8}
2.44	2.07 × 10^{-7}	0.99	1.86 × 10^{7}	3.49 × 10^{-1}	1.88 × 10^{-8}
2.62	2.87 × 10^{-7}	2.54	2.45 × 10^{7}	4.71 × 10^{-1}	1.93 × 10^{-8}
2.82	3.64 × 10^{-6}	2.96	2.27 × 10^{6}	4.81 × 10^{-2}	2.12 × 10^{-8}
3.03	1.91 × 10^{-5}	2.50	3.63 × 10^{5}	5.42 × 10^{-3}	1.50 × 10^{-8}
3.18	3.38 × 10^{-5}	2.27	1.87 × 10^{5}	2.58 × 10^{-3}	1.38 × 10^{-8}
3.32	5.83 × 10^{-5}	2.18	1.05 × 10^{5}	1.69 × 10^{-3}	1.61 × 10^{-8}
3.48	7.78 × 10^{-5}	2.20	7.91 × 10^{4}	1.52 × 10^{-3}	1.93 × 10^{-8}
3.67	8.36 × 10^{-5}	2.19	7.35 × 10^{4}	1.41 × 10^{-3}	1.93 × 10^{-8}
			1/P = $\sum \frac{\exp(\Delta G(z)/RT)}{D(z)}\Delta z$ (cm^{-1} s)	4.45 x 10^{4}	
			P (cm s^{-1}) =	2.25 x 10^{-5}	

Table S4. Green-Kubo components (Equation S1) for temozolomide per window: (1) average z value of window, (2) D(z) (cm^{2}s^{-1}) from Green-Kubo relation using the converged part of the force (350 to 500 ns) and 5 x 10^4 lags. D(z) is calculated by solution of the fit to a general exponential function (Fitzgerald et al., 2023). G(z) (kJ mol^{-1}) from integration of force profile, ISD equation S1 component $\exp(\Delta G(z)/RT)/D(z)$ (cm^{-2} s), ISD equation S1 component $\Delta z * \exp(\Delta G(z)/RT)/D(z)$ (cm^{-1} s), Δz (cm).

Temozolomide z (nm)	D(z) (cm^{2}s^{-1})	G(z) (kJ mol^{-1})	$\frac{\exp(\Delta G(z)/RT)}{D(z)}$ cm^{-2} s	$\frac{\exp(\Delta G(z)/RT)}{D(z)}\Delta z$ cm^{-1} s	Δz (cm)
-3.65	9.14×10^{-5}	0	4.25×10^{5}	8.79×10^{-3}	2.07×10^{-8}
-3.44	8.76×10^{-5}	-0.09	4.28×10^{5}	8.19×10^{-3}	1.91×10^{-8}
-3.25	5.91×10^{-5}	-0.39	5.65×10^{5}	1.25×10^{-2}	2.21×10^{-8}
-3.03	1.78×10^{-5}	-0.94	1.51×10^{6}	2.68×10^{-2}	1.77×10^{-8}
-2.85	7.31×10^{-6}	-1.24	3.28×10^{6}	6.93×10^{-2}	2.11×10^{-8}
-2.64	1.82×10^{-6}	-1.81	1.06×10^{7}	2.12×10^{-1}	2.01×10^{-8}
-2.44	1.27×10^{-6}	-3.23	8.74×10^{6}	1.52×10^{-1}	1.74×10^{-8}
-2.26	4.51×10^{-7}	-5.2	1.15×10^{7}	1.83×10^{-1}	1.60×10^{-8}
-2.10	1.42×10^{-8}	-7.63	1.42×10^{8}	3.15×10^{0}	2.22×10^{-8}
-1.88	4.24×10^{-7}	-9.43	2.36×10^{6}	4.43×10^{-2}	1.88×10^{-8}
-1.69	1.53×10^{-6}	-8.31	1.01×10^{6}	1.99×10^{-2}	1.98×10^{-8}
-1.50	2.48×10^{-7}	-5.04	2.21×10^{7}	3.89×10^{-1}	1.76×10^{-8}
-1.32	2.01×10^{-7}	-0.49	1.60×10^{8}	2.90×10^{0}	1.82×10^{-8}
-1.14	2.78×10^{-7}	5.14	1.03×10^{9}	2.33×10^{1}	2.27×10^{-8}
-0.91	4.93×10^{-7}	13.03	1.24×10^{10}	1.35×10^{2}	1.09×10^{-8}
-0.80	8.54×10^{-9}	17.3	3.74×10^{12}	7.02×10^{4}	1.88×10^{-8}
-0.61	4.19×10^{-7}	21.86	4.47×10^{11}	8.91×10^{3}	1.99×10^{-8}
-0.41	9.34×10^{-7}	22.45	2.52×10^{11}	5.02×10^{3}	1.99×10^{-8}
-0.22	2.01×10^{-6}	22.43	1.16×10^{11}	1.56×10^{3}	1.34×10^{-8}
-0.08	2.89×10^{-6}	22.6	8.63×10^{10}	1.79×10^{3}	2.08×10^{-8}
0.13	1.62×10^{-6}	22.43	1.44×10^{11}	3.14×10^{3}	2.18×10^{-8}
0.34	1.64×10^{-6}	22.06	1.23×10^{11}	3.05×10^{3}	2.47×10^{-8}
0.59	7.83×10^{-7}	21.22	1.87×10^{11}	3.92×10^{3}	2.10×10^{-8}
0.80	4.23×10^{-7}	16.62	5.79×10^{10}	1.19×10^{3}	2.05×10^{-8}
1.01	9.25×10^{-8}	9.46	1.65×10^{10}	4.20×10^{2}	2.55×10^{-8}
1.26	4.31×10^{-7}	3.18	3.10×10^{8}	5.04×10^{0}	1.63×10^{-8}
1.42	2.81×10^{-7}	0.63	1.77×10^{8}	3.72×10^{0}	2.10×10^{-8}
1.63	9.86×10^{-8}	-2.18	1.69×10^{8}	3.29×10^{0}	1.95×10^{-8}
1.83	6.46×10^{-7}	-4.75	9.51×10^{6}	1.71×10^{-1}	1.80×10^{-8}
2.01	9.95×10^{-7}	-5.54	4.55×10^{6}	9.55×10^{-2}	2.10×10^{-8}
2.22	3.68×10^{-7}	-3.34	2.88×10^{7}	6.91×10^{-1}	2.40×10^{-8}
2.46	7.84×10^{-7}	0.42	5.83×10^{7}	1.33×10^{0}	2.28×10^{-8}
2.69	8.16×10^{-7}	2.21	1.12×10^{8}	1.61×10^{0}	1.44×10^{-8}
2.83	5.86×10^{-6}	2.18	1.54×10^{7}	3.33×10^{-1}	2.16×10^{-8}
3.05	2.13×10^{-5}	2.3	4.44×10^{6}	9.18×10^{-2}	2.07×10^{-8}
3.25	5.87×10^{-5}	2.65	1.85×10^{6}	4.12×10^{-2}	2.23×10^{-8}
3.48	8.82×10^{-5}	2.94	1.38×10^{6}	2.59×10^{-2}	1.88×10^{-8}
3.66	9.11×10^{-5}	3.05	1.39×10^{6}	3.02×10^{-2}	2.17×10^{-8}
3.88	9.17×10^{-5}	3.06	1.39×10^{6}	2.43×10^{-2}	1.75×10^{-8}
4.06	9.18×10^{-5}	3.08	1.40×10^{6}	2.45×10^{-2}	1.75×10^{-8}
			1/P = $\sum \frac{\exp(\Delta G(z)/RT)}{D(z)}\Delta z$ (cm^{-1} s)	9.94×10^{4}	
			P (cm s^{-1}) =	1.01×10^{-5}	

Table S5. Gamma function fitting coefficients (*a, b, c*) components obtained by solving the fit of the ACF onto the general exponential in Equation S5 for ethanol per window: (1) average *z* value of window, fitting coefficients (*a, b, c*), and resulting diffusivity $D(z)$ ($cm^2\,s^{-1}$) obtained from the Green-Kubo equation.

Ethanol fitting coefficients *a, b, c*					
Window	Ethanol z (nm)	*a*	*b*	*c*	D(z) (cm^2s^{-1})
1	-3.48	1.28×10^4	1.97×10^9	4.90	2.10×10^{-4}
2	-3.29	1.28×10^4	1.25×10^9	5.00	2.10×10^{-4}
3	-3.12	1.28×10^4	5.24×10^8	5.21	2.00×10^{-4}
4	-2.90	1.30×10^4	41.3	2.91	4.80×10^{-5}
5	-2.73	1.29×10^4	24.0	3.39	2.50×10^{-5}
6	-2.57	1.35×10^4	8.86	5.54	2.80×10^{-6}
7	-2.35	1.30×10^4	8.42	5.33	2.10×10^{-6}
8	-2.23	1.28×10^4	9.84	4.81	3.60×10^{-6}
9	-2.04	1.30×10^4	6.04	6.67	3.20×10^{-7}
10	-1.82	1.30×10^4	7.43	5.88	1.20×10^{-6}
11	-1.62	1.30×10^4	7.46	5.77	1.20×10^{-6}
12	-1.46	1.32×10^4	6.54	6.92	5.20×10^{-7}
13	-1.23	1.21×10^4	6.50	5.55	5.60×10^{-7}
14	-1.03	1.27×10^4	5.97	6.98	2.80×10^{-7}
15	-0.88	1.29×10^4	5.68	9.21	7.80×10^{-8}
16	-0.69	1.26×10^4	6.51	7.25	5.00×10^{-7}
17	-0.49	1.27×10^4	8.18	6.69	2.50×10^{-6}
18	-0.26	1.26×10^4	9.75	6.09	6.50×10^{-6}
19	-0.06	1.27×10^4	9.93	6.13	7.30×10^{-6}
20	0.13	1.26×10^4	10.2	6.02	8.20×10^{-6}
21	0.32	1.28×10^4	8.64	6.70	3.50×10^{-6}
22	0.71	1.27×10^4	8.70	6.30	3.40×10^{-6}
23	0.90	1.27×10^4	4.90	8.68	2.90×10^{-8}
24	1.09	1.22×10^4	7.09	5.82	9.50×10^{-7}
25	1.32	1.21×10^4	6.84	5.35	7.30×10^{-7}
26	1.52	1.29×10^4	7.18	5.86	9.70×10^{-7}
27	1.69	1.30×10^4	6.70	6.28	6.40×10^{-7}
28	1.89	1.29×10^4	7.24	5.94	1.00×10^{-6}
29	2.06	1.33×10^4	5.84	7.37	2.10×10^{-7}
30	2.21	1.29×10^4	9.06	5.02	2.70×10^{-6}
31	2.41	1.31×10^4	10.0	4.91	4.10×10^{-6}
32	2.65	1.30×10^4	17.2	3.80	1.40×10^{-5}
33	2.86	1.30×10^4	28.6	3.25	3.30×10^{-5}
34	3.06	1.30×10^4	138	2.17	9.60×10^{-5}
35	3.27	1.28×10^4	1.04×10^9	5.04	2.10×10^{-4}
36	3.46	1.28×10^4	1.79×10^9	4.92	2.10×10^{-4}
37	3.66	1.28×10^4	2.38×10^9	4.85	2.10×10^{-4}
38	3.89	1.28×10^4	2.29×10^9	4.86	2.10×10^{-4}
39	4.10	1.28×10^4	2.38×10^9	4.85	2.10×10^{-4}

Table S6. Gamma function fitting coefficients (*a, b, c*) components obtained by solving the fit of the ACF onto the general exponential in Equation S5 for caffeine per window: (1) average *z* value of window, fitting coefficients (*a, b, c*), and resulting diffusivity *D*(z) ($cm^2\,s^{-1}$) obtained from the Green-Kubo equation.

Caffeine fitting coefficients *a, b, c*					
Window	Caffeine z (nm)	*a*	*b*	*c*	*D*(z) (cm^2s^{-1})
1	-3.60	1.31×10^4	5.37×10^5	7.27	8.14×10^{-5}
2	-3.38	1.35×10^4	1.09×10^4	1.03	6.88×10^{-5}
3	-3.24	1.37×10^4	454	1.56	4.80×10^{-5}
4	-3.07	1.38×10^4	49.3	2.44	2.10×10^{-5}
5	-2.88	1.41×10^4	15.9	3.46	6.21×10^{-6}
6	-2.71	1.45×10^4	7.41	5.05	8.65×10^{-7}
7	-2.52	1.48×10^4	6.04	5.66	3.07×10^{-7}
8	-2.29	1.31×10^4	5.3	5.67	1.65×10^{-7}
9	-2.09	1.48×10^4	6.23	5.8	3.68×10^{-7}
10	-1.88	1.32×10^4	6.57	4.83	4.98×10^{-7}
11	-1.72	1.4×10^4	6.27	5.36	3.96×10^{-7}
12	-1.55	1.27×10^4	7.27	4.77	8.23×10^{-7}
13	-1.35	1.44×10^4	4.87	7.14	5.53×10^{-8}
14	-1.19	1.32×10^4	5.84	5.78	2.84×10^{-7}
15	-1.04	1.41×10^4	4.73	7.36	4.17×10^{-8}
16	-0.87	1.27×10^4	6.43	5.79	5.11×10^{-7}
17	-0.67	1.38×10^4	4.24	1.14	6.54×10^{-10}
18	-0.46	1.28×10^4	8	5.27	1.56×10^{-6}
19	-0.26	1.33×10^4	7.4	5.57	1.06×10^{-6}
20	-0.06	1.34×10^4	8.46	5.15	1.91×10^{-6}
21	0.10	1.32×10^4	8.46	5.06	1.87×10^{-6}
22	0.50	1.31×10^4	6.83	5.83	7.09×10^{-7}
23	0.72	1.35×10^4	5.39	7.62	1.03×10^{-7}
24	0.89	1.36×10^4	5.52	6.43	1.78×10^{-7}
25	1.15	1.4×10^4	6.24	5.9	3.88×10^{-7}
26	1.31	1.47×10^4	5.82	6.28	2.34×10^{-7}
27	1.50	1.29×10^4	7.03	5	7.31×10^{-7}
28	1.62	1.16×10^4	8.24	4.03	1.12×10^{-6}
29	1.79	1.49×10^4	5.69	5.9	2.12×10^{-7}
30	2.00	1.38×10^4	5.6	5.97	2.07×10^{-7}
31	2.19	1.27×10^4	6.36	4.82	4.37×10^{-7}
32	2.44	1.45×10^4	5.65	5.98	2.07×10^{-7}
33	2.62	1.47×10^4	5.98	6	2.87×10^{-7}
34	2.82	1.41×10^4	12	3.89	3.64×10^{-6}
35	3.03	1.38×10^4	42.4	2.54	1.91×10^{-5}
36	3.18	1.37×10^4	121	1.99	3.38×10^{-5}
37	3.32	1.36×10^4	1.64×10^3	1.29	5.83×10^{-5}
38	3.48	1.32×10^4	1.08×10^5	8.27	7.78×10^{-5}
39	3.67	1.3×10^4	1.26×10^6	6.84	8.36×10^{-5}

Table S7. Gamma function fitting coefficients (*a, b, c*) components obtained by solving the fit of the ACF onto the general exponential in Equation S5 for temozolomide per window: (1) average *z* value of window, fitting coefficients (*a, b, c*), and resulting diffusivity *D*(z) ($cm^2\ s^{-1}$) obtained from the Green-Kubo equation.

Temozolomide fitting coefficients a, b, c					
Window	Temozolomide z (nm)	*a*	*b*	*c*	*D*(z) (cm^2s^{-1})
1	-3.65	1.28×10^4	4.07×10^7	0.55	9.14×10^{-5}
2	-3.44	1.30×10^4	5.64×10^6	0.62	8.76×10^{-5}
3	-3.25	1.37×10^4	1.54×10^3	1.30	5.91×10^{-5}
4	-3.03	1.38×10^4	38.0	2.63	1.78×10^{-5}
5	-2.85	1.38×10^4	17.6	3.33	7.31×10^{-6}
6	-2.64	1.20×10^4	10.5	3.53	1.82×10^{-6}
7	-2.44	1.29×10^4	8.35	4.35	1.27×10^{-6}
8	-2.26	1.27×10^4	6.34	5.04	4.51×10^{-7}
9	-2.10	1.60×10^4	4.45	8.18	1.42×10^{-8}
10	-1.88	1.38×10^4	6.32	5.63	4.24×10^{-7}
11	-1.69	1.29×10^4	8.74	4.32	1.53×10^{-6}
12	-1.50	1.37×10^4	5.74	5.69	2.48×10^{-7}
13	-1.32	1.38×10^4	5.55	5.85	2.01×10^{-7}
14	-1.14	1.42×10^4	5.90	5.82	2.78×10^{-7}
15	-0.91	1.32×10^4	6.44	5.53	4.93×10^{-7}
16	-0.80	1.38×10^4	4.37	8.88	8.54×10^{-9}
17	-0.61	1.29×10^4	6.25	6.26	4.19×10^{-7}
18	-0.41	1.32×10^4	7.13	6.29	9.34×10^{-7}
19	-0.22	1.31×10^4	8.40	5.32	2.01×10^{-6}
20	-0.08	1.33×10^4	9.36	4.94	2.89×10^{-6}
21	0.13	1.34×10^4	7.98	5.60	1.62×10^{-6}
22	0.34	1.31×10^4	8.01	5.49	1.64×10^{-6}
23	0.59	1.27×10^4	6.90	6.06	7.83×10^{-7}
24	0.80	1.29×10^4	6.25	6.09	4.23×10^{-7}
25	1.01	1.54×10^4	5.22	6.95	9.25×10^{-8}
26	1.26	1.37×10^4	6.33	5.43	4.31×10^{-7}
27	1.42	1.40×10^4	5.89	5.77	2.81×10^{-7}
28	1.63	1.48×10^4	5.18	6.72	9.86×10^{-8}
29	1.83	1.01×10^4	6.88	4.14	6.46×10^{-7}
30	2.01	1.35×10^4	7.65	4.78	9.95×10^{-7}
31	2.22	1.38×10^4	6.16	5.47	3.68×10^{-7}
32	2.46	1.44×10^4	7.18	5.30	7.84×10^{-7}
33	2.69	1.44×10^4	7.20	5.41	8.16×10^{-7}
34	2.83	1.39×10^4	14.60	3.64	5.86×10^{-6}
35	3.05	1.38×10^4	46.60	2.50	2.13×10^{-5}
36	3.25	1.37×10^4	1.31×10^3	1.33	5.87×10^{-5}
37	3.48	1.30×10^4	8.75×10^6	0.60	8.82×10^{-5}
38	3.66	1.28×10^4	3.61×10^7	0.56	9.11×10^{-5}
39	3.88	1.28×10^4	5.36×10^7	0.54	9.17×10^{-5}
40	4.06	1.28×10^4	5.45×10^7	0.54	9.18×10^{-5}

Force timeseries raw data

Force time series for ethanol:

pull_prod_z_0_pullf.xvg

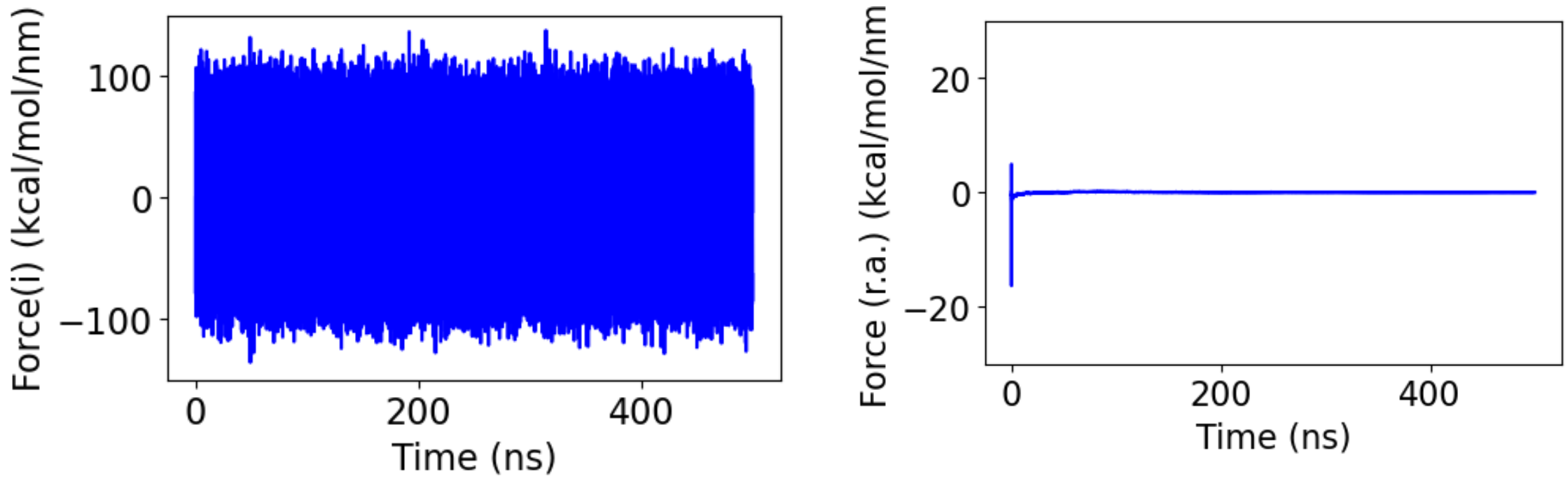

pull_prod_z_1_pullf.xvg

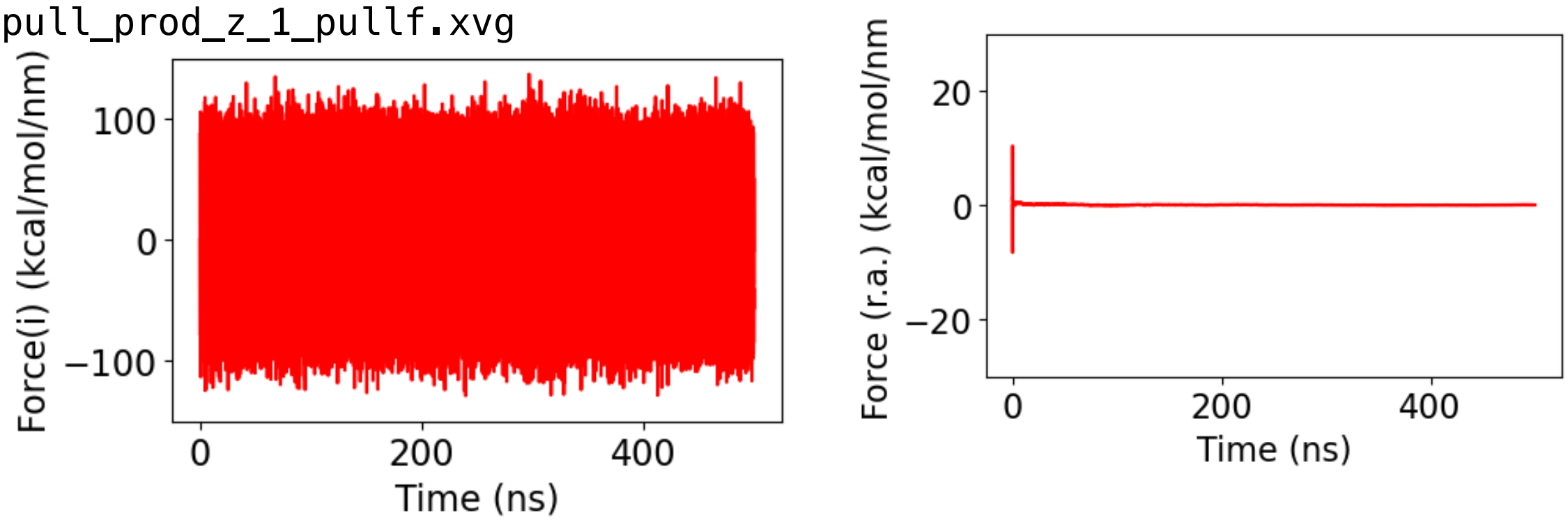

pull_prod_z_2_pullf.xvg

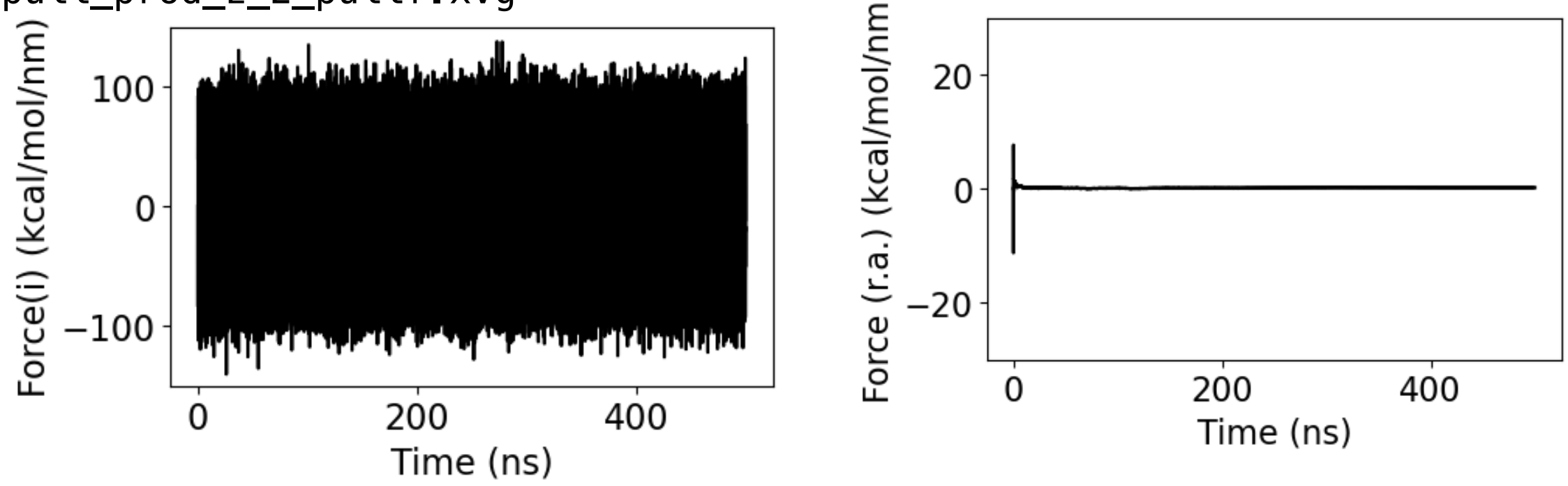

pull_prod_z_3_pullf.xvg

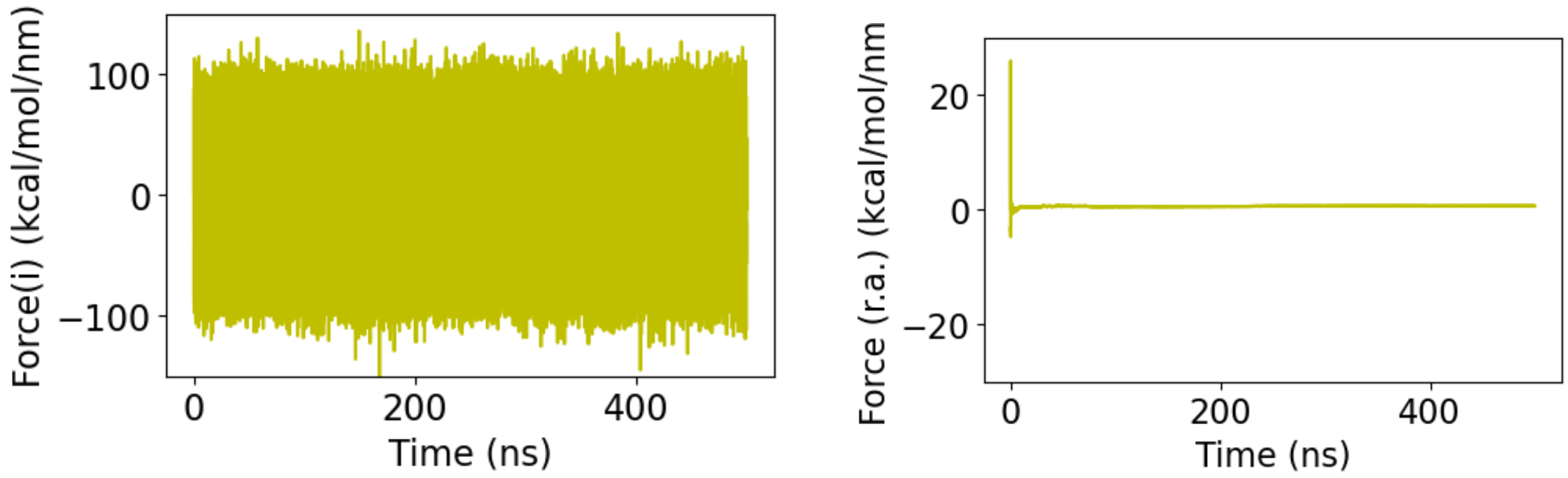

pull_prod_z_4_pullf.xvg

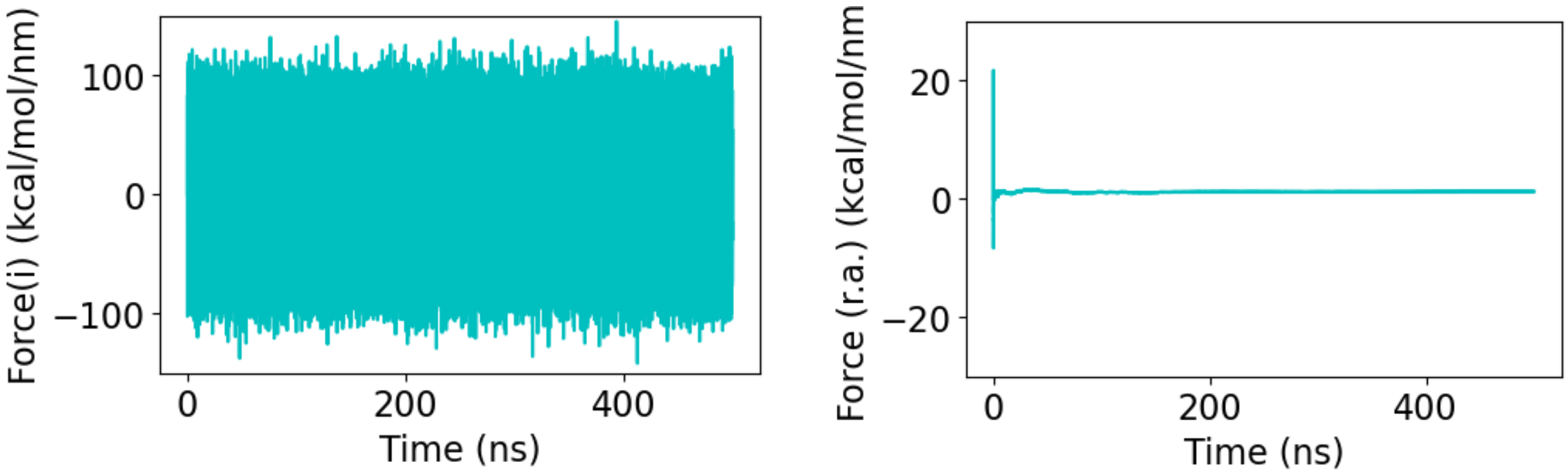

pull_prod_z_5_pullf.xvg

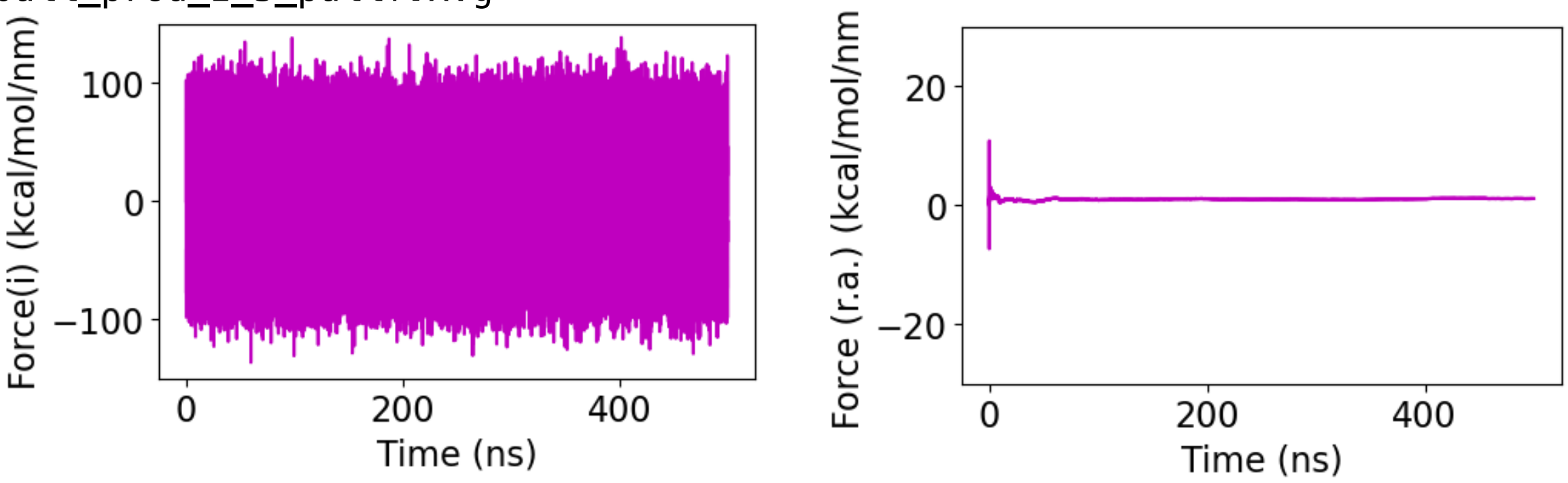

pull_prod_z_6_pullf.xvg

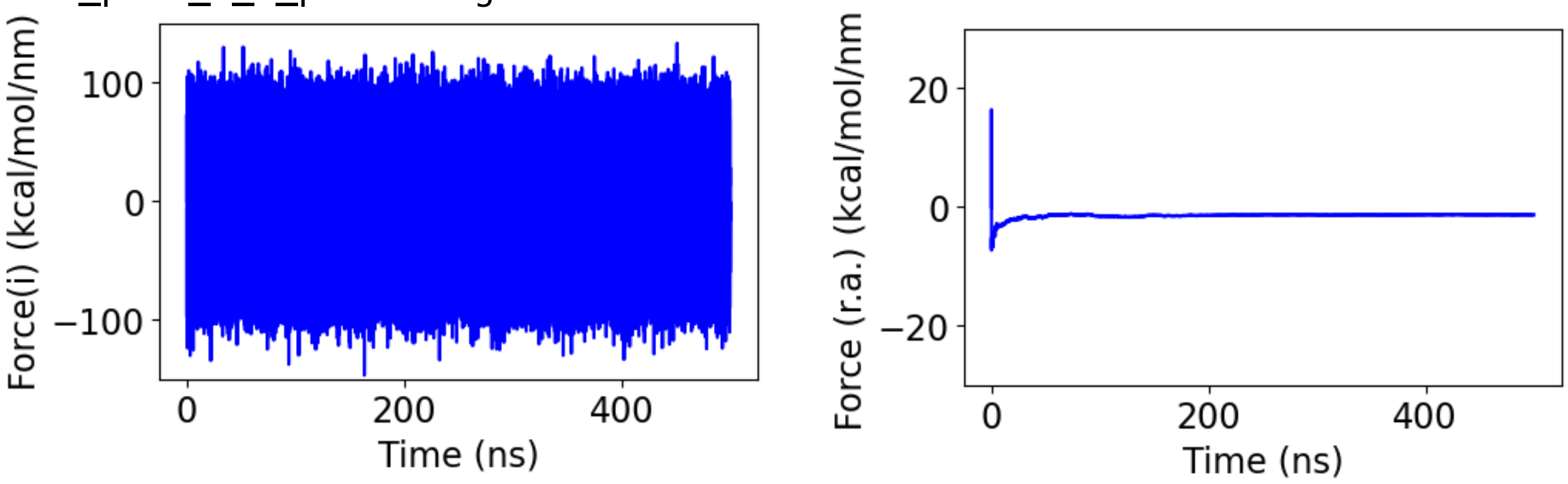

pull_prod_z_7_pullf.xvg

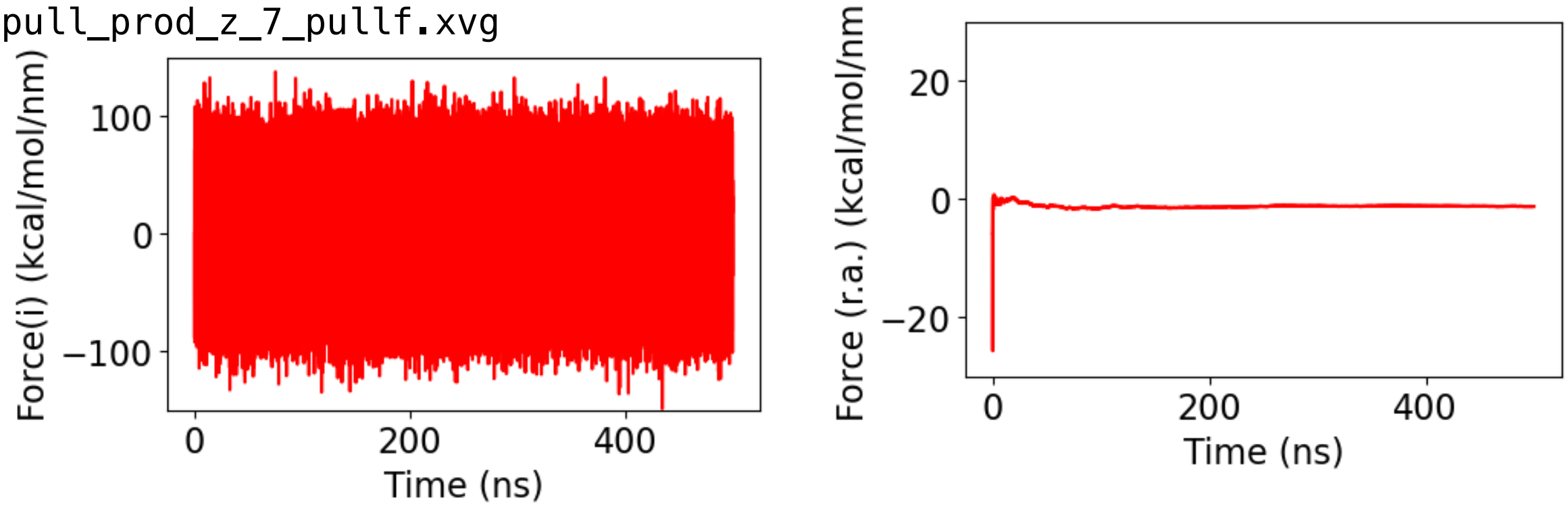

pull_prod_z_8_pullf.xvg

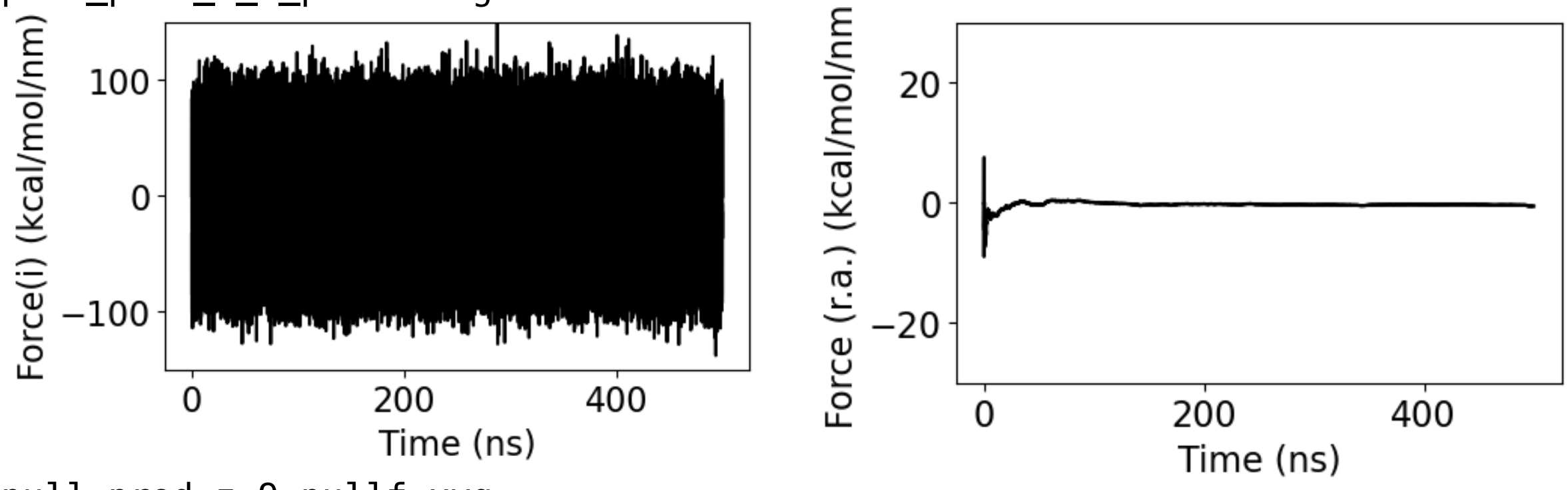

pull_prod_z_9_pullf.xvg

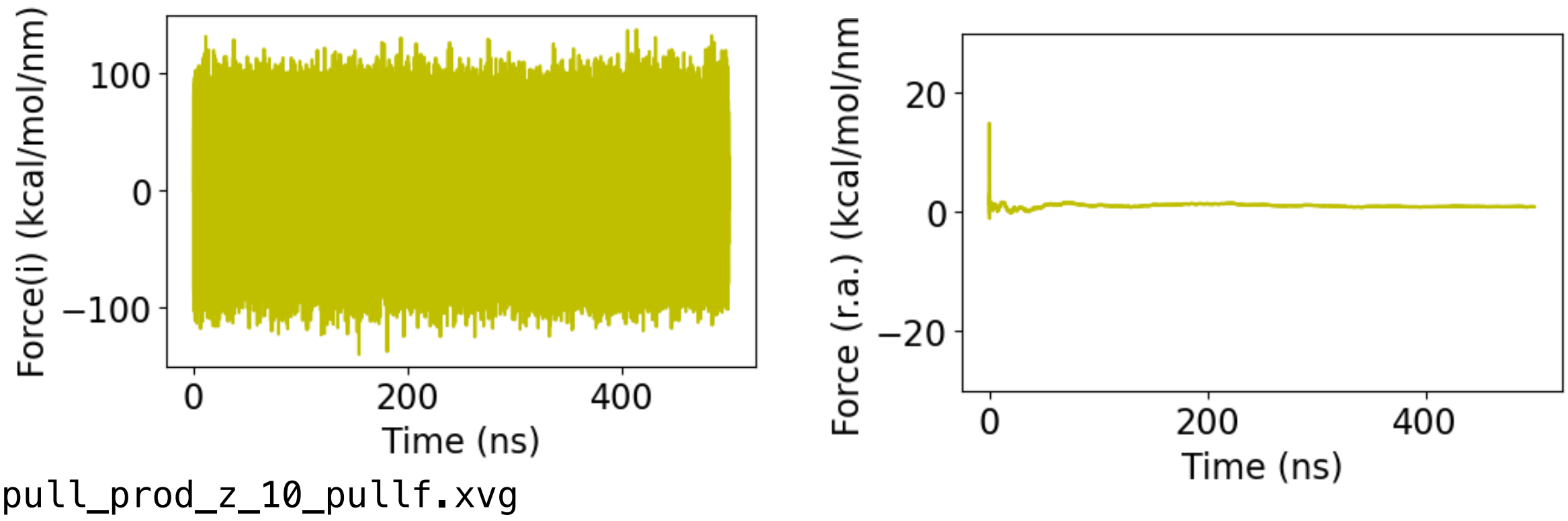

pull_prod_z_10_pullf.xvg

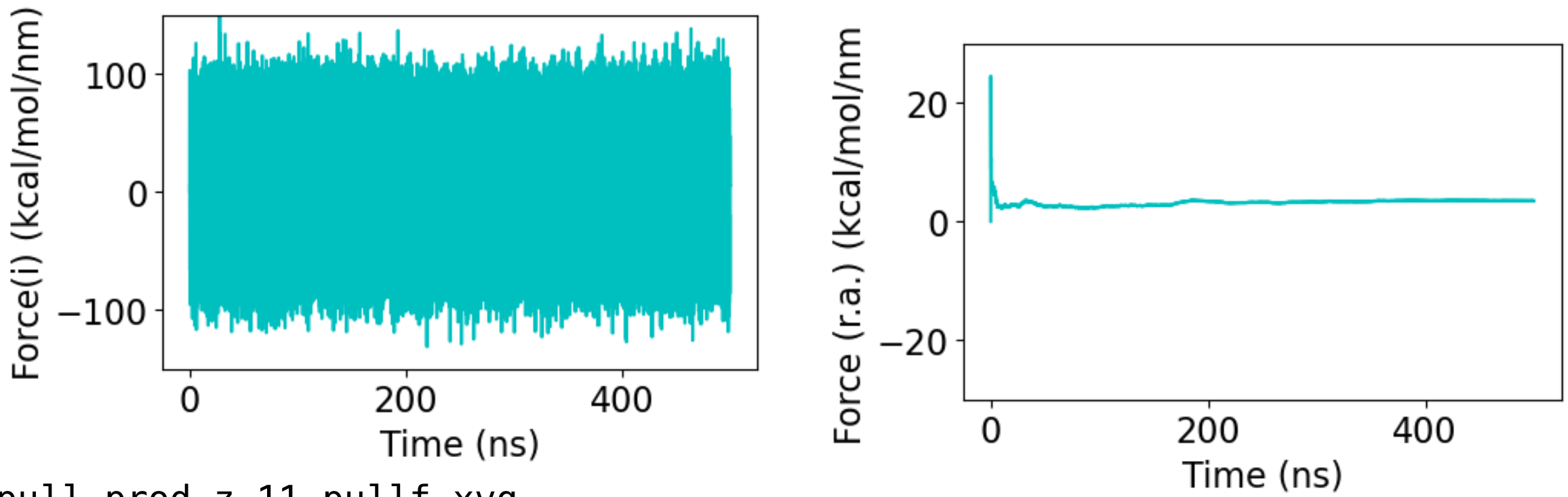

pull_prod_z_11_pullf.xvg

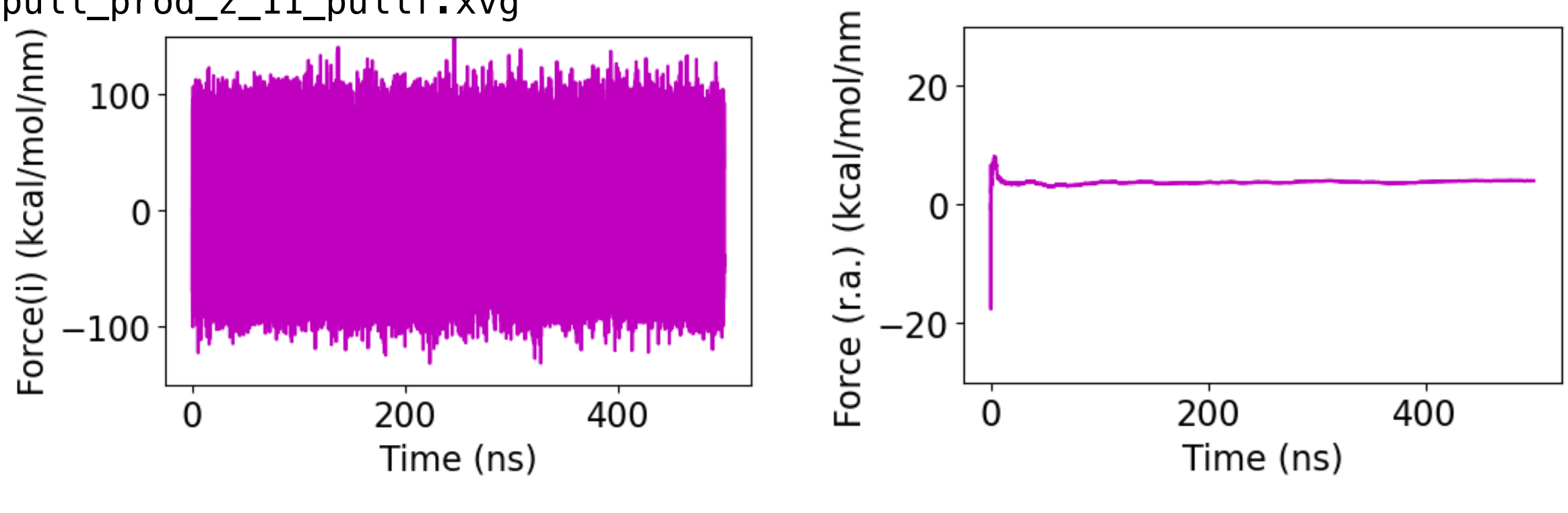

pull_prod_z_12_pullf.xvg

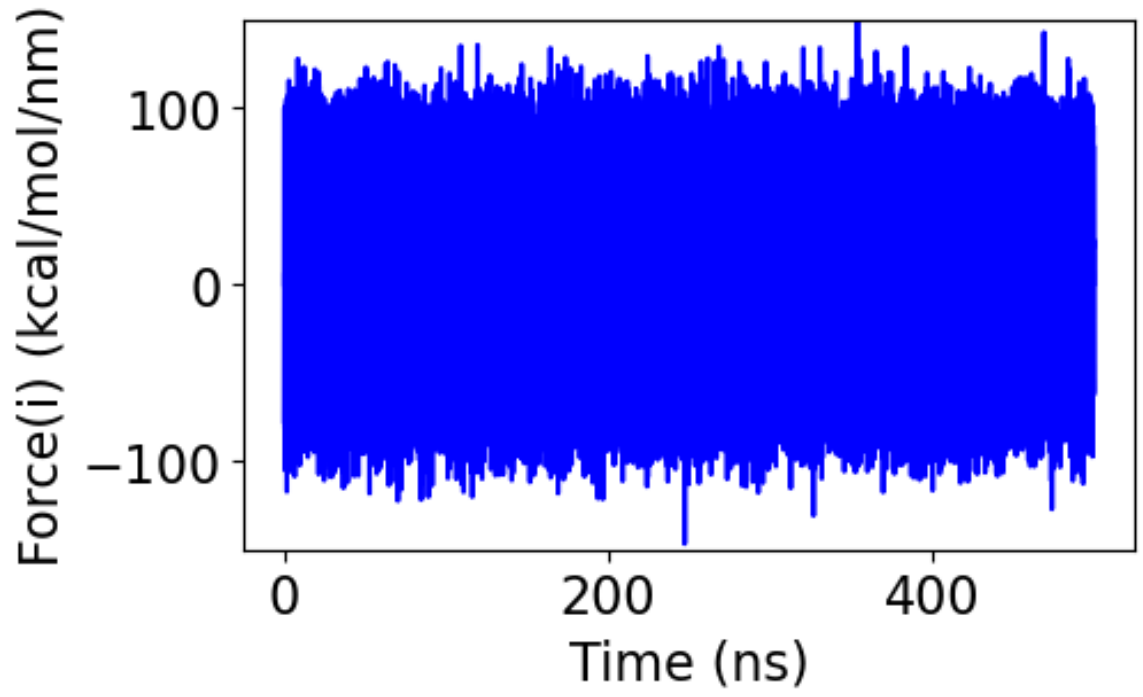

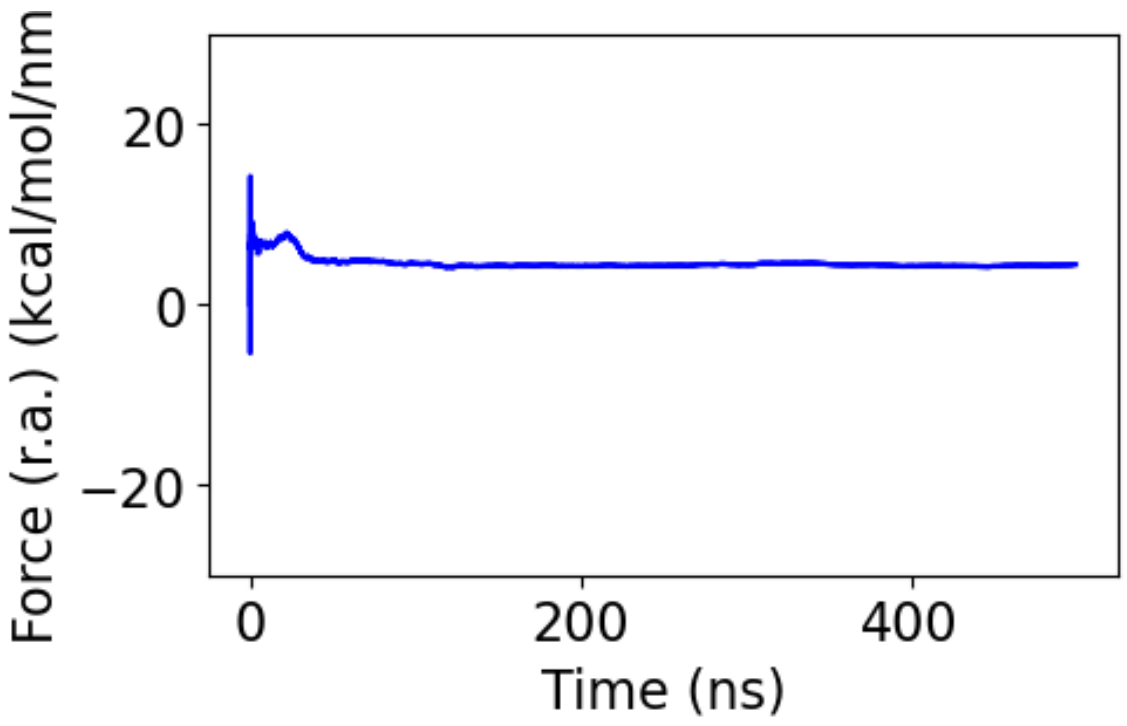

pull_prod_z_13_pullf.xvg

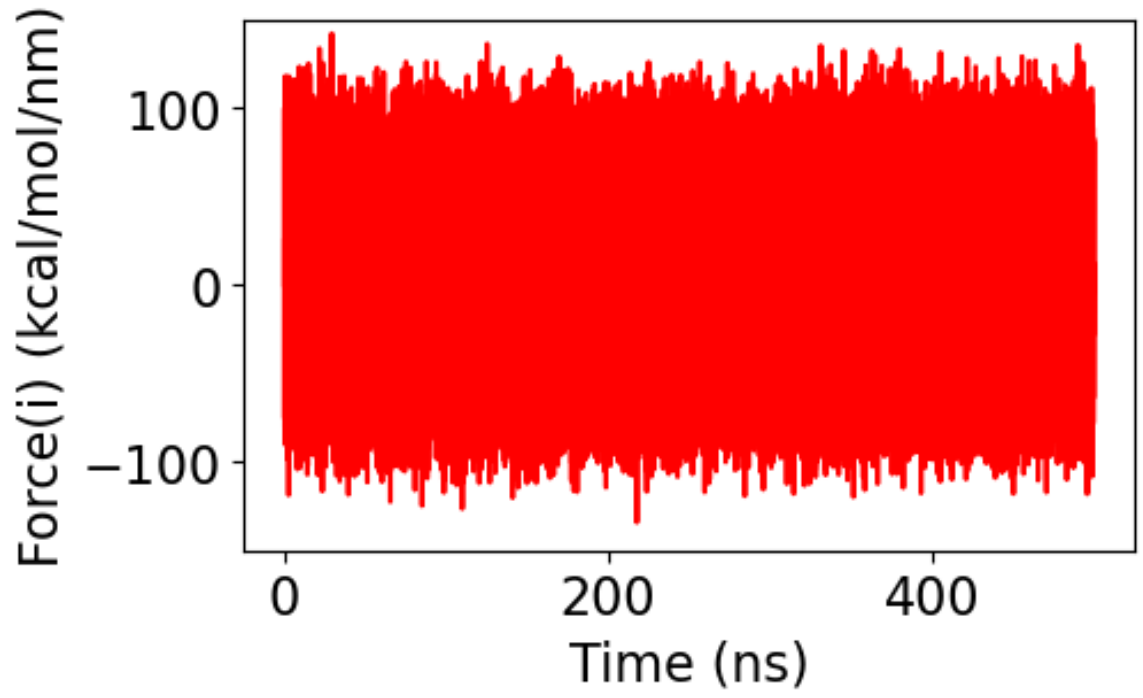

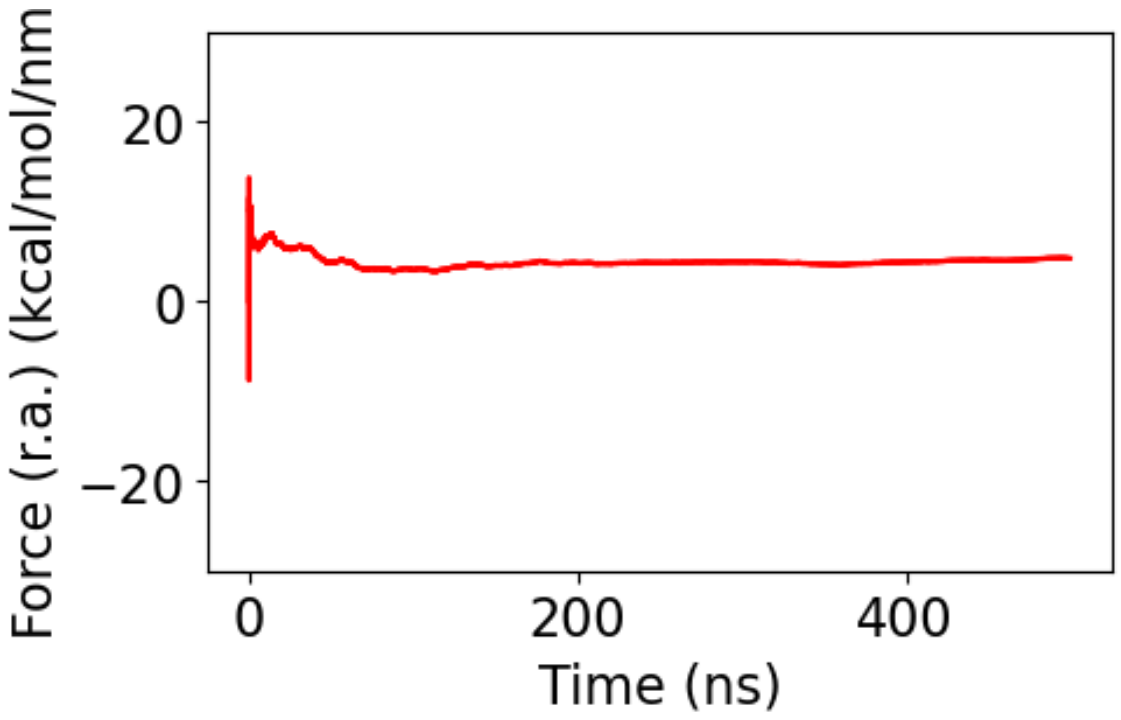

pull_prod_z_14_pullf.xvg

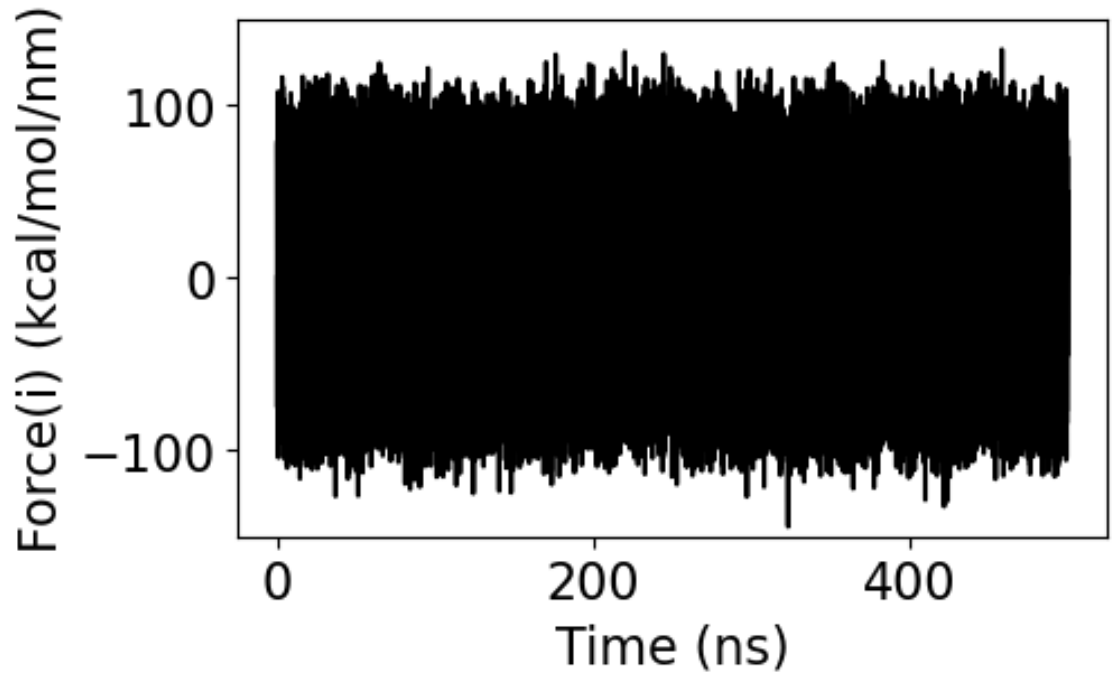

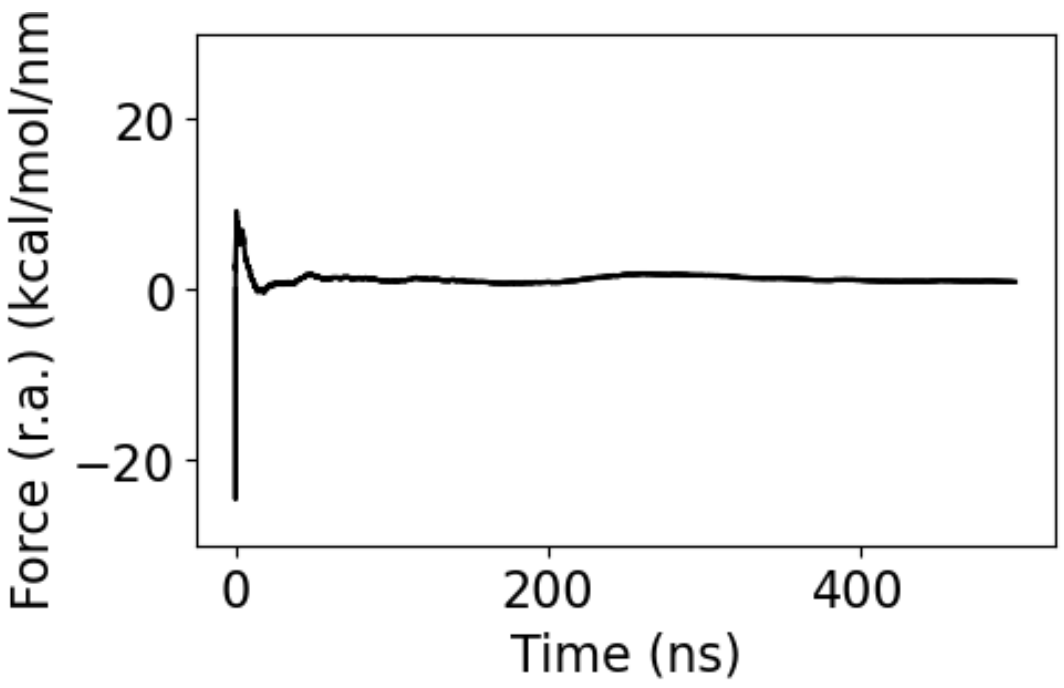

pull_prod_z_15_pullf.xvg

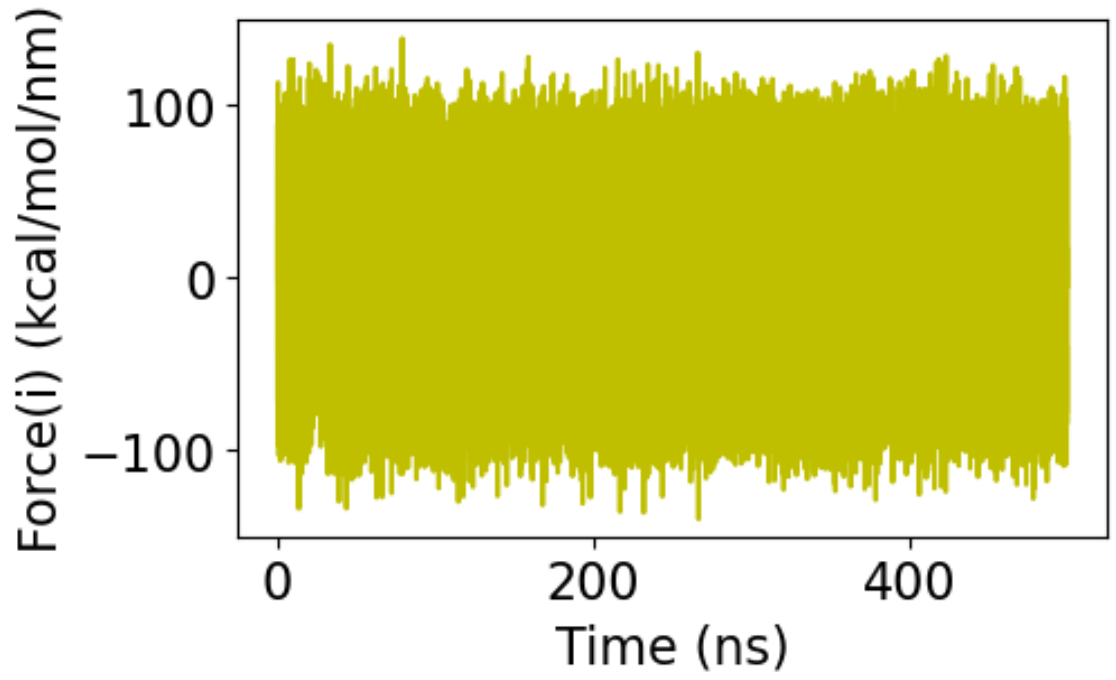

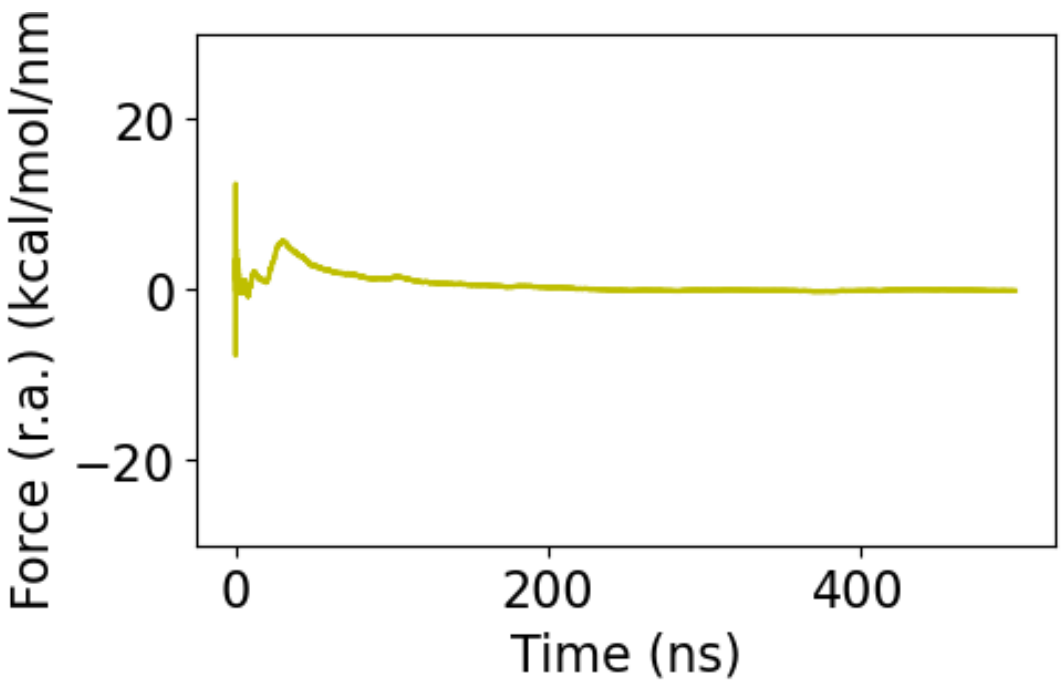

pull_prod_z_16_pullf.xvg

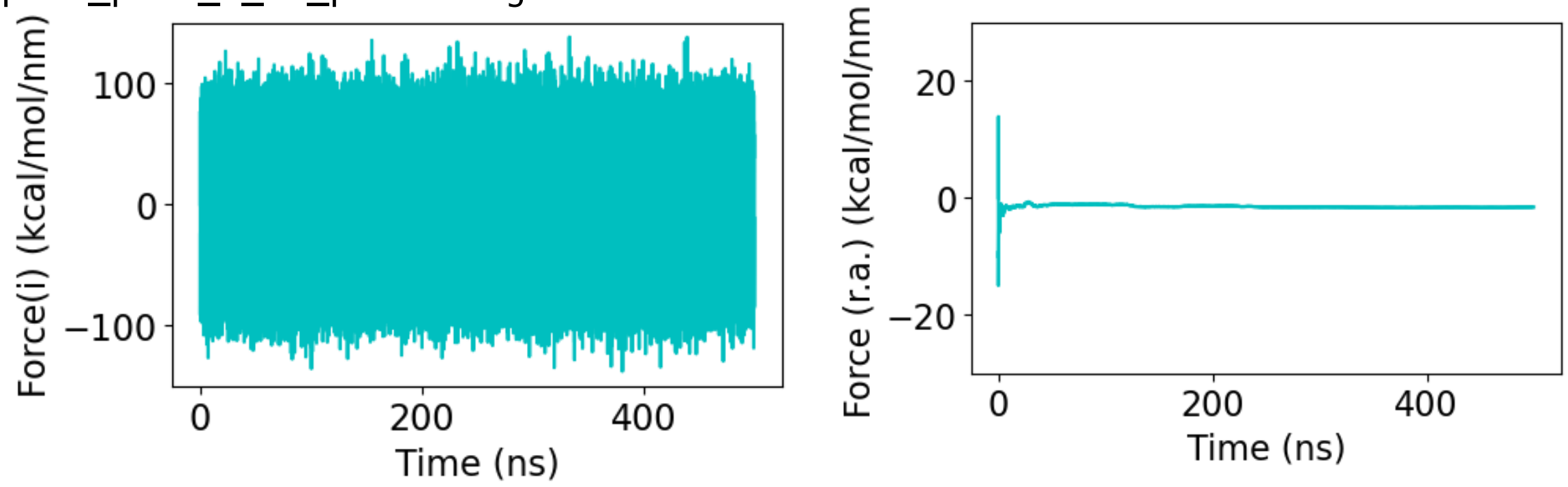

pull_prod_z_17_pullf.xvg

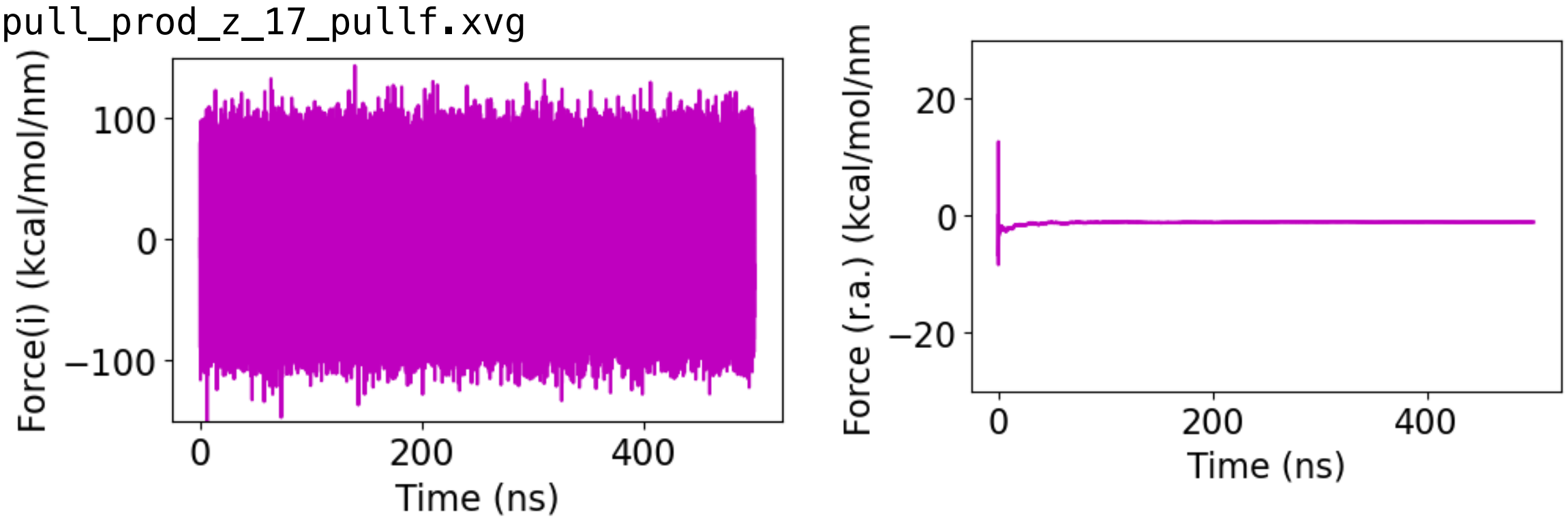

pull_prod_z_18_pullf.xvg

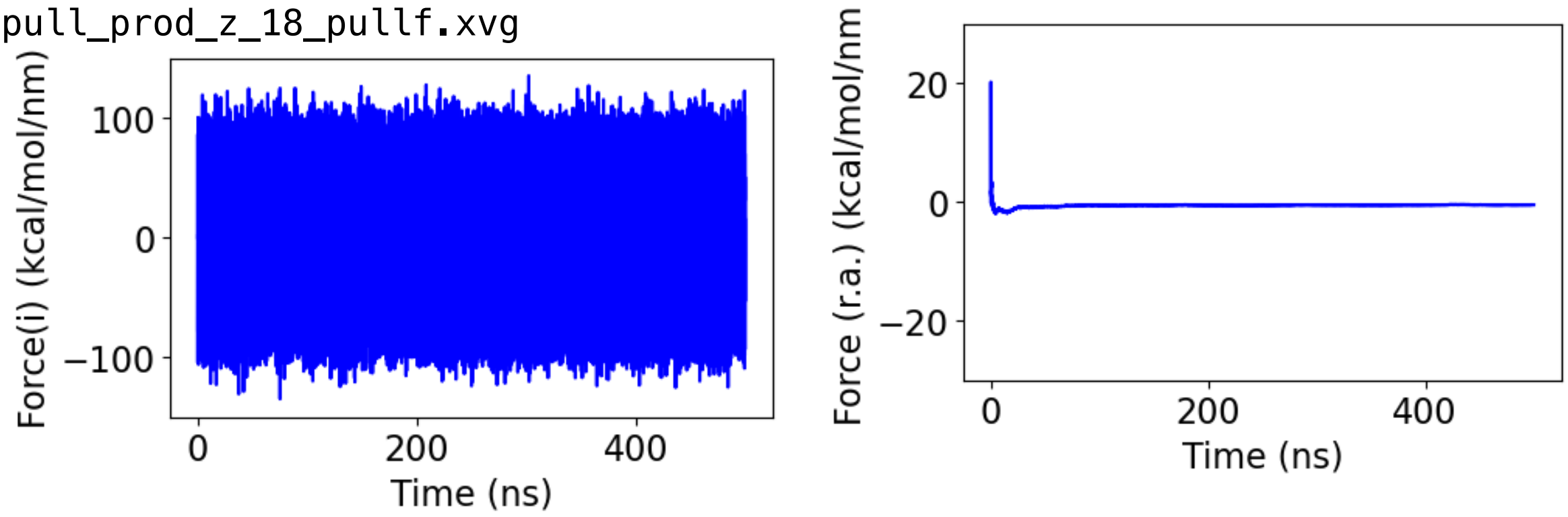

pull_prod_z_19_pullf.xvg

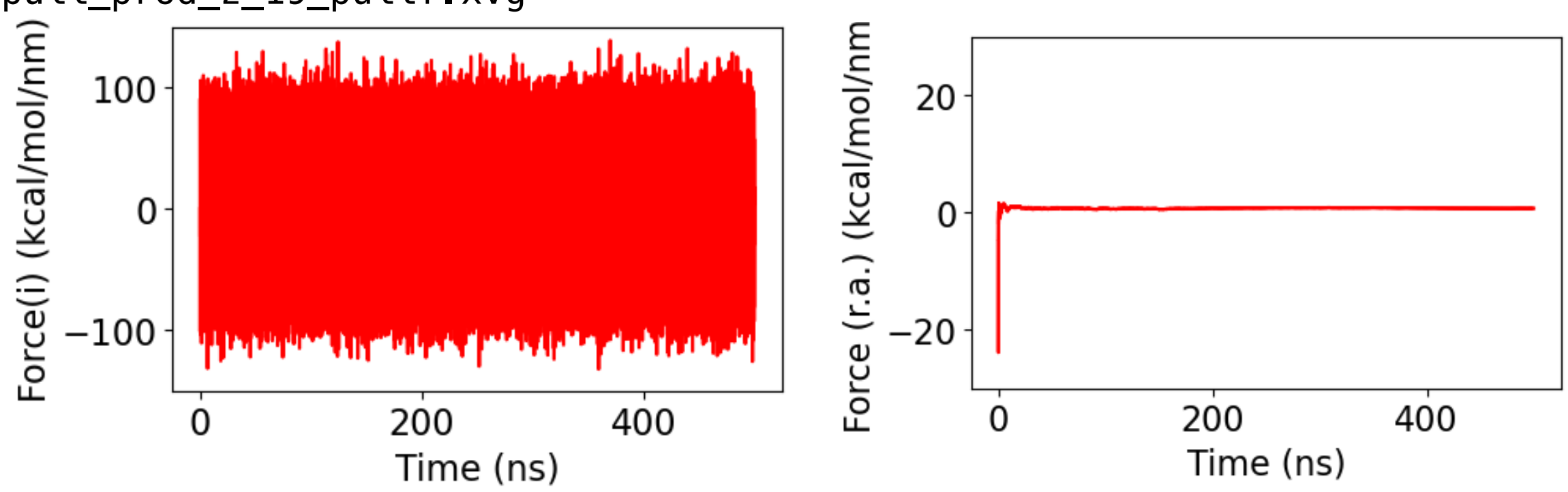

pull_prod_z_20_pullf.xvg

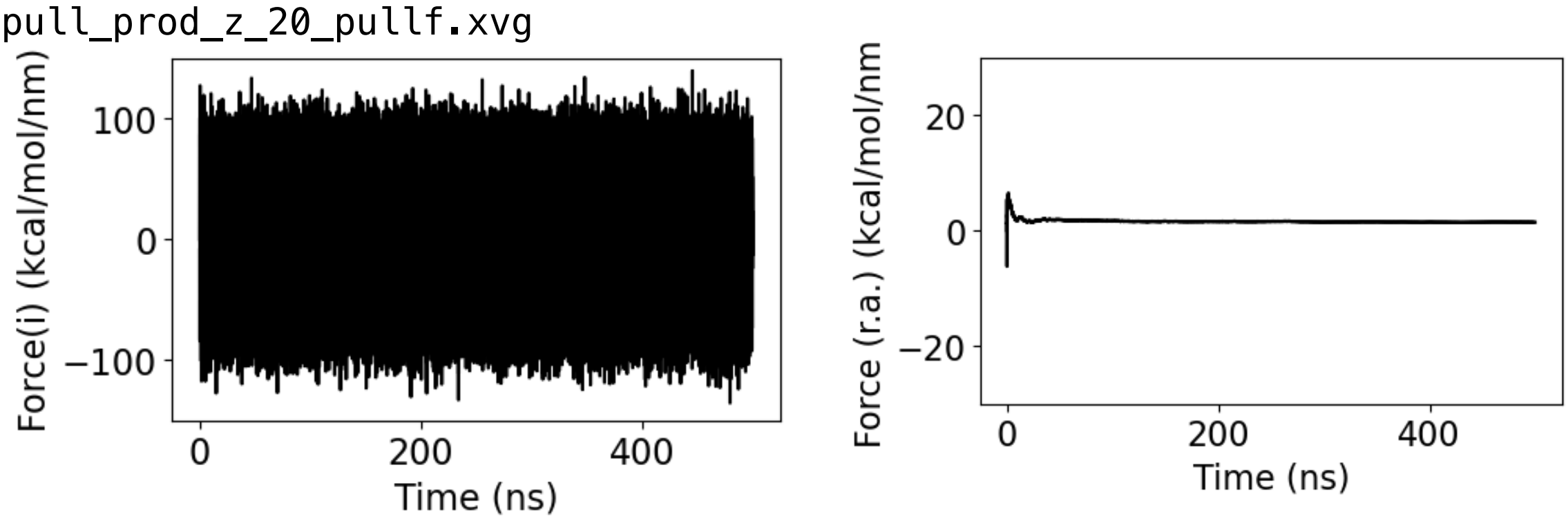

pull_prod_z_21_pullf.xvg

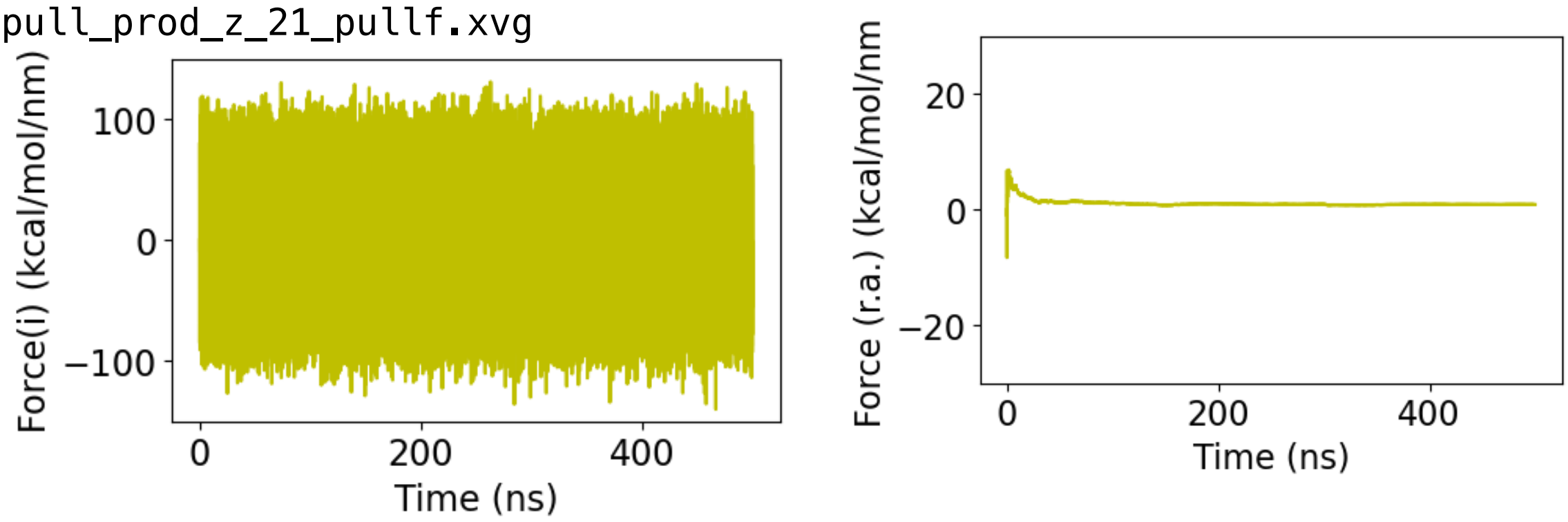

pull_prod_z_23_pullf.xvg

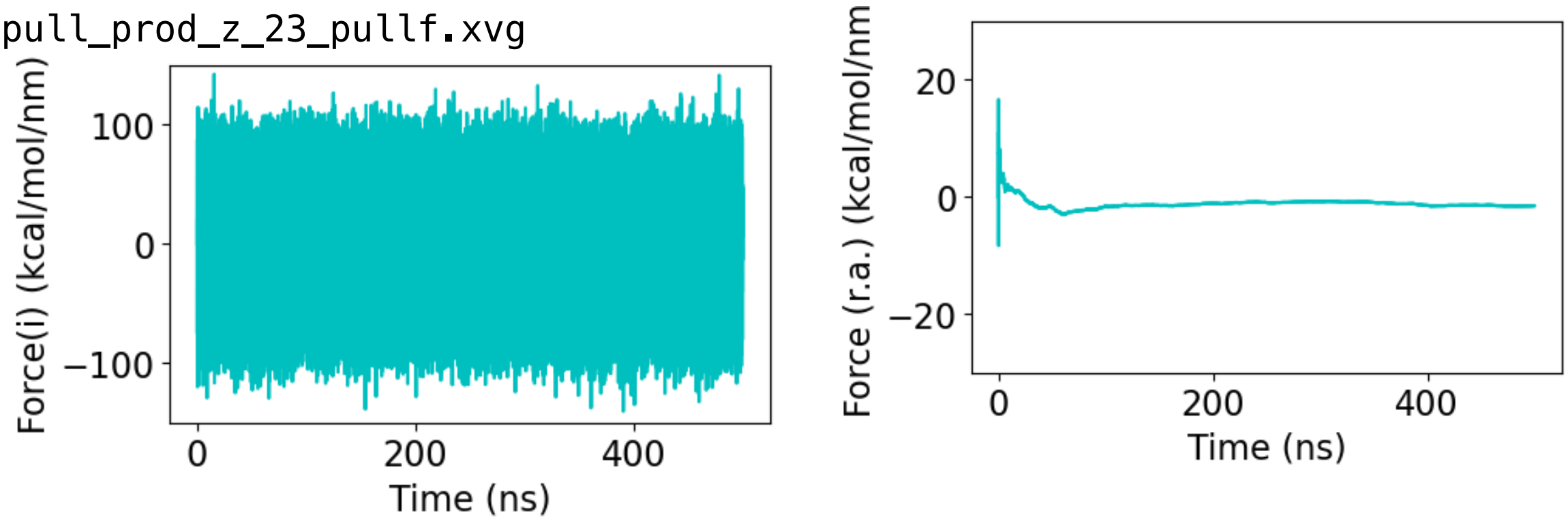

pull_prod_z_24_pullf.xvg

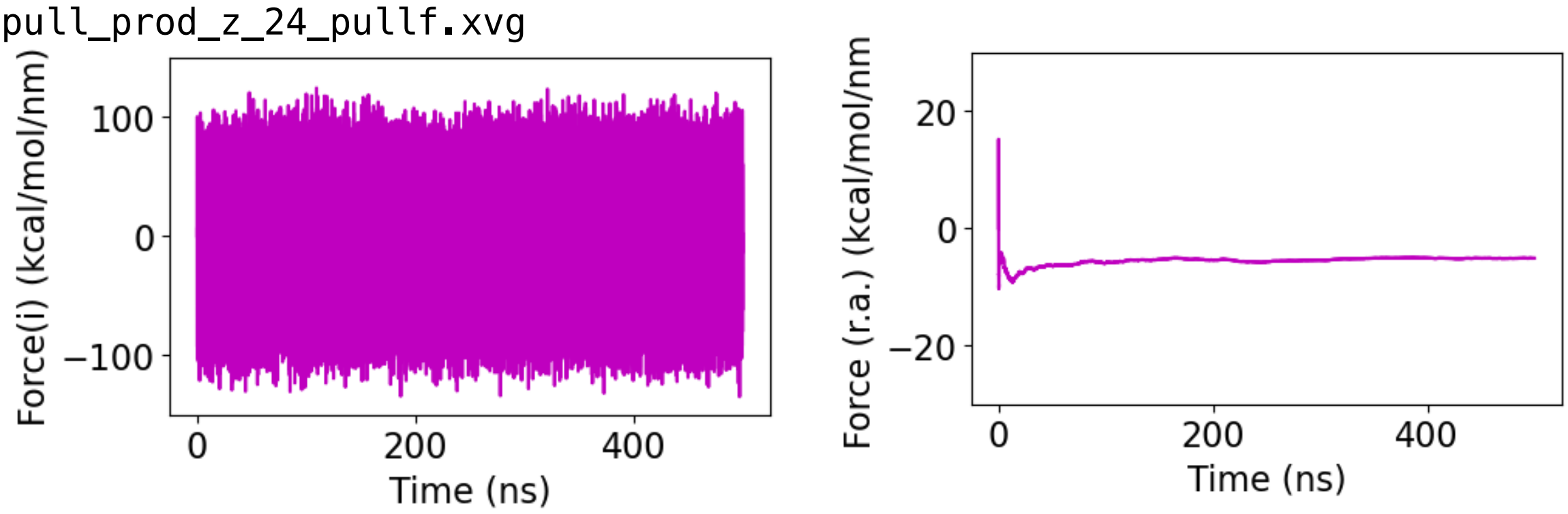

pull_prod_z_25_pullf.xvg

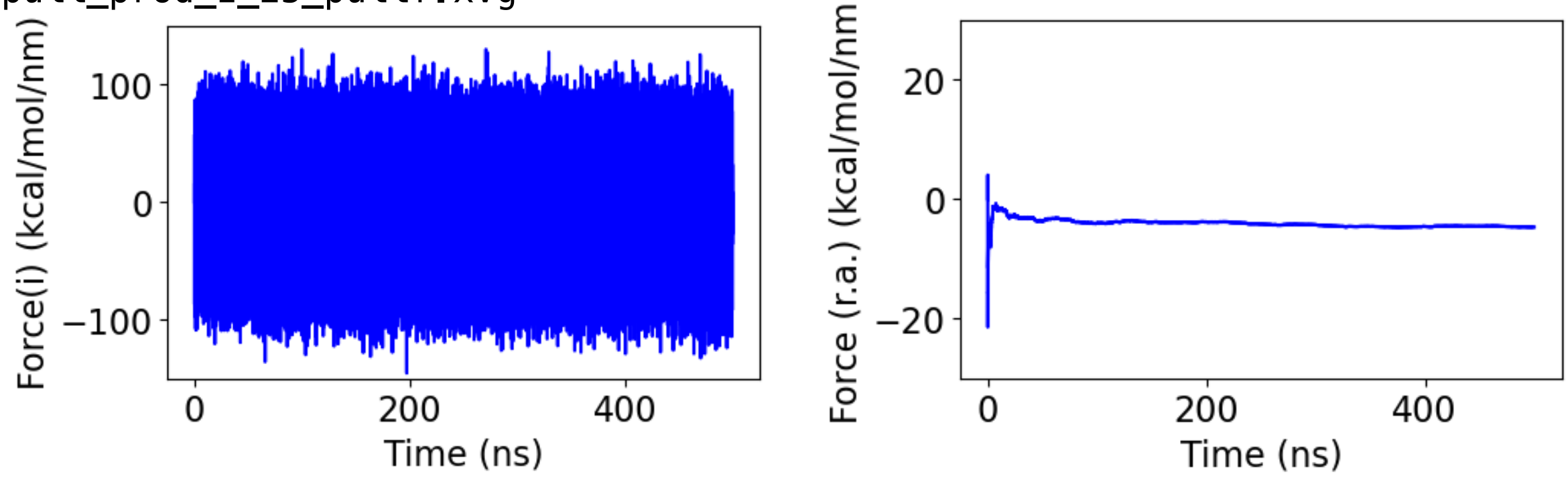

pull_prod_z_26_pullf.xvg

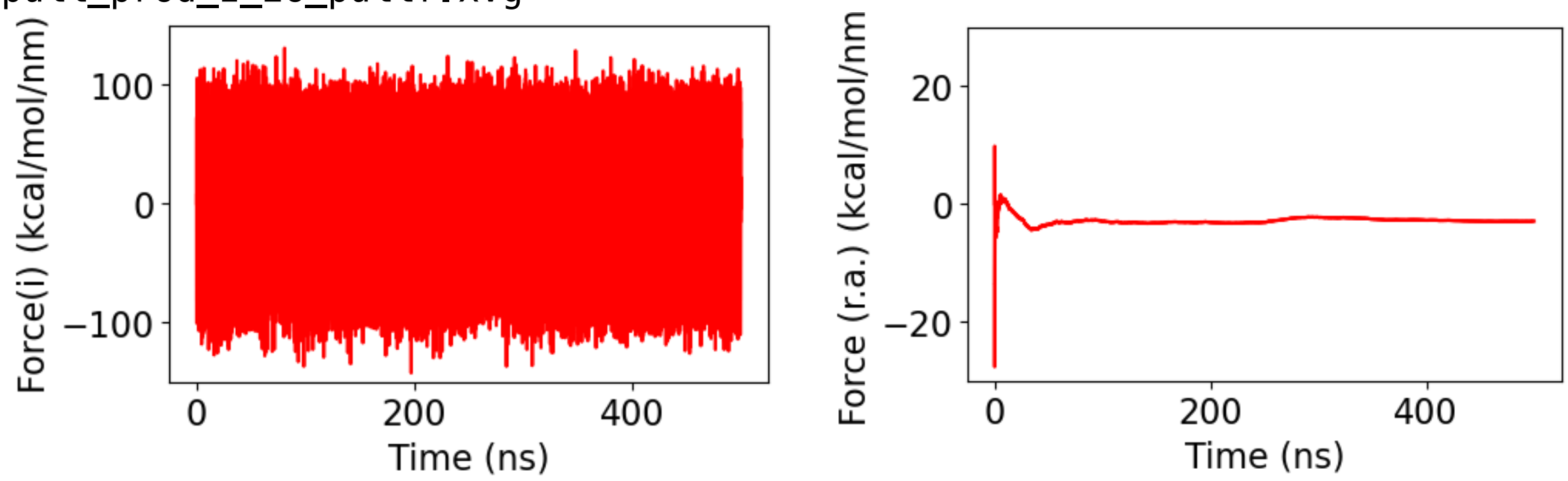

pull_prod_z_27_pullf.xvg

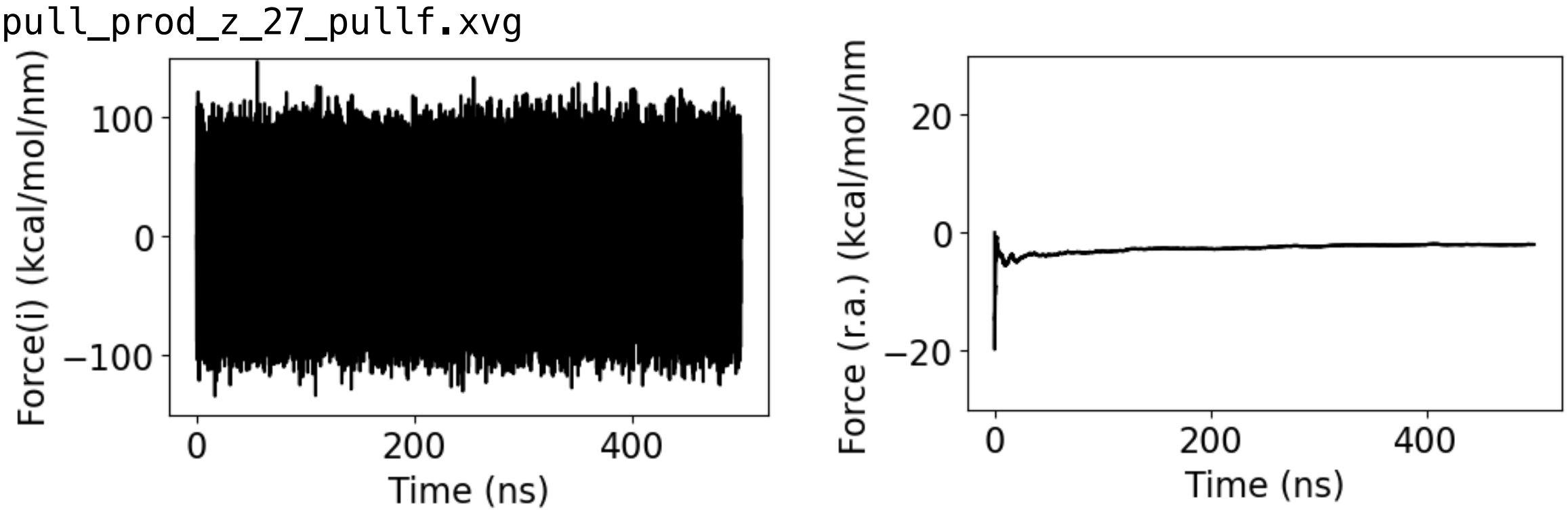

pull_prod_z_28_pullf.xvg

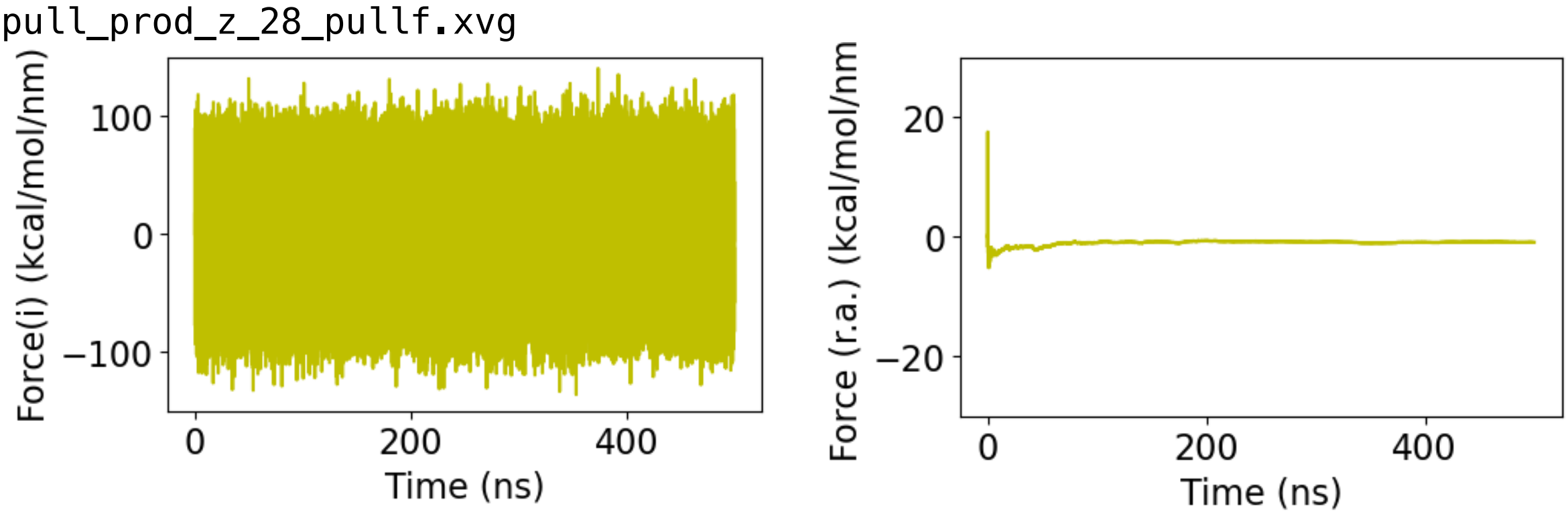

pull_prod_z_29_pullf.xvg

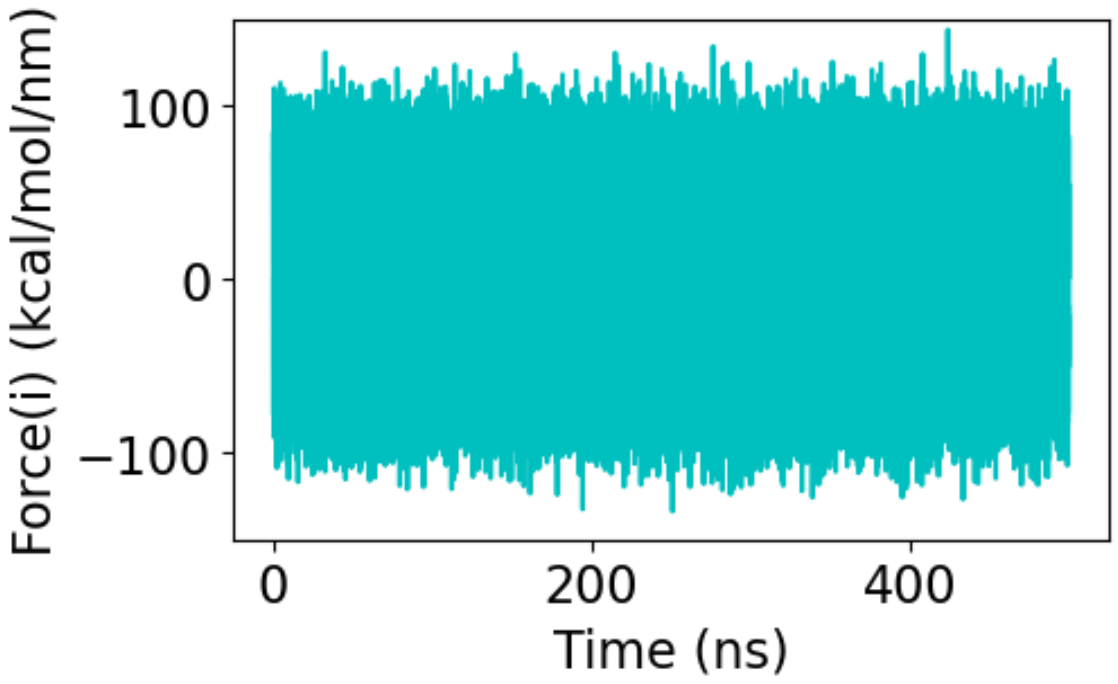

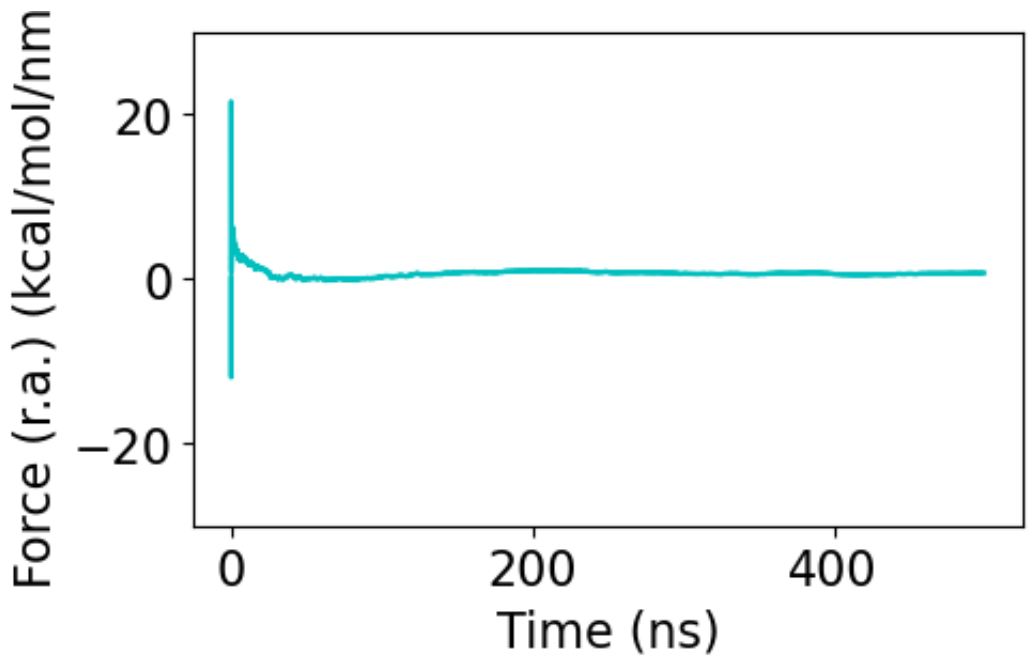

pull_prod_z_30_pullf.xvg

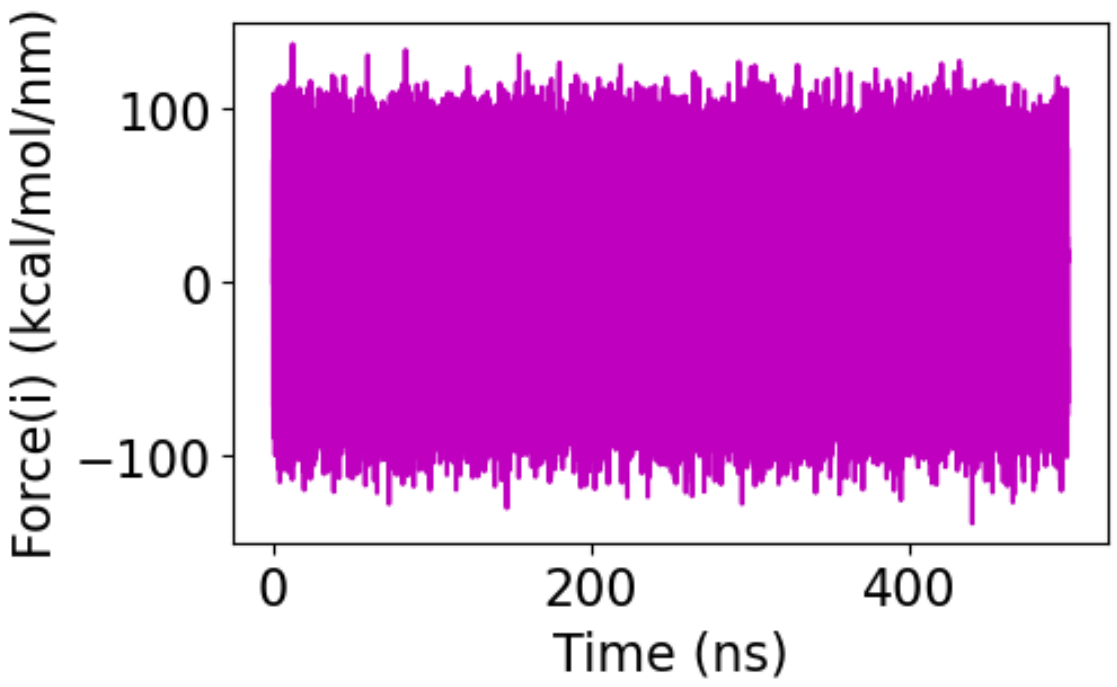

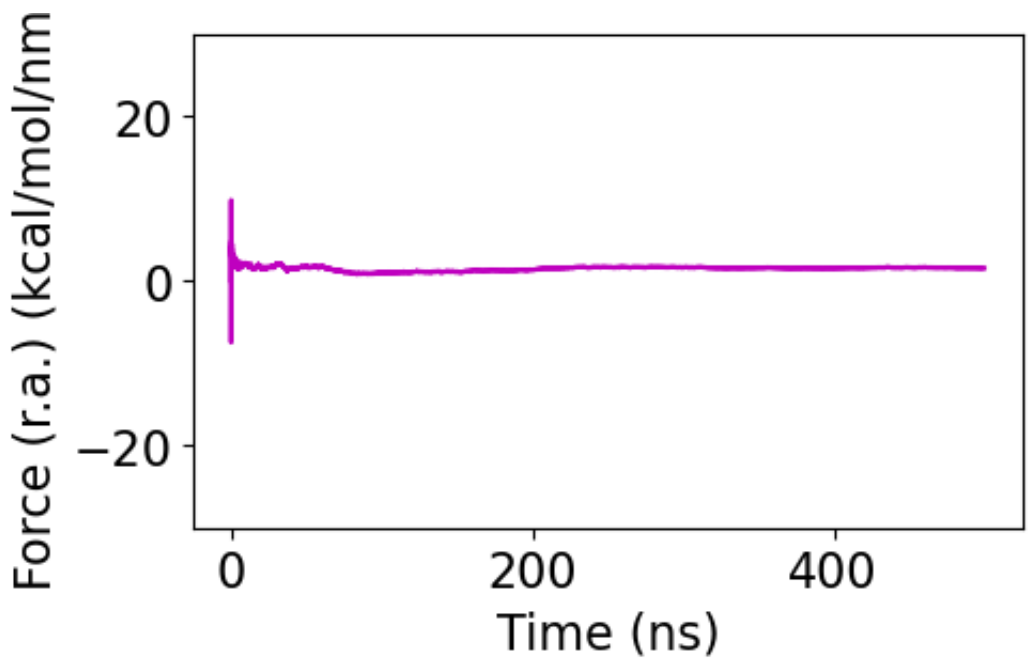

pull_prod_z_31_pullf.xvg

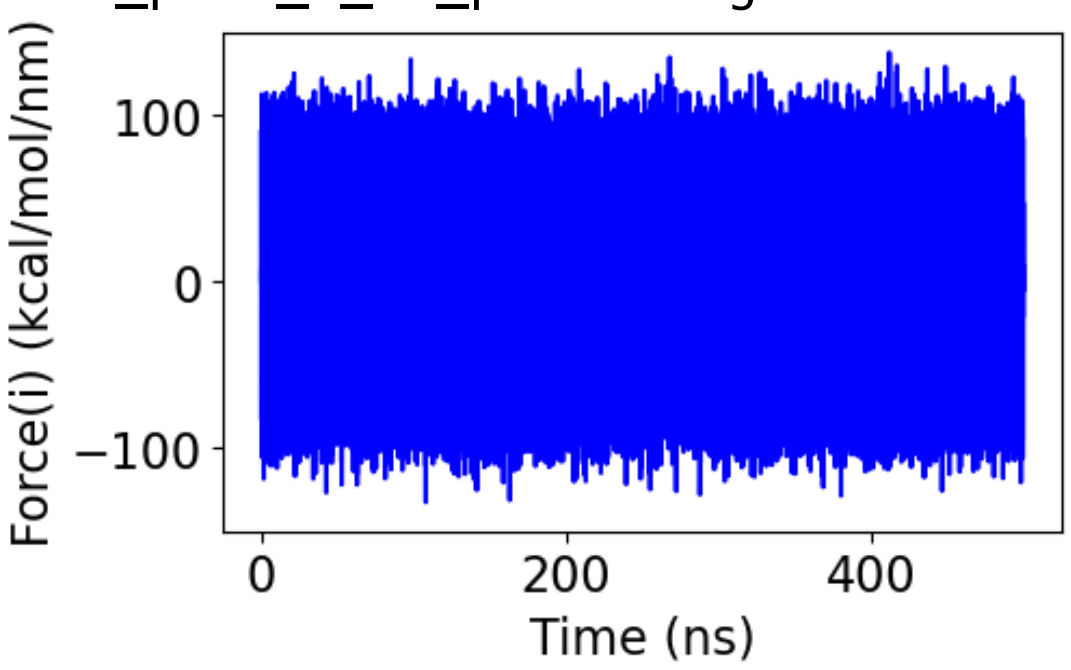

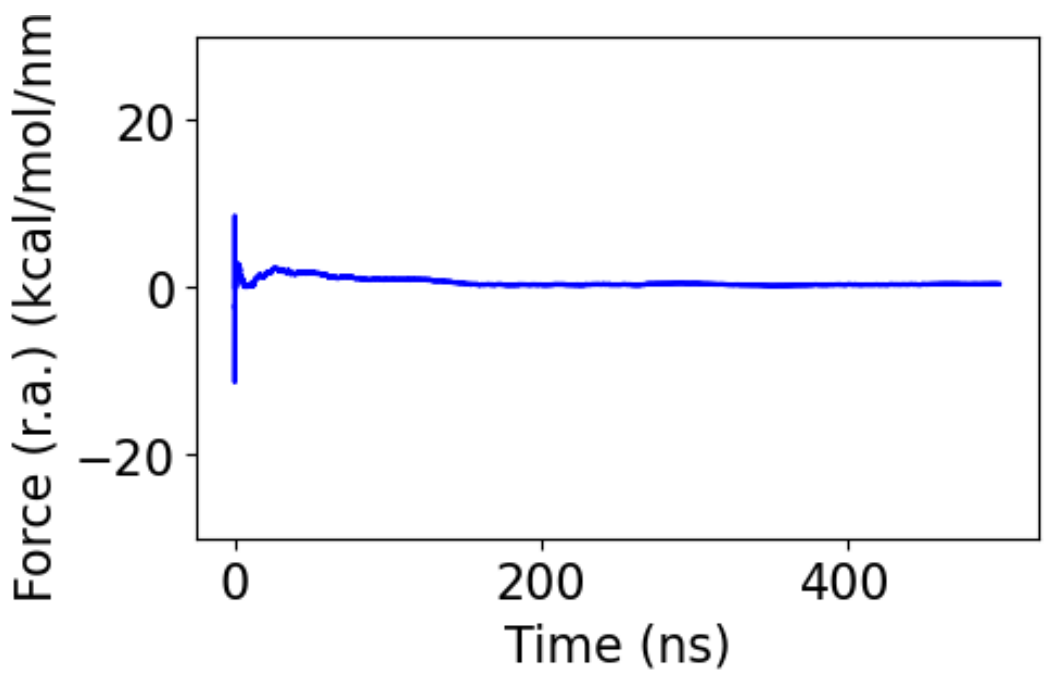

pull_prod_z_32_pullf.xvg

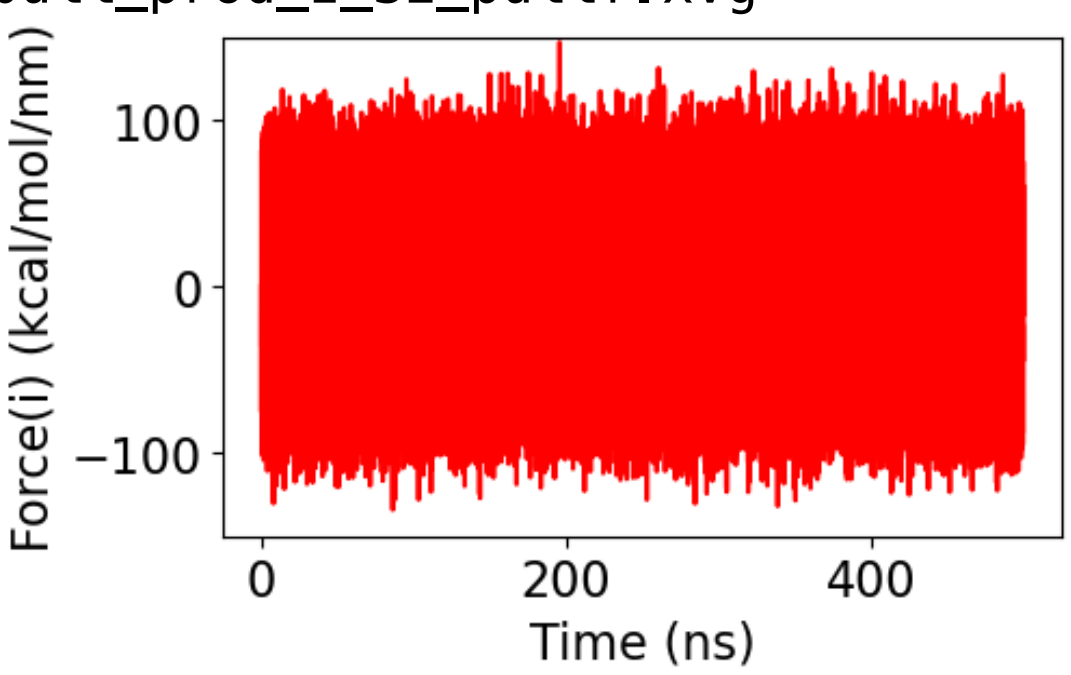

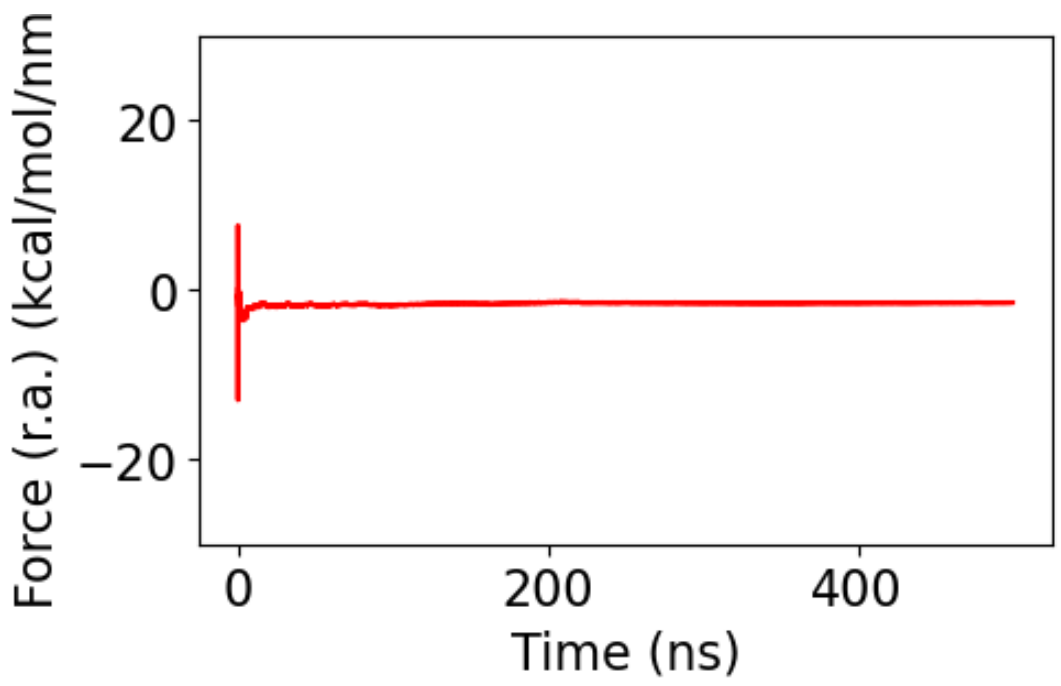

pull_prod_z_33_pullf.xvg

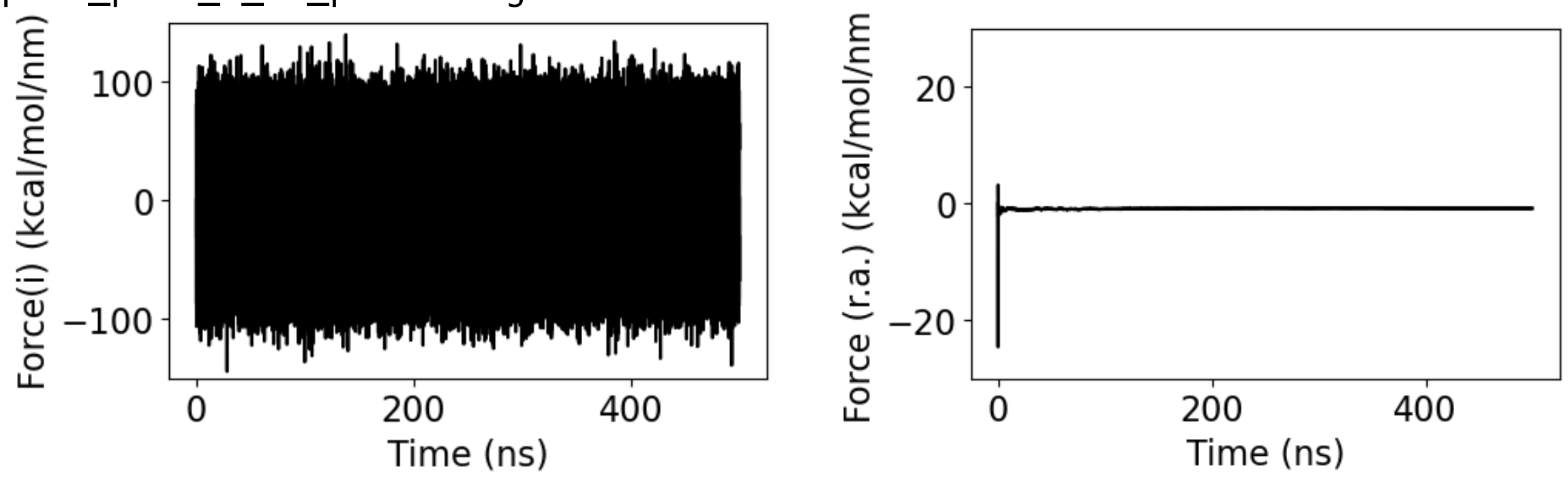

pull_prod_z_34_pullf.xvg

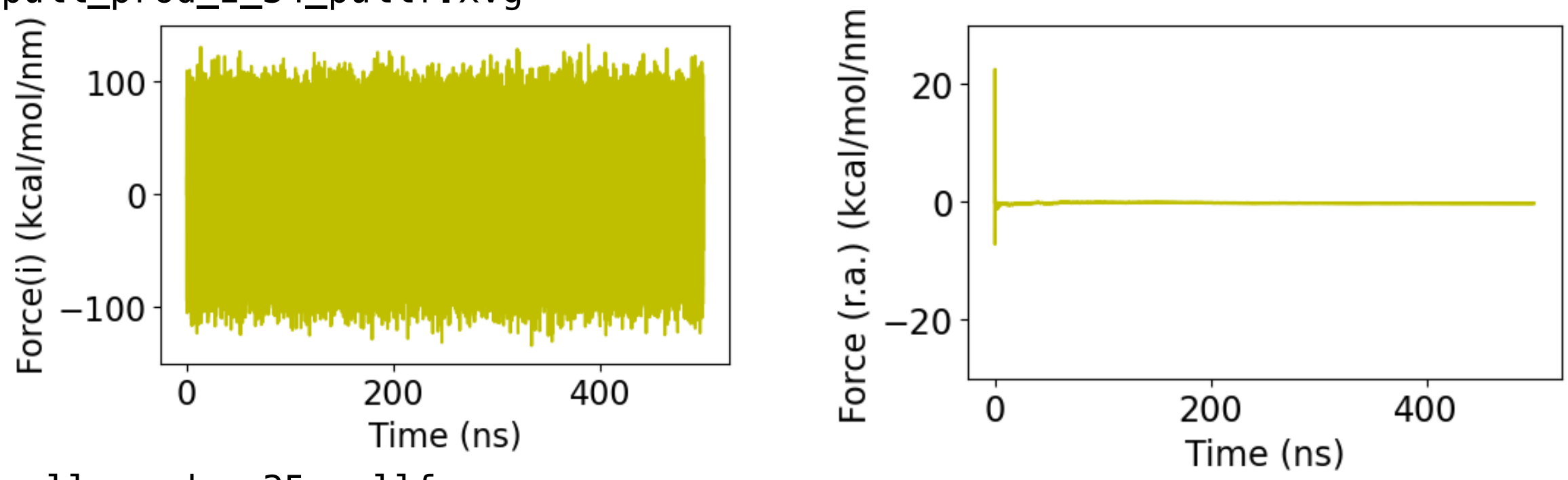

pull_prod_z_35_pullf.xvg

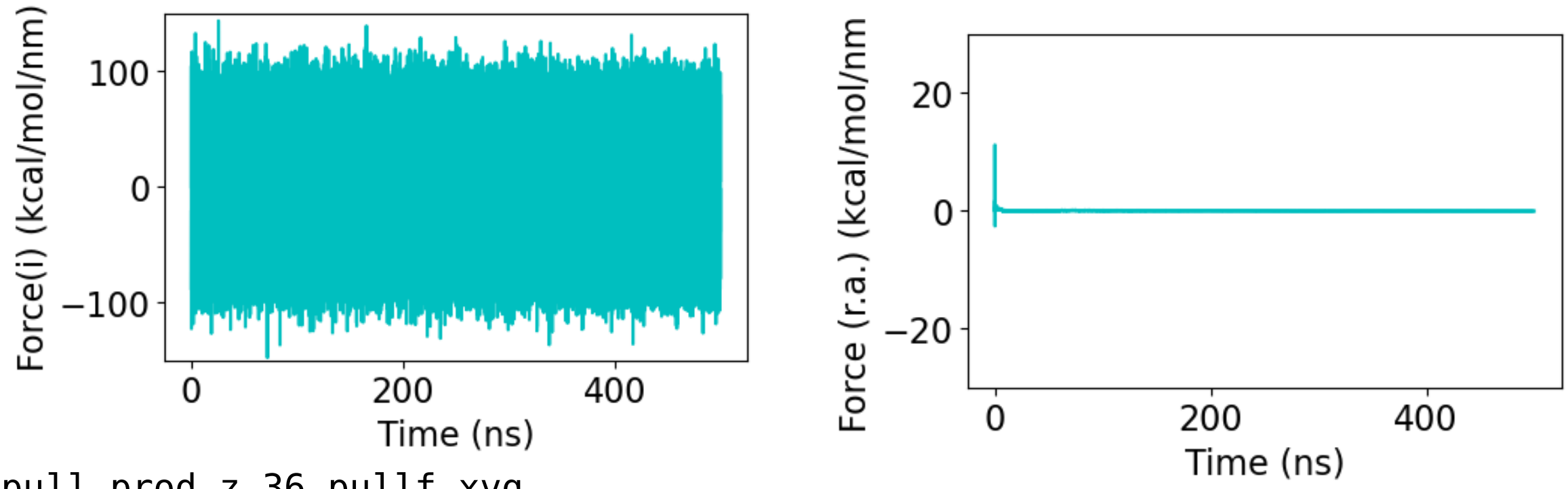

pull_prod_z_36_pullf.xvg

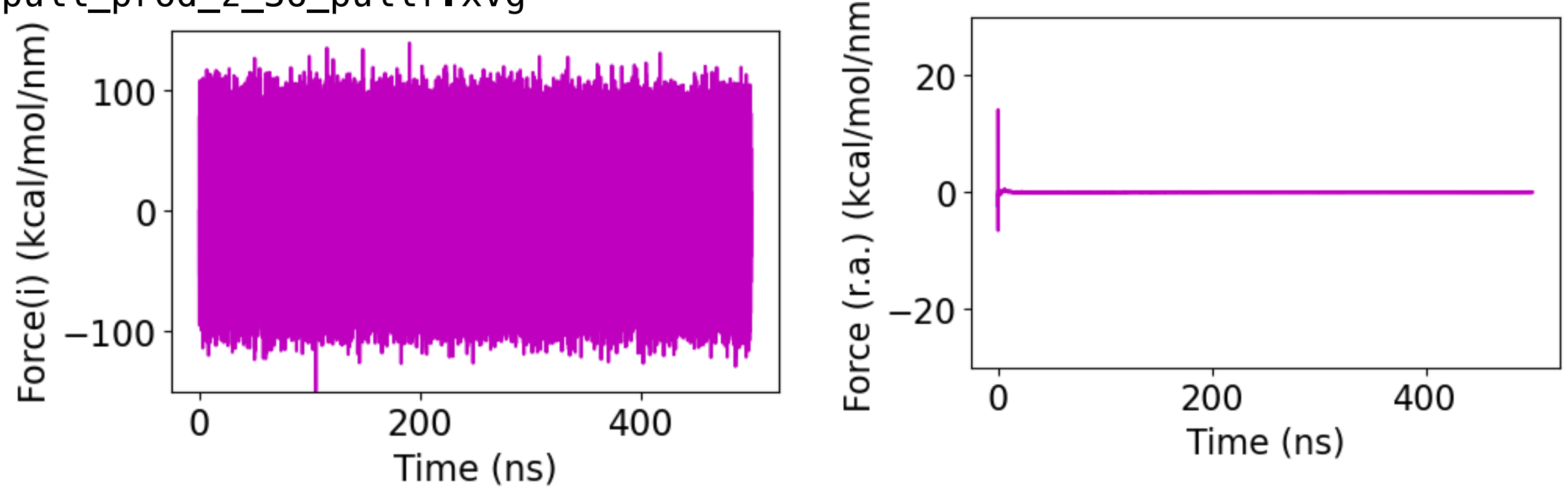

pull_prod_z_37_pullf.xvg

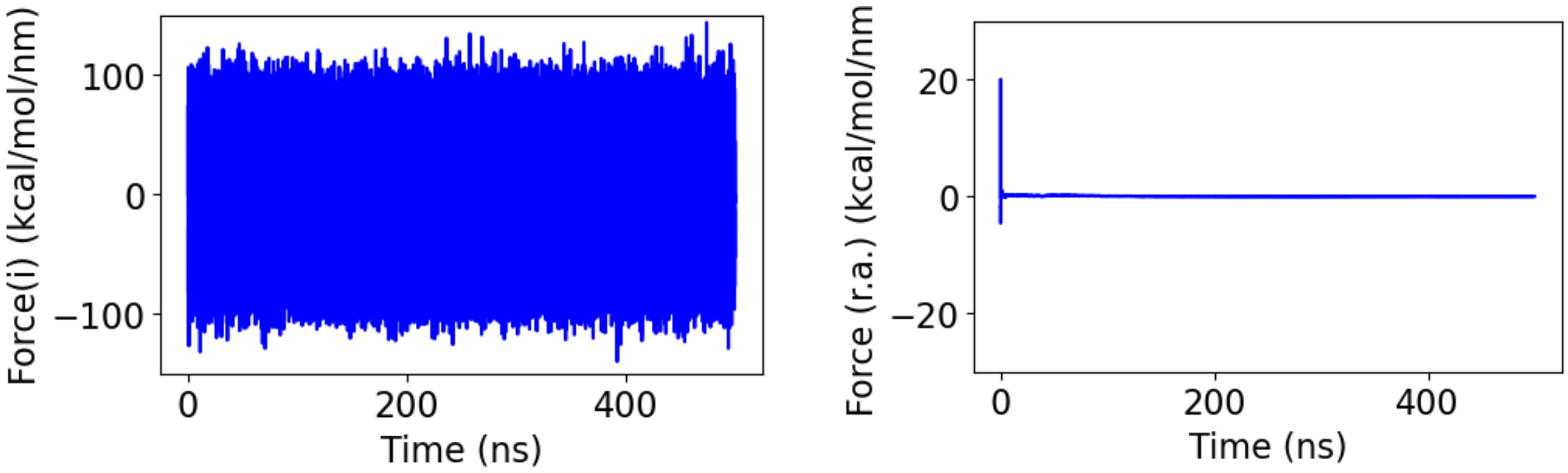

pull_prod_z_38_pullf.xvg

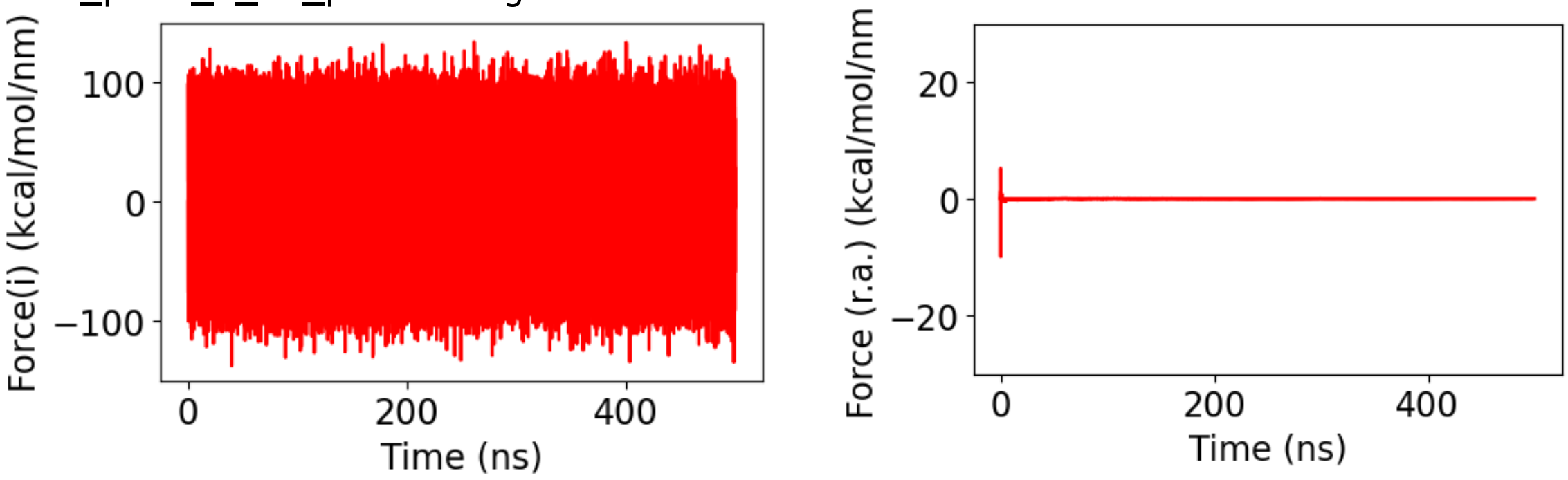

pull_prod_z_39_pullf.xvg

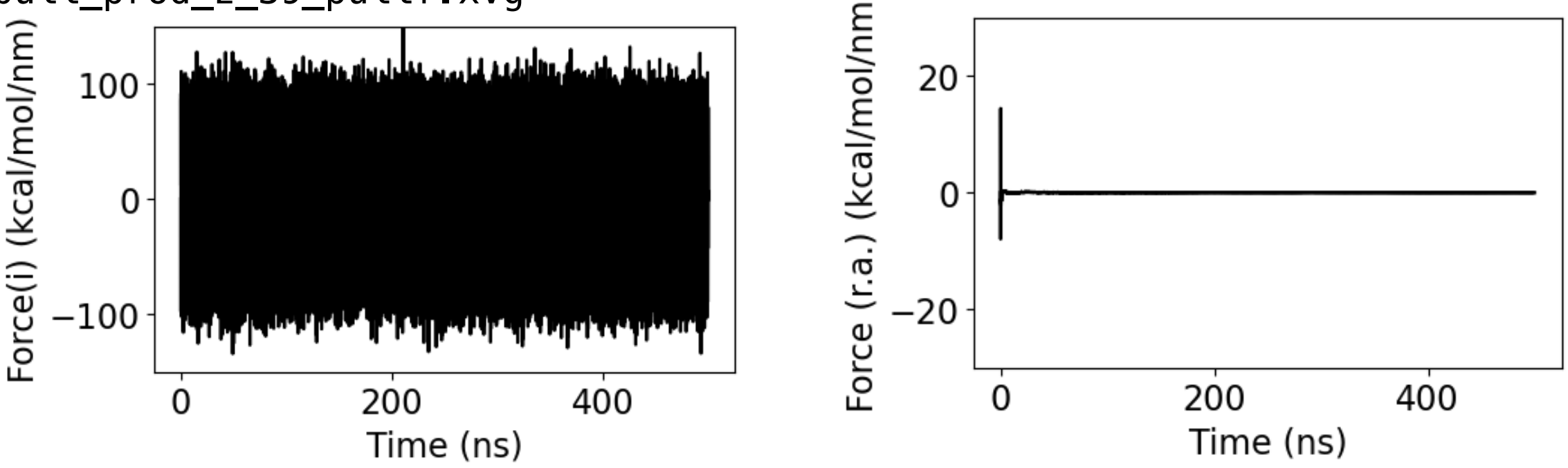

Force time series for caffeine:

pull_prod_z_0_pullf.xvg

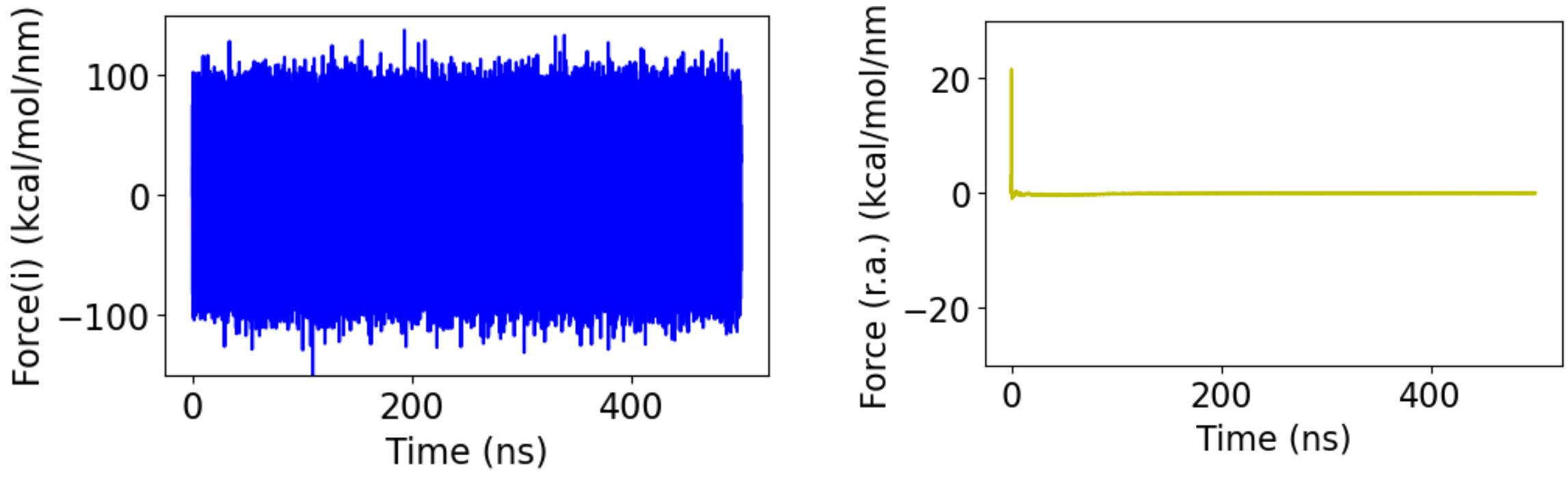

pull_prod_z_1_pullf.xvg

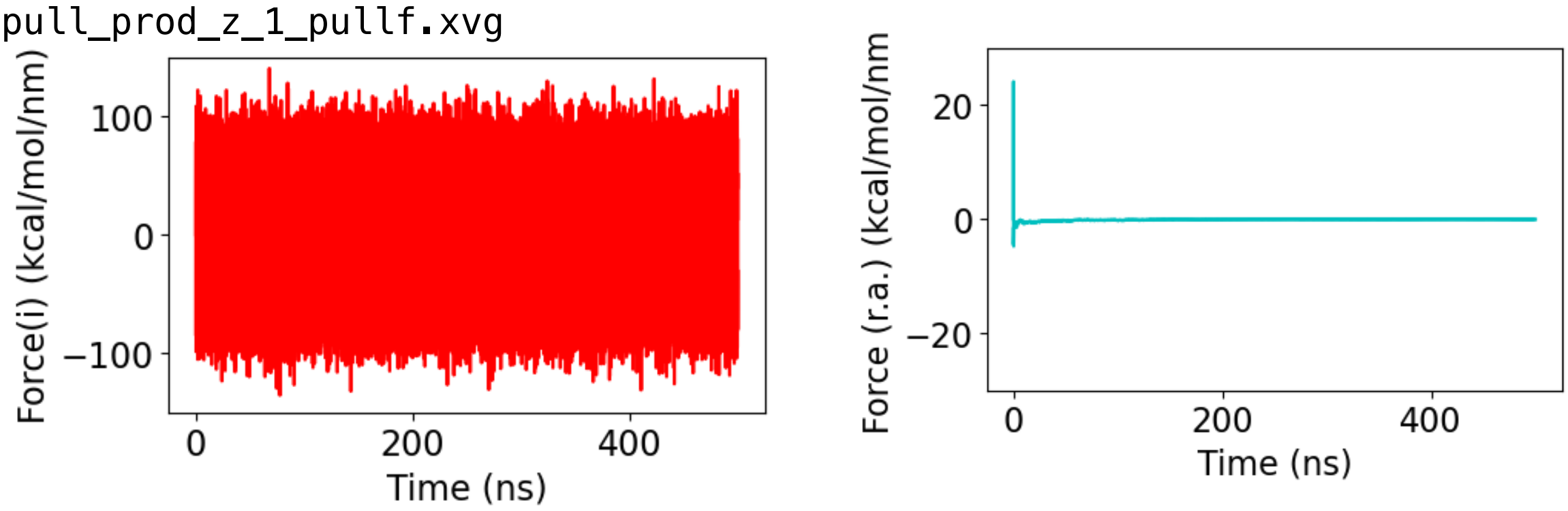

pull_prod_z_2_pullf.xvg

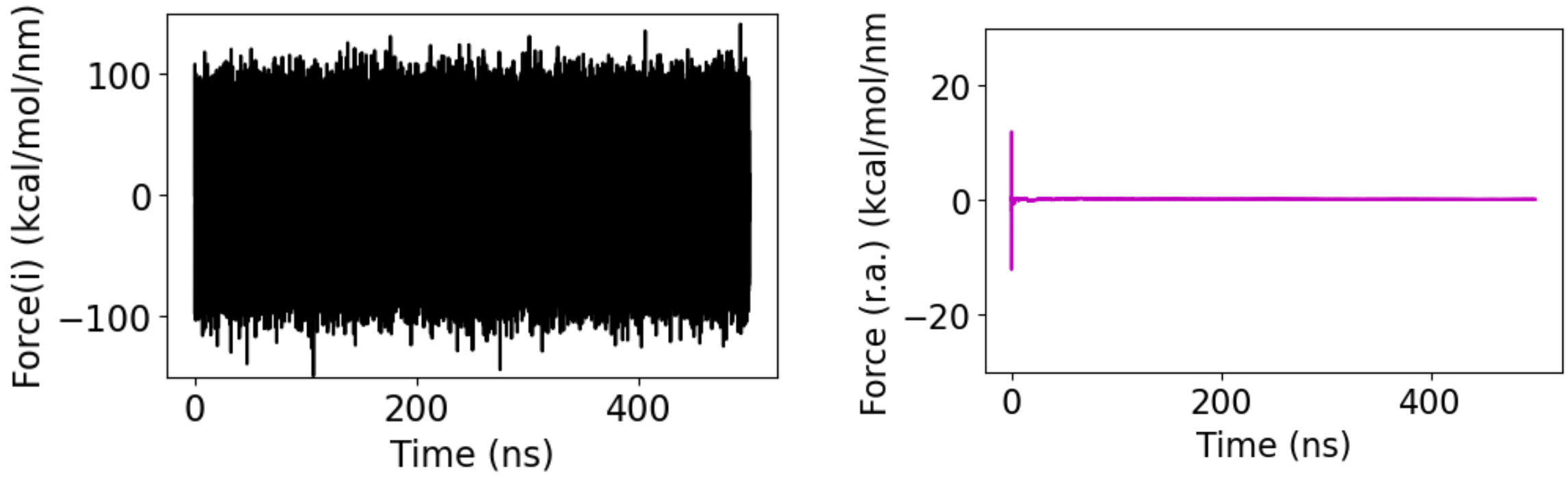

pull_prod_z_3_pullf.xvg

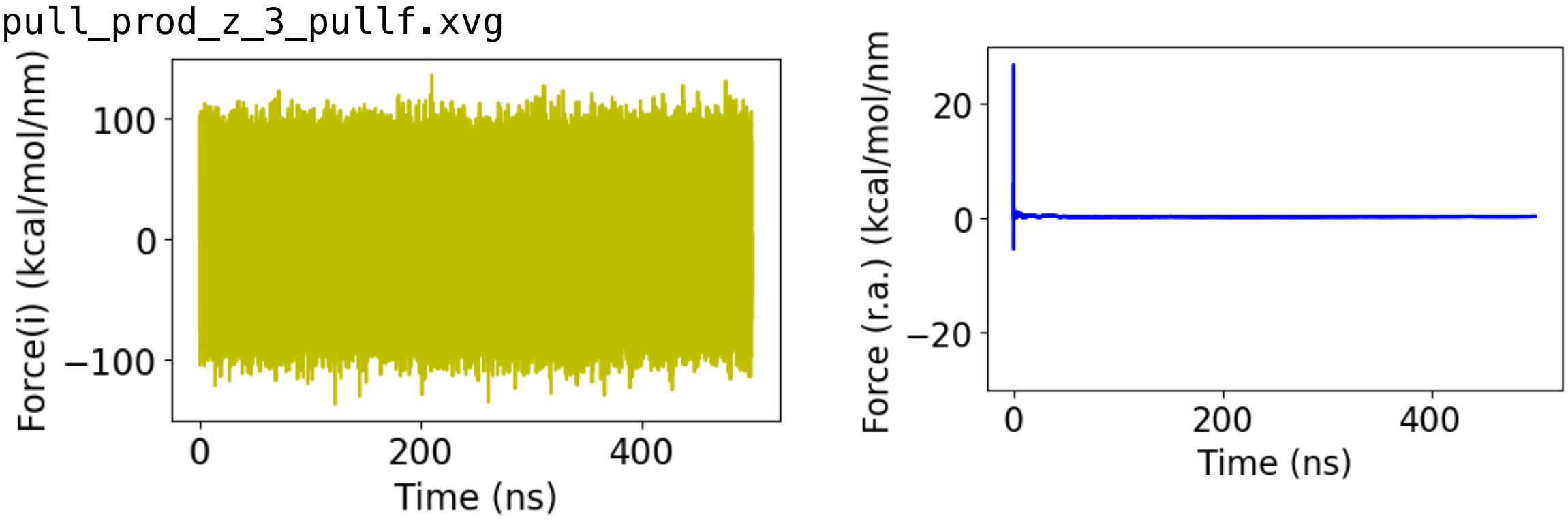

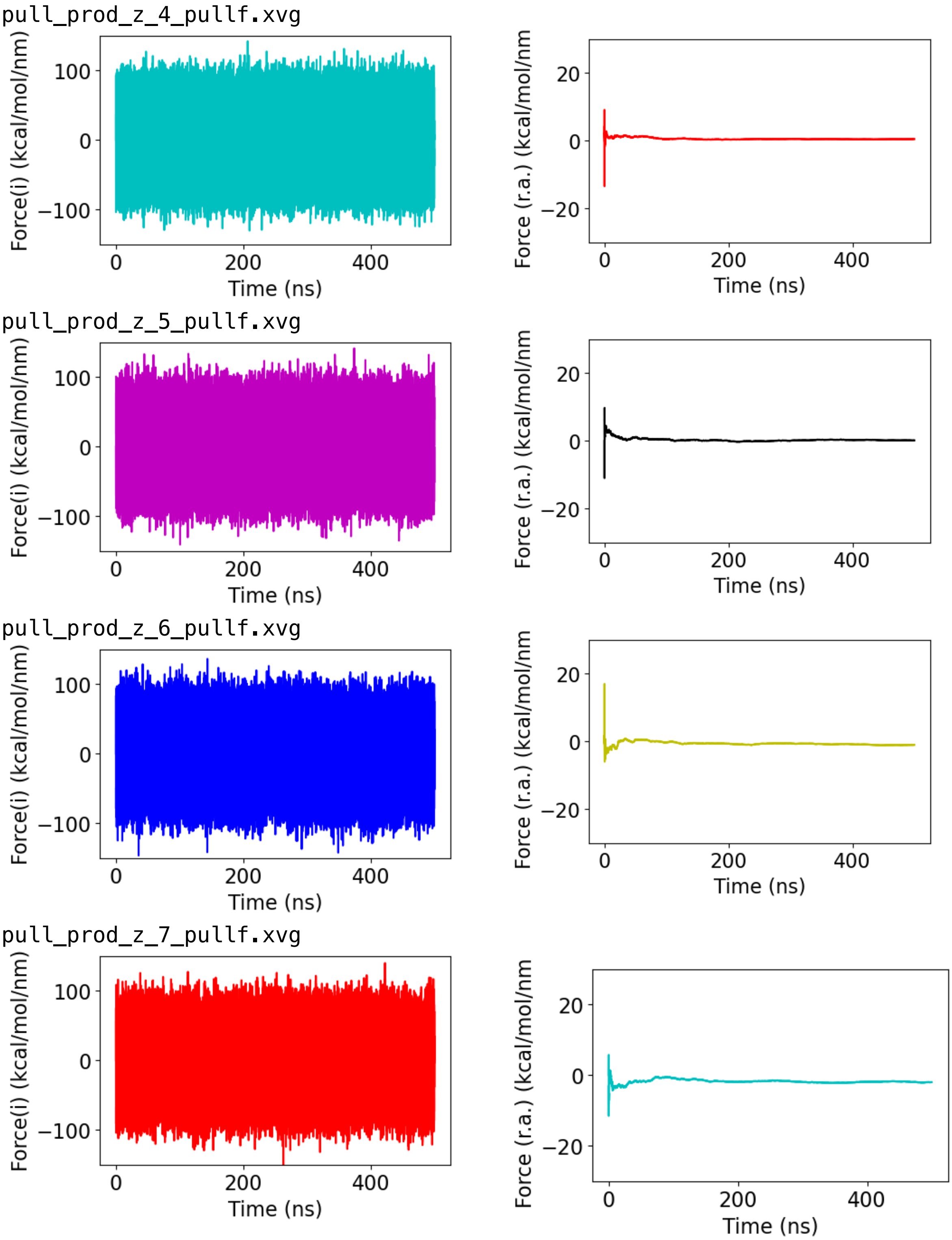
pull_prod_z_4_pullf.xvg
Force(i) (kcal/mol/nm)
Time (ns)
Force (r.a.) (kcal/mol/nm
Time (ns)
pull_prod_z_5_pullf.xvg
Force(i) (kcal/mol/nm)
Time (ns)
Force (r.a.) (kcal/mol/nm
Time (ns)
pull_prod_z_6_pullf.xvg
Force(i) (kcal/mol/nm)
Time (ns)
Force (r.a.) (kcal/mol/nm
Time (ns)
pull_prod_z_7_pullf.xvg
Force(i) (kcal/mol/nm)
Time (ns)
Force (r.a.) (kcal/mol/nm
Time (ns)

pull_prod_z_8_pullf.xvg

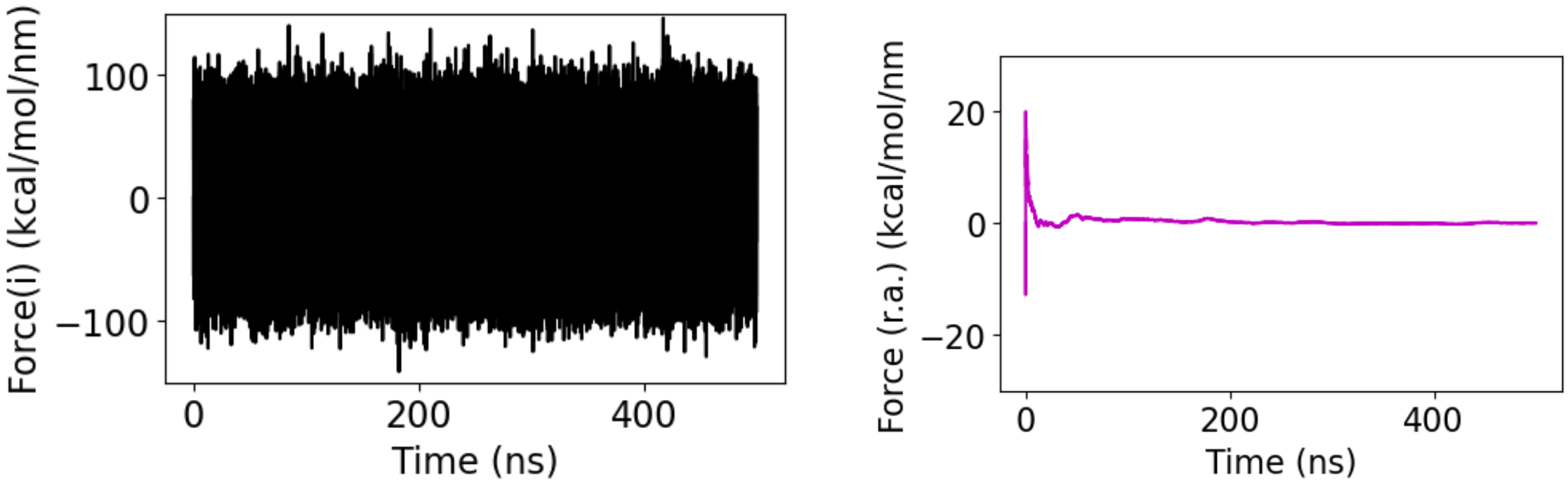

pull_prod_z_9_pullf.xvg

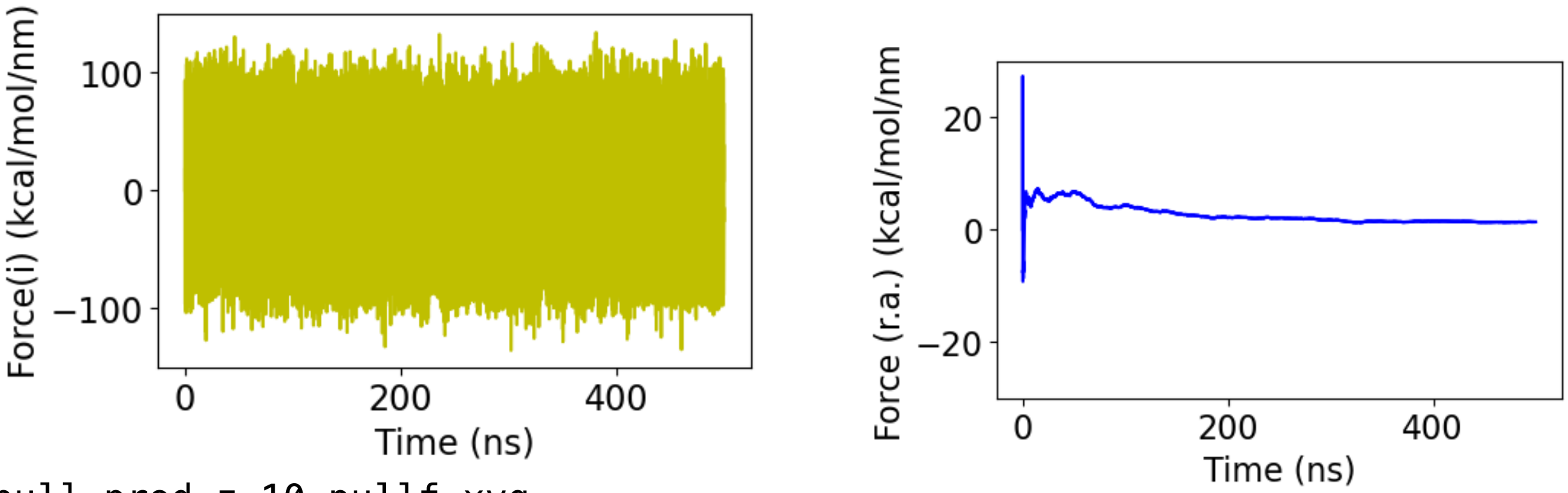

pull_prod_z_10_pullf.xvg

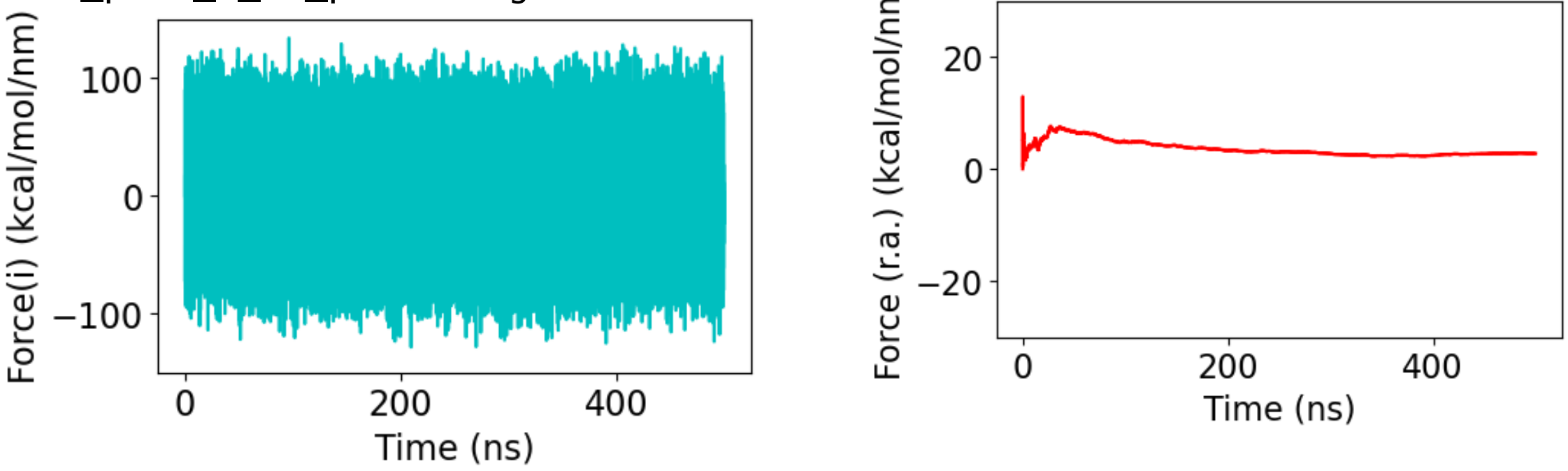

pull_prod_z_11_pullf.xvg

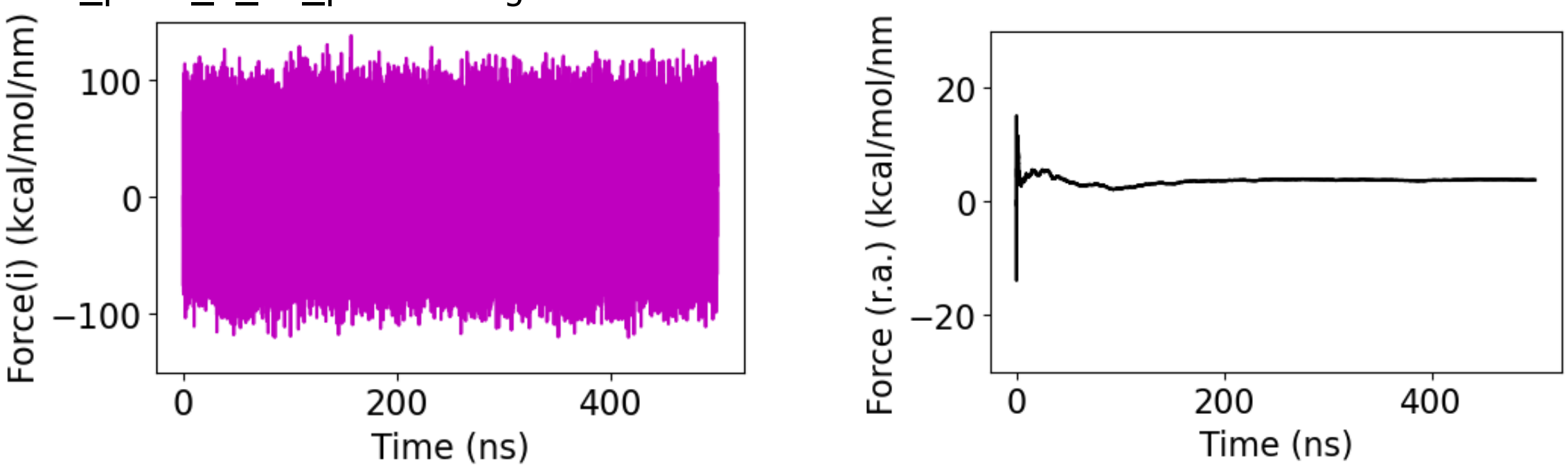

pull_prod_z_12_pullf.xvg

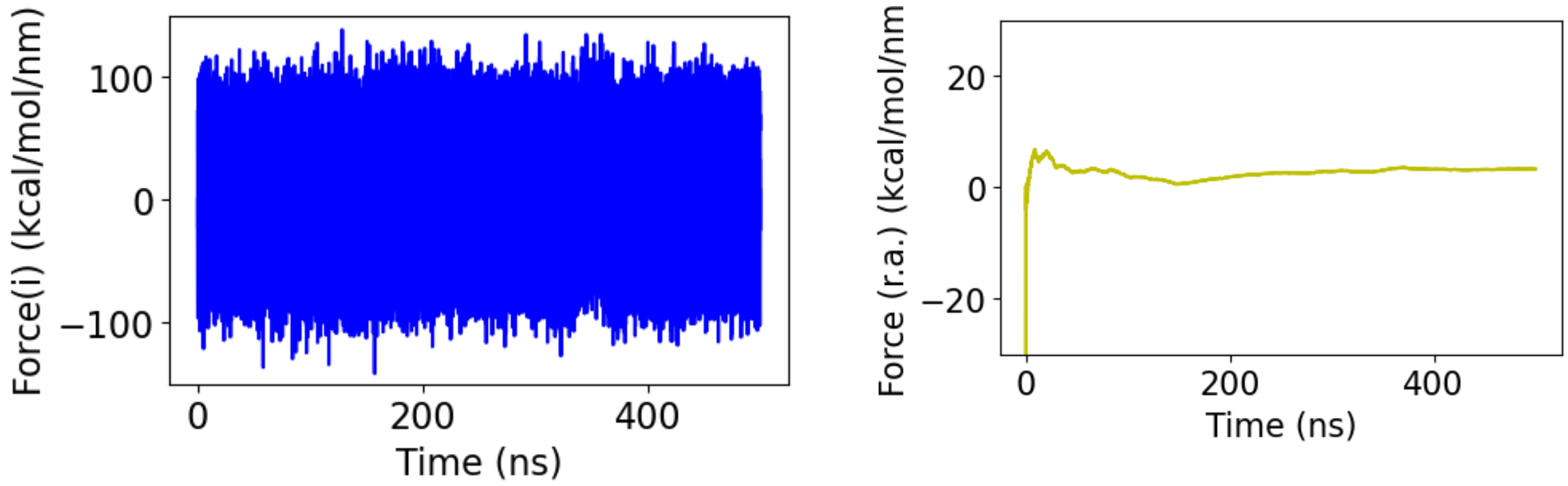

pull_prod_z_13_pullf.xvg

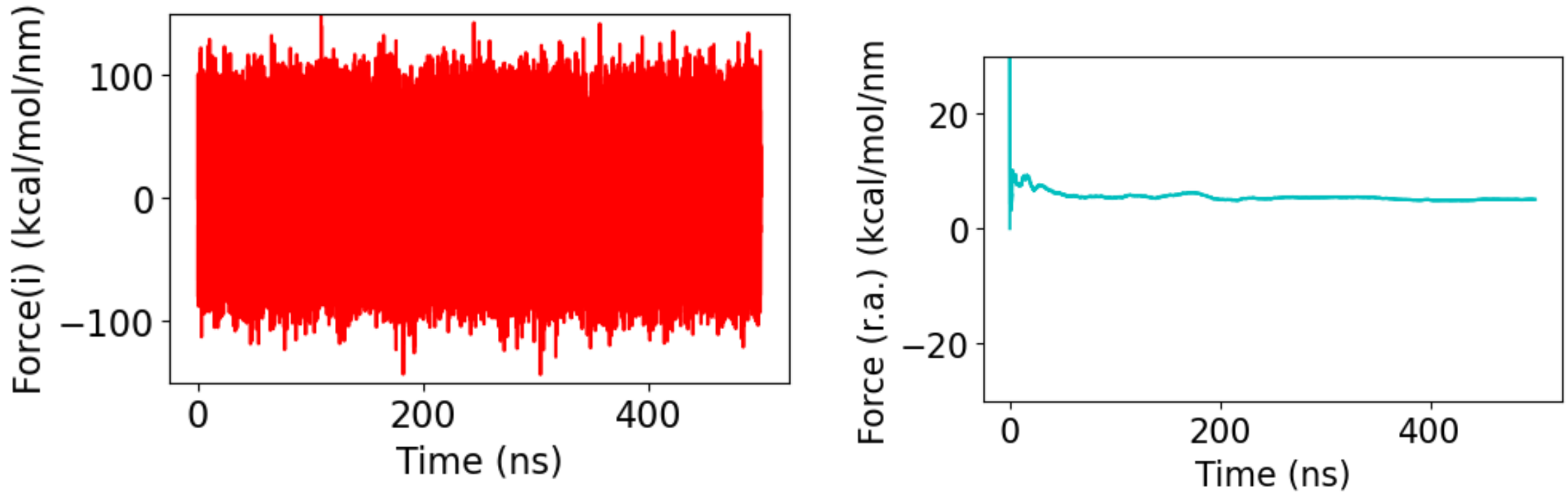

pull_prod_z_14_pullf.xvg

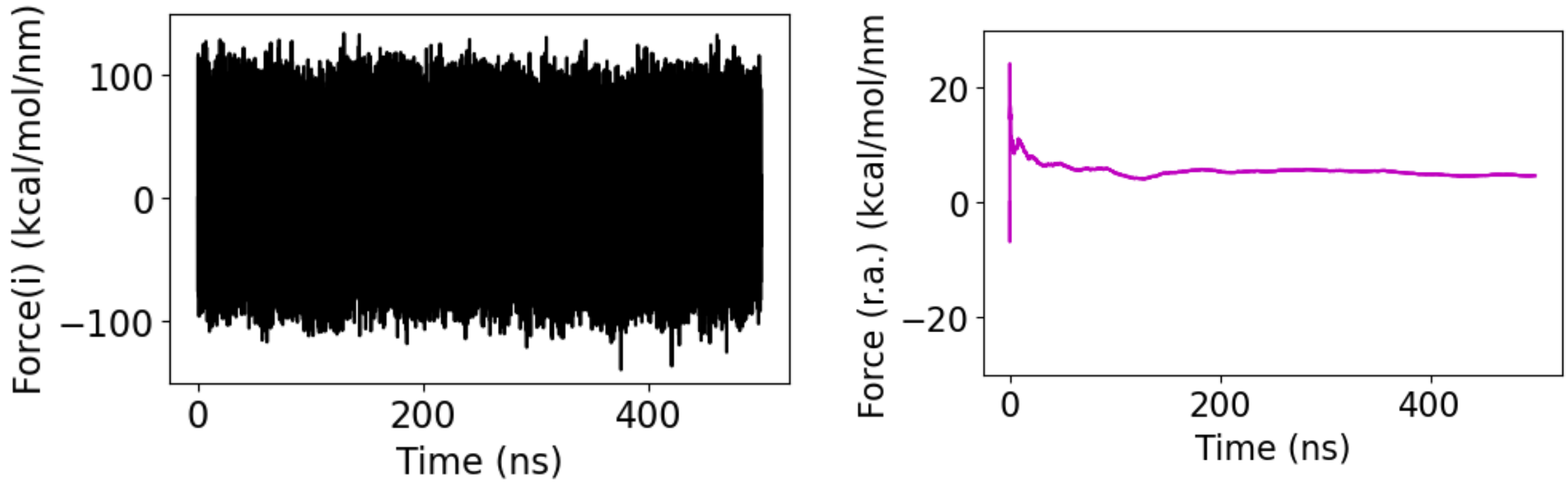

pull_prod_z_15_pullf.xvg

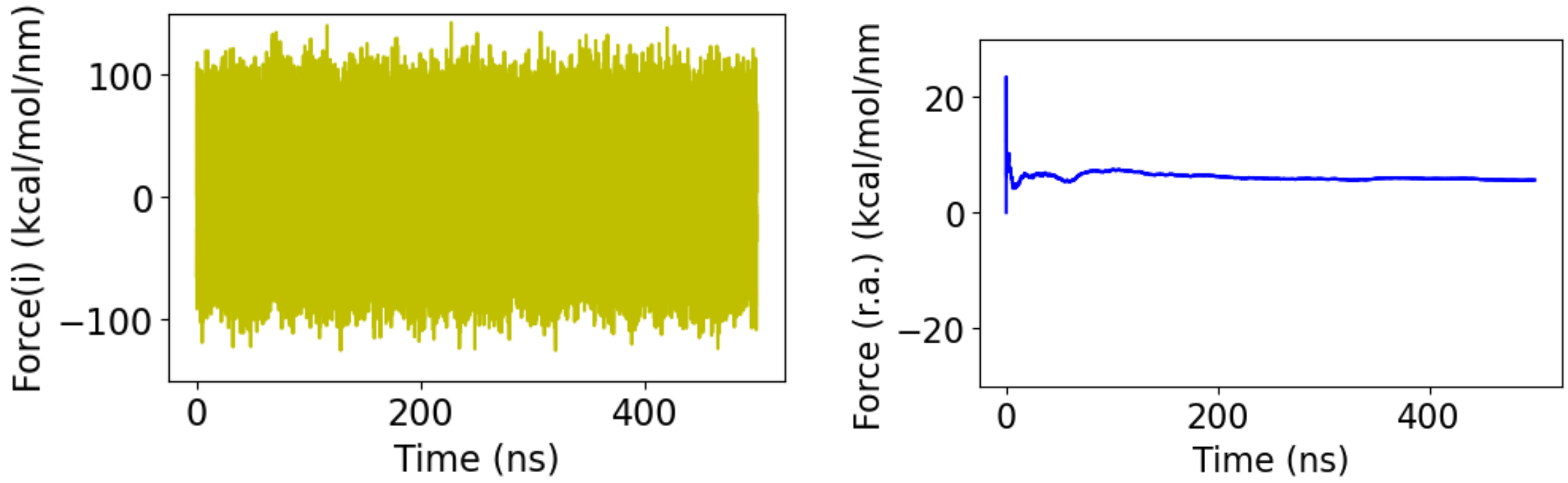

pull_prod_z_16_pullf.xvg

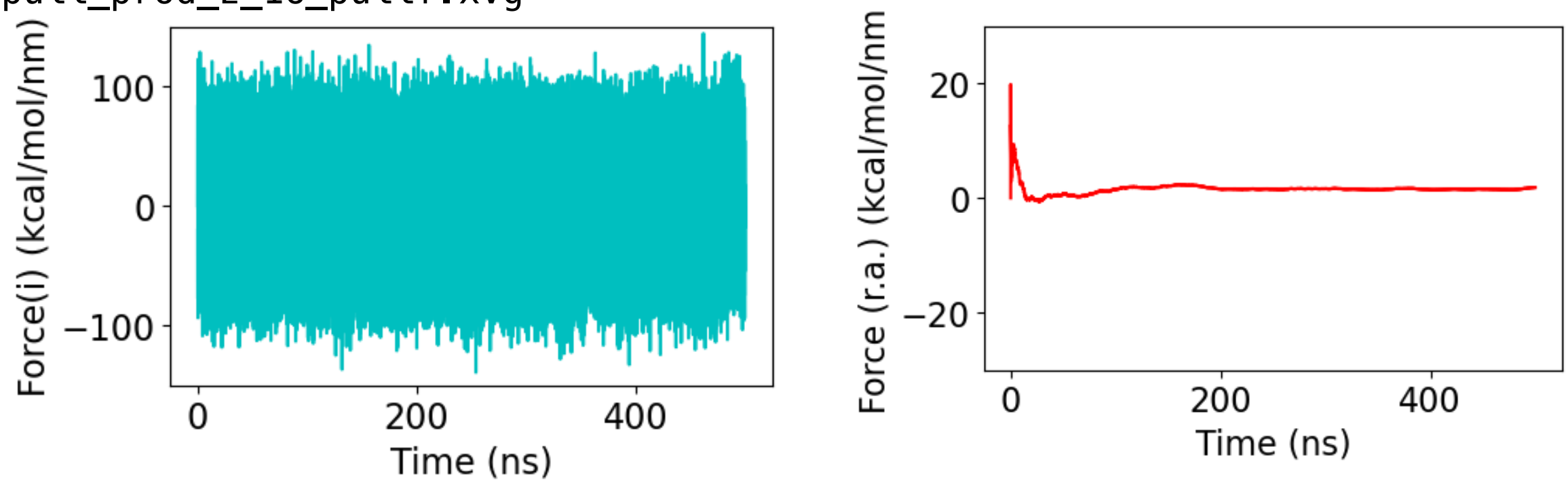

pull_prod_z_17_pullf.xvg

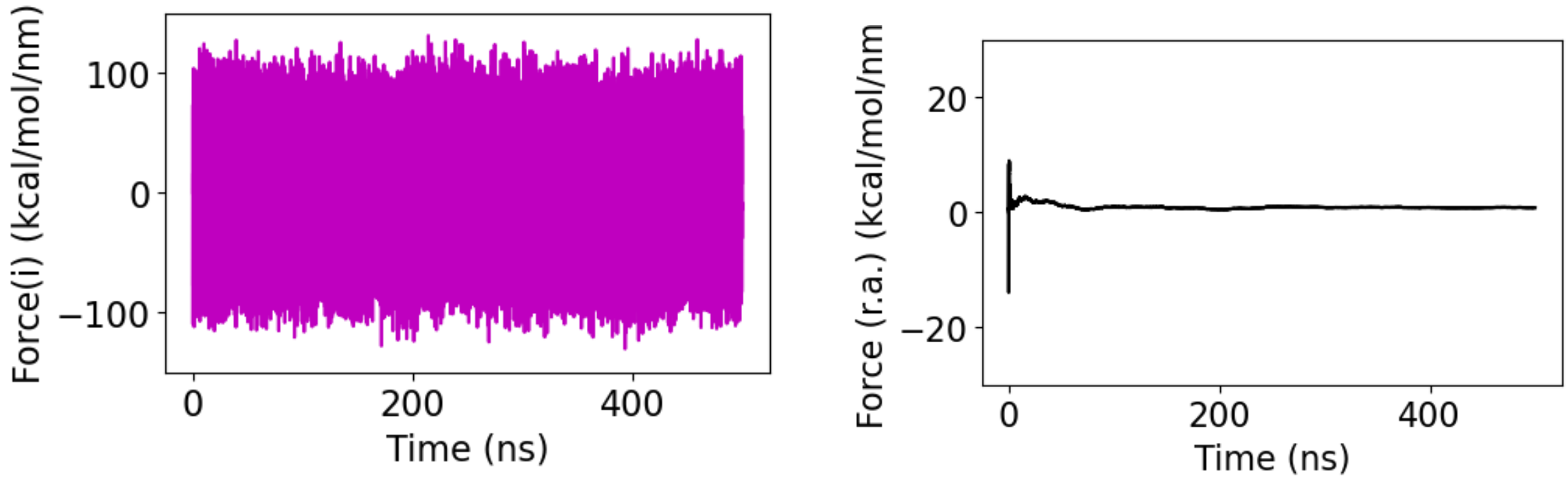

pull_prod_z_18_pullf.xvg

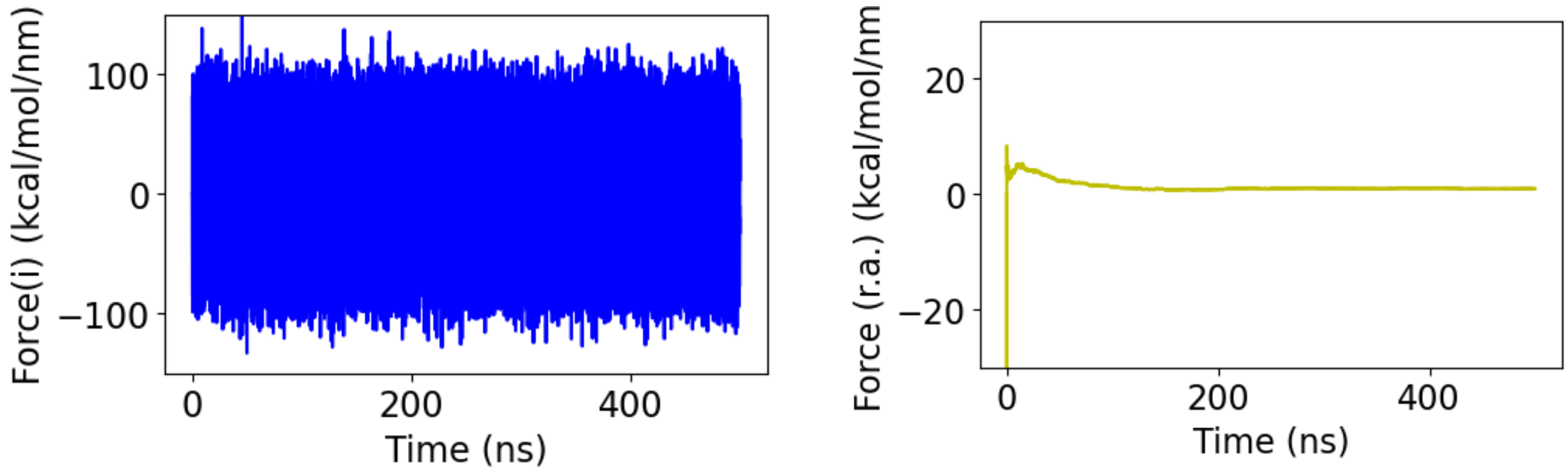

pull_prod_z_19_pullf.xvg

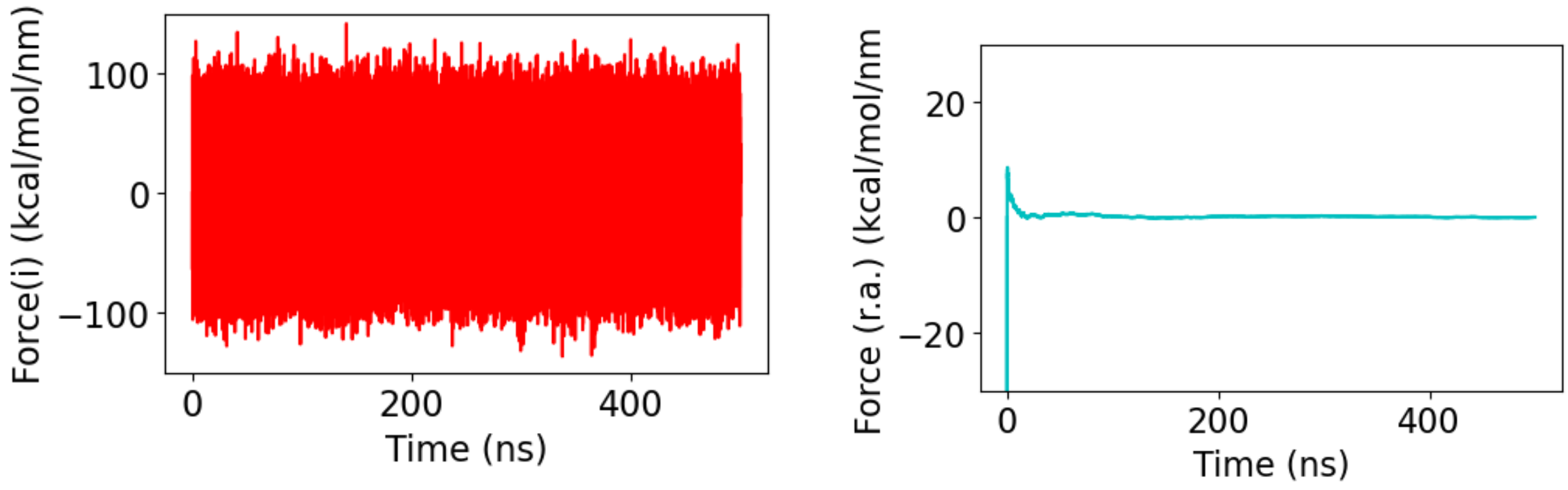

pull_prod_z_20_pullf.xvg

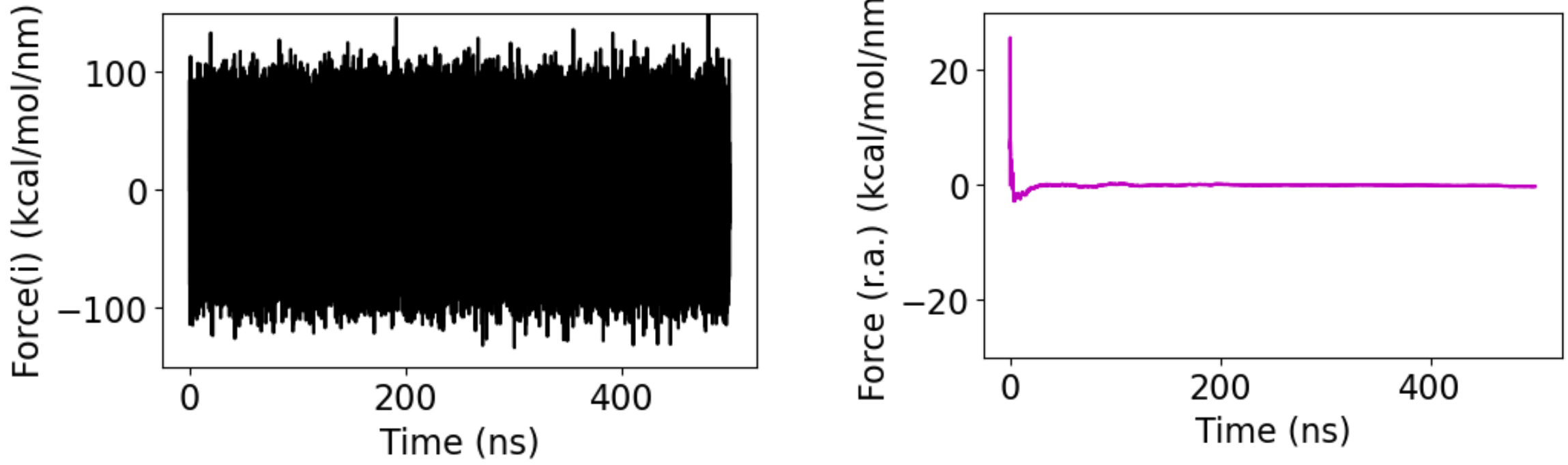

pull_prod_z_21_pullf.xvg

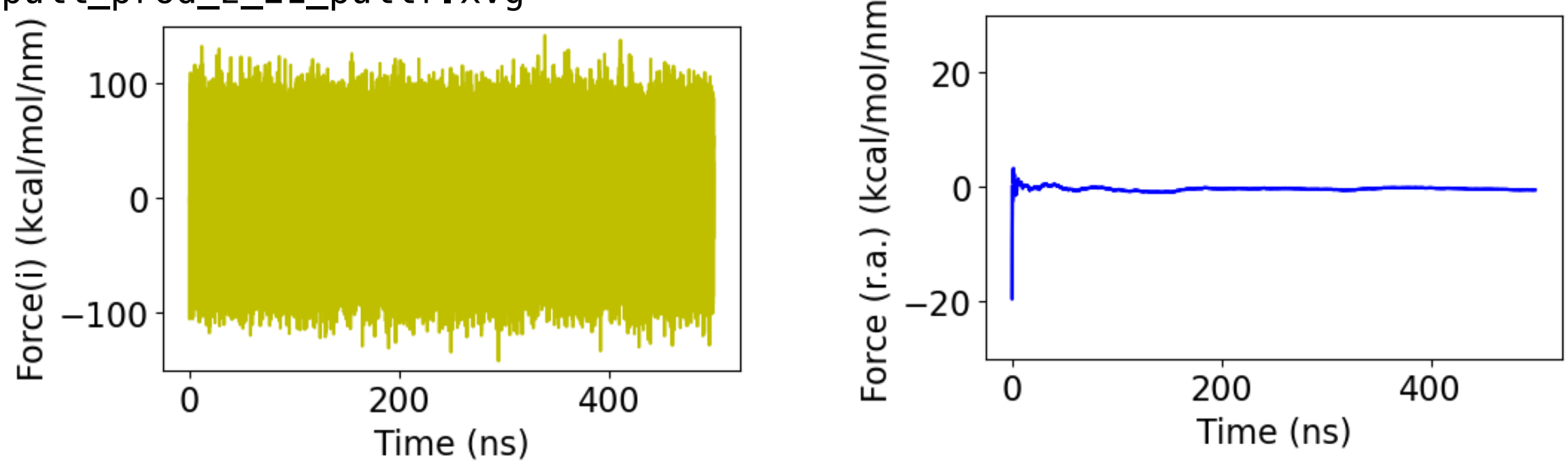

pull_prod_z_23_pullf.xvg

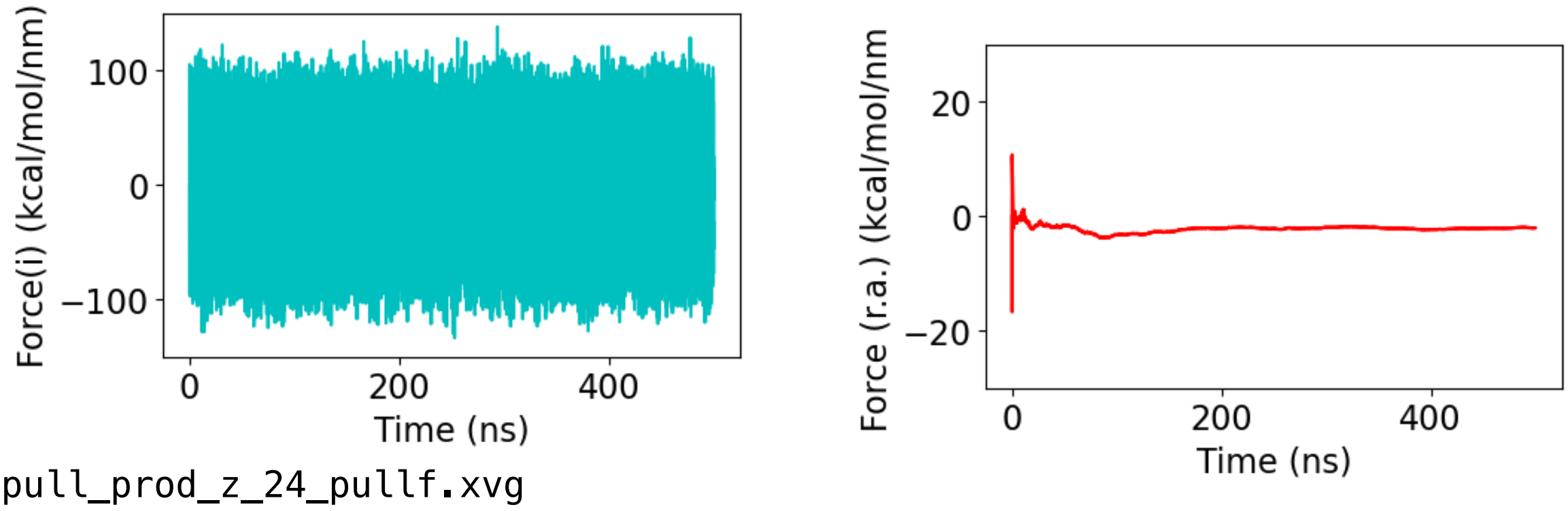

pull_prod_z_24_pullf.xvg

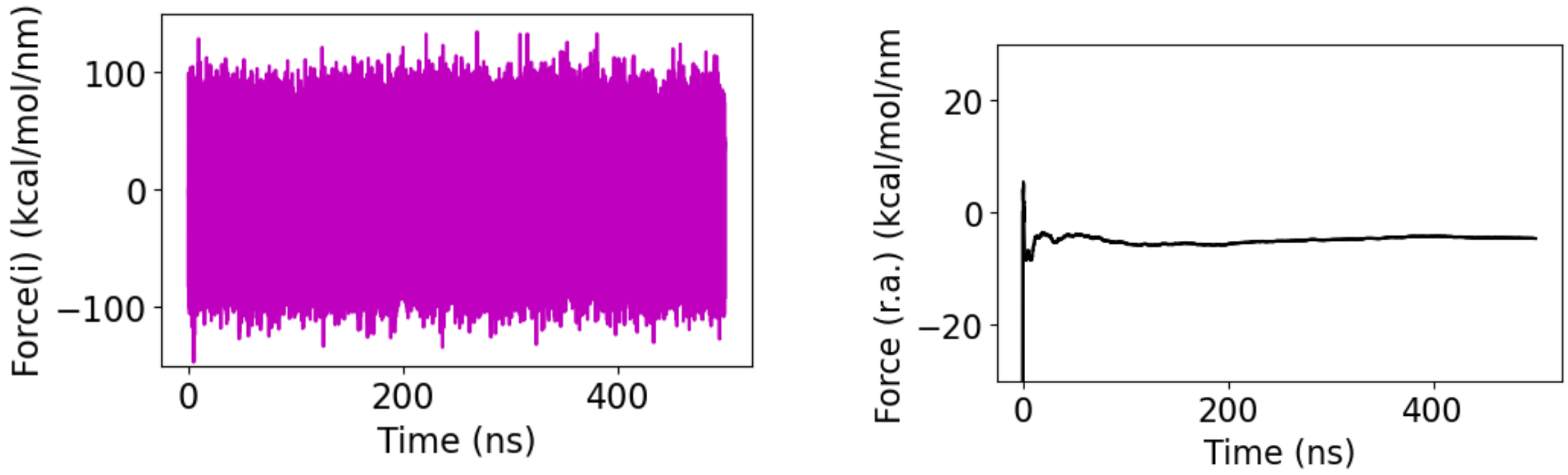

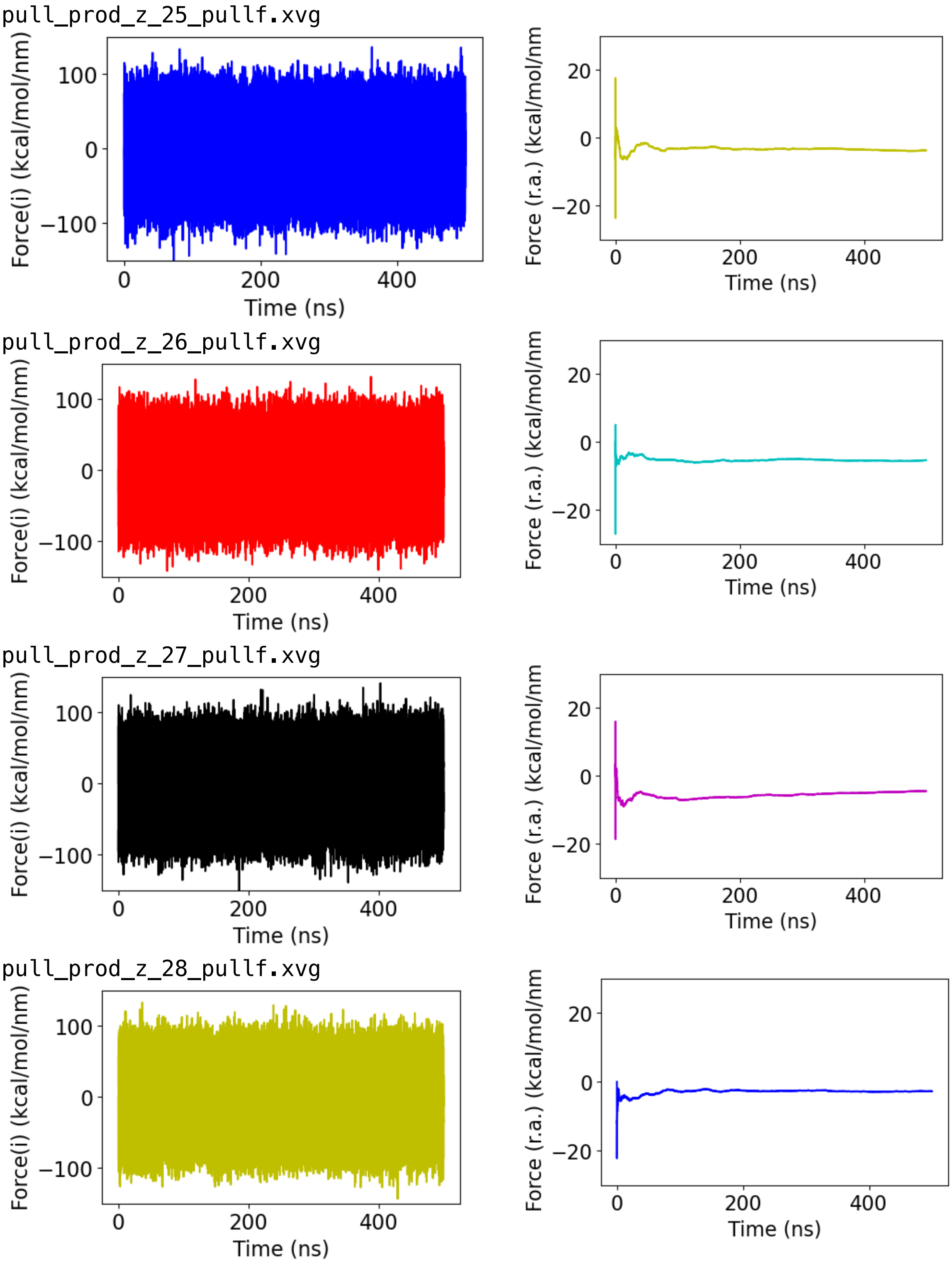
pull_prod_z_25_pullf.xvg
pull_prod_z_26_pullf.xvg
pull_prod_z_27_pullf.xvg
pull_prod_z_28_pullf.xvg
Force(i) (kcal/mol/nm)
Force (r.a.) (kcal/mol/nm
Time (ns)
100
0
−100
20
−20
200
400

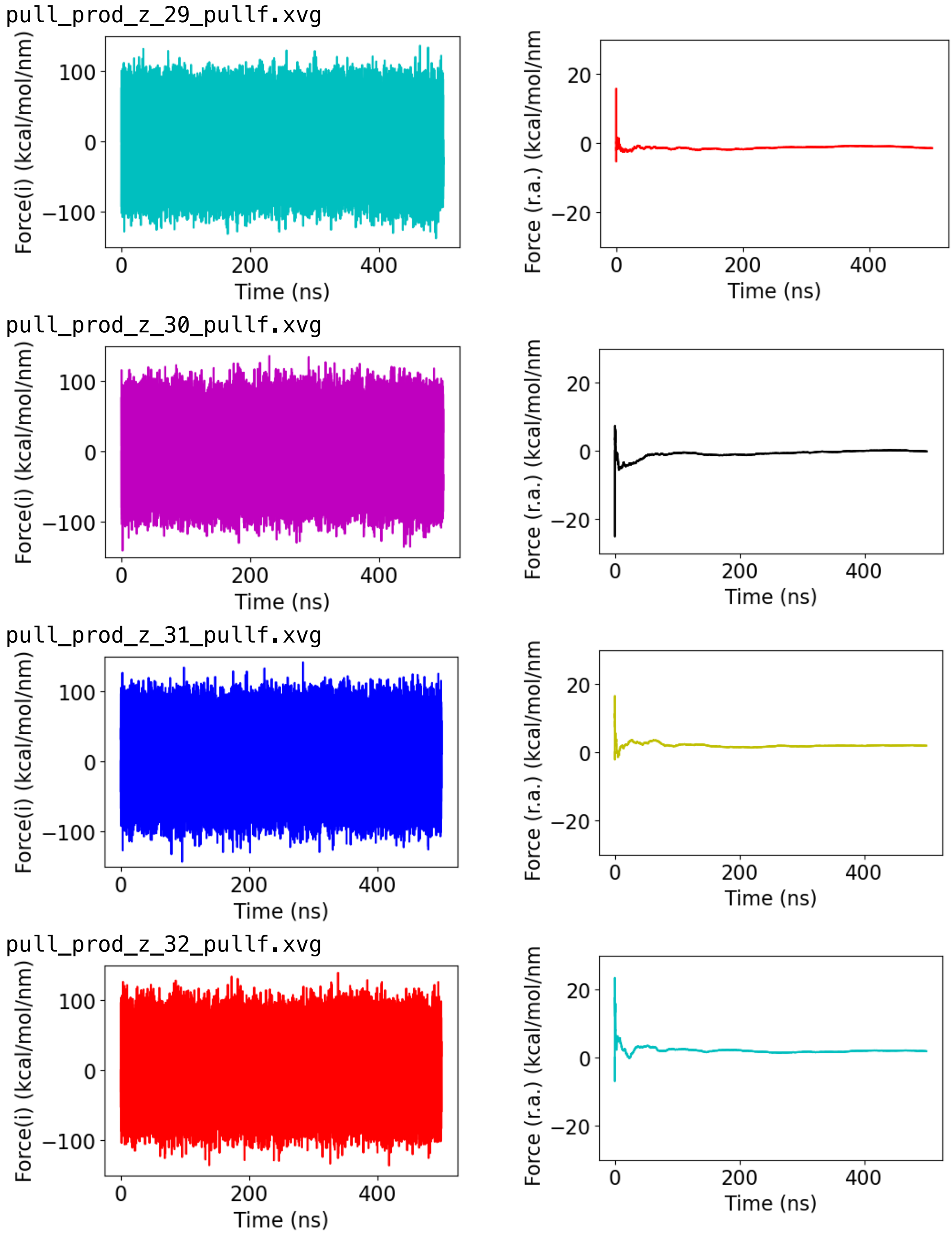
pull_prod_z_29_pullf.xvg
pull_prod_z_30_pullf.xvg
pull_prod_z_31_pullf.xvg
pull_prod_z_32_pullf.xvg
Force(i) (kcal/mol/nm)
Force (r.a.) (kcal/mol/nm
Time (ns)
100
0
−100
20
−20
200
400

pull_prod_z_33_pullf.xvg

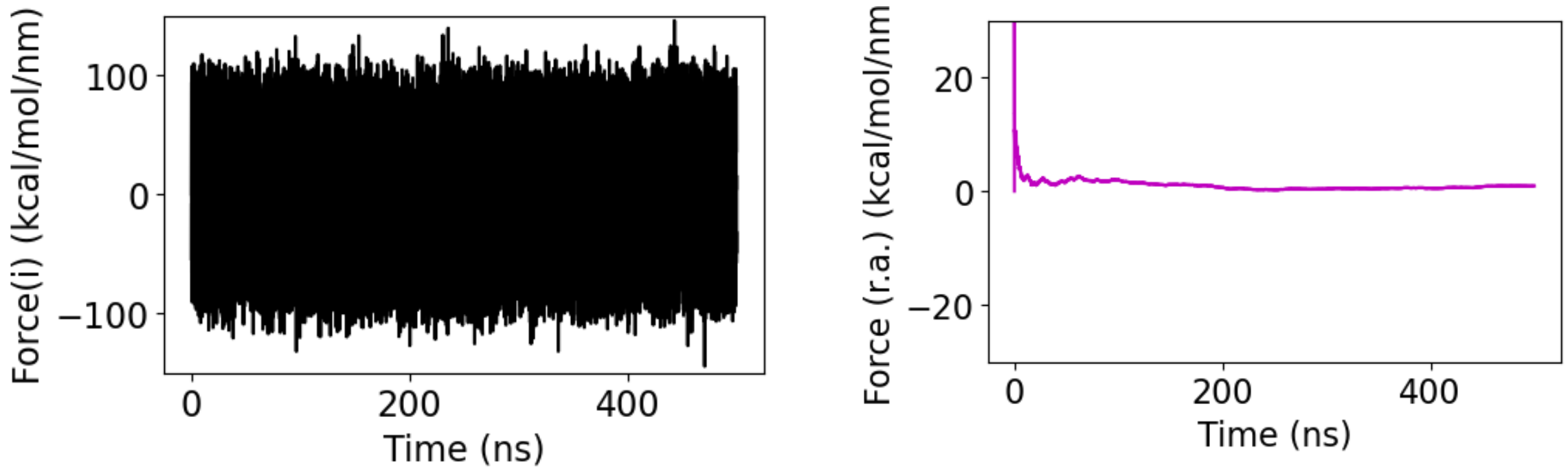

pull_prod_z_34_pullf.xvg

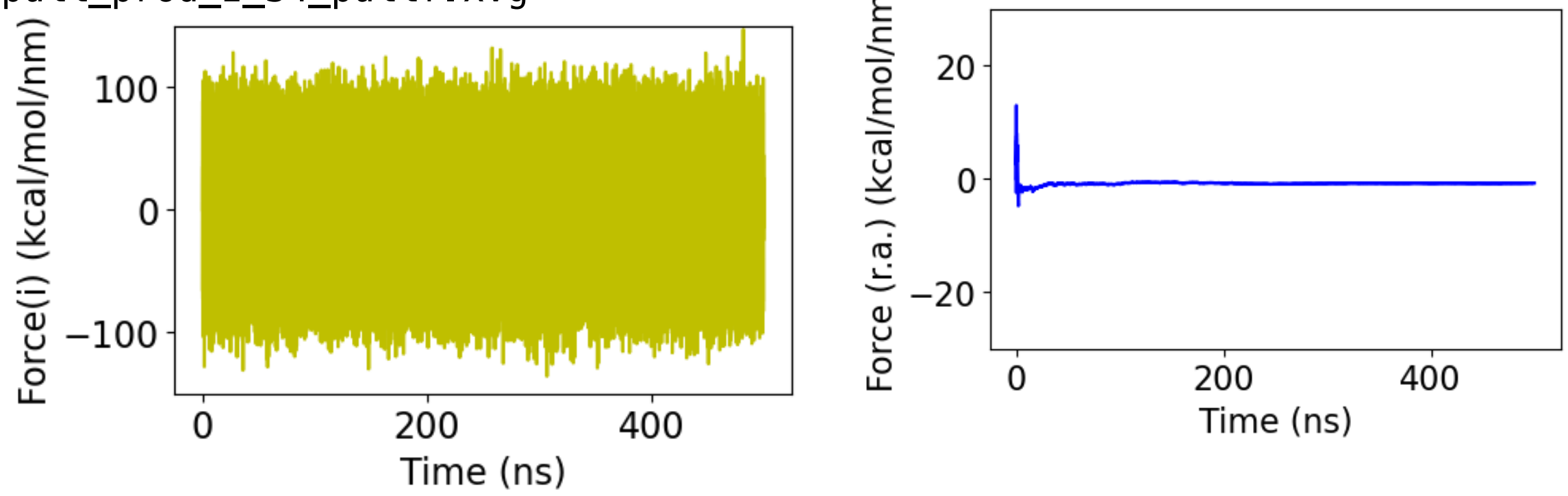

pull_prod_z_35_pullf.xvg

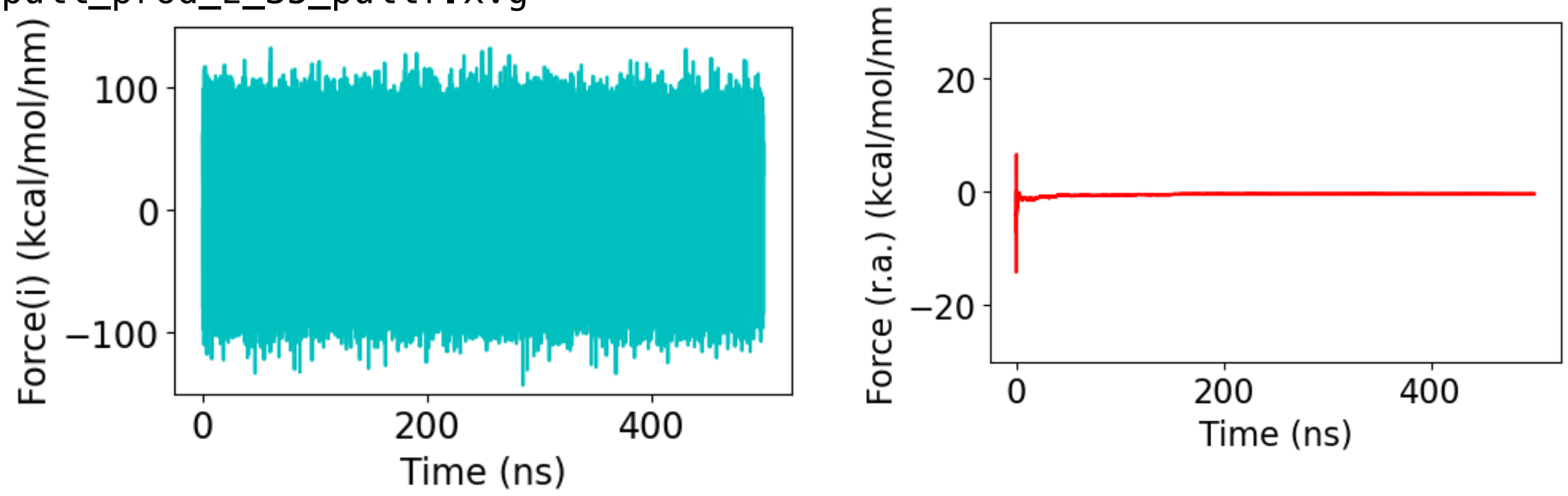

pull_prod_z_36_pullf.xvg

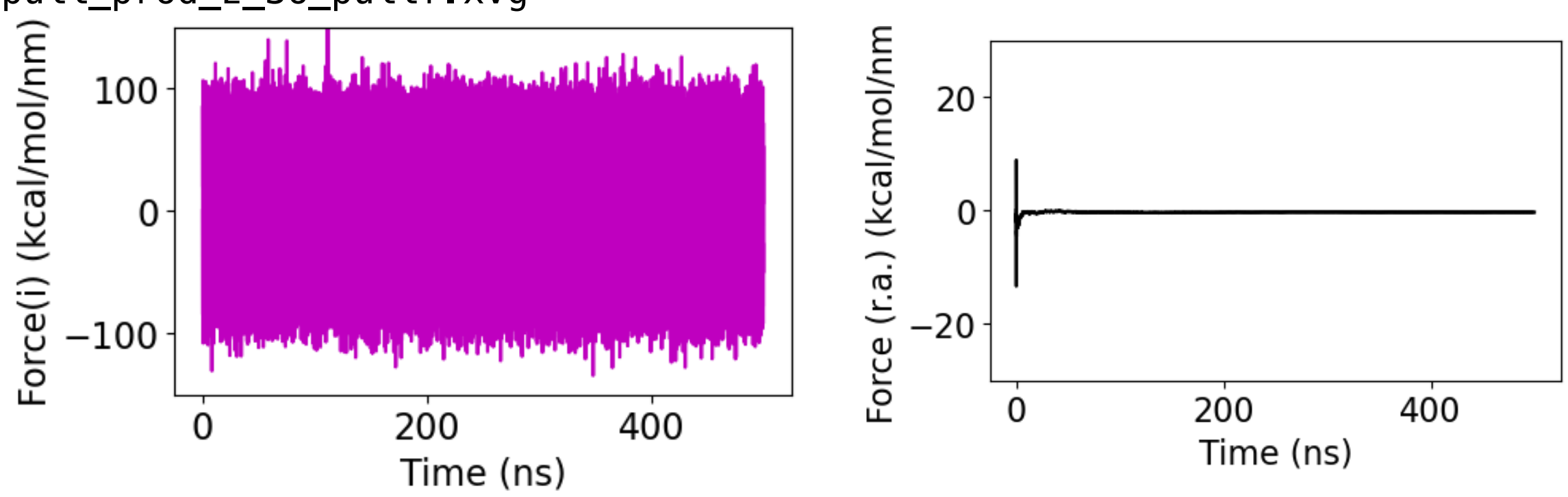

pull_prod_z_37_pullf.xvg

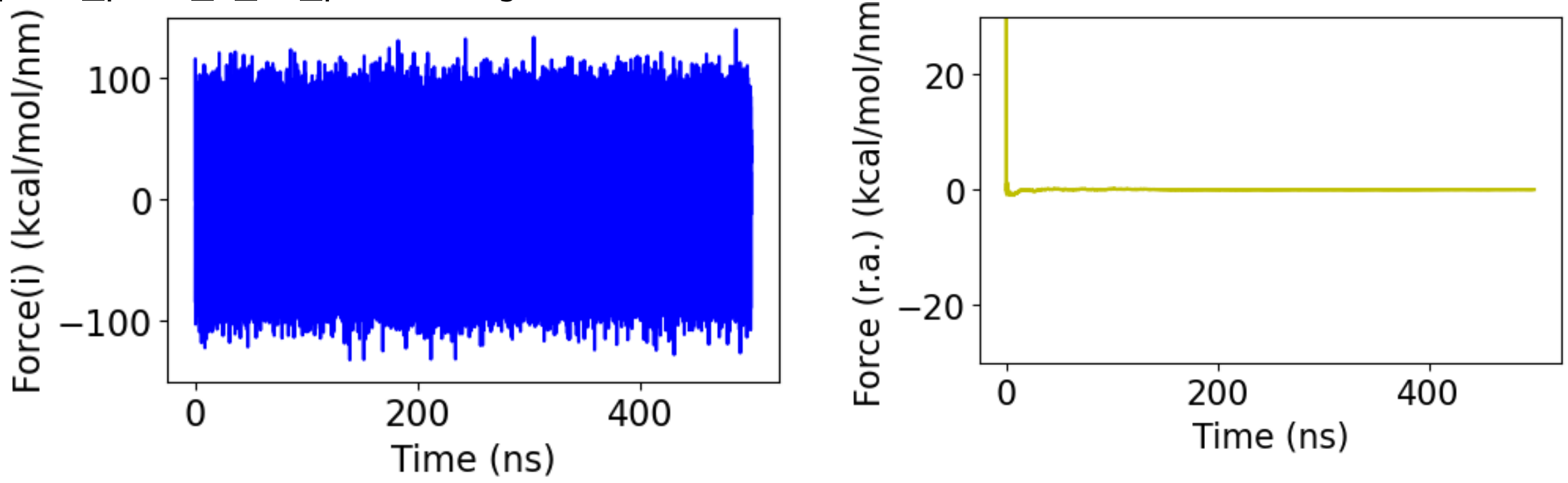

pull_prod_z_38_pullf.xvg

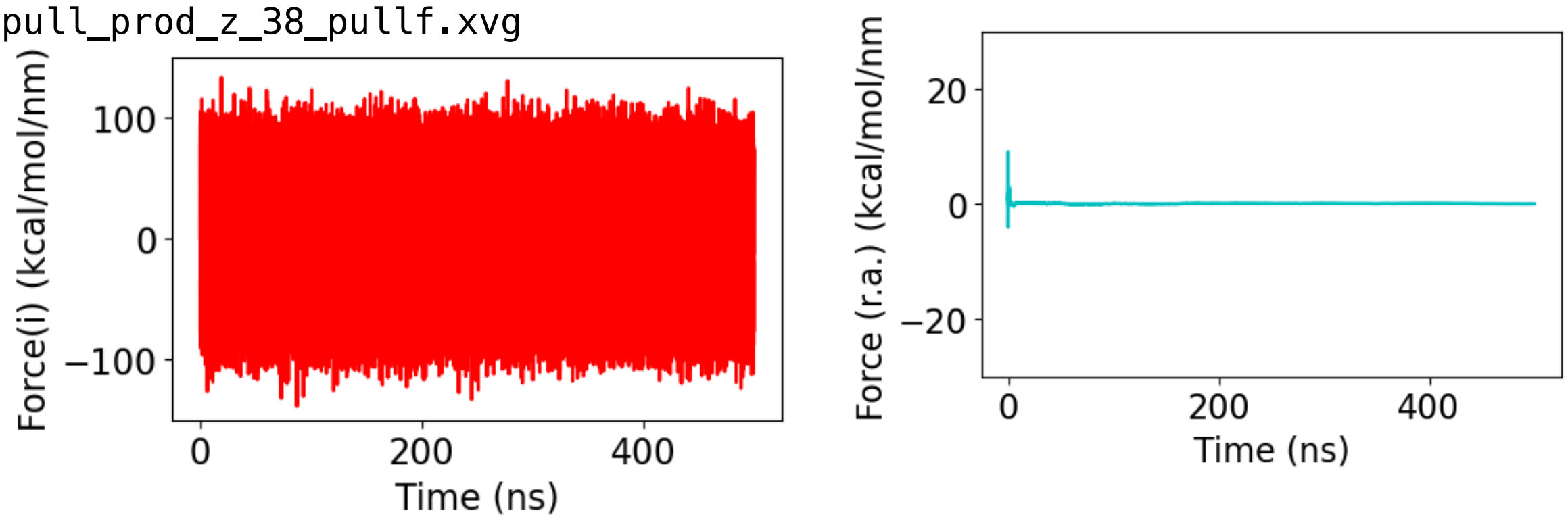

pull_prod_z_39_pullf.xvg

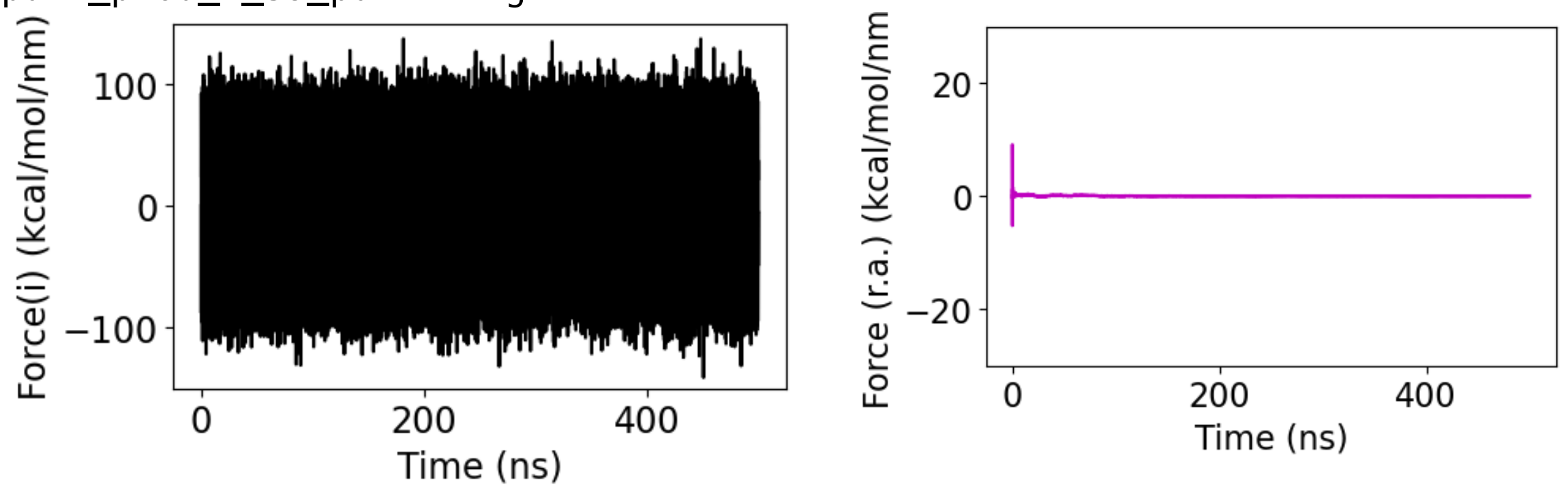

Force time series for temozolomide:

pull_prod_z_0_pullf.xvg

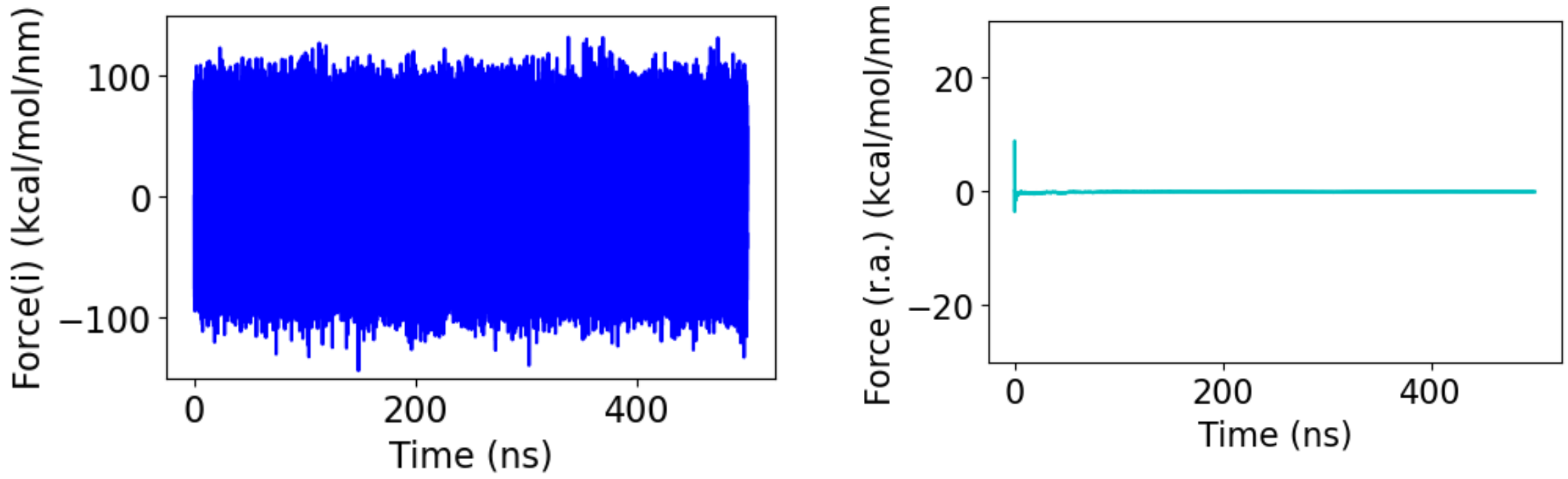

pull_prod_z_1_pullf.xvg

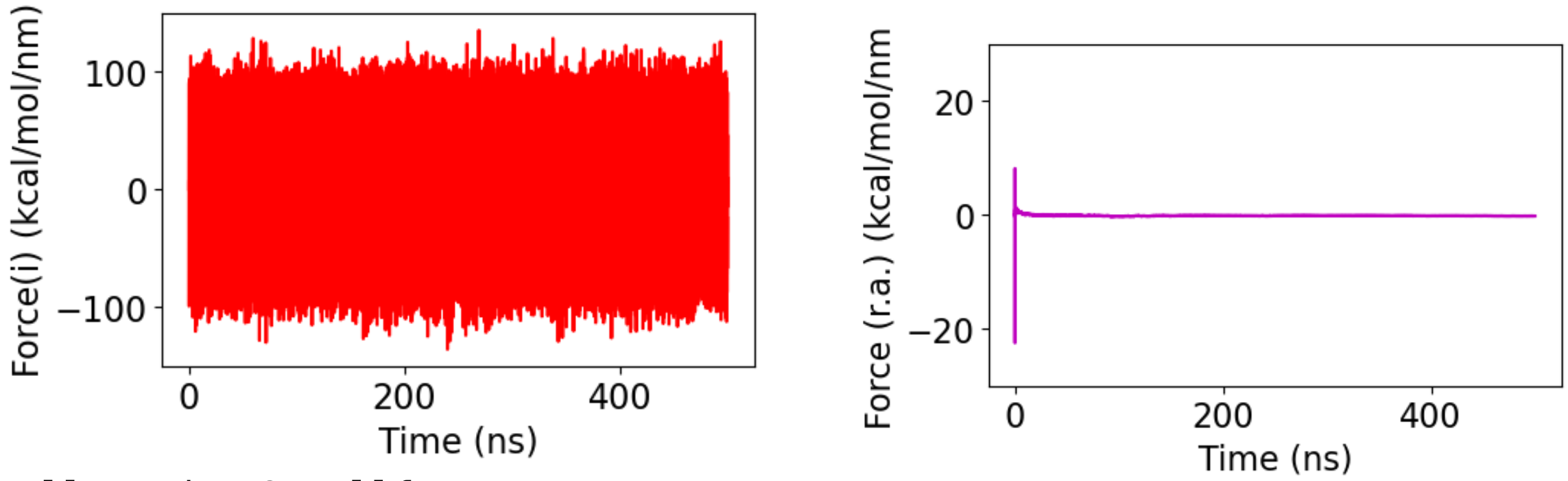

pull_prod_z_2_pullf.xvg

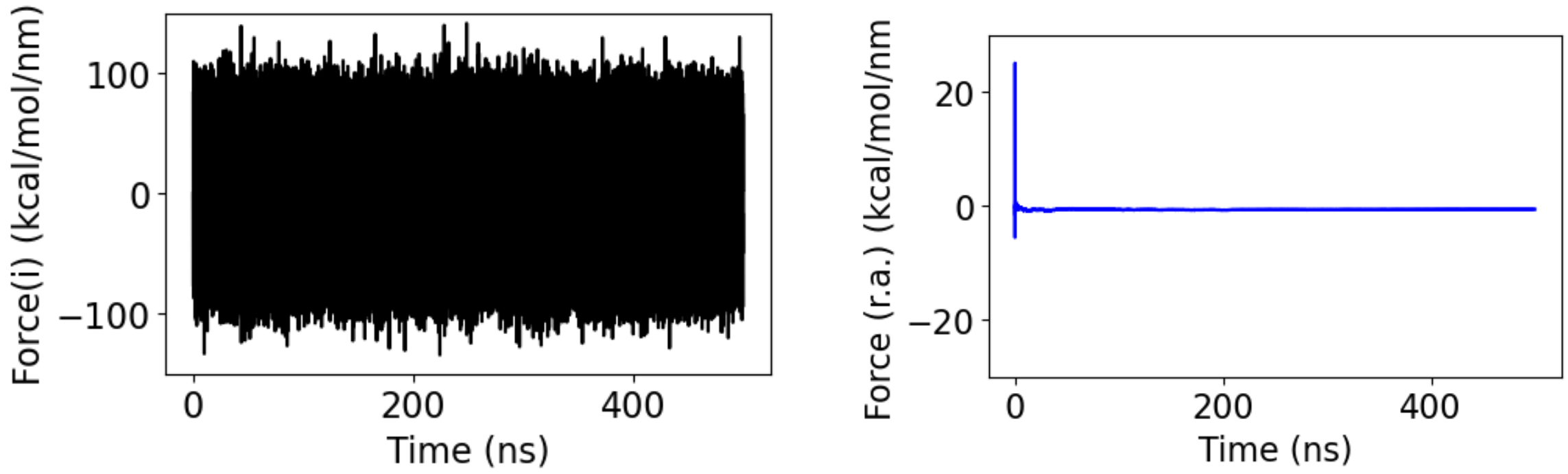

pull_prod_z_3_pullf.xvg

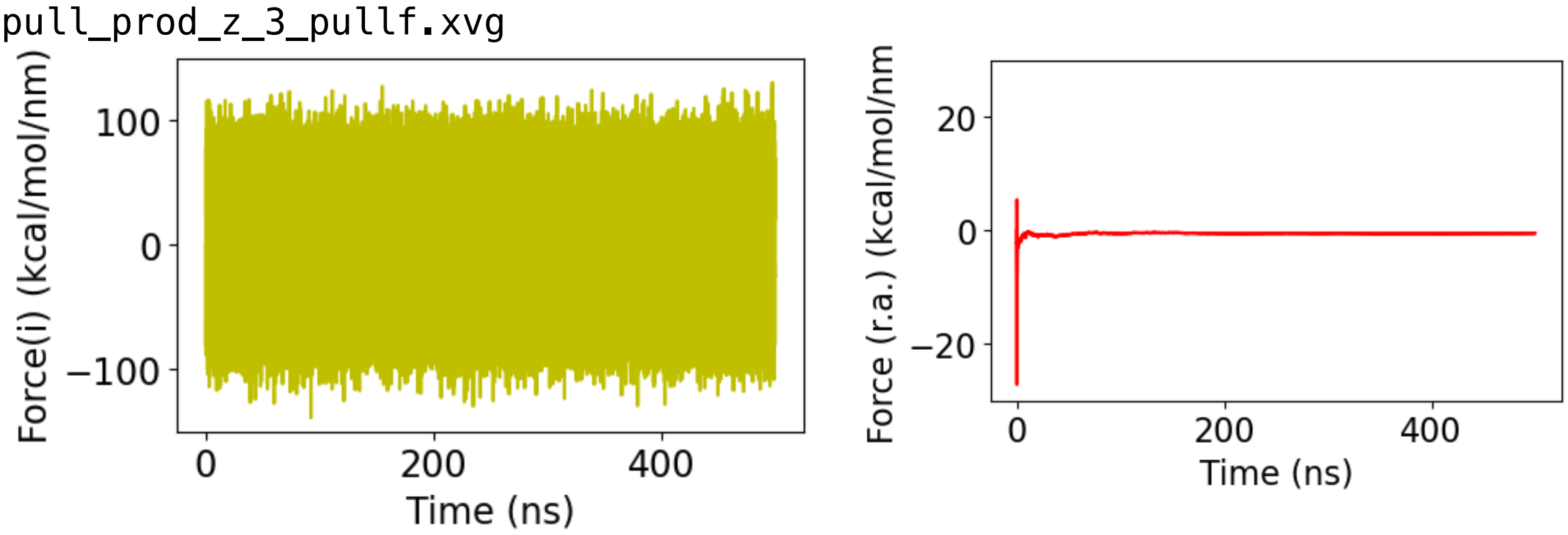

pull_prod_z_4_pullf.xvg

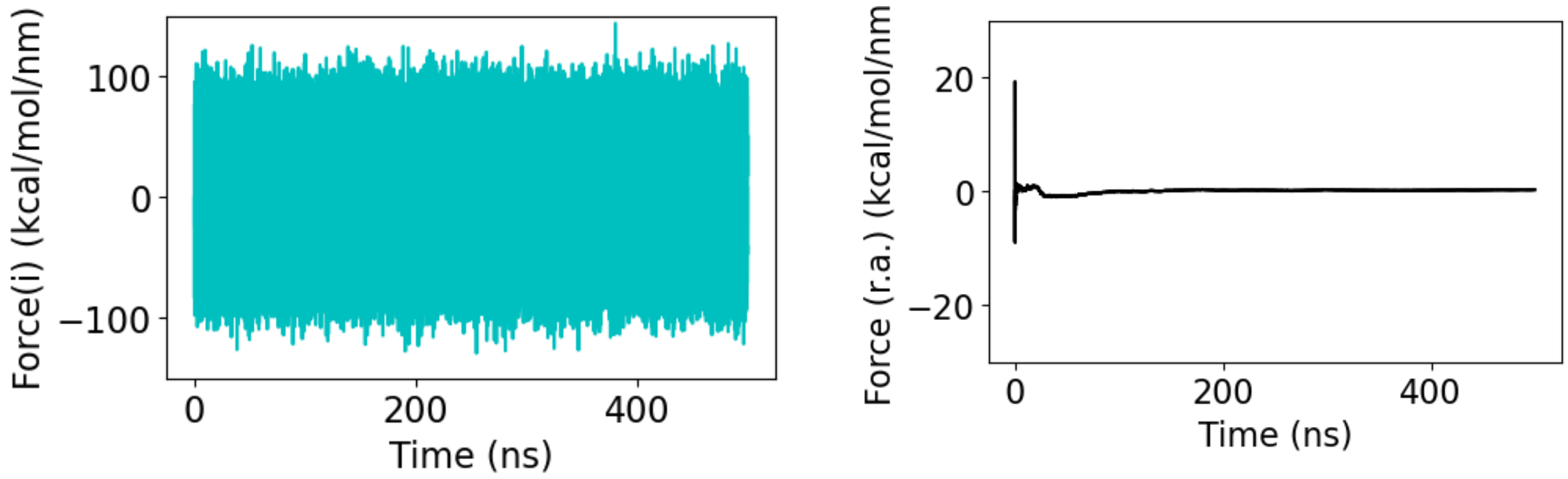

pull_prod_z_5_pullf.xvg

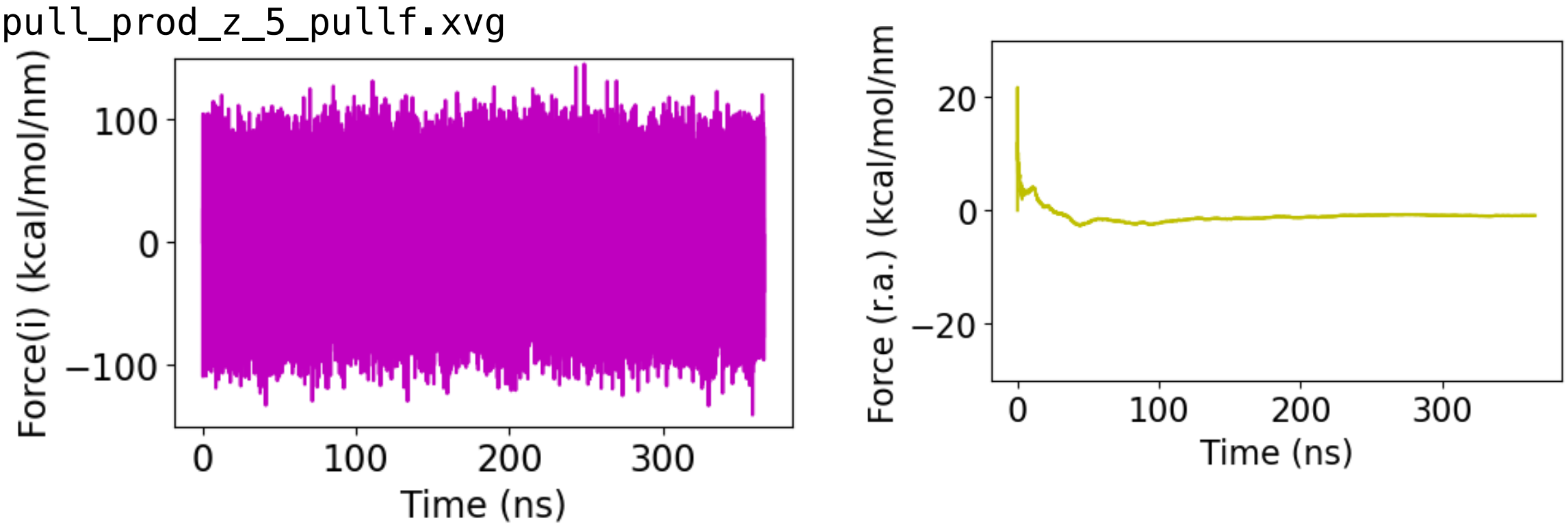

pull_prod_z_6_pullf.xvg

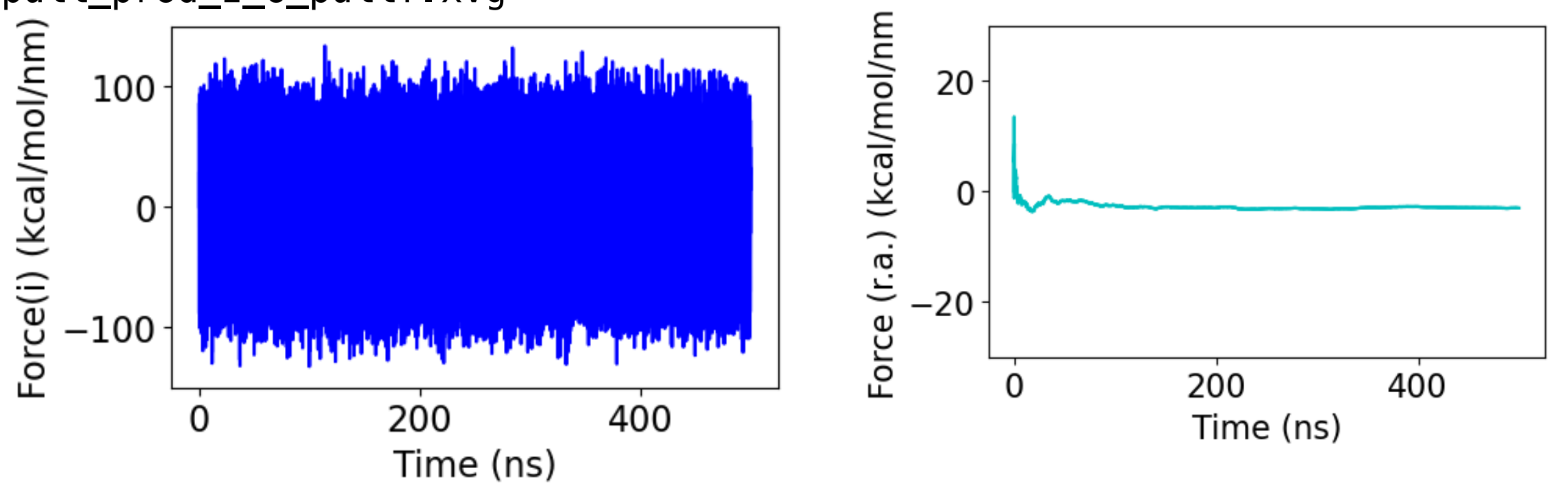

pull_prod_z_7_pullf.xvg

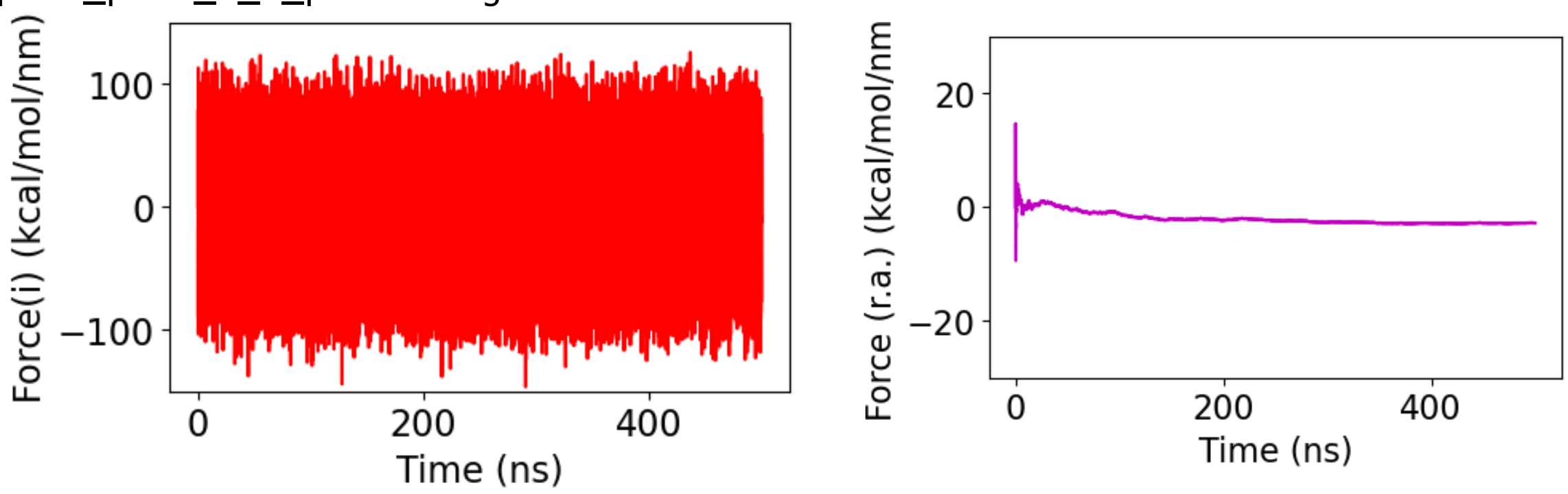

pull_prod_z_8_pullf.xvg

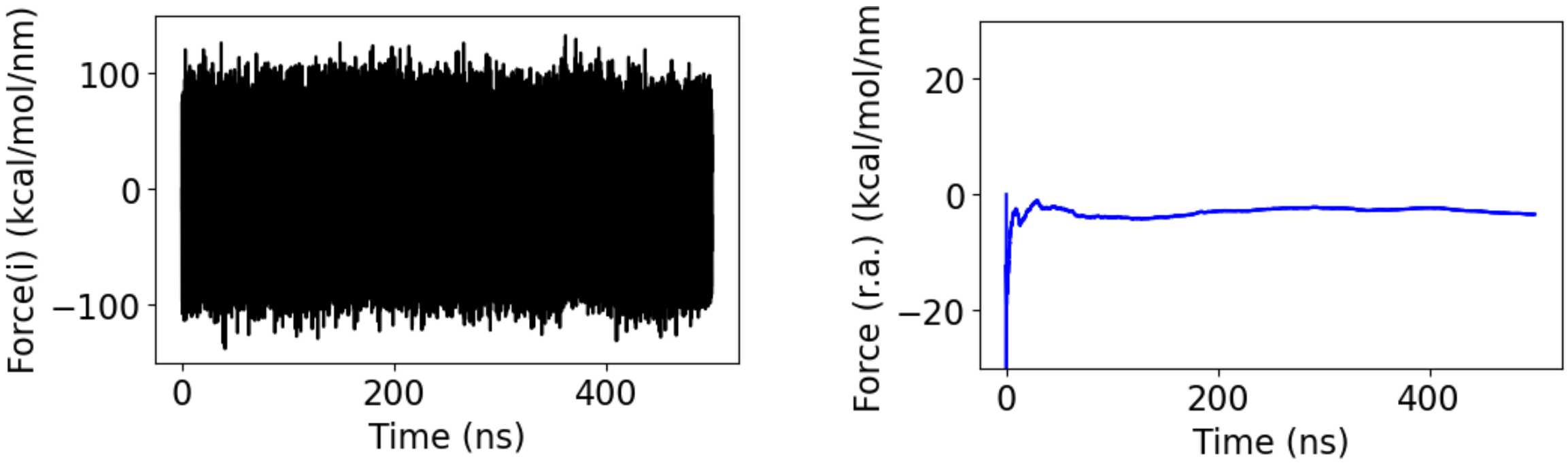

pull_prod_z_9_pullf.xvg

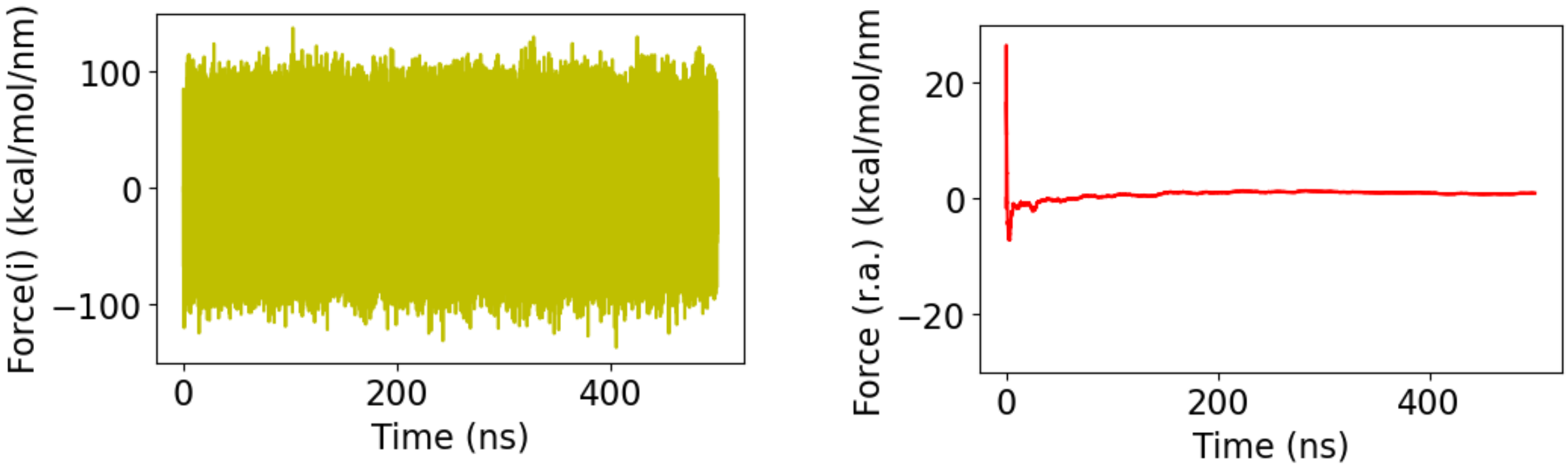

pull_prod_z_10_pullf.xvg

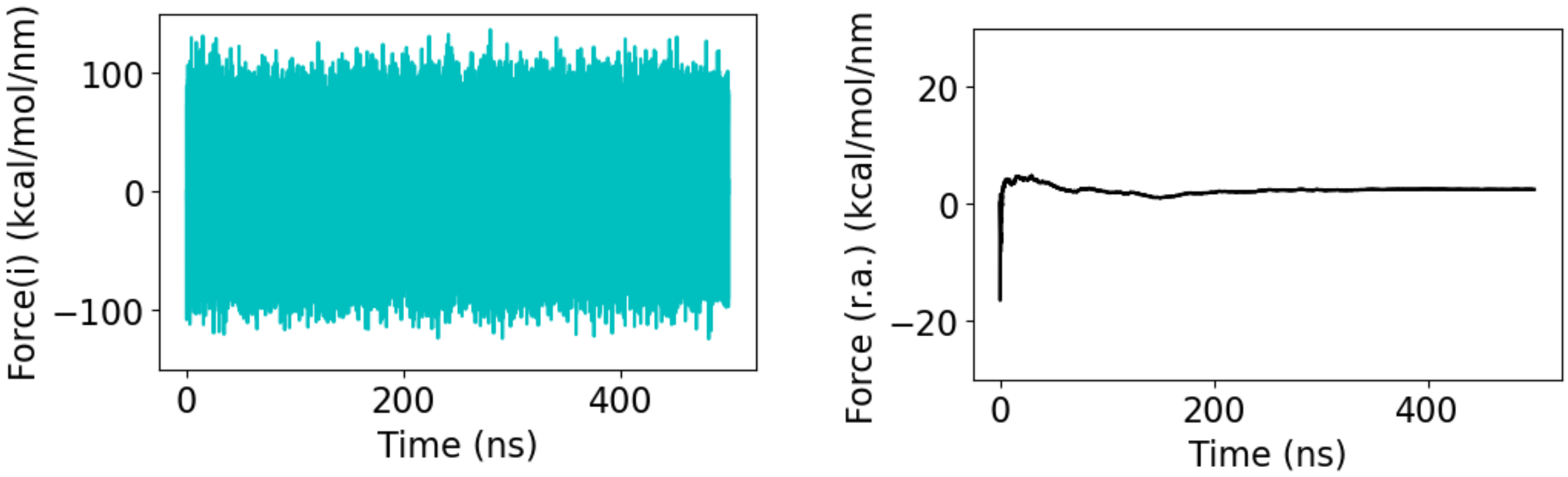

pull_prod_z_11_pullf.xvg

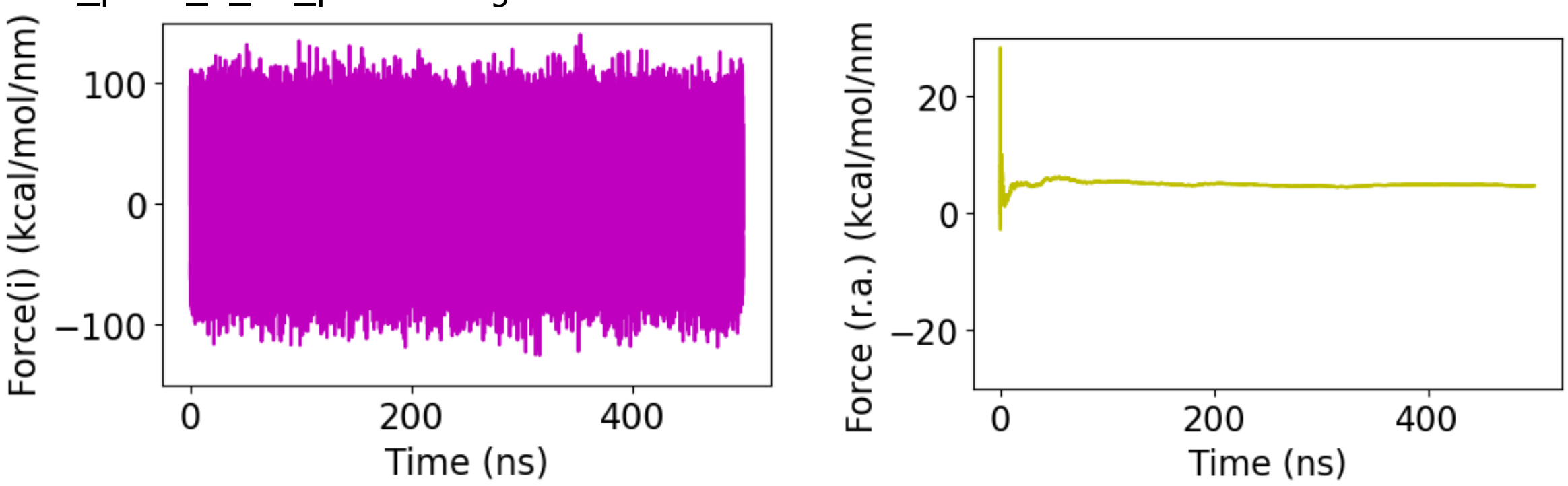

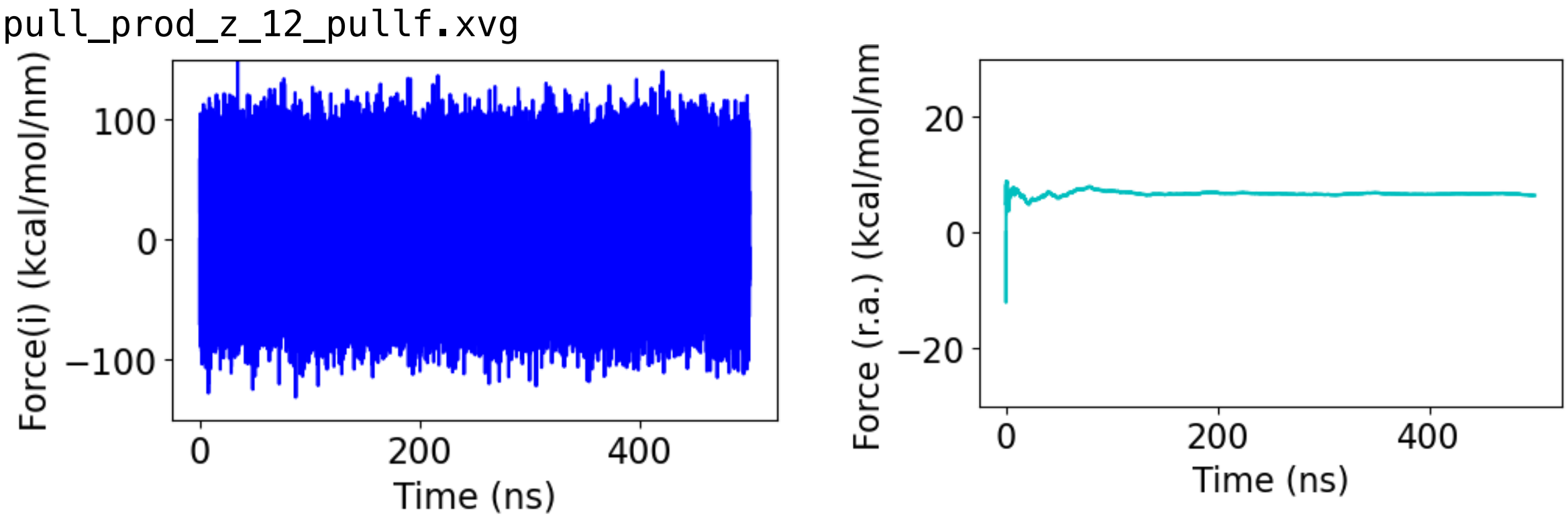
pull_prod_z_12_pullf.xvg
Force(i) (kcal/mol/nm)
Time (ns)
Force (r.a.) (kcal/mol/nm
Time (ns)

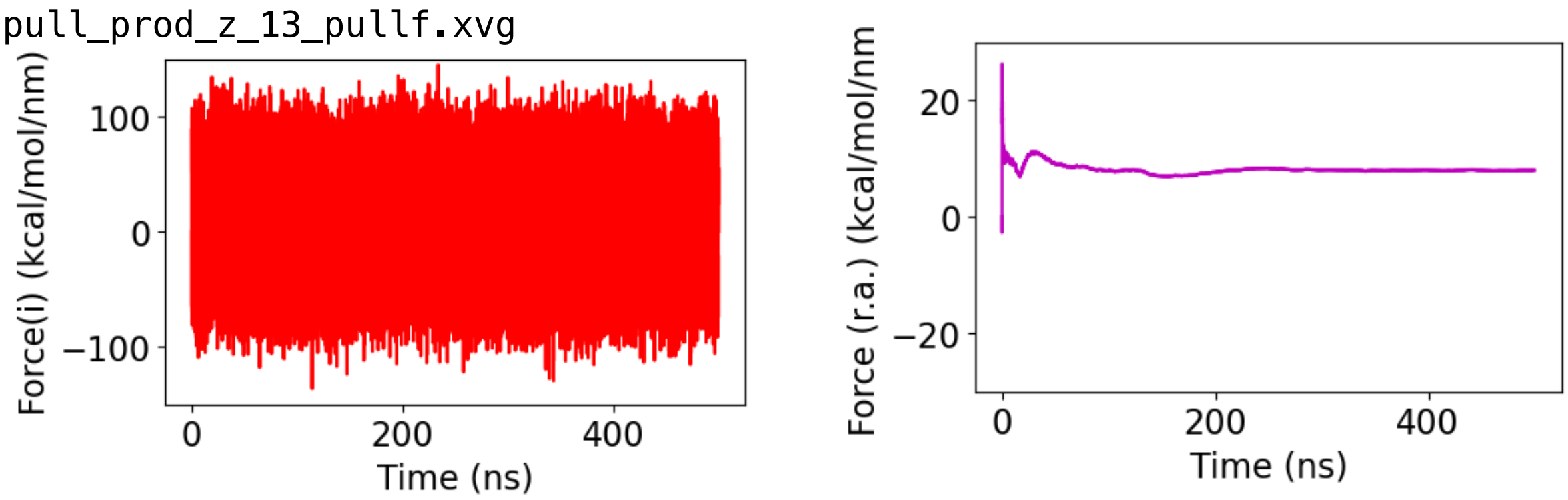
pull_prod_z_13_pullf.xvg
Force(i) (kcal/mol/nm)
Time (ns)
Force (r.a.) (kcal/mol/nm
Time (ns)

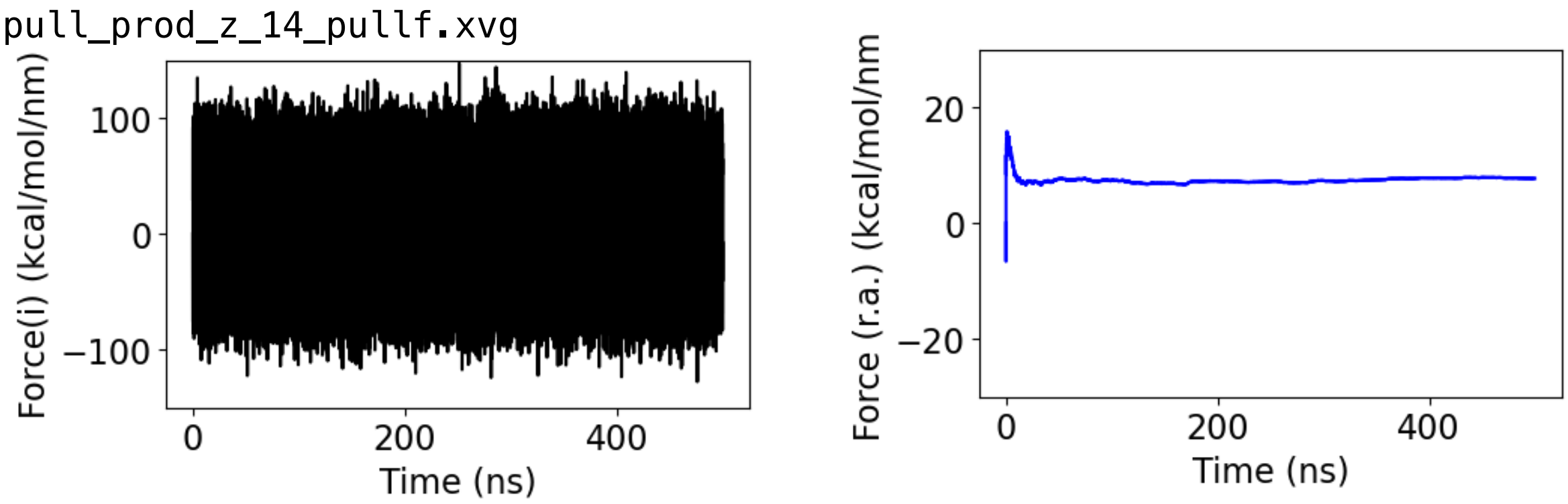
pull_prod_z_14_pullf.xvg
Force(i) (kcal/mol/nm)
Time (ns)
Force (r.a.) (kcal/mol/nm
Time (ns)

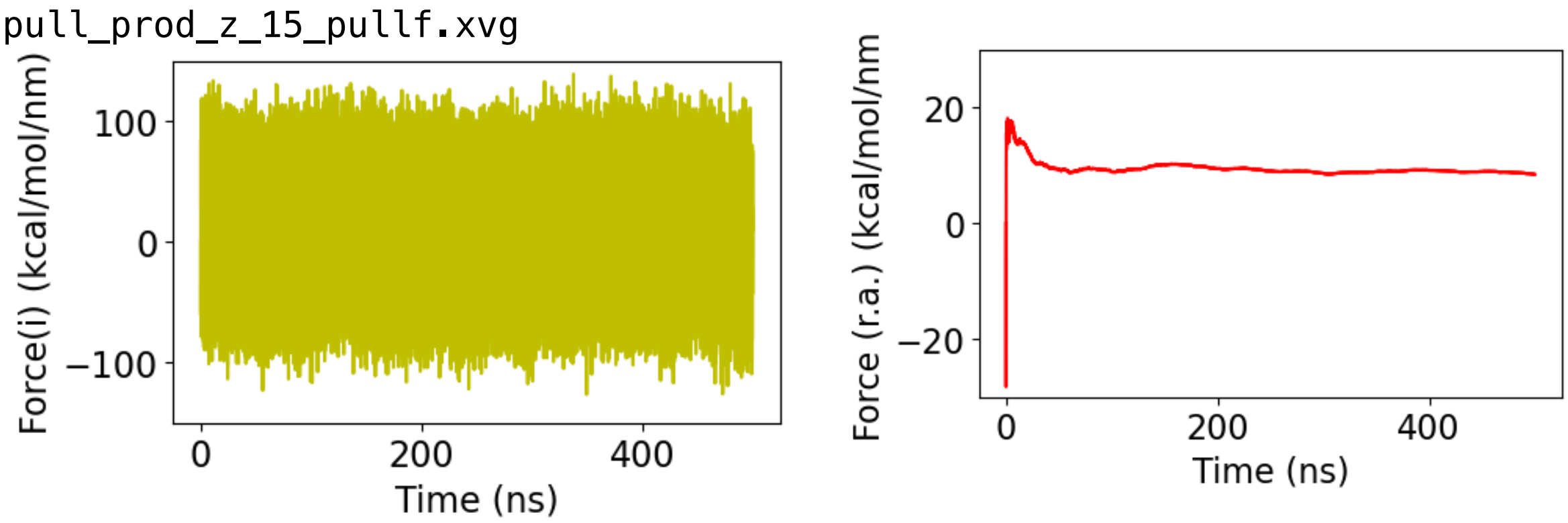
pull_prod_z_15_pullf.xvg
Force(i) (kcal/mol/nm)
Time (ns)
Force (r.a.) (kcal/mol/nm
Time (ns)

pull_prod_z_16_pullf.xvg

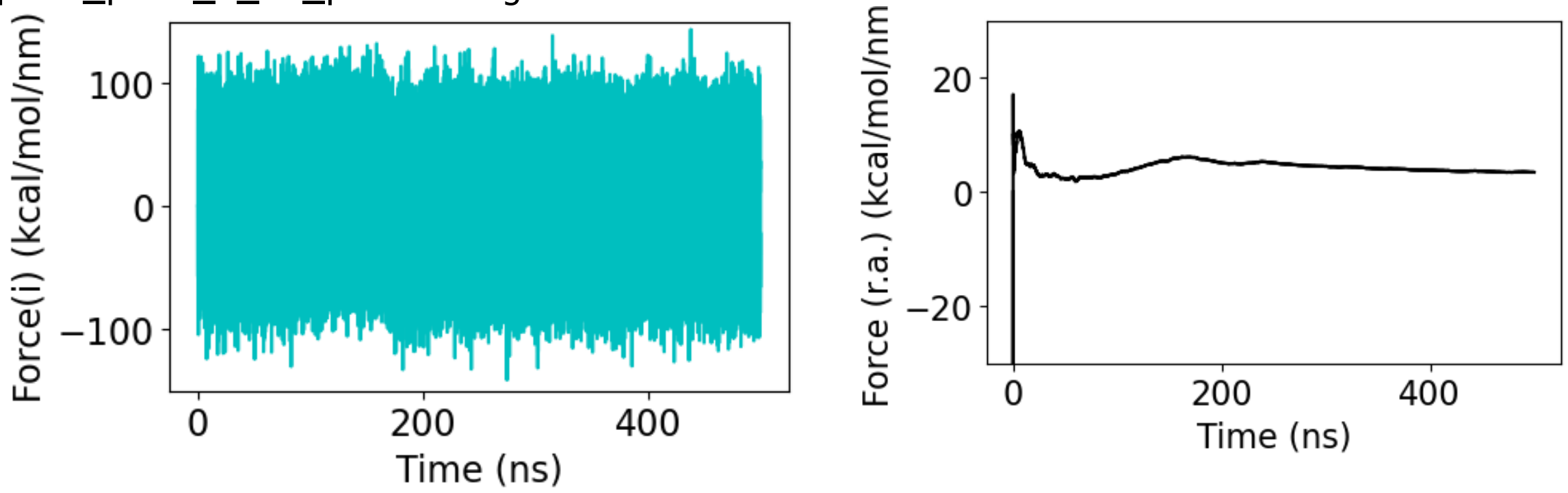

pull_prod_z_17_pullf.xvg

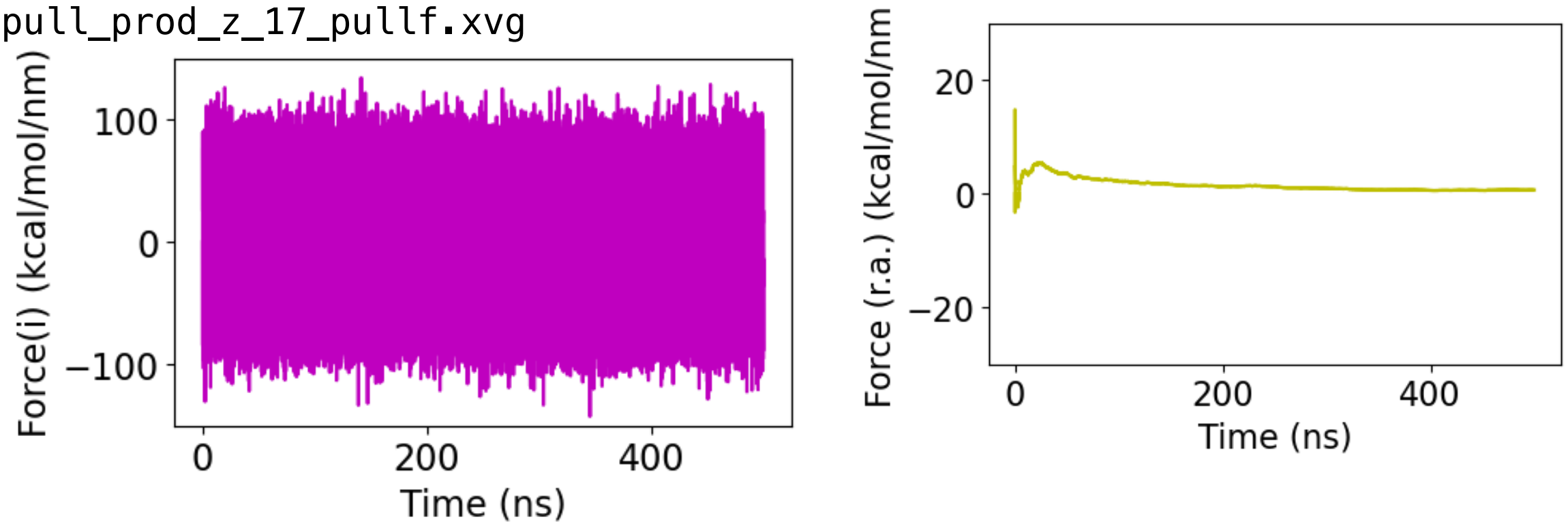

pull_prod_z_18_pullf.xvg

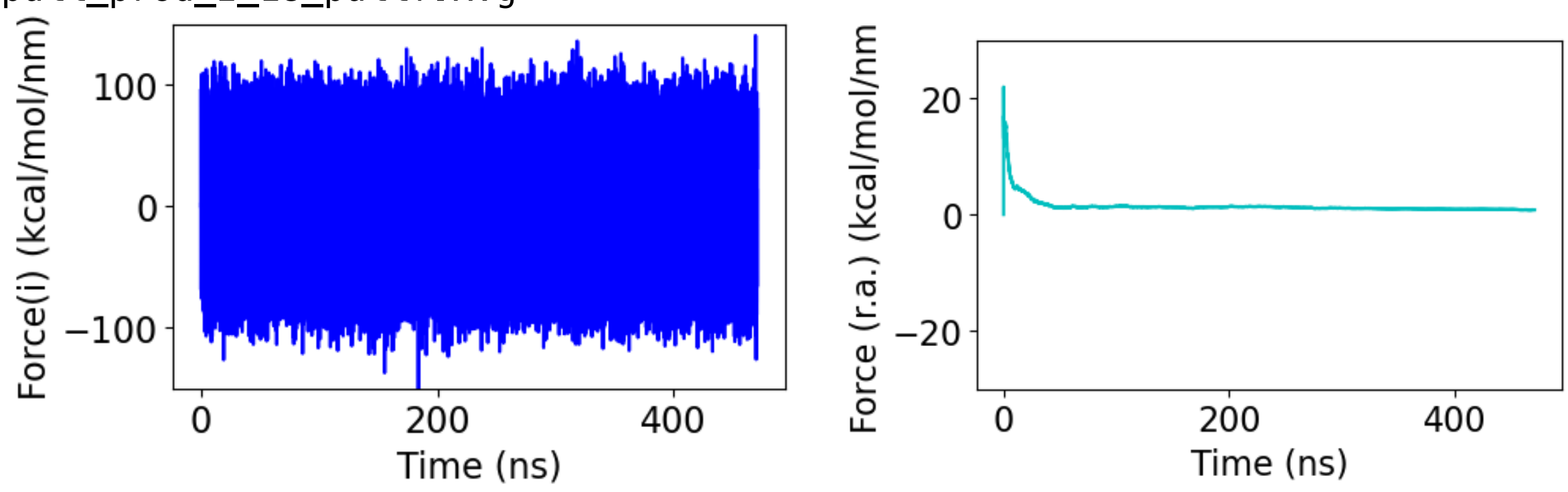

pull_prod_z_19_pullf.xvg

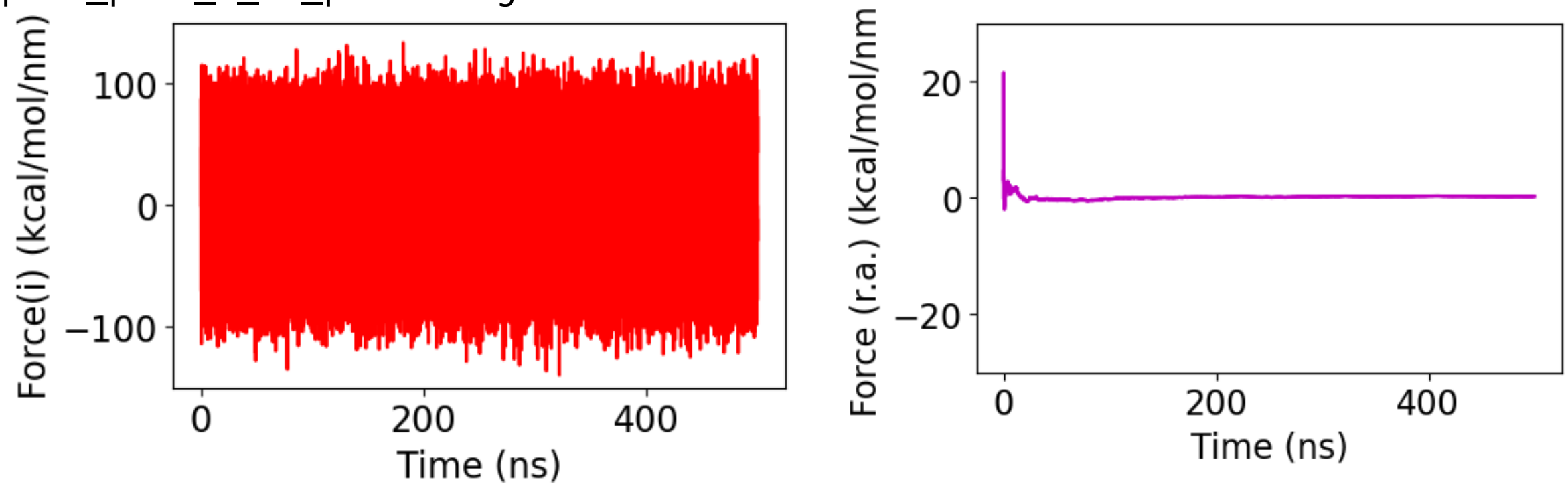

pull_prod_z_20_pullf.xvg

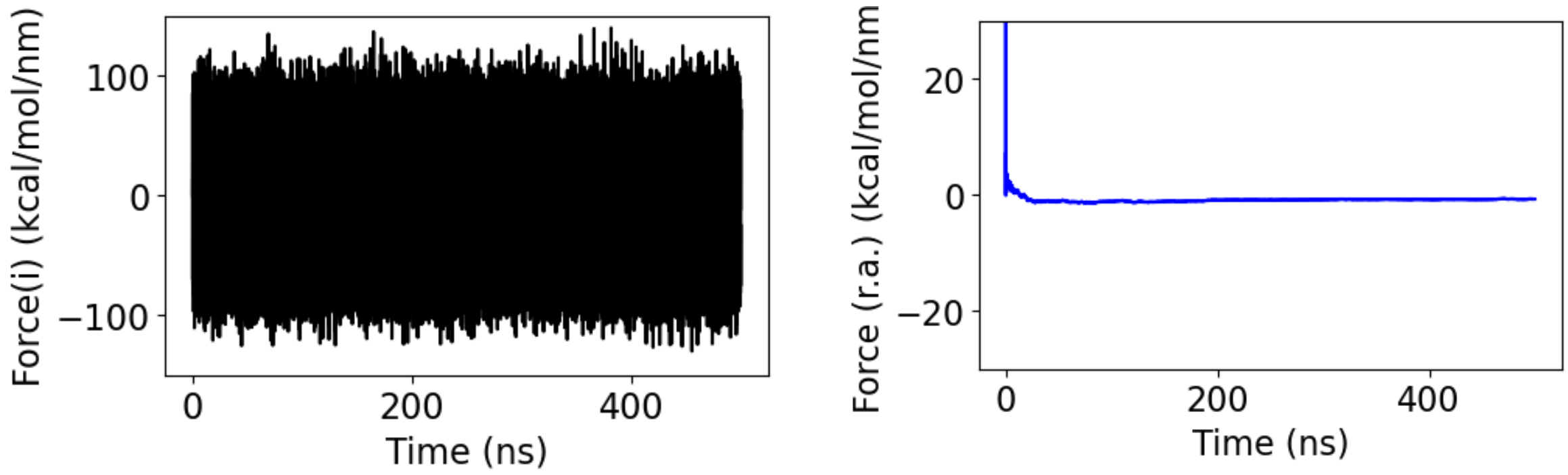

pull_prod_z_21_pullf.xvg

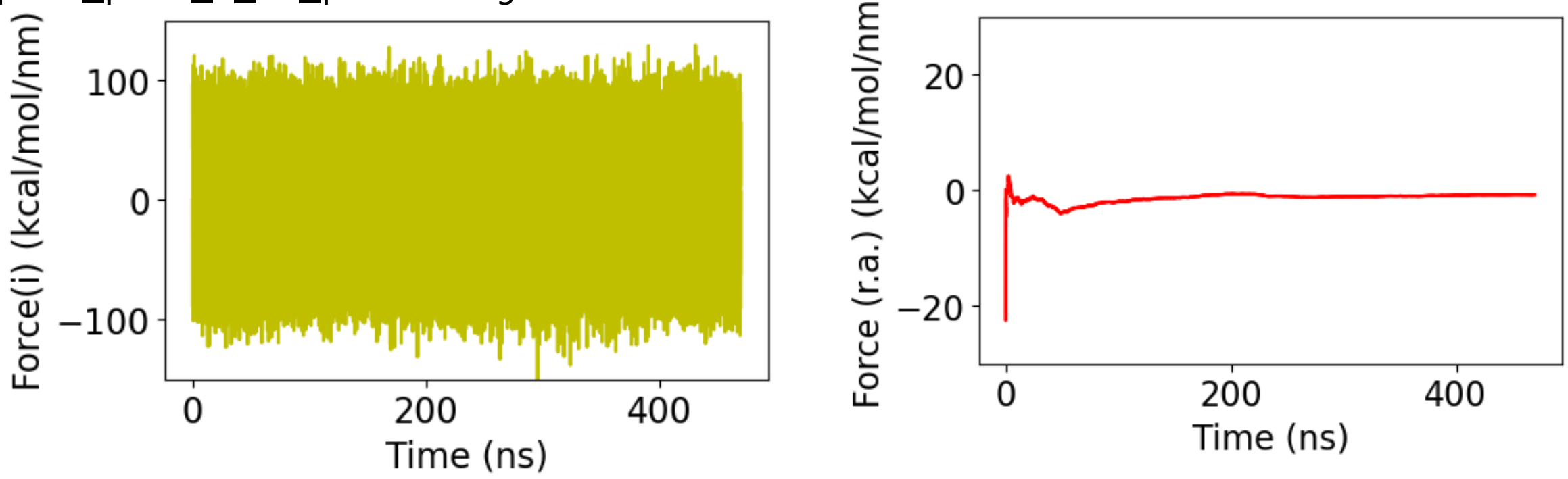

pull_prod_z_22_pullf.xvg

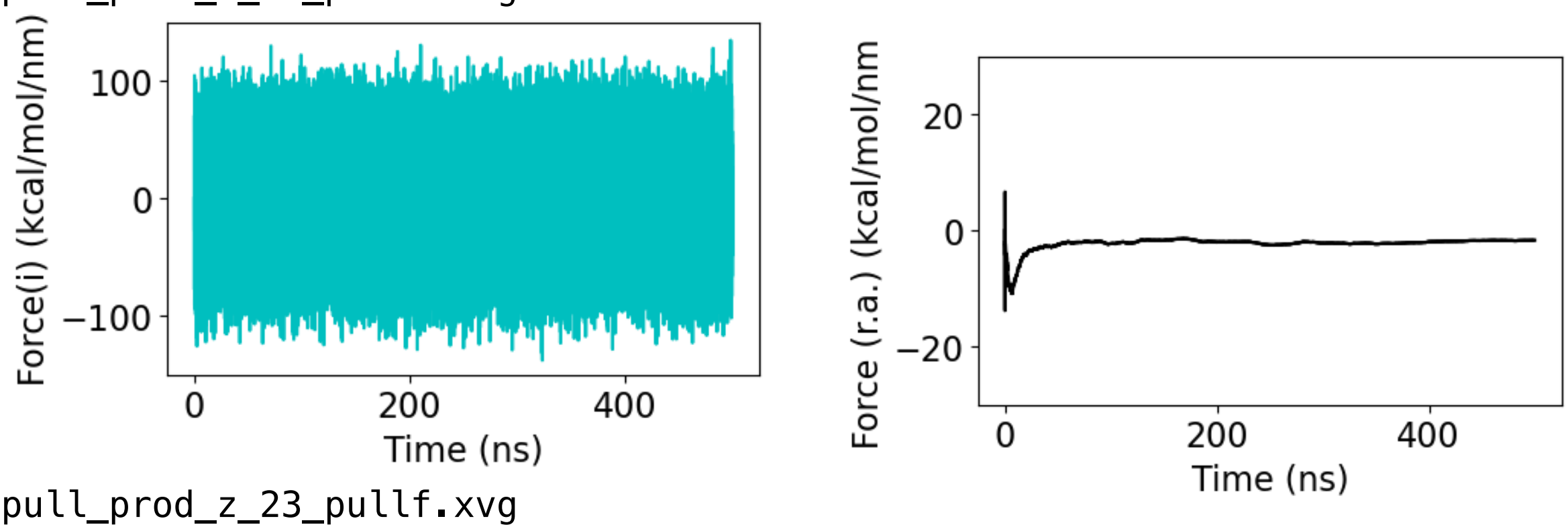

pull_prod_z_23_pullf.xvg

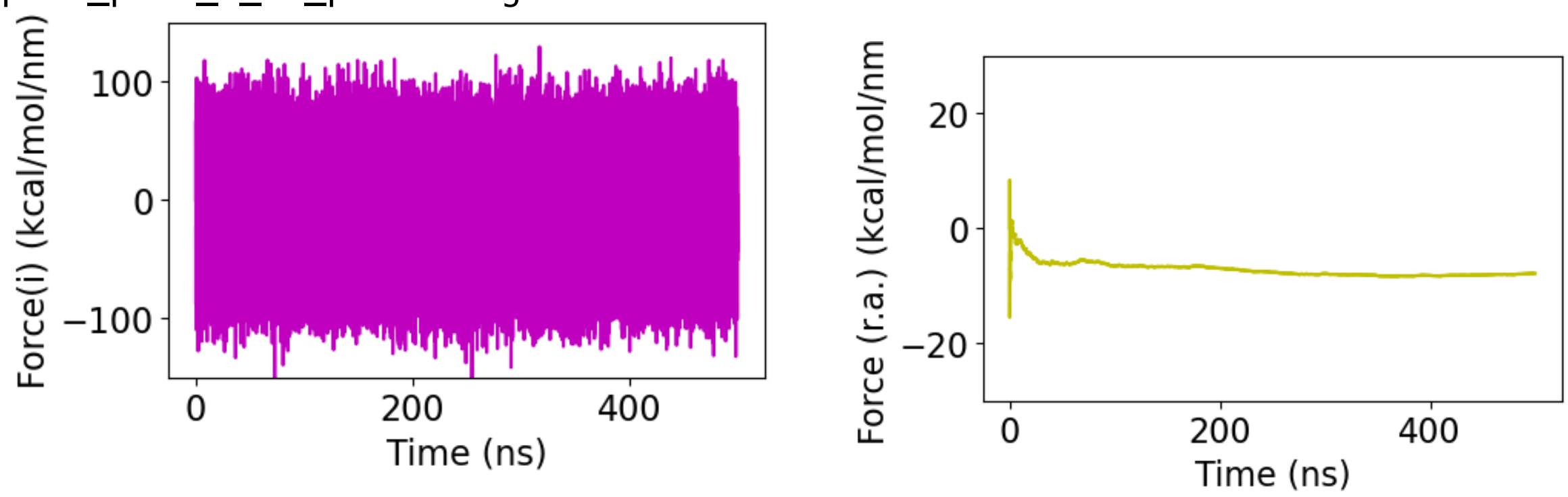

pull_prod_z_24_pullf.xvg

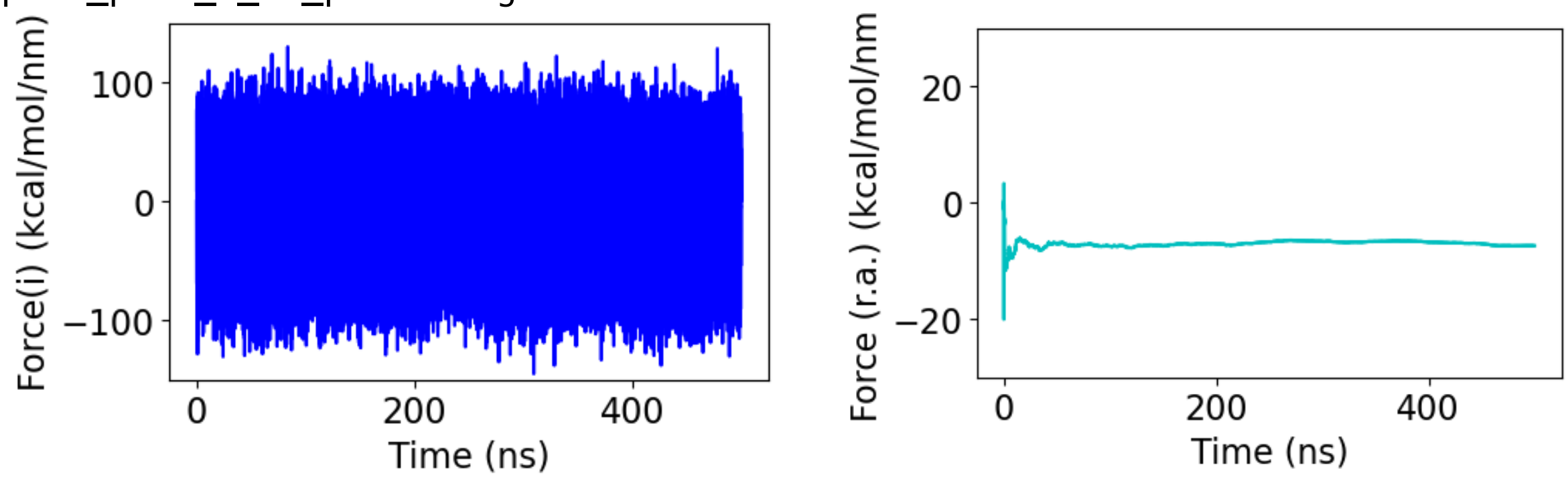

pull_prod_z_25_pullf.xvg

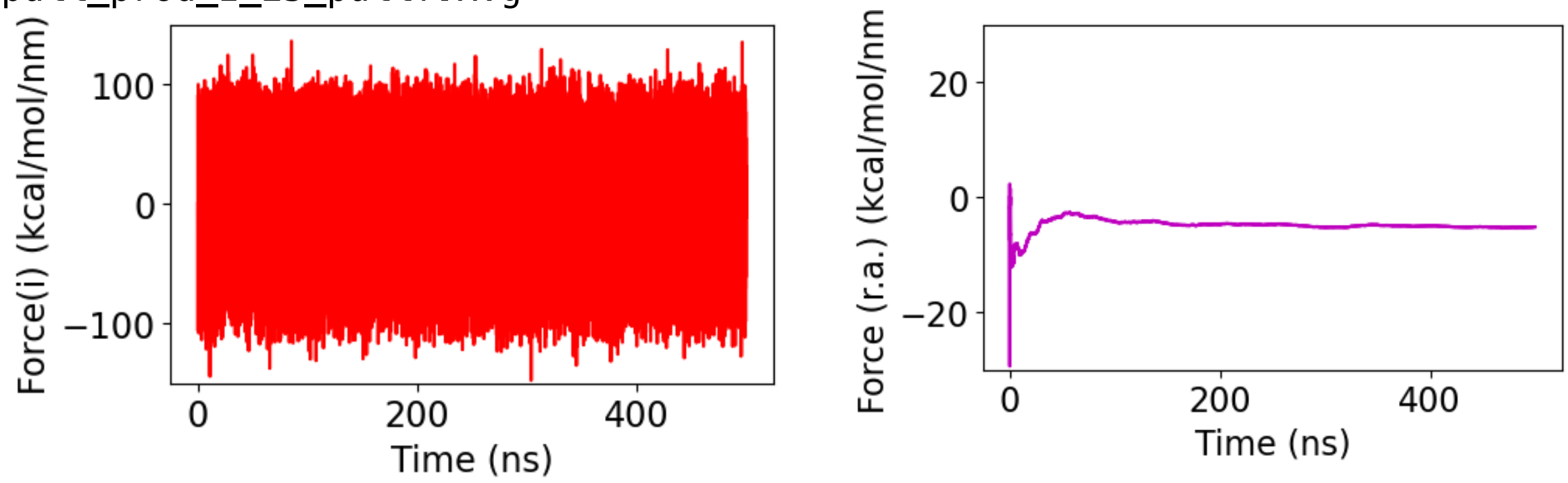

pull_prod_z_26_pullf.xvg

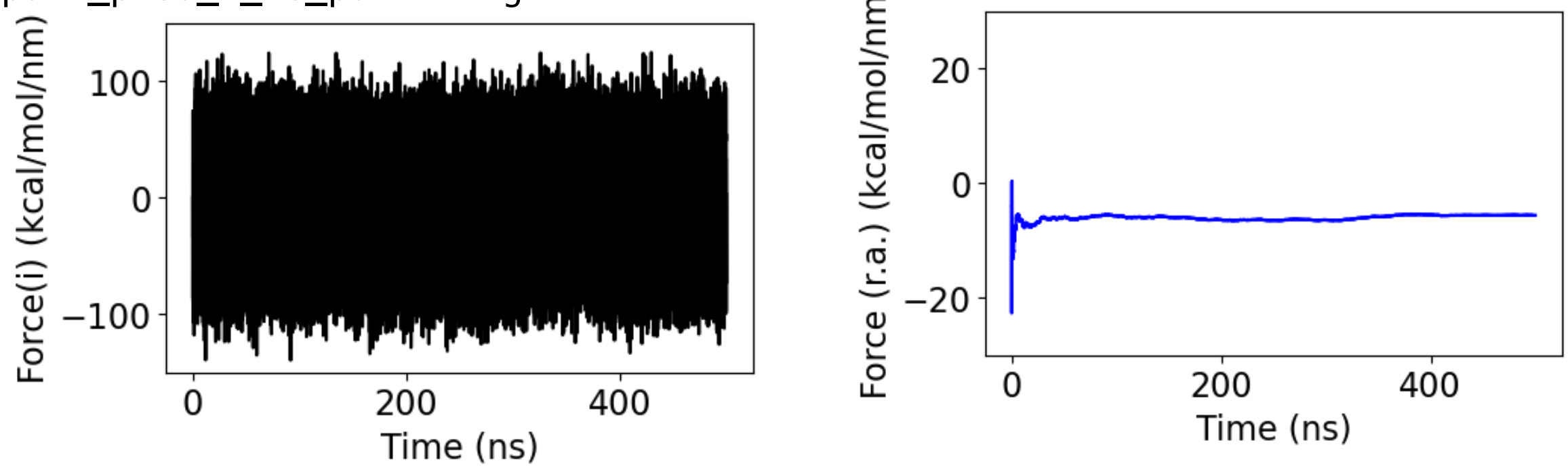

pull_prod_z_27_pullf.xvg

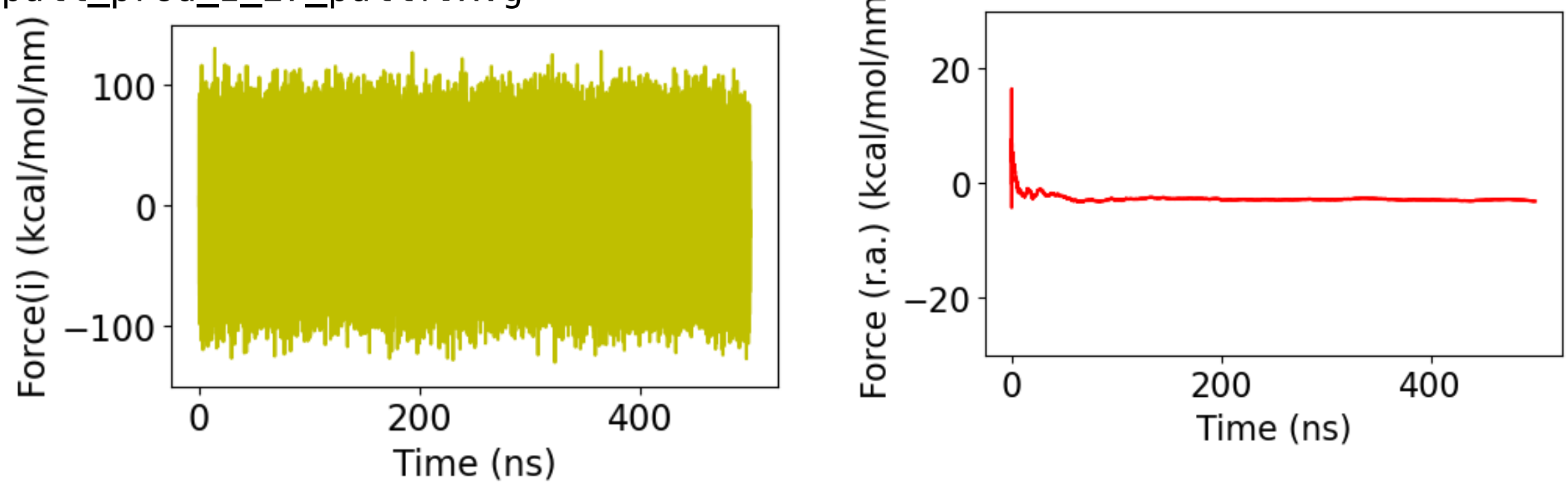

pull_prod_z_28_pullf.xvg

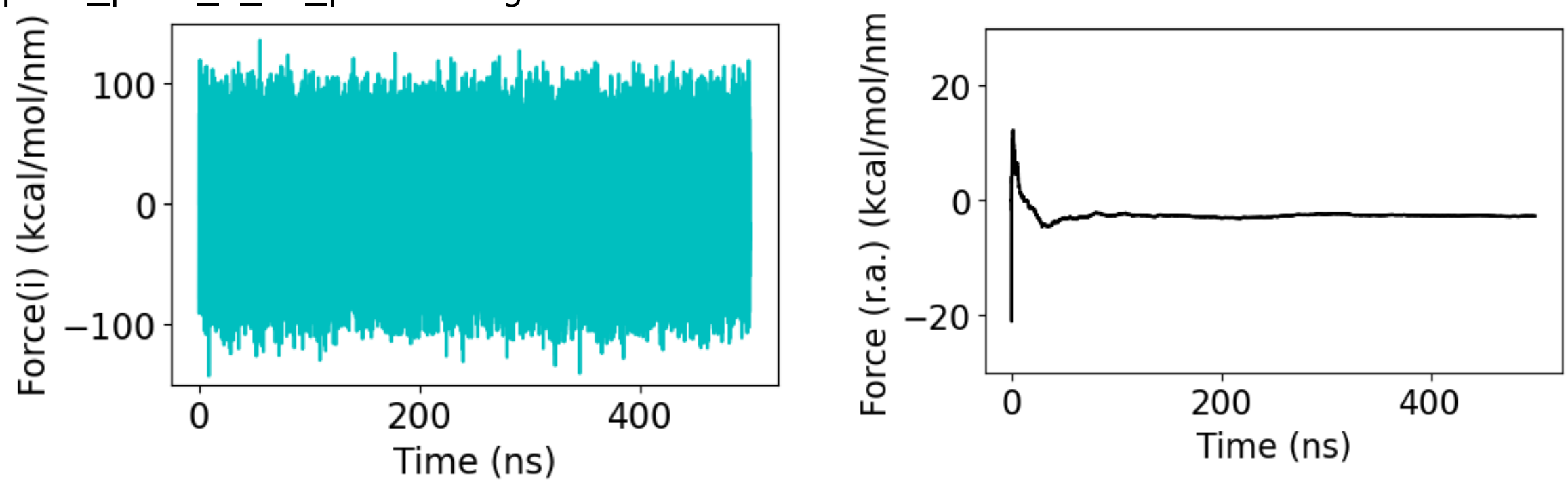

pull_prod_z_29_pullf.xvg

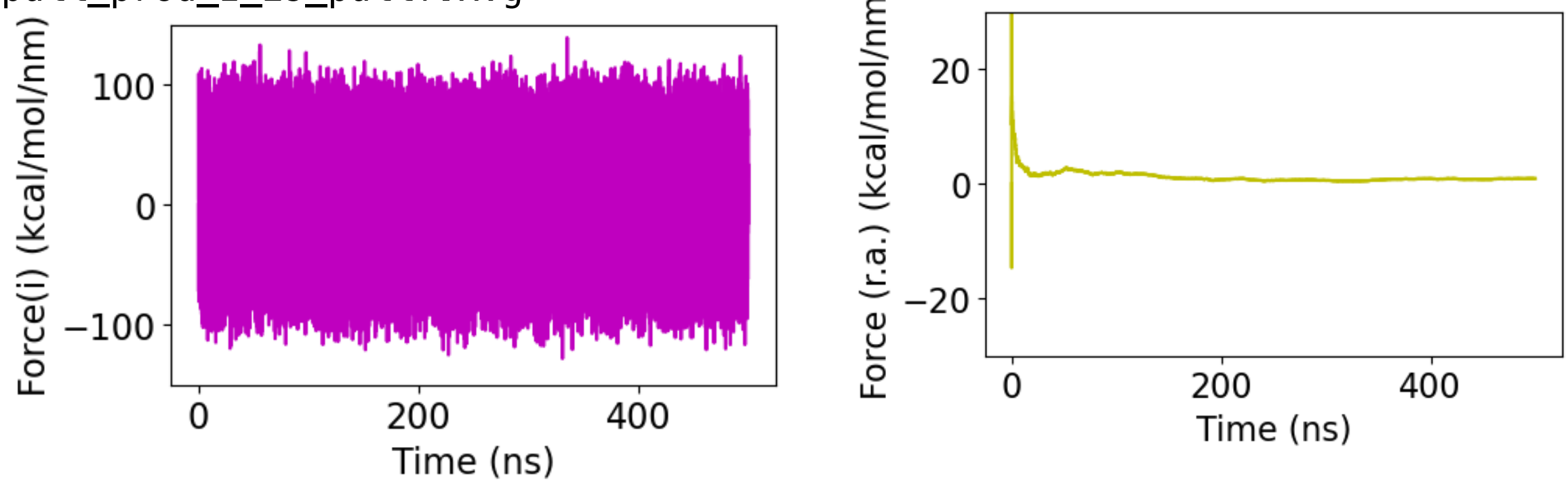

pull_prod_z_30_pullf.xvg

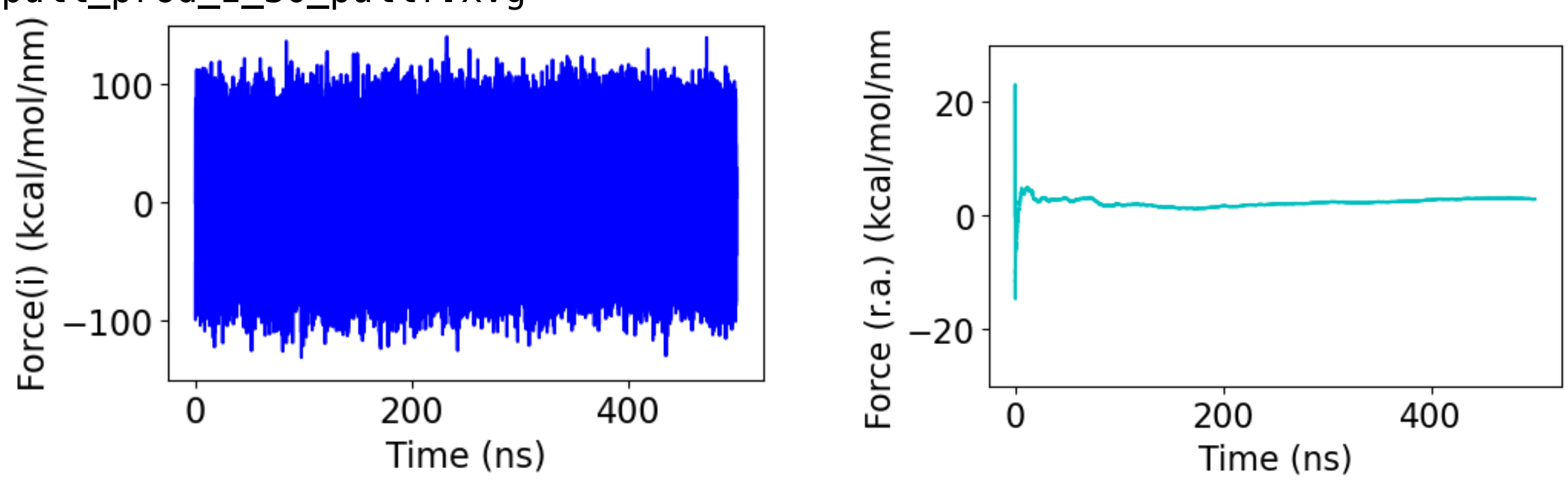

pull_prod_z_31_pullf.xvg

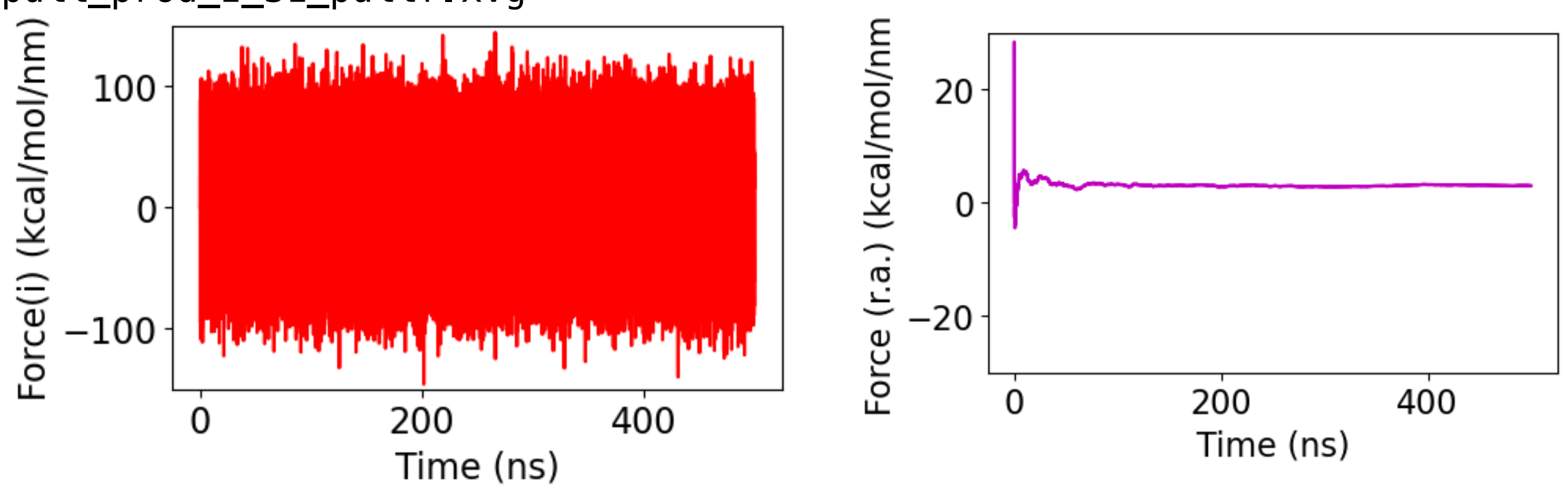

pull_prod_z_32_pullf.xvg

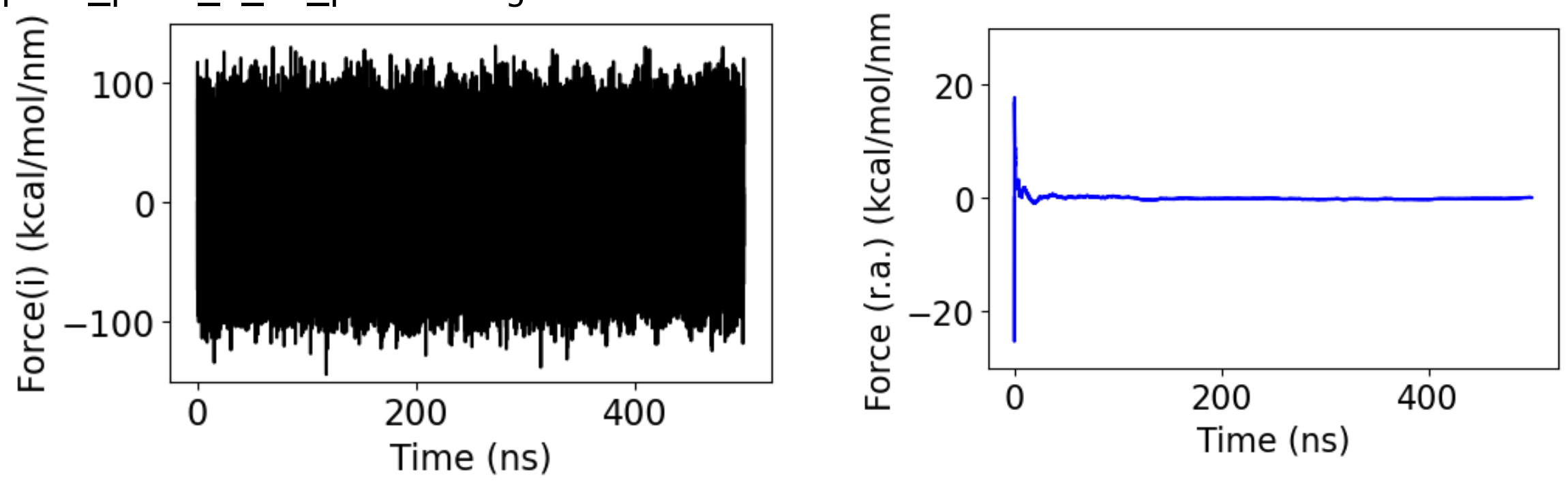

pull_prod_z_33_pullf.xvg

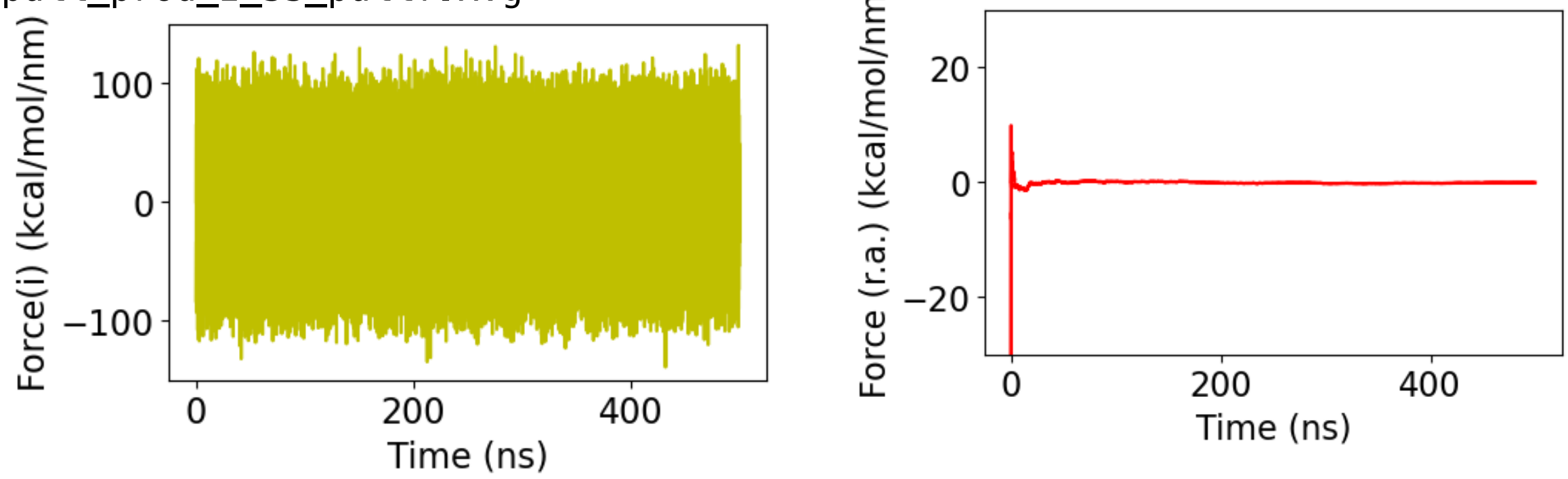

pull_prod_z_34_pullf.xvg

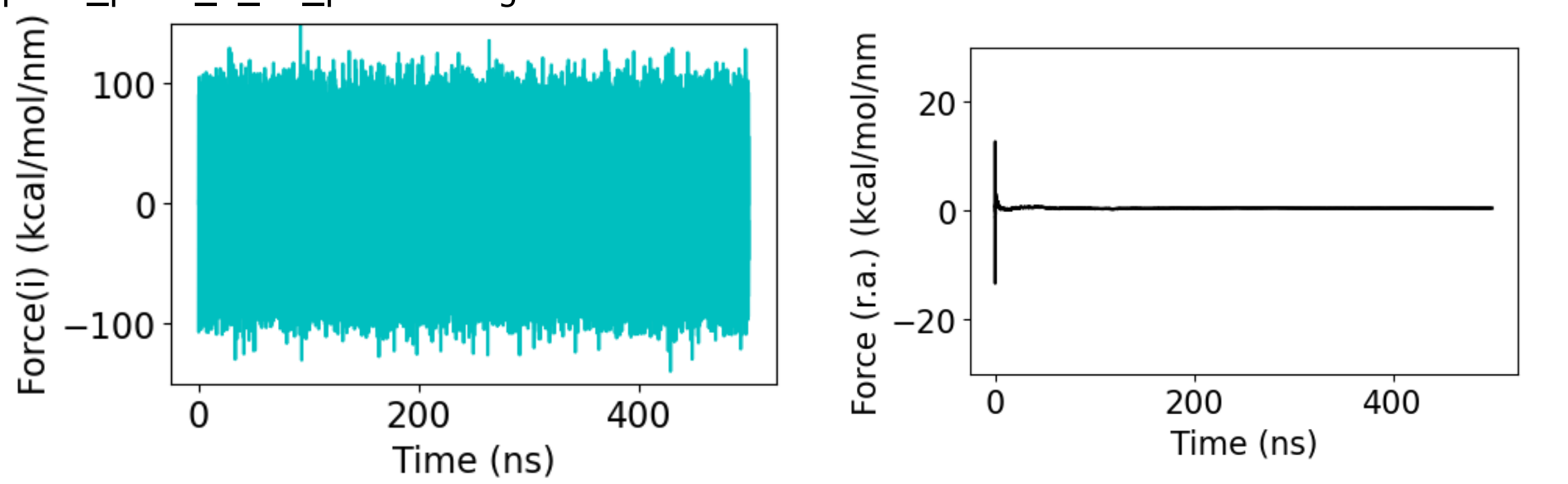

pull_prod_z_35_pullf.xvg

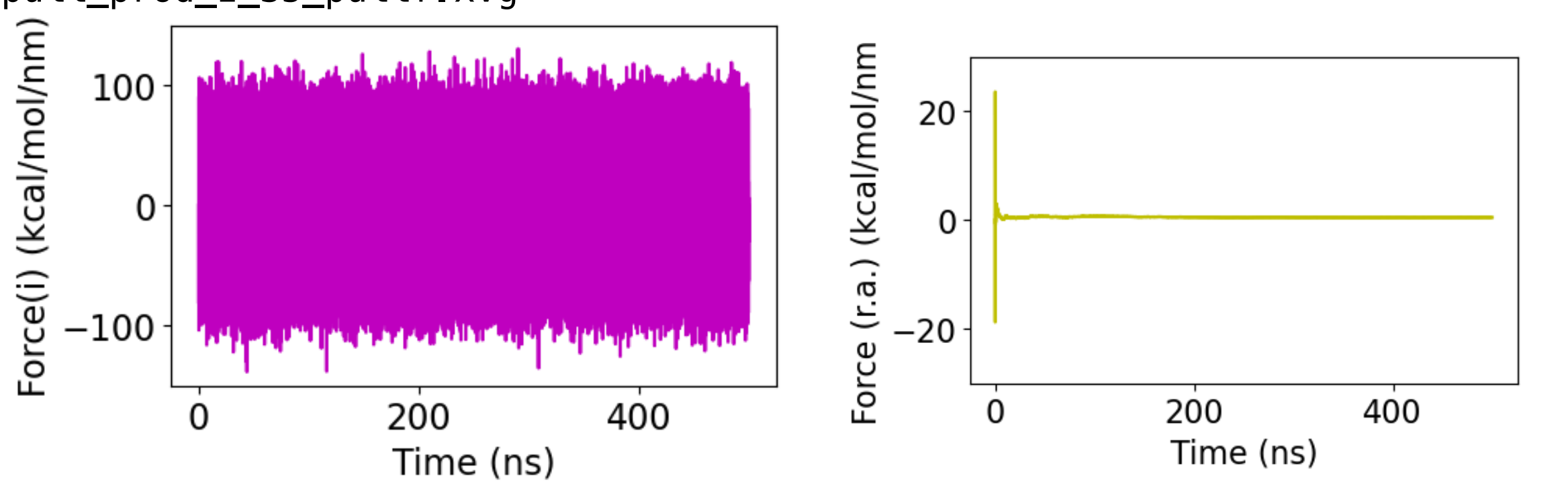

pull_prod_z_36_pullf.xvg

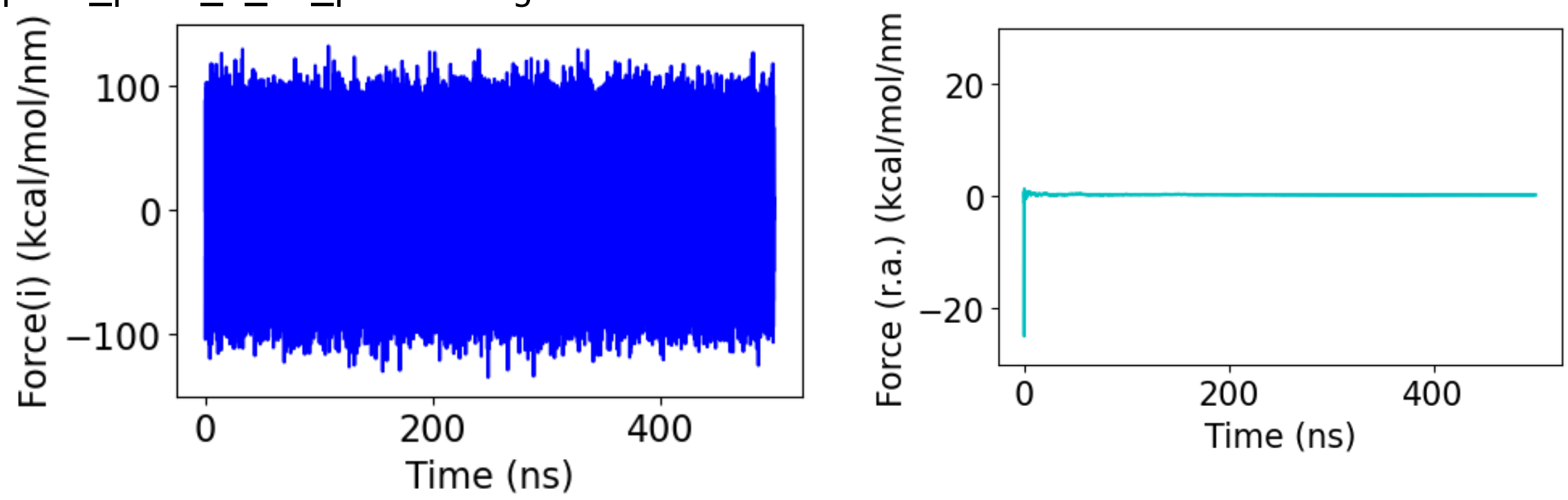

pull_prod_z_37_pullf.xvg

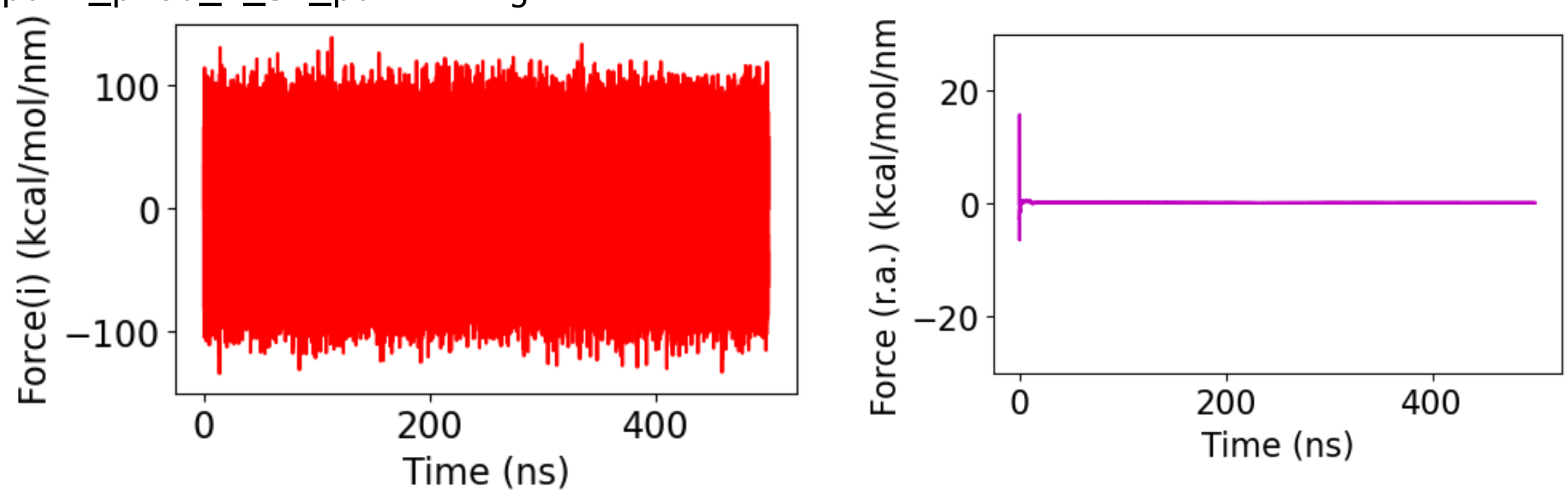

pull_prod_z_38_pullf.xvg

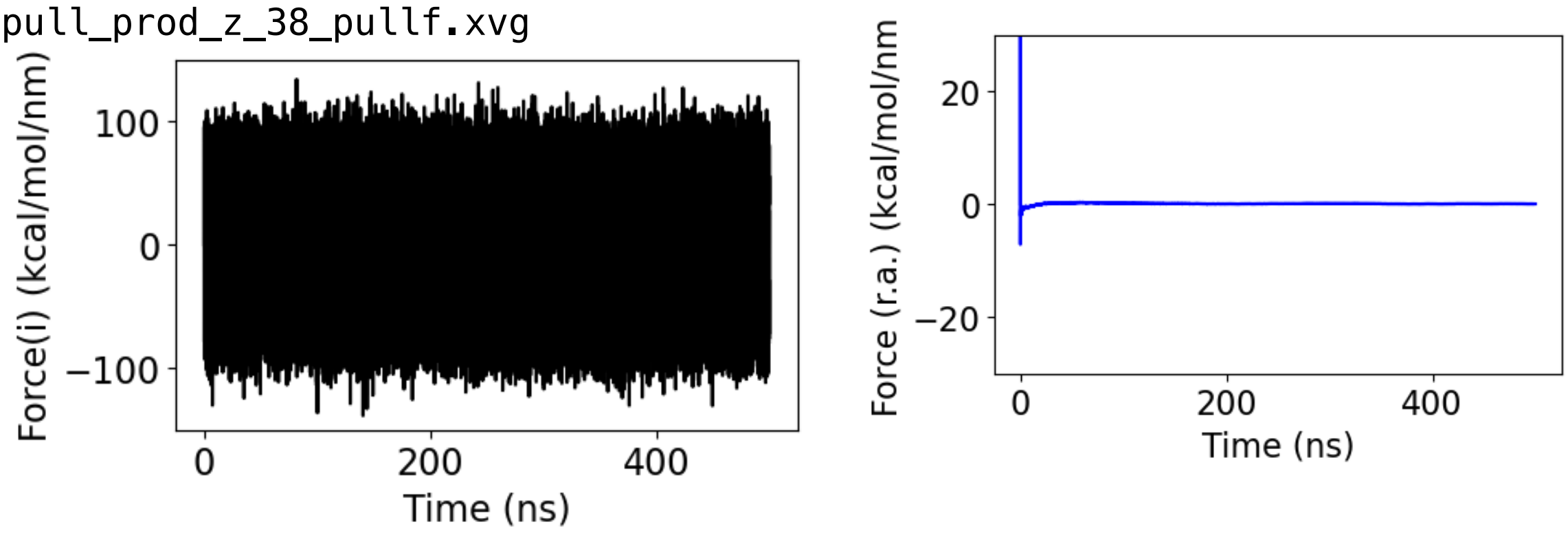

pull_prod_z_39_pullf.xvg

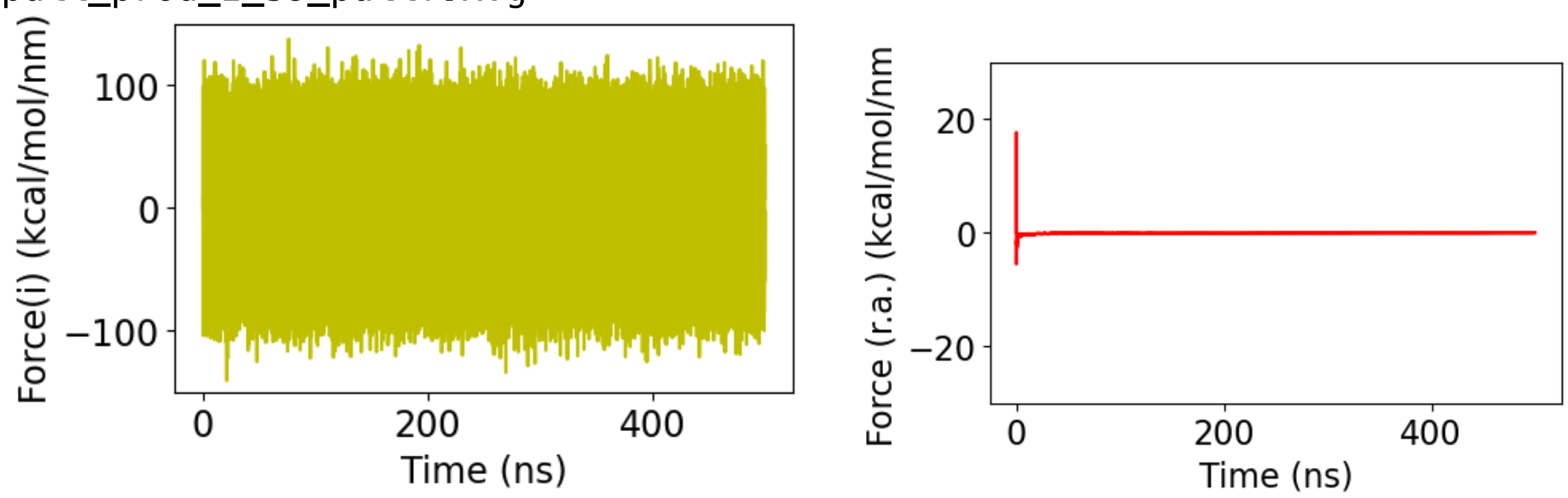

References

Einstein, A. 1905. On the motion of small particles suspended in liquids at rest required by the molecular-kinetic theory of heat. *Ann. Phys.* 17:208.

Español, P., J.A. De La Torre, and D. Duque-Zumajo. 2019. Solution to the plateau problem in the Green-Kubo formula. *Phys. Rev. E*. 99:022126.

Fitzgerald, J.E., R.M. Venable, R.W. Pastor, and E.R. Lyman. 2023. Surface viscosities of lipid bilayers determined from equilibrium molecular dynamics simulations. *Biophys. J.* 122:1094–1104.

Green, M.S. 1954. Markoff random processes and the statistical mechanics of time-dependent phenomena. II. Irreversible processes in fluids. *J. Chem. Phys.* 22:398–413.

Harris, J., C. Chipot, and B. Roux. 2024. Statistical Mechanical Theories of Membrane Permeability. *J. Phys. Chem. B*.

Kluyver, T., B. Ragan-Kelley, F. Pérez, B. Granger, M. Bussonnier, J. Frederic, K. Kelley, J. Hamrick, J. Grout, and S. Corlay. 2016. Jupyter Notebooks–a publishing format for reproducible computational workflows. *In* Positioning and power in academic publishing: Players, agents and agendas. IOS press. 87–90.

Kubo, R. 1957. Statistical-mechanical theory of irreversible processes. I. General theory and simple applications to magnetic and conduction problems. *J. Physical Soc. Japan*. 12:570–586.

Rossum, V. 2009. Python 3 reference manual. *(No Title)*.

Seabold, S., and J. Perktold. 2010. Statsmodels: econometric and statistical modeling with python. *SciPy*. 7:92–96.
