# Supplementary material for: Estimating Permeabilities from Biased Molecular Dynamics with a Green–Kubo Formalism for Transport across the Blood-Brain Barrier
Source: J Chem Inf Model. 2026 Jul 21;66(15):9495–506. doi: 10.1021/acs.jcim.6c01624 (PMC13471738; doi:10.1021/acs.jcim.6c01624)
Supplement: Supplementary file 2 [file ci6c01624_si_001.zip › Caffeine_files_SI.zip!/Parameters/Version2/caff_gromacs/charmm36.ff/forcefield.doc]

CHARMM36 all-atom force field (November 2018)

*******************************************************************************

*	CHARMM36 port writted by	*

*	E. Prabhu Raman, Justin A. Lemkul, Robert Best	*

*	and Alexander D. MacKerell, Jr.	*

*	CHARMM force field homepage:	*

*	www.mackerell.umaryland.edu/CHARMM_ff_params.html	*

*******************************************************************************

Parameters taken from CHARMM36 and CGenFF 4.1

Included are topologies and parameters from

top_all36_prot.rtf	par_all36m_prot.prm

toppar_all36_prot_heme.str

toppar_all36_prot_mod_d_aminoacids.str

toppar_all36_prot_modify_res.str

toppar_all36_prot_retinol.str

top_all36_na.rtf	par_all36_na.prm

toppar_all36_na_modifications.str

toppar_all36_na_nad_ppi.str

toppar_all36_na_rna_modified.str

top_all36_lipid.rtf	par_all36_lipid.prm

toppar_all36_lipid_bacterial.str

toppar_all36_lipid_cardiolipin.str

toppar_all36_lipid_cholesterol.str

toppar_all36_lipid_detergent.str

toppar_all36_lipid_inositol.str

toppar_all36_lipid_miscellaneous.str

toppar_all36_lipid_prot.str

toppar_all36_lipid_sphingo.str

toppar_all36_lipid_yeast.str

top_all36_carb.rtf	par_all36_carb.prm

toppar_all36_carb_glycolipid.str

toppar_all36_carb_glycopeptide.str

top_all36_cgenff.rtf	par_all36_cgenff.prm

top_all35_ethers.rtf	par_all35_ethers.prm

toppar_water_ions.str

top_all22_prot_metals.inp	par_all22_prot_metals.inp

top_silicates.inp	par_silicates.inp

References:

** Proteins

! CHARMM36m

Huang, J., Rauscher, S., Nawrocki, G., Ran, T., Feig, M., de Groot, B.L.,

Grubmuller, H., and MacKerell, Jr., A.D. CHARMM36m: an improved force field

for folded and intrinsically disordered proteins, Nature Methods, 2016, DOI:

10.1038/nmeth.4067.

! CHARMM36

Best, R. B. et al. Optimization of the Additive CHARMM All-Atom Protein

Force Field Targeting Improved Sampling of the Backbone phi, psi and Side-Chain

chi1 and chi2 Dihedral Angles, Journal of Chemical Theory and Computation,

2012, 8: 3257-3273.

MacKerell, Jr., A.D., Feig, M., Brooks, III, C.L. Extending the

treatment of backbone energetics in protein force fields: limitations

of gas-phase quantum mechanics in reproducing protein conformational

distributions in molecular dynamics simulations, Journal of

Computational Chemistry, 2004, 25: 1400-1415.

MacKerell, Jr., A.D., et al. All-atom empirical potential for

molecular modeling and dynamics Studies of proteins, Journal of

Physical Chemistry B, 1998, 102, 3586-3616.

! phosphotyrosine

Feng, M.-H., Philippopoulos, M., MacKerell, Jr., A.D. and Lim, C.

Structural Characterization of the Phosphotyrosine Binding Region of a

High-Affinity aSH2 Domain-Phosphopeptide Complex by Molecular Dynamics

Simulation and Chemical Shift Calculations. Journal of the American

Chemical Society, 1996, 118: 11265-11277.

** Lipids

Klauda, J. B. et al. Improving the CHARMM Force Field for Polyunsaturated

Fatty Acid Chains, Journal of Physical Chemistry B, 2012, 116: 9424-9431.

Klauda, J. B. et al. Update of the CHARMM All-Atom Additive Force Field

for Lipids: Validation on Six Lipid Types, Journal of Physical Chemistry B,

2010, 114: 7830-7843.

Feller, S. and MacKerell, Jr., A.D. An Improved Empirical Potential

Energy Function for Molecular Simulations of Phospholipids, Journal

of Physical Chemistry B, 2000, 104: 7510-7515.

! cholesterol

Lim, J.B., Rogaski, B. and Klauda, J.B. Update of the Cholesterol Force Field

Parameters in CHARMM, 2012, 116: 203-210.

! bacterial lipids

Lim, J.B. & Klauda, J.B. Branching at the Iso- and Anteiso- Positions in Complex Chlamydia

Membranes: A Molecular Dynamics Study. Biochimica et Biophysica Acta (BBA) - Biomembranes,

2011, 1808: 323-331.

Pandit, K.R. & Klauda, J.B. Membrane models of E. coli containing cyclic moieties in

the aliphatic lipid chain. Biochimica et Biophysica Acta (BBA) - Biomembranes

1818:1205-1210 (2012).

! sphingomyelin

Venable, R.M., Sodt, A.J., Rogaski, B., Rui, H., Hatcher, E., MacKerell, Jr., A.D.,

Pastor, R.W., and Klauda, J.B. CHARMM All-Atom Additive Force Field for Sphingomyelin:

Elucidation of Hydrogen Bonding and of Positive Curvature. Biophysical Journal, 2014,

107: 134-145.

** Nucleic Acids

! Modified RNA nucleotides

Xu, Y., Vanommeslaeghe, K., Aleksandrov, A., MacKerell, Jr., A.D., and

Nilsson, L. Additive CHARMM Force Field for Naturally Occurring Modified

Ribonucleotides, Journal of Computational Chemistry, 2016, 37: 896-912.

! CHARMM36

Denning, E.J., Priyakumar, U.D., Nilsson, L., and MacKerell Jr., A.D.,

"Impact of 2'-hydroxyl sampling on the conformational properties of

RNA: Update of the CHARMM all-atom additive force field for RNA,"

JCC, 32: 1929-1943, 2011, PMC3082605

Hart, K., Foloppe, N., Baker, C.M., Denning, E.J., Nilsson, L.

and MacKerell Jr., A.D. "Optimization of the CHARMM additive force

field for DNA: Improved treatment of the BI/BII conformational

equilibrium," JCTC, 8:348-362, 2012, PMC3285246

! NAD, PPI, etc.

Pavelites, J.J., Bash, P.A., Gao, J., and MacKerell, Jr., A.D.

A Molecular Mechanics Force Field for NAD+, NADH and the

Pyrophosphate Groups of Nucleotides, Journal of Computational

Chemistry, 1997, 18: 221-239.

! Reactive RNA intermediates

Mayaan, E., Moser, A., MacKerell, Jr., A.D., and York, D.M. CHARMM force field

parameters for simulation of reactive intermediates in native and

thio-substituted ribozymes. Journal of Computational Chemistry, 2007, 28:495-507.

! LNA and PNA

Wang, P., Nicklaus, M.C., Marquez, V.E., Brank, A.S.,

Christman, J., Banavali, N.K. and MacKerell, Jr., A.D., Use of

Oligodeoxyribonucleotides with Conformationally Constrained

Abasic Sugar Targets to Probe the Mechanism of Base Flipping by

HhaI DNA (Cytosine C5)-Methyltransferase. Journal of the

American Chemical Society, 2000, 122:12422-12434

Hartono, YD, Xu, Y, Karshikoff, A, Nilsson, L, Villa, A. Modeling pKa

Shift in DNA Triplexes Containing Locked Nucleic Acids. J Chem Inf

Model, 58:773-783, 2018, doi: 10.1021/acs.jcim.7b00741

Jasinski, M., Feig, M., Trylska, J. Improved Force

Fields for Peptide Nucleic Acids with Optimized Backbone Torsion

Parameters. J. Chem. Theory Comput., 14: 3603-3620, 2018,

doi: 10.1021/acs/jctc.8b00291

** Carbohydrates

! pyranose monosaccharides

Guvench, O., Greene, S.N., Kamath, G., Brady, J.W., Venable, R.M.,

Pastor, R.W., MacKerell, Jr., A.D. "Additive empirical force field for

hexopyranose monosaccharides," Journal of Computational Chemistry, 29:

2543-2564, 2008. PMID: 18470966

! linear sugars, sugar alcohols, and inositol

Hatcher, E., Guvench, O., and MacKerell, Jr., A.D. "CHARMM Additive

All-Atom Force Field for Acyclic Polyalcohols, Acyclic Carbohydrates

and Inositol," Journal of Chemical Theory and Computation, 5:

1315-1327, 2009, DOI: 10.1021/ct9000608.

! hexopyranose glycosidic linkages

Guvench, O., Hatcher, E. R., Venable, R. M., Pastor, R. W., MacKerell, Jr.,

A. D. "Additive Empirical CHARMM Force Field for glycosyl linked

hexopyranoses," Journal of Chemical Theory and Computation, 5,

2353-2370, 2009, DOI: 10.1021/ct900242e

! furanose monosaccharides

Hatcher, E. R.; Guvench, O. and MacKerell, Jr., A.D.

"CHARMM Additive All-Atom Force Field for Aldopentofuranose

Carbohydrates and Fructofuranose." Journal of Physical Chemistry B.

113:12466-76, 2009, PMID: 19694450

! glycosidic linkages involving furanoses

Raman, E. P., Guvench, O., MacKerell, Jr., A.D., "CHARMM Additive All-Atom

Force Field for Glycosidic Linkages in Carbohydrates Involving Furanoses,"

Journal of Physical Chemistry B, 114: 12981-12994, 2010, PMID: 20845956

! carbohydrate derivatives and glycosidic linkages for glycoproteins

Guvench, O., Mallajosyula, S.S. Raman, E.P., Hatcher, E. Vanommeslaeghe, K.,

Foster, T.J., Jamison II, F.W., and MacKerell, Jr., A.D. "CHARMM additive

all-atom force field for carbohydrate derivatives and their utility in

polysaccharide and carbohydrate-protein modeling," JCTC, 7: 3162-3180, 2011.

PMC3224046

! O-glycan linkages

Mallajosyula, S. S. and MacKerell, Jr., A.D., "Influence of Solvent and

Intramolecular Hydrogen Bonding on the Conformational Properties of O-Linked

Glycopeptides," Journal of Physical Chemistry B, 115: 11215-11229, 2011

PMC3224046

! Phosphates and sulfates

Mallajosyula, S. S.; Guvench, O.; Hatcher, E. R. and MacKerell, Jr., A.D.,

CHARMM Additive All-Atom Force Field for Phosphate and Sufate Linkages in

carbohydrates" JCTC, 8: 759-776, 2012.

** CHARMM General Force Field

Vanommeslaeghe, K. et al. CHARMM General Force Field: A Force Field for Drug-Like

Molecules Compatible with the CHARMM All-Atom Additive Biological Force Fields,

Journal of Computational Chemistry, 2010, 31: 671-690.

Yu, W., He, X., Vanommeslaeghe, K., and MacKerell, Jr., A.D. Extension of the CHARMM

general force field to sulfonyl-containing compounds and its utility in biomolecular

simulations, J. Comput. Chem. 2012, 33, 2451-2468.

! Halogens with lone pairs

Gutierrez, I.S., Lin, F.-Y., Vanommeslaeghe, K., Lemkul, J.A., Armacost, K.A.,

Brooks III, C.L., and MacKerell, Jr., A.D. Parametrization of halogen bonds in the CHARMM

general force field: Improved treatment of ligand-protein interactions, Bioorganic &

Medicinal Chemistry, 2016, 24: 4812-4825.

** Silicates

Lopes, P.E.M., Murashov, V., Tazi, M., Demchuk, E. and MacKerell, Jr., A.D.

Development of an Empirical Force Field for Silica. Application to the Quartz-Water Interface,

Journal of Physcial Chemistry B, 2006, 110: 2782-2792.

** Ions

! Zn

Stote, R.H. and Karplus, M. Zinc Binding in Proteins and

Solution: A Simple but Accurate Nonbonded Representation, PROTEINS:

Structure, Function, and Genetics, 1995, 23:12-31.

! Neutral FCC metals (AG, AL, AU, CU, NI, PB, PD, PT)

Heinz, H., Vaia, R.A., Farmer, B.L., and Naik, R.R. Accurate Simulation

of Surfaces and Interfaces of Face-Centered Cubic Metals Using 12-6 and 9-6

Lennard-Jones Potentials, Journal of Physical Chemistry C, 2008, 112, 17281-17290.

** Implementation of CHARMM in GROMACS

Bjelkmar, P., Larsson, P., Cuendet, M. A, Bess, B., Lindahl, E.

Implementation of the CHARMM force field in GROMACS: Analysis of protein

stability effects from correction maps, virtual interaction sites, and

water models, Journal of Chemical Theory and Computation, 2010, 6: 459-466.
